# Precemtabart tocentecan, an anti-CEACAM5 antibody–drug conjugate, in metastatic colorectal cancer: a phase 1 trial

**DOI:** 10.1038/s41591-025-03843-z

**Published:** 2025-07-30

**Authors:** Scott Kopetz, Valentina Boni, Ken Kato, Kanwal P. S. Raghav, Maria Vieito, Athanasios Pallis, Christina Habermehl, Abdul Siddiqui, Perrine Courlet, Willem Sloot, Sabine Raab-Westphal, Felix Hart, Ildefonso Rodriguez-Rivera

**Affiliations:** 1 The University of Texas, MD Anderson Cancer Center, Houston, TX USA; 2 NEXT Oncology, Universitary Hospital Quironsalud, Madrid, Spain; 3 National Cancer Center Hospital, Tokyo, Japan; 4 Department of Medical Oncology, Vall d’Hebron University Hospital, Barcelona, Spain; 5 Merck Santé S.A.S., Lyon, France, an affiliate of Merck KGaA, Darmstadt, Germany; 6 the healthcare business of Merck KGaA, Darmstadt, Germany; 7 Merck Serono Ltd., Feltham, UK, an affiliate of Merck KGaA, Darmstadt, Germany; 8 Merck Institute of Pharmacometrics, Lausanne, Switzerland, an affiliate of Merck KGaA, Darmstadt, Germany; 9 NEXT Oncology, San Antonio, TX USA

**Keywords:** Colorectal cancer, Drug development, Targeted therapies

## Abstract

CEACAM5, a cell surface protein, is overexpressed in colorectal cancer (CRC). Precemtabart tocentecan (Precem-TcT, previously M9140) is an anti-CEACAM5 antibody–drug conjugate with the topoisomerase 1 inhibitor exatecan as payload. Precem-TcT demonstrated strong antitumor activity and potent bystander activity in preclinical models. Its toxicity profile in cynomolgus monkeys was consistent with that of exatecan. In the dose-escalation stage of the phase 1 trial of Precem-TcT (PROCEADE-CRC-01), 40 heavily pretreated patients with irinotecan-refractory metastatic CRC received Precem-TcT every 3 weeks across seven dose levels (DLs, 0.6–3.2 mg kg^−1^). Primary endpoints were dose-limiting toxicities (DLTs), adverse events and preliminary clinical activity to establish the recommended dose(s) for expansion (RDEs). Secondary endpoints included pharmacokinetic parameters, objective response and median progression-free survival (mPFS). At the planned, end-of-dose-escalation analysis with extended follow-up (cutoff: 1 August 2024), seven patients had experienced DLTs, primarily hematologic events at 3.0 mg kg^−1^ and 3.2 mg kg^−1^. A treatment-related death, also deemed disease related, was reported in a patient with multiple comorbidities and grade 3 obesity. The maximum tolerated dose was determined to be 2.8 mg kg^−1^ every 3 weeks. Total and conjugated antibody pharmacokinetic profiles largely overlapped, indicating stability of the linker–payload (β-glucuronide–exatecan) in circulation. After a median treatment of 19.1 weeks (range: 1.7–48.3), three of 40 patients (7.5%) had confirmed partial responses (15.0% (6/40) unconfirmed), all at DLs ≥2.4 mg kg^−1^. mPFS was 5.9 months (95% confidence interval: 4.6–7.2); at DLs ≥2.4 mg kg^−1^ (*n* = 34), mPFS was 6.7 months (95% confidence interval: 4.6–8.8). Four patients (10.0%) remained on treatment at cutoff. These early clinical data corroborate preclinical findings, showing predictable safety and encouraging antitumor activity of Precem-TcT at DLs ≥2.4 mg kg^−1^, with no interstitial lung disease or ocular toxicity. The dose-optimization part at the RDEs of 2.4 mg kg^−1^ and 2.8 mg kg^−1^ (both every 3 weeks) in PROCEADE-CRC-01 is ongoing. ClinicalTrials.gov identifier: NCT05464030.

## Abstract (web summary)

On the basis of encouraging preclinical activity, an anti-CEACAM5 antibody–drug conjugate with a topoisomerase 1 inhibitor payload is being tested in an ongoing phase 1 trial where recommended doses for further testing have been identified and an unconfirmed overall response rate of 15% was observed in patients with metastatic colorectal cancer.

## Main

CRC represents 10% of all cancer diagnoses and is the second most common cause of cancer-related fatalities globally^1^. Nearly 1.9 million new CRC cases were documented in 2022, and almost 1 million deaths were attributed to the disease^2^. At diagnosis, 20% of patients with CRC present with metastatic CRC (mCRC)^3^. In addition, approximately 40% of patients who are initially diagnosed with localized disease eventually progress to metastatic disease^4–6^. The outlook for patients with mCRC remains poor, with a 5-year survival rate of roughly 15%^7,8^.

In the initial lines of therapy, the standard of care (SoC) for mCRC involves a systemic chemotherapy backbone comprising two-drug or three-drug regimens (fluoropyrimidine, irinotecan, oxaliplatin and their combinations) with or without targeted therapy (anti-vascular endothelial growth factor (VEGF)/anti-epidermal growth factor receptor (EGFR) monoclonal antibodies), tailored based on the location and mutational profile of the tumor as well as the patient’s clinical profile^9–11^. Despite receiving the SoC, most patients eventually develop progressive disease, with approximately 40% receiving at least three lines of treatment (3L+)^12,13^. Currently, four therapies are approved by the US Food and Drug Administration (FDA) and recommended by National Comprehensive Cancer Network guidelines for the treatment of 3L+ mCRC: regorafenib, trifluridine/tipiracil alone or combined with bevacizumab and fruquintinib^14^. However, the overall efficacy of current treatment options in later lines is limited, with a median overall survival of less than 12 months, response rates below 5% and mPFS of 1.9–5.6 months, suggesting a substantial unmet need for novel and more efficacious therapies for 3L+ mCRC^15–17^.

Antibody–drug conjugates (ADCs) represent a rapidly evolving therapeutic class designed to deliver cytotoxic payloads directly to tumor cells by conjugating them to a tumor-specific monoclonal antibody through a linker. Currently, eight ADCs are approved by the FDA for the treatment of solid tumors, with indications including breast, urothelial, non-small cell lung and ovarian cancers; however, no ADCs are approved for the treatment of CRC^18–20^. CEACAM5, a cell surface glycoprotein that modulates cell adhesion, differentiation and proliferation, shows limited expression on healthy adult cells but is overexpressed in CRC and other solid tumors^21–23^. With approximately 90% of CRCs overexpressing CEACAM5 (ref. ^24^), it is a rational therapeutic target for an ADC.

Precem-TcT (previously M9140) is an innovative anti-CEACAM5 ADC with the topoisomerase 1 (TOP1) inhibitor exatecan as its payload^23^. The cleavable β-glucuronide linker, which connects the Precem-TcT antibody backbone to exatecan, is highly hydrophilic and stable in circulation (drug-to-antibody ratio = 8). This linker is cleaved by β-glucuronidase, an enzyme present in lysosomes and the tumor interstitium^25,26^. Initial preclinical data indicate that Precem-TcT can mediate a potent ‘bystander effect’, which is the ability to induce cell death in neighboring cells via the diffusion of the cytotoxic payload across cell membranes. This effect is particularly advantageous for treating tumors with heterogeneous target expression (for example, CEACAM5), as it can also affect target-negative tumor cells^23^.

Here we present the preclinical evidence that encouraged the initiation of the first-in-human study of Precem-TcT—PROCEADE-CRC-01 (NCT05464030)—in heavily pretreated patients with mCRC and the first clinical data from this study.

## Results

### Preclinical properties of Precem-TcT

#### Inhibition of cancer cell line viability by Precem-TcT

**Fig. 1:**
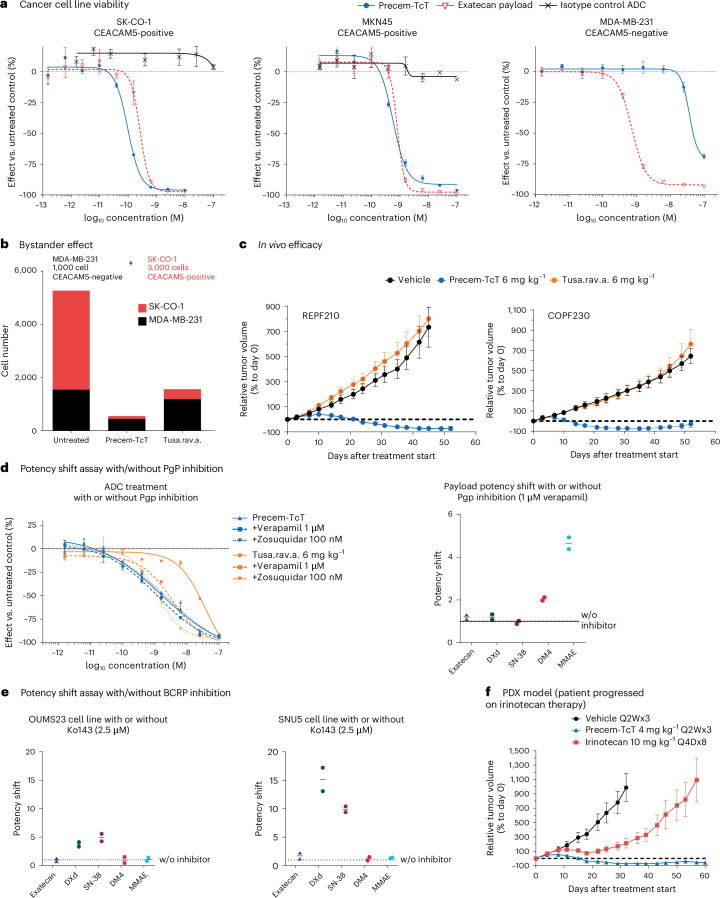
Preclinical data overview. **a**, Selective killing of CEACAM5-positive cancer cell lines compared to CEACAM5-negative cell line. Data are mean ± s.d. (*n* = 3) from a representative replicate of three independent experiments. **b**, Bystander effect of Precem-TcT and tusa.rav.a. in in vitro co-culture experiments (1 nM ADC). Data are mean from a representative replicate of two independent experiments. **c**, In vivo efficacy of Precem-TcT in comparison to tusa.rav.a. in two CRC PDX models: REPF210 and COPF230; data are mean ± s.e.m. from *n* = 6 mice per treatment group and *n* = 5 mice per treatment group, respectively. **d**, Potency shift assay using Precem-TcT in comparison to tusa.rav.a. or different payloads with and without Pgp inhibition using verapamil/zosuquidar (LS513 cell line). Left panel: data are mean ± s.e.m. (*n* = 2) from a representative replicate of two independent experiments; right panel: each data point represents an independent experiment and is the mean of two technical replicates; horizontal black line represents the mean of the two data points. **e**, Potency shift assay using payloads with and without BCRP inhibition in OUMS23 cell line (left) and SNU5 cell line (right). Each data point represents an independent experiment and is the mean of two technical replicates. Horizontal black line represents the mean of the two data points. **f**, Antitumor efficacy of Precem-TcT compared to that of irinotecan in the human CRC PDX model CXF4102, derived from a patient with CRC previously treated with irinotecan-based SoC (FOLFIRI/Avastin). Data are mean ± s.e.m. from *n* = 6 mice per treatment group. w/o, without. Source data

Precem-TcT demonstrated potent inhibition of cancer cell growth in cell lines expressing the CEACAM5 protein, specifically SK-CO-1 and MKN-45, with 50% inhibitory concentration (IC_50_) values of 0.09 nM and 0.63 nM, respectively. In contrast, Precem-TcT had minimal effects on the CEACAM5-negative MDA-MB-231 cell line, requiring concentrations greater than 10 nM for any considerable inhibition. Exatecan alone effectively inhibited all three cell lines, indicating that CEACAM5 expression is crucial for the efficacy of Precem-TcT (Fig. 1a). The analog of tusamitamab ravtansine used in our study, tusa.rav.a., inhibited the viability of SK-CO-1 cells with a potency similar to that of Precem-TcT.

#### Bystander effect

**Extended Data Fig. 1:**
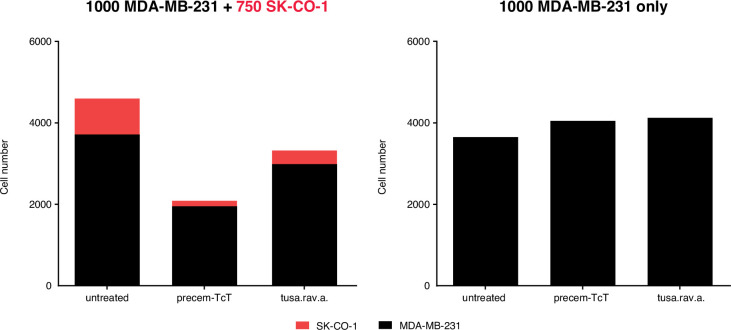
Bystander effect of Precem-TcT and tusa.rav.a. In vitro co-culture experiments (1 nM ADC) with lower number of CEACAM5-positive cells and a control of CEACAM5-negative cells only. Data are mean (n = 2) from a representative replicate of 2 independent experiments.

To evaluate the bystander effect of Precem-TcT, co-culture experiments using CEACAM5-positive and CEACAM5-negative cancer cells were performed. Although it effectively inhibited CEACAM5-positive cells (SK-CO-1) and spared CEACAM5-negative cells (MDA-MB-231) in standard monoculture viability assays, Precem-TcT was able to induce cell death in CEACAM5-negative cells in co-culture experiments when in proximity to CEACAM5-positive cells (Fig. 1b). In contrast, tusa.rav.a. exhibited only minor effects in co-culture experiments. The bystander effect on CEACAM5-negative cells increased with increasing number of CEACAM5-positive cells added in co-culture experiments (Extended Data Fig. 1).

#### Antitumor efficacy in CRC patient-derived xenograft mouse models

In patient-derived xenograft (PDX) mouse models of CRC with high CEACAM5 expression, a single administration of Precem-TcT (6 mg kg^−1^) resulted in tumor stasis in the COPF230 model (−29% tumor volume on day 52) and tumor regression in the REPF210 model (−72% tumor volume on day 52). In contrast, tusa.rav.a. did not reduce tumor growth compared with vehicle, leading to marked progression in both models (Fig. 1c).

#### Resistance mechanisms

A common mechanism of resistance to anticancer therapies involves membrane drug transporters, such as MDR1 and BCRP, which can expel therapeutic agents, leading to reduced efficacy. To assess the impact of these resistance mechanisms on Precem-TcT and exatecan, potency shift assays were conducted. Notably, the efficacy of Precem-TcT and exatecan was not affected by MDR1 inhibition in LS513 cell lines, unlike tusa.rav.a. and other payloads (Fig. 1d). Similarly, the potency of exatecan remained unaffected by BCRP inhibition in OUMS23 and SNU5 cell lines, indicating that exatecan is not prone to BCRP-mediated resistance (Fig. 1e).

#### Evaluation in a post-irinotecan setting

In a CRC PDX mouse model (CXF4102) derived from a patient who had previously progressed on irinotecan, treatment with Precem-TcT (4 mg kg^−1^, administered every 2 weeks for three doses) resulted in substantial tumor regression (−73% tumor volume on day 32), with sustained tumor-inhibiting effect until the end of the study (−57% tumor volume on day 60). In contrast, irinotecan (10 mg kg^−1^, administered every 4 days for eight doses) achieved tumor stasis as the best response, followed by subsequent progression (Fig. 1f).

#### Toxicology results in cynomolgus monkeys

Repeat-dose toxicity studies in cynomolgus monkeys revealed dose-dependent effects of Precem-TcT on hematolymphoid and intestinal systems^27^, consistent with the toxicity profile of exatecan^28–30^. Transient reductions in hematologic parameters (neutrophils and reticulocytes) were observed at 24 mg kg^−1^ or higher every 3 weeks, after each dosing event. In addition, erythrocyte counts gradually declined over time and recovered during the 3-week dosing-free period. The no-observed-adverse-effect level was established at 24 mg kg^−1^, and the maximum tolerable dose (MTD) was established at 30 mg kg^−1^ due to adverse clinical signs and body weight reduction. The difference in toxicity by this small dose increment was determined by a 2.5-fold difference in plasma exatecan exposure driving the effects. After dosing at 30 mg kg^−1^ every 3 weeks for three times, microscopic changes were up to moderate in lymphoid tissues and up to mild in the gastrointestinal tract. Toxicities commonly associated with ADCs, such as interstitial lung disease (ILD) or ocular toxicity, were not observed.

### Phase 1 dose escalation

#### Study design

**Extended Data Fig. 2:**
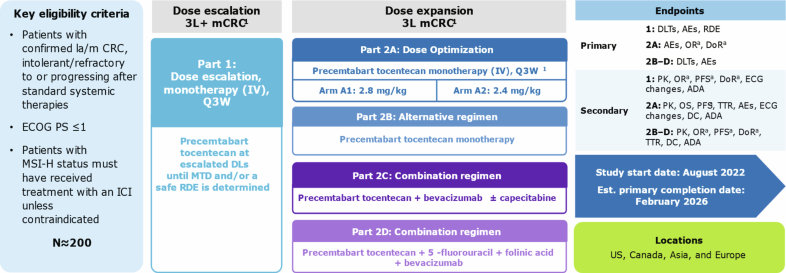
Study design of PROCEADE-CRC-01. 3L, third line; 3 L + , third- or later-line; ADA, anti-drug antibody; AE, adverse event; CRC, colorectal cancer; DC, disease control; DL, dose level; DLT, dose-limiting toxicity; DoR, duration of response; ECG, electrocardiogram; ECOG PS, Eastern Cooperative Oncology Group performance status; Est. estimated; FiH, first-in-human; ICI, immune checkpoint inhibitor; IV, intravenous; la/m, locally advanced/metastatic; MSI-H, microsatellite instability high; MTD, maximum tolerable dose; OR, objective response; OS, overall survival; PFS, progression-free survival; PK, pharmacokinetics; Q2W, every 2 weeks; Q3W, every 3 weeks; RDE, recommended dose for expansion; RECIST v1.1, Response Evaluation Criteria In Solid Tumors version 1.1; TTR, time to response; US, United States. ^a^Assessed by investigator per RECIST v1.1. 1. Kopetz S, et al. *J Clin Oncol*. 2024;42(16_Suppl):3000.

PROCEADE-CRC-01 (NCT05464030) is an ongoing, first-in-human phase 1 evaluation of Precem-TcT in patients with mCRC that is being conducted in the United States, Europe and Japan. The study includes a dose-escalation part (Part 1) and a dose-expansion part (Part 2; not described here). An overview of the PROCEADE-CRC-01 study design is included in Extended Data Fig. 2.

Part 1 included dose cohorts without primary granulocyte colony-stimulating factor (G-CSF) prophylaxis (Part 1A) as well as a cohort with patients who received primary G-CSF prophylaxis (Part 1B; data not reported separately).

Adult patients with a confirmed diagnosis of locally advanced/metastatic CRC, an Eastern Cooperative Oncology Group performance status (ECOG PS) of ≤1 and adequate hematologic, hepatic and renal function (creatinine clearance (CrCl) ≥60 ml min^−1^) were included in the study. Additional eligibility criteria included prior treatment with fluoropyrimidine, irinotecan, oxaliplatin-based therapy, an anti-EGFR agent (for *RAS*/*BRAF* wild-type patients), an anti-VEGF agent or immune checkpoint inhibitors (for microsatellite instability-high (MSI-H) patients). Patients were required to have archived formalin-fixed, paraffin-embedded (FFPE) tumor tissue samples or a fresh biopsy available before study initiation. Patients were included regardless of CEACAM5 expression levels.

Primary endpoints included DLTs, adverse events, pharmacokinetic profile and preliminary clinical activity to establish the RDEs. Secondary endpoints included assessment of pharmacokinetic parameters, objective response, duration of response, mPFS and antidrug antibodies (ADAs). In addition, the disease control rate (DCR, a post hoc endpoint) at 12 weeks was also assessed.

#### Patient disposition and baseline characteristics

**Fig. 2:**
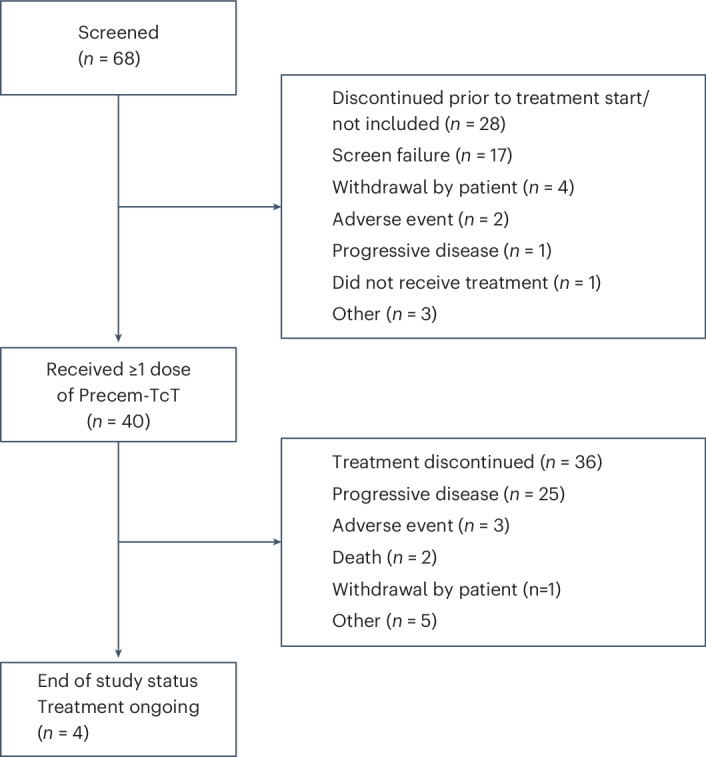
Patient disposition. CONSORT diagram for the PROCEADE-CRC-01 dose escalation study.

The dose-escalation part of the study was conducted from August 2022 to February 2024, with the first patient dosed on 4 August 2022 and the last patient dosed on 3 January 2024. An overview of the patient disposition is included in Fig. 2. In a planned end-of-dose-escalation analysis that was repeated with a longer follow-up (data cutoff: 1 August 2024), a total of 40 patients from the United States, Europe and Japan had been treated with Precem-TcT across seven DLs: 0.6 mg kg^−1^ and 1.2 mg kg^−1^ (*n* = 3 each), 2.4 mg kg^−1^ (*n* = 7), 2.6 mg kg^−1^ (*n* = 4), 2.8 mg kg^−1^ (*n* = 12), 3.0 mg kg^−1^ (*n* = 4), 3.2 mg kg^−1^ (*n* = 4) and 3.2 mg kg^−1^ with primary G-CSF prophylaxis (*n* = 3). Most patients were heavily pretreated, with 80% having received three or more prior lines of treatment, and all had previously received irinotecan (Table 1). The median time since initial cancer diagnosis was 3.4 years (range: 0.9–12.0).

**Table 1:** Baseline and demographic characteristics

Baseline characteristics		All patients (*N* = 40)
Age, mean (s.d.), years		58.2 (10.98)
Sex, *n* (%)	Male	20 (50.0)
	Female	20 (50.0)
Race, *n* (%)	White	26 (65.0)
	Asian	11 (27.5)
	Black or African American	2 (5.0)
	Other	1 (2.5)
BMI, mean (s.d.), kg m^−2^		26.8 (7.28)
Site of primary tumor^a^, *n* (%)	Colon	31 (77.5)^b^
	Rectum	8 (20.0)
ECOG PS	0	15 (37.5)
	1	25 (62.5)
Time since initial cancer diagnosis, median (minimum, maximum), years		3.4 (0.9, 12.0)
Number of previous systemic anticancer therapies, *n* (%)	2	8 (20.0)
	3	16 (40.0)
	≥4	16 (40.0)
Major treatments before trial initiation, *n* (%)	Cetuximab	8 (20.0)
	Capecitabine	12 (30.0)
	Panitumumab	15 (37.5)
	Bevacizumab	37 (92.5)
	Fluorouracil	38 (95.0)
	Oxaliplatin	39 (97.5)
	Irinotecan	40 (100.0)
*KRAS* mutation status	Yes	19 (47.5)
	No	17(42.5)
	Unknown	4 (10.0)
*NRAS* mutation status	Yes	3 (7.5)
	No	31(77.5)
	Unknown	6 (15.0)
*BRAF* mutation status	Yes	5 (12.5)
	No	28 (70.0)
	Unknown	7 (17.5)

^a^Data for one patient were missing.

^b^Left colon: *n* = 19 (47.5%); right colon: *n* = 8 (20.0%); sigmoid colon: *n* = 2 (5.0%).

#### DLTs

Overall, seven patients experienced DLTs (*n* = 1 each at 2.4 mg kg^−1^, 2.8 mg kg^−1^ and 3.0 mg kg^−1^; *n* = 4 at 3.2 mg kg^−1^) (Table 2). Most DLTs were hematologic adverse events. One patient with multiple comorbidities and grade 3 obesity died due to sepsis at DL 2.8 mg kg^−1^. The patient’s clinical course was further complicated by a concurrent ischemic cerebrovascular event, and the septic event was attributed by the treating physician to both Precem-TcT and the underlying disease. The patient had grade 4 neutropenia (neutrophil nadir: zero cells per mm^3^) and a progressive fever with a maximum temperature of 101.7 °F. Clinical and laboratory findings suggested a urinary tract infection as the cause of fever. No other septic events occurred in any patient at any DL.

**Table 2:** Number of patients with DLTs at the tested DLs (all every 3 weeks)

DLT event (SMC decision)	0.6 mg kg^−1^ (*n* = 3)	1.2 mg kg^−1^ (*n* = 3)	2.4 mg kg^−1^ (*n* = 7)	2.6 mg kg^−1^ (*n* = 4)	2.8 mg kg^−1^ (*n* = 12)	3.0 mg kg^−1^ (*n* = 4)	3.2 mg kg^−1^ (*n* = 7)^a^	Total (*N* = 40)
Total^b^, *n* (%)	0	0	1 (14.3)	0 (0.0)	1 (8.3)	1 (25.0)	4 (57.1)	7 (17.5)
Anemia	0	0	0	0	0	0	1 (14.3)	1 (2.5)
Febrile neutropenia	0	0	1 (14.3)	0	0	0	2 (28.6)	3 (7.5)
Neutrophil count decreased	0	0	0	0	0	1 (25.0)	1 (14.3)	2 (5.0)
Platelet count decreased	0	0	0	0	0	0	2 (28.6)	2 (5.0)
Sepsis	0	0	0	0	1 (8.3)	0	0	1 (2.5)
Thrombocytopenia	0	0	0	0	0	0	1 (14.3)	1 (2.5)
Administration of G-CSF within the DLT period, *n* (%)	0	0	1 (14.3)	1 (25.0)	1 (8.3)	1 (25.0)	1 (14.3)	5 (12.5)

^a^Includes three patients with primary G-CSF prophylaxis

^b^Includes the total number of patients and not the total number of events.

#### Safety overview

The most frequently (overall frequency, ≥20%) reported grade 3 or higher treatment-emergent adverse events (TEAEs) were neutropenia in 20 patients (50.0%), anemia in 16 patients (40.0%), thrombocytopenia in 13 patients (32.5%) and leukopenia in 12 patients (30.0%) (Table 3). Most of these hematological TEAEs were asymptomatic laboratory abnormalities that self-resolved or resolved with appropriate intervention. Gastrointestinal disorders associated with Precem-TcT were mostly grade 1 and were observed in 28 patients (70%), with nausea (19 (47.5%)), vomiting (12 (30.0%)) and diarrhea (11 (27.5%)) being the most frequently reported events (Supplementary Table 1). Dose reductions due to TEAEs were reported in nine patients, all at DLs ≥2.8 mg kg^−1^ (overall, 22.5%; at DLs ≥2.8 mg kg^−1^, 39.1%). No events of ocular toxicity or ILD were reported. In addition to the grade 5 sepsis (DLT), one additional patient died after gastrointestinal hemorrhage at a DL of 2.8 mg kg^−1^; however, this adverse event was considered unrelated to Precem-TcT by the investigator and, instead, related to the disease under study.

**Table 3:** Most frequently reported grade 3 or higher TEAEs (≥10% patients, overall) by primary System Organ Class and Preferred Term (Safety Analysis Set)

	0.6 mg kg^−1^ (*n* = 3)	1.2 mg kg^−1^ (*n* = 3)	2.4 mg kg^−1^ (*n* = 7)	2.6 mg kg^−1^ (*n* = 4)	2.8 mg kg^−1^ (*n* = 12)	3.0 mg kg^−1^ (*n* = 4)	3.2 mg kg^−1^ (*n* = 7)^a^	Total (*N* = 40)
Patients with ≥1 grade ≥3 TEAEs, *n* (%)	1 (33.3)	1 (33.3)	3 (42.9)	3 (75.0)	9 (75.0)	4 (100.0)	7 (100.0)	28 (70.0)
Grade ≥3 TEAEs reported in ≥10% of patients (total), *n* (%)								
Neutropenia^b^	0	0	3 (42.9)	2 (50.0)	6 (50.0)	3 (75.0)	6 (85.7)	20 (50.0)
Anemia^c^	0	0	2 (28.6)	1 (25.0)	6 (50.0)	1 (25.0)	6 (85.7)	16 (40.0)
Thrombocytopenia^d^	0	0	2 (28.6)	1 (25.0)	3 (25.0)	2 (50.0)	5 (71.4)	13 (32.5)
Leukopenia^e^	0	0	1 (14.3)	1 (25.0)	4 (33.3)	1 (25.0)	5 (71.4)	12 (30.0)
Lymphopenia^f^	0	1 (33.3)	1 (14.3)	0	2 (16.7)	2 (50.0)	3 (42.9)	9 (22.5)

All patients were tested at every-3-week dosing.

^a^Includes three patients with primary G-CSF prophylaxis

^b^Includes neutropenia and neutrophil count decreased

^c^Includes anemia, hemoglobin decreased, red blood cell count decreased and hematocrit decreased

^d^Includes thrombocytopenia and platelet count decreased

^e^Includes leukopenia and white blood cell count decreased

^f^Includes lymphopenia and lymphocyte count decreased.

Additional safety data are summarized in Extended Data Tables 1 and 2. Due to the small number of patients assessed at each DL, a possible dose-dependent correlation between the frequency and severity of adverse events was not evaluated. However, based on the overall safety results and the observation that more patients experienced DLTs at DLs >2.8 mg kg^−1^, the MTD was determined to be 2.8 mg kg^−1^. The median DLT probability estimated using the Bayesian model at the selected MTD of 2.8 mg kg^−1^ was 23.9% (95% quantile 37.8%).

#### Efficacy overview

**Fig. 3:**
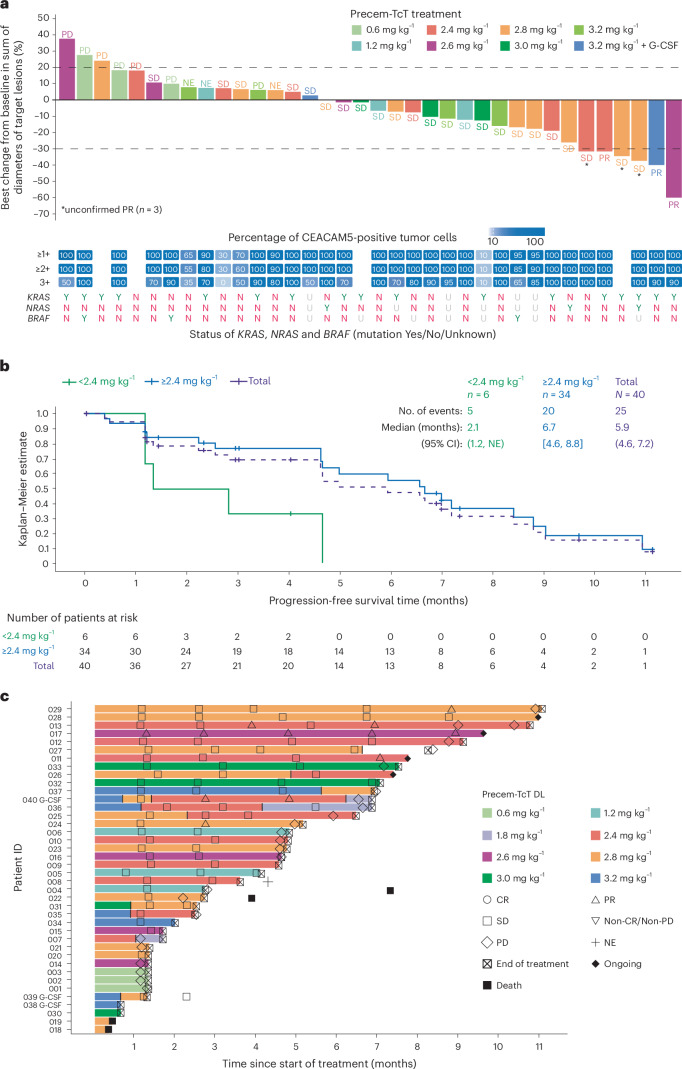
Efficacy overview. **a**, Best overall response (confirmed). The waterfall plot only includes patients who have a post-baseline assessment (*n* = 36). Most of the patients had high CEACAM5 expression, as indicated by the dark blue boxes in the ‘Percentage of CEACAM5-positive tumors’ category. Blank boxes indicate that CEACAM5 expression data were not available. **b**, Progression-free survival. **c**, Treatment duration and response over time. CI, confidence interval; CR, complete response; N, no; NE, not evaluable; PD, progressive disease; PR, partial response; SD, stable disease; U, unknown; Y, yes.

The median treatment duration of Precem-TcT was 19.1 weeks (range: 1.7–48.3). The best overall response was partial response (confirmed) in three of 40 patients (7.5%) (Fig. 3a) at DLs of 2.4 mg kg^−1^, 2.6 mg kg^−1^ and 3.2 mg kg^−1^. The duration of response in these three patients was 3.8 months, 5.1 months and 8.4 months; treatment was ongoing in one patient at data cutoff. The unconfirmed partial response rate was 15.0% (*n* = 6), which included the three patients with confirmed partial responses. The overall DCR (post hoc endpoint) at week 12 was 55.0%, and the mPFS was 5.9 months (95% confidence interval: 4.6–7.2) (Fig. 3b). At DLs ≥2.4 mg kg^−1^ (*n* = 34), which corresponded to the predicted effective dosing range, the confirmed partial response rate was 8.8% (unconfirmed partial response rate: 17.6% (6/34)); the DCR at week 12 was 58.8%; and the mPFS was 6.7 months (95% confidence interval: 4.6–8.8). Fourteen of 34 (41.2%) patients continued treatment for at least 6 months, five of whom were on treatment for more than 9 months. Overall, four (10.0%) patients remained on treatment at the data cutoff (Fig. 3c).

#### Clinical pharmacokinetics

**Extended Data Fig. 3:**
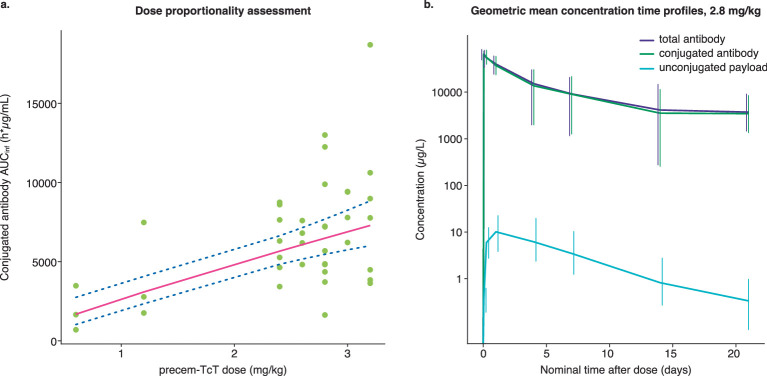
Clinical pharmacokinetics of Precem-TcT. Panel **a**. Solid line represents the predicted value, dotted lines show 95% CI. Panel **b**. Error bars show 95% CIs (assessed as the 2.5^th^ and 97.5^th^ quantiles by nominal time points). AUC, area under curve; CI, confidence interval.

Across the 1.2–3.2 mg kg^−1^ dose range, exposures of the conjugated antibody and exatecan increased approximately proportionally with the administered dose (Extended Data Fig. 3a). Overall, the pharmacokinetic profiles of the total antibody and conjugated antibody largely overlapped, highlighting the stability of the linker–payload in circulation (Extended Data Fig. 3b). The half-life (*t*_1/2_) for the conjugated antibody was 5.7 days, whereas that for exatecan was 4.9 days. Minimal accumulation of exatecan was observed upon repeated dosing, which is consistent with manageable multicycle tolerability. Precem-TcT systemic exposures at DLs ≥2.4 mg kg^−1^ (every 3 weeks) were found to be pharmacologically relevant for antitumor activity based on modeling and simulation. Although detailed analyses of translational and pharmacokinetic/pharmacodynamic (PK/Pd) modeling of Precem-TcT have not been presented, they played a crucial role in the selection of the RDEs.

#### Clinical biomarker overview

Most patients had high CEACAM5 expression levels, consistent with expectations in patients with mCRC^24^. Based on the currently available data from biomarker analyses, Precem-TcT activity does not appear to be impacted by *KRAS*, *NRAS* or *BRAF* mutation status (Fig. 3a). The analysis of immunohistochemistry-assessed CEACAM5 expression levels (histoscore) and baseline serum carcinoembryonic antigen (sCEA) levels indicated no statistically significant relationship (Spearman’s rho = −0.14, *P* = 0.4).

#### ADA against Precem-TcT

All 40 patient samples were assessed for ADAs against Precem-TcT; only one patient in the 0.6 mg kg^−1^ cohort tested positive.

Considering the totality of clinical and preclinical data, along with PK/Pd modeling analyses, 2.4 mg kg^−1^ and 2.8 mg kg^−1^ were selected as the RDEs and are included in the ongoing randomized dose-optimization study.

## Discussion

The anti-CEACAM5 ADC Precem-TcT demonstrated a predictable safety profile and encouraging clinical activity in this dose-escalation part of the first-in-human phase 1 PROCEADE-CRC-01 study. These findings corroborate preclinical data, which showed high antitumor activity of Precem-TcT in vitro and in vivo, along with a toxicity profile in cynomolgus monkeys that was consistent with the known safety profile of exatecan^27–30^.

In this phase 1 dose escalation, the observed adverse event profile of Precem-TcT was overall consistent with the known safety profile of exatecan. The observed adverse events were primarily hematologic toxicities that were mostly resolved with appropriate intervention, allowing patients to continue Precem-TcT therapy, as indicated by the low number of permanent treatment discontinuations. These transient cytopenic events were consistent with the adverse effects to hematolymphoid tissues and transient reductions in hematologic parameters reported in monkeys. As hematopoietic cells lack CEACAM5 expression, the observed cytopenia can be attributed to the known myelosuppressive effects of exatecan, with plasma exposure levels in monkeys^27^ being similar to that of unconjugated exatecan after Precem-TcT infusion in patients.

Other adverse events commonly associated with ADCs include ILD, ocular toxicities and gastrointestinal toxicities. In this study, no cases of ILD or ocular toxicities were reported, and Precem-TcT-related gastrointestinal events were mostly grade 1. ILD/pneumonitis has a reported incidence of 1–15.8% in patients treated with ADCs, with some specific deruxtecan-based ADCs being associated with the development of ILDs up to grade 5 (refs. ^31–33^). Data suggest that ILD is not directly induced by exatecan^28–30^ or the exatecan-derivative payload DXd (the TOP1i part of deruxtecan), nor is it dependent on the antigen target such as human epidermal growth factor receptor 2 (HER2) in case of trastuzumab deruxtecan^33^. As recently postulated, the deruxtecan linker–payload construct includes amino-methylene as a self-immolative spacer, which may mediate ILD by producing toxic by-products directly or via local amine oxidase processing of an intermediate as substrate, resulting in oxidative stress, inflammation and, finally, fibrosis in lung parenchyma when co-released with DXd^27^. Precem-TcT does not contain an amino-methylene spacer between the linker and the payload. According to the hypothesis, this could, at least in part, explain the absence of ILD cases in our phase 1 study and in cynomolgus monkeys. Severe ocular toxicities are routinely observed for certain ADCs, especially those with auristatin (MMAF) and maytansinoid-derivative (DM4) payloads^34^. For example, grade 3 microcystic keratopathy was observed in five of 28 patients (17.8%) in a phase 1 study of tusamitamab ravtansine, an anti-CEACAM5 ADC with a maytansine derivative (DM4) payload, in patients with advanced solid tumors^35^. In our phase 1 study, no ocular toxicities were observed, in accordance with eye investigations in cynomolgus monkeys. Although factors such as vascularity of the eye and rapid cell division within ocular cells make it more susceptible to off-target toxicities, linker instability or premature cleavage in extracellular environments and non-specific uptake via pinocytosis or Fcγ receptor (FcγR)-mediated cellular uptake of the ADC may also contribute to the development of ocular toxicities^34,36–38^. Some of these factors—for example, rapidly proliferating cells in the gastrointestinal tract and premature release of cytotoxic payloads—have also been implicated in gastrointestinal toxicities observed with ADCs, and grade 3/4 gastrointestinal adverse events have been frequently reported in ADCs with TOP1i payloads^37^. For instance, the incidence of diarrhea and other gastrointestinal toxicities has been shown to be associated with the metabolism of SN-38, which is used as a payload in certain ADCs and is the active metabolite of irinotecan (a member of the TOP1i family). SN-38 is inactivated through glucuronidation by UGT1A1 in the liver; however, during enterohepatic cycling, gut microbiota reactivates it via the enzyme β-glucuronidase. This reactivation concentrates the active metabolite in the gut mucosa, leading to cellular damage. In contrast, the primary biotransformation pathway for unconjugated exatecan in Precem-TcT is oxidation, primarily mediated by the CYP3A4 enzyme^28,29^. This enzyme is abundantly present in the intestine, minimizing local toxic effects on enterocytes, as confirmed in monkeys^27^. Unlike irinotecan, no glucuronides were detected with exatecan, consistent with existing literature and supporting the decision to forego UGT1A testing in our clinical trial^29,39^. In general, either the payload causes cytotoxic effects directly on cells that take up the ADC or ADC metabolism results in circulating free payload. Linker–payload stability in circulation was established as a key attribute of Precem-TcT in our clinical pharmacokinetic assessments. We hypothesize that the combination of our CEACAM5-targeted antibody with Fc protein engineering to limit FcγR binding and prevent C1q interaction^40^, with a highly stable, hydrophilic, linker–payload combination (β-glucuronide–exatecan), and the favorable metabolic profile of the exatecan payload, positively contributed to the overall favorable safety profile of Precem-TcT compared to other ADCs. The absence of immune-related adverse events after Precem-TcT infusion in the phase 1 study, likely due to the elimination of Fc effector functions of Precem-TcT, provides additional evidence supporting its favorable safety profile.

The Precem-TcT-related death, which was also attributed by the investigator to the underlying disease burden, underscores the challenge of distinguishing between mortality due to disease progression and treatment-related toxicity in advanced solid tumors, including CRC. In recent phase 3 clinical trials involving heavily pretreated patients with mCRC, most treatment discontinuations^15^ and deaths^16,41^ were attributed to disease progression rather than treatment-related causes. When examining deaths in the context of ADCs, a systematic review and meta-analysis of 169 clinical trials using ADCs revealed that the overall incidence of treatment-related deaths was 1.3% (95% confidence interval: 1.1–1.5%)^42^. Among anti-CEACAM5 ADCs, the phase 1/2 study of labetuzumab govitecan reported no treatment-related deaths in patients with refractory/relapsing mCRC^43^. In the phase 1 dose-escalation study of tusamitamab ravtansine in patients with advanced solid tumors, five of the six reported deaths were attributed to disease progression, with one death occurring 3 months after treatment was stopped^35^.

Tumor response assessment in this phase 1 dose escalation suggests potential clinical benefit with Precem-TcT. At doses ≥2.4 mg kg^−1^—the predicted clinically effective dose—the DCR was 58.8% (confirmed partial response rate, 8.8%) and the mPFS was 6.7 months (95% confidence interval: 4.6–8.8). These early efficacy data are encouraging, especially considering that all study participants had mCRC and had progressed on irinotecan therapy, and most (80%) had received three or more prior lines of treatment. In addition, current monotherapy SoCs for 3L+ mCRC treatment, such as trifluridine/tipiracil, regorafenib and fruquintinib, show mPFS durations of 1.9–3.7 months and overall response rates (ORRs) of only 1–2%^16,17,41,44^. Notably, in the recent phase 3 SUNLIGHT study, which included fewer pretreated patients with mCRC who had received two or fewer prior chemotherapy regimens, the combination of trifluridine/tipiracil with bevacizumab achieved an ORR of 6.1% and an mPFS of 5.6 months^15^. The early efficacy results from our phase 1 dose-escalation study of Precem-TcT in this difficult-to-treat patient population with an urgent unmet need are encouraging and are being further evaluated in the ongoing expansion portion of the study.

Targeting tumors through CEACAM5 has been an attractive approach for ADC development, but there has been a recent setback with the discontinuation of clinical development of the CEACAM5-targeting ADC tusamitamab ravtansine after its failure to improve mPFS compared to docetaxel in a phase 3 study for non-small cell lung cancer^35,45^. Of note, tusamitamab ravtansine was not evaluated beyond phase 1 in mCRC^32,46^, possibly due to the low sensitivity of CRC to microtubule inhibitor-based payloads. This recent failure highlights the importance of selecting the appropriate linker–payload combination for the specific target patient population, in addition to the target antigen. In CRC, CEACAM5 is recognized as a reliable biomarker of tumor cells, with elevated serum CEACAM5 levels associated with increased mortality in patients with CRC^47,48^. In contrast to tusamitamab ravtansine, Precem-TcT uses a cleavable β-glucuronide linker and a TOP1i payload (exatecan), and our preclinical data suggest markedly improved antitumor activity compared to tusa.rav.a (the analog of tusamitamab ravtansine) in CRC PDX models. Together with our encouraging phase 1 data, these results indicate that ADCs targeting CEACAM5 with a potent TOP1i payload—demonstrated to have clinical activity in CRC^32,49^—may be effective in patients with mCRC. Notably, the investigation of CEACAM5 as an ADC target continues across tumor types, as observed with the two recent anti-CEACAM5 ADCs with TOP1i payloads—SGNCEACAM5C/SAR445953 and BG-C477—which are currently being assessed in phase 1 open-label studies in adults with select advanced solid tumors^50,51^. Currently, Precem-TcT is the only anti-CEACAM5 ADC that has reported clinical data in CRC and is continuing to be developed in this indication.

Heterogeneous expression of target proteins on tumor cells may present an additional hurdle to delivering payloads effectively. Although CEACAM5 is overexpressed and highly prevalent in CRC, CEACAM5-positive tumor cells are interspersed with tumor cells expressing low or no CEACAM5 (refs. ^35,52^), posing a potential challenge for CEACAM5-targeting ADCs. However, Precem-TcT demonstrated a potent bystander effect in our co-culture experiments—an effect not observed with tusa.rav.a—suggesting that Precem-TcT can effectively target tumors with heterogeneous CEACAM5 expression.

The development of intrinsic or acquired drug resistance presents yet another key challenge. In CRC, drug efflux pumps are known to be associated with limited drug activity^53^. The in vitro potency of Precem-TcT and its exatecan payload was not affected by the inhibition of efflux pumps, suggesting that these resistance mechanisms do not impact the efficacy of Precem-TcT. In addition to efflux pumps, many known resistance mechanisms related to irinotecan (such as uptake or prodrug metabolism) may not be relevant for exatecan payload ADCs^54^. In a PDX mouse model derived from a patient with CRC who had progressed after receiving irinotecan-containing SoC, Precem-TcT demonstrated notable antitumor activity that was not observed with irinotecan. Taken together, these findings indicate that Precem-TcT could be considered after irinotecan SoC treatment and may improve treatment outcomes in patients with irinotecan resistance.

The non-randomized design of this phase 1 study, combined with the small sample size across the multiple DLs tested, limits the study’s generalizability. Given that all patients had high levels of CEACAM5 expression and that the sample size was limited, no inferences could be made regarding a potential correlation between CEACAM5 expression and efficacy.

In conclusion, the anti-CEACAM5 ADC Precem-TcT demonstrated a predictable safety profile and promising early efficacy in 40 heavily pretreated patients with mCRC. Given the strong preclinical rationale and encouraging antitumor activity at tolerable doses (2.4 mg kg^−1^ and 2.8 mg kg^−1^), the ongoing PROCEADE-CRC-01 study is exploring alternative administration schedules of Precem-TcT (DLs of 2.4 mg kg^−1^ and 2.8 mg kg^−1^) and combinations with bevacizumab±capecitabine or bevacizumab+5-fluorouracil in this patient population with a high unmet need. The outcomes of these exploratory cohorts will inform the next steps in the evaluation of Precem-TcT in this setting.

## Methods

### Preclinical evaluation of Precem-TcT

The effects of Precem-TcT on cancer cell viability, including its potential to kill CEACAM5-negative cells in the vicinity of CEACAM5-positive cells (bystander effect), its interactions with transporters, its efficacy in CRC models and its preclinical safety profile, were evaluated. To understand how the linker–payload combination in Precem-TcT (β-glucuronide and exatecan) compares to traditional ADC combinations, we used an analog of tusamitamab ravtansine as a comparator in our preclinical studies. Tusamitamab ravtansine is an anti-CEACAM5 ADC with a microtubulin inhibitor payload (DM4, belonging to the class of maytansinoids) and a cleavable disulfide linker. In our experiments, its analog, referred to as tusa.rav.a., is an anti-CEACAM5 huMab3-2 antibody conjugated with the DM4 payload.

### Bystander effect assay

ADCs were tested at a concentration of 1 nM on co-cultured SKCO-1 and MDA-MB-231 cells. In total, 1,000 MDA-MB-231 cells were used in co-culture experiments with 750 or 3,000 SKCO-1 cells per well. As a control, wells with only 1,000 MDA-MB-231 cells were tested in parallel. Cells were seeded in a total volume of 90 µl in 96-well plates (Corning, no. 3603) the day before treatment. Test compound was formulated at tenfold of the final concentration of 1 nM in cell culture medium, and 10 µl was added to duplicate wells. Plates were cultured at 37 °C in a CO_2_ incubator for 6 days.

Prior to immunofluorescence staining, medium was removed, and cells were treated with ice-cold 100% methanol at 20 °C for 30 minutes. After methanol removal and one PBS wash step, cells were treated with 2.5% paraformaldehyde with 0.2% Triton X-100 in PBS for 15 minutes at room temperature. After solution removal and one PBS wash step, cells were treated with 1% BSA/0.1% Tween/0.1% sodium azide in PBS for at least 1 hour at room temperature. Antigen-positive and antigen-negative cells were discriminated by immunofluorescence staining with 10 µg ml^−1^ human anti-CEACAM5 primary antibody (antibody moiety of Precem-TcT) and a 1:200 dilution of donkey anti-human IgG fluorescently (phycoerythrin) labeled secondary antibody (Jackson ImmunoResearch, no. 709-116-149). Cells were identified by nuclei staining using 1 µg ml^−1^ Hoechst 33342 dye. Staining was carried out in 1% BSA/0.1% sodium azide PBS solutions for 30 minutes at room temperature. Secondary antibody staining was combined with Hoechst dye staining. Between and after staining steps, cells were washed three times with PBS.

Plates were imaged with a CQ1 confocal quantitative image cytometer (Yokogawa Electric Corporation). Data analysis was performed using CQ1 software, FlowJo, Microsoft Excel and GraphPad Prism.

The number of cells after treatment was determined by quantifying the number of stained nuclei using Hoechst 33342 staining and the CQ1 confocal quantitative image cytometer. Based on staining or absence of staining with fluorescently labeled anti-CEACAM5 antibody around the nucleus, antigen-positive and antigen-negative cells were discriminated and quantified.

### In vitro viability assays

The effect of Precem-TcT, a tusamitamab ravtansine analog (tusa.rav.a.; anti-CEACAM5 huMab3-2 with DM4 payload), or free payloads (exatecan or DM4) on cell viability was investigated using SKCO-1, MKN-45 and MDA-MB-231 cancer cell lines. The cells were plated at a defined density 1 day before treatment. After overnight incubation under 5% or 10% CO_2_, treatment was performed with a serial dilution of Precem-TcT, tusa.rav.a. or exatecan payload for 144 hours in triplicate. In control wells, RPMI with 2 mM stable glutamine, 10% FBS and 1 mM sodium pyruvate (control for ADC treatment) or RPMI with 2 mM stable glutamine, 10% FBS and 1 mM sodium pyruvate containing the respective amount of DMSO (control for payload treatment) was added to the cells. Background wells (no-cell control) contained only the medium. To assess cell viability, the CellTiter-Glo^®^ Luminescent Cell Viability assay was performed according to the manufacturer’s instructions.

Raw data of relative luminescence units were processed in Microsoft Excel by subtracting the mean of the background values (no-cell control, only medium) and by calculating the %viability (untreated control cells were set as 100%) or %effect (%viability − 100%). Dose–response curves and IC_50_ values were obtained by data transformation and subsequent data fitting using nonlinear regression analysis function (log(inhibitor) versus response–variable slope (four parameters)) in GraphPad Prism. Alternatively, data were processed and evaluated equivalently using Genedata Screener software. Data were expressed as %effect versus log_10_(dose of compound concentration (M)) with error bars indicating the s.d. of the technical triplicates.

### MDR1 inhibition potency shift assay

To investigate whether the effect of ADCs or free payloads was affected by MDR1 inhibition, a potency shift assay was performed, adapted from a viability assay. ADC or payload potency against LS513 and LoVo cancer cell lines was determined in the absence or presence of the MDR1 inhibitor zosuquidar. After overnight incubation in a 5% CO_2_ incubator, treatment was performed with a serial dilution of exatecan, DM4 or MMAE for 144 hours in duplicate. Each compound was tested with or without the addition of a constant concentration of zosuquidar (100 nM). In control wells, RPMI with 2 mM glutamine, 10% FBS and 1 mM sodium pyruvate containing the respective amount of DMSO was added to the cells. Background wells (no-cell control) contained only the medium. To assess cell viability, the CellTiter-Glo Luminescent Cell Viability assay was performed according to the manufacturer’s instructions.

### Efficacy assessment of Precem-TcT in CRC PDX mouse models

The efficacy of ADC Precem-TcT was evaluated in different CRC PDX models with high CEACAM5 expression according to immunohistochemical analysis and mRNA levels. CEACAM5 expression was evaluated via immunohistochemical analysis using a proprietary anti-CEACAM5 antibody. The antitumor efficacies of Precem-TcT and tusa.rav.a. were compared in the human CRC PDX models COPF230 and REPF210. The experiments were performed at LIDE Biotech according to the internal quality management system defined in the LDIACUC001 Quality Manual for non-GxP-regulated activities. The protocol was approved by the LIDE Institutional Animal Care and Use Committee (IACUC). Immunodeficient female mice (5–6 weeks old; Crl:NUFoxn1nu, Beijing Vital River Laboratories) were transplanted subcutaneously in the right flank with COPF230 or REPF210 tumor fragments. When the tumor reached the desired mean volume (150–250 mm^3^), the mice were randomized, and six mice per group (REPF210) and five mice per group (COPF230) were treated once (day 0) intravenously with the vehicle (normal saline) or a single dose of 6 mg kg^−1^ Precem-TcT or tusa.rav.a.

The antitumor efficacies of Precem-TcT and irinotecan were compared in the human CRC PDX model CXF4102, derived from a patient with CRC treated with irinotecan-based SoC (FOLFIRI/Avastin; that is, pretreatment with irinotecan). The experiment was performed at Charles River Discovery Research Services Germany GmbH, and the protocol was approved by the local animal welfare authorities (Regierungspräsidium Freiburg, Baden-Württemberg; general license number, G-20/163; study-specific authorization number, G-20/163.137). Six-to-eight-week-old immunodeficient female mice (NMRI nude, Charles River Laboratories) were transplanted subcutaneously in the right flank with CXF4102 tumor fragments. When the tumor reached the desired mean volume (150–250 mm^3^), the mice were randomized, and six mice per group were treated intravenously with the vehicle (normal saline, every 2 weeks × 3) or 4 mg kg^−1^ Precem-TcT (every 2 weeks × 3) or 10 mg kg^−1^ irinotecan (every 4 days × 8) (Accord, PZN 12422479, batch M2005409).

Tumor length (L) and width (W) were measured with calipers, and tumor volumes were calculated using L × W^2^ / 2. Graphs of tumor suppression were plotted using GraphPad Prism. Percentage of tumor volume (%TV) was calculated using the following formula: %TV = ((meanTV doa treatment − meanTV start treatment) / mean TV start treatment)) × 100%, where TV is ‘tumor volume’ and doa is ‘day of analysis’.

Tumor progression, tumor stasis and tumor regression were defined as follows: tumor progression: mean %TV > 73%; tumor stasis: %TV between −66% and 73%; and tumor regression: mean %TV ≤ −66% at day of analysis compared to the TV at start of treatment.

### Toxicology assessment in cynomolgus monkeys

Precem-TcT was administered every 3 weeks by 30-minute (±5-minute) intravenous infusion to four groups of male and female monkeys (vehicle and three test item groups) in each study in the dose range of 3–30 mg kg^−1^ for three consecutive times (days 1, 22 and 43). Toxicity indices consisted of clinical observations, body weight, food consumption, ophthalmology, clinical pathology, immunophenotyping, gross pathology, organ weights, histopathology and toxicokinetic assessment as well as integrated safety pharmacology measurements (cardiovascular, respiratory and neurological functions)^27^. Necropsy for histopathology was executed at day 50. All procedures and aforementioned experimental investigations with purpose-bred, naive Vietnamese cynomolgus monkeys, including purchasing, source, housing conditions, body weight and age and compliance with the animal welfare act(s) following the recommendations of the Association for Assessment and Accreditation of Laboratory Animal Care (AAALAC) and national animal health regulations and animal ethics approval of study protocols by the IACUC of the healthcare business of Merck KGaA, Darmstadt, Germany and Istituto di Ricerche Biomediche ‘Antoine Marxer’–RBM S.p.A., were described previously^27^.

#### Clinical evaluation of Precem-TcT

##### Phase 1 study oversight

The study is being conducted in compliance with the International Council for Harmonization Good Clinical Practice guidelines and the principles of the Declaration of Helsinki. The study protocol, amendments and informed consent forms were reviewed and approved by the institutional review board/independent ethics committee at each of the five study sites before study initiation (NEXT US (approved 14 June 2022); MD Anderson Cancer Center US (approved 30 June 2022); Vall d’Hebron Institute of Oncology Spain (approved 29 August 2022); NEXT Spain (approved 20 August 2022); and National Cancer Center Japan (approved 26 August 2022)). Written informed consent was obtained from all patients before screening.

The study is overseen by a safety monitoring committee (SMC), which reviewed safety, pharmacokinetic and preliminary clinical activity data after the completion of each dosing cohort. Formal SMC approval was required for each dose escalation.

##### Patient population

A detailed overview of the inclusion/exclusion criteria for the dose-escalation part of the trial is presented below:

##### Inclusion criteria

- ≥18 years of age at the time of signing the informed consent. In Taiwan and South Korea, participants who are ≥20 years of age at the time of signing the informed consent.
- Patients with documented histopathological diagnosis of locally advanced or metastatic CRC who were intolerant/refractory to or progressed after standard systemic therapies for the advanced/metastatic stage that included, and are restricted to, fluoropyrimidine, irinotecan, a platinum agent (for example, oxaliplatin), an anti-EGFR agent (if clinically indicated—that is, *RAS/BRAF* wild-type), an anti-VEGF agent and/or either trifluridine/tipiracil or regorafenib, if locally indicated and available to the patient. Participants with a known MSI-H status must have received treatment with an immune checkpoint inhibitor (if locally indicated and available) unless contraindicated.
- Sex and contraception/barrier requirements: all sexes allowed.
  - The investigator confirmed that each participant agrees to use appropriate contraception and barriers, if applicable. Details of the contraception, barrier and pregnancy testing requirements are included in the protocol.
- Informed consent: capable of giving signed informed consent, which includes compliance with the requirements and restrictions listed in the informed consent form and the protocol.
- ECOG PS ≤1
- Hematologic function: is adequate, as indicated by:
  - Platelet count ≥100,000 per mm^3^ (no transfusion in the past 2 weeks before first dose)
  - Hemoglobin ≥9.0 g dl^−1^ (no transfusion in the past 2 weeks before first dose)
  - Absolute neutrophil count ≥1,500 per μl (no hematopoietic growth factors G-CSF in the past 2 weeks before first dose)
  - International normalized ratio ≤1.5× the upper limit of normal (ULN)
- Hepatic function: is adequate, as defined by a total bilirubin level ≤1.5× ULN, an aspartate aminotransferase (AST) level ≤2.5× ULN and an alanine transaminase (ALT) level ≤2.5× ULN:
  - For documented Gilbert’s syndrome, a total bilirubin <3× ULN is accepted.
  - For participants with liver metastases, AST and ALT <5× ULN is accepted.
- Renal function: is adequate, as defined by serum creatinine ≤1.5× ULN. If serum creatinine is >1.5× ULN, CrCl needs to be ≥30 ml min^−1^ by calculation using the Cockcroft–Gault formula: CrCl (ml min^−1^) = (((140–age (years)) × weight (kg)) / (72 × serum creatinine (mg dl^−1^))) × 0.85 (if female).
- Prior therapy: participants who received prior chemotherapy, radiotherapy (except limited local palliative radiotherapy), biological therapy (for example, antibodies) or investigational drugs must have a wash-out period of 21 days or 5× half-life, whichever is shorter, before receiving the first dose of Precem-TcT.
- sCEA concentration level ≤10 μg ml^−1^
- Archival FFPE tumor tissue is required. If archived tumor material is not available, fresh biopsy is required.

##### Exclusion criteria

- Medical conditions:
  - If adverse events related to previous therapies have not recovered to grade ≤1 by National Cancer Insitute Common Terminology Criteria for Adverse Events version 5.0 (except for lymphopenia, grade 2 peripheral neuropathy, grade 2 alopecia, grade 2 lab abnormalities that are clinically not relevant and grade 2 adverse events from prior immune checkpoint inhibitor therapy that are not relevant as an exclusion criterion per investigator assessment (for example, stable, substituted hypothyroidism)).
  - Participant has a history of malignancy within 3 years before the date of enrollment (exceptions are squamous and basal cell carcinomas of the skin and carcinoma in situ of the cervix, benign prostate neoplasm/hypertropia or malignancy that, in the opinion of the investigator, with concurrence of the Sponsor’s Medical Monitor, is considered cured with minimal risk of recurrence within 3 years).
  - Participants with known brain metastases, except those meeting the following criteria: (1) brain metastases that have been treated locally and are clinically stable for at least 4 weeks prior to the start of treatment; (2) no ongoing neurological symptoms that are related to the brain localization of the disease (sequelae that are a consequence of the treatment of the brain metastases are acceptable).
  - Participants with diarrhea (liquid stool) or ileus grade >1.
  - Participants with active chronic inflammatory bowel disease (for example, ulcerative colitis, Crohn’s disease, intestinal perforation) and/or bowel obstruction.
  - Unstable angina, myocardial infarction, congestive heart failure (New York Heart Association ≥ II) or a coronary revascularization procedure within 180 days of study entry. Calculated QTc average (using the Fridericia correction calculation) of >470 ms.
  - Cerebrovascular accident/stroke (<6 months prior to enrollment).
  - Active or prior ILD/pneumonitis. History of idiopathic pulmonary fibrosis, obliterative bronchiolitis or idiopathic pneumonitis (history of prior resolved radiation pneumonitis allowed).
  - Active symptomatic fungal, bacterial and/or viral infection. Individuals with known or positive testing for HIV or actively infected viral hepatitis B or hepatitis C based on local standards of detection are excluded. Participants with hepatitis C who have been treated with curative therapy are not considered actively infected. Participants with a history of hepatitis C infection will be eligible for enrollment only if the viral load according to the local standards of detection is documented to be below the level of detection in the absence of antiviral therapy during the previous 12 weeks (for example, sustained viral response according to the local product label but no less than 12 weeks, whichever is longer). Opportunistic infections and active COVID-19 infection. Testing for COVID-19 according to local medical practice. In Japan, patients are excluded if hepatitis B surface antigen positive (HBsAg+) or hepatitis B virus DNA (HBV DNA) ≥ 20 IU ml^−1^ (if HBcAb+ and/or HBeAb+).
  - Uncontrolled concurrent illness (for example, serious uncontrolled diabetes (blood glucose >250 mg dl^−1^), symptomatic congestive heart failure, unstable angina pectoris, cardiac arrhythmia or psychiatric illness/social situations that would limit compliance with the study requirements).
  - Estimated life expectancy of <4 months.
  - Steroid therapy for antineoplastic intent taken <7 days prior to the first dose of study intervention.
  - Prior therapy targeting CEACAM5 (for example, anti-CEACAM5 ADC) or an ADC with a TOP1i payload (for example, trastuzumab deruxtecan).
  - Participants currently receiving (or unable to stop using prior to the first dose of study intervention) prohibited medication, as listed in the protocol.
  - Received growth factors (including erythropoietin, darbepoetin, G-CSF, granulocyte-macrophage colony-stimulating factor (GM-CSF)) and platelet stimulators (for example, eltrombopag, romiplostim or interleukin-11) or transfusions within 2 weeks prior to the first day of study intervention.
  - Major surgery within 4 weeks prior to start of study interventional drug.
  - History of severe hypersensitivity reactions to prior therapies with biologicals or excipient of Precem-TcT.

##### Study design and treatment

The treatment period comprised consecutive 21-day cycles of Precem-TcT treatment; the drug was administered intravenously on the first day of each cycle (once every 3 weeks); and treatment was continued until disease progression, unacceptable toxicity or withdrawal of patient consent. The observation period for DLTs was 21 days, starting on the day of the first Precem-TcT administration. The safety follow-up period was 30 ± 3 days after the last treatment administration.

Dose calculation was performed based on the patient’s weight, rounded to the nearest kilogram (that is, the assigned DL in mg kg^−1^ × body weight in kilograms). After a protocol amendment, a dosing cap based on body mass index (BMI) was implemented midway through the dose-escalation part of the study. For patients with a BMI higher than 30 kg m^−^^2^, the investigator used a weight that, based on the patient’s height, corresponded to a maximum BMI of 30 kg m^−^^2^. Dose capping is a recognized strategy for controlling inter-individual pharmacokinetic variability and minimizing the risk of adverse events in heavier patients^55^, with precedent in the development of several ADCs. In our study, BMI was chosen as the basis for the cap to avoid the use of an arbitrary weight threshold for obesity, as a BMI threshold of 30 kg m^−^^2^ aligns with its definition and provides a more consistent approach across diverse populations.

Primary prophylaxis with G-CSF was not allowed and was considered a DLT if used within the DLT period (Part 1A). Therapeutic use was permitted at the physician’s discretion in cases of neutropenia and/or febrile neutropenia events or as secondary prophylaxis if these events had occurred in previous cycles. Part 1B (with primary G-CSF prophylaxis) could mitigate the risk of neutropenia, potentially allowing further dose escalation.

This study employed a Bayesian dose-escalation design. The first dose-escalation cohort, consisting of three patients, received Precem-TcT at the starting dose of 0.6 mg kg^−1^, and subsequent dose escalations proceeded according to the recommendations of the SMC, which were based on available safety and preliminary pharmacokinetic data and supported by a Bayesian two-parameter logistic regression model^56^.

##### Study objectives and endpoints

The primary objectives of the study were to determine (1) the dose–toxicity relationship and the MTD (if reached) of Precem-TcT based on the occurrence of DLTs and adverse events and (2) the RDE(s) of the drug, based on its safety, pharmacokinetic profile and preliminary clinical activity.

Secondary objectives included characterization of the pharmacokinetic profile of Precem-TcT (conjugated antibody, total antibody and unconjugated exatecan payload) and evaluation of its clinical activity indicators, specifically the objective response (with ORR defined as the proportion of patients achieving a confirmed best overall response of complete response or partial response), duration of response and mPFS, all assessed by the investigator using Response Evaluation Criteria in Solid Tumors version 1.1. The response criteria for evaluation of target lesions were as follows:

Complete response: Disappearance of all target lesions. Any pathological lymph nodes (whether target or non-target) must have reduction in short axis to <10 mm.

Partial response: At least a 30% decrease in the sum of diameters of target lesions, taking as reference the baseline sum diameters.

Progressive disease: At least a 20% increase in the sum of diameters of target lesions, taking as reference the smallest sum on trial (this includes the baseline sum if that is the smallest on trial). In addition to the relative increase of 20%, the sum must also demonstrate an absolute increase of at least 5 mm. (Note: the appearance of one or more new lesions is also considered progression).

Stable disease: Neither sufficient shrinkage to qualify for partial response nor sufficient increase to qualify for progressive disease, taking as reference the smallest sum diameters while on trial. Only stable diseases ≥6 weeks after start date are considered a stable disease.

Additional secondary endpoints included the assessment of ADA against Precem-TcT and triplicate digital electrocardiogram measures (change from the baseline QTc over predefined timepoints (not evaluated for the current analysis)). In addition, the DCR (post hoc endpoint) at 12 weeks was assessed, defined as the proportion of patients achieving complete response, partial response, stable disease or non-complete response/non-progressive disease at the week 12 visit (or later) prior to documented progressive disease.

##### Pharmacokinetic analyses

The pharmacokinetics of Precem-TcT was characterized during dose escalation using an intensive pharmacokinetic sample collection schedule. Blood samples were collected on day 1 (pre-dose, end of infusion (EOI) and 6 hours), day 2 (24 hours), day 5 (96 hours), day 8 (168 hours) and day 15 (336 hours) of cycle 1; on day 1 (pre-dose) of cycle 2; on day 1 (pre-dose, EOI and 6 hours) and day 8 (168 hours) of cycle 3; and at pre-dose every two cycles from cycle 4 until the end of treatment. A non-compartmental computation of pharmacokinetic parameters from plasma concentration–time data was performed using Phoenix WinNonlin version 6.4 or higher. Graphical explorations were performed using R (version 4.2.0 or higher, R Project for Statistical Computing).

Based on the collected data, population pharmacokinetic models for the conjugated antibody and unconjugated payload were established.

##### Bioanalytical assays

Serum or plasma samples at multiple prespecified timepoints were quantified for three analytes: total antibody, conjugated antibody and free payload. Total antibody was analyzed using an ELISA-based immunoassay, and conjugated antibody was analyzed with a MesoScale Discovery-Electrochemiluminescence (MSD-ECL) immunoassay. Both methods had a lower limit of quantification (LLOQ) of 100 ng ml^−1^. Free payload was measured by using a validated liquid chromatography with tandem mass spectrometry (LC–MS/MS) assay with an LLOQ of 10 pg ml^−1^. ADAs were analyzed using an ECL bridging format assay.

##### Biomarker analysis

As part of the integrated analysis plan, the tissue expression of CEACAM5 was assessed using an immunohistochemical assay with a proprietary anti-CEACAM5 antibody clone (sCEA–ELECSYS CEA (Roche Cobas system)). The staining intensity of CEACAM5 (combined membrane and cytoplasm score) was semi-quantitatively evaluated in 100 or more viable tumor cells using a standard 0 to 3+ scale. In addition to CEACAM5 immunohistochemical assay of archival tissue, biomarker analyses included the investigation of patient records for *KRAS*, *NRAS* and *BRAF* mutations. CEACAM5 levels in the blood were also assessed (sCEA).

### Statistical analysis

#### Preclinical pharmacology

All in vitro preclinical data were derived from at least two independent experiments. Means and s.d./s.e.m. values are reported. No statistical comparisons were made among the treatment groups.

#### Clinical

All study parameters were summarized descriptively according to each DL and for the overall patient population. Median and 95% quantiles of posterior DLT probabilities for each DL were estimated using the Bayesian two-parameter logistic regression model. The target DLT probability for the MTD suggested by the Bayesian model was set at 30%. A minimum of six evaluable patients needed to be treated at the MTD/RDE, with at least four of these patients required to receive at least 80% of the assigned dose.

Data collection was performed using INFORM (version 7.0.0.1.41, 64-bit). Data analysis was conducted with R (version 4.2.1).

### Major protocol amendments

The maximum absolute dose limit (dose cap) in obese participants with BMI > 30 kg m^−^^2^ was added, and a dosing guidance for patients whose BMI was higher than 30 kg m^−^^2^ was included (described in the ‘Study design and treatment’ subsection). BMI was also added to the vital sign measurements.

Moreover, to clarify the composition of each cohort with respect to capped doses as well as how data from patients whose absolute dose was capped will be analyzed, it was specified that two or more patients in each cohort should have received 80% or more of the actual non-capped dose. In addition, the sensitivity analysis (Bayesian logistic regression model (BLRM)) with actual received dose level (based on absolute dose) was added. For patients whose absolute dose was capped due to their BMI, the SMC was to receive results from the same Bayesian model, where capped patients were considered in the dose level (in mg kg^−1^) that matched their actual received (capped) dose (in milligrams).

To permit the possibility of deviating from the general dose modification guidance in justified cases, if clinically indicated and after discussion with the Sponsor, the protocol was amended to add that physicians may diverge from these recommendations if clinically indicated. To clarify the sourcing of G-CSF, it was added that G-CSF will be provided by the Sponsor.

Additional amendments included clarification regarding the timing of the tumor assessments, pharmacokinetic parameters, serum biomarkers and ADA sampling.

### Reporting summary

Further information on research design is available in the Nature Portfolio Reporting Summary linked to this article.

## Online content

Any methods, additional references, Nature Portfolio reporting summaries, source data, extended data, supplementary information, acknowledgements, peer review information; details of author contributions and competing interests; and statements of data and code availability are available at 10.1038/s41591-025-03843-z.

## Supplementary information

Supplementary Table 1 and Single-letter amino acid sequence of Precem-TcT, along with redacted protocol.

Reporting Summary

## Source data

Source Data Fig. 1 Statistical source data for Fig. 1 and Extended Data Fig. 1.

## Figures and Tables

**Extended Data Table 1:**
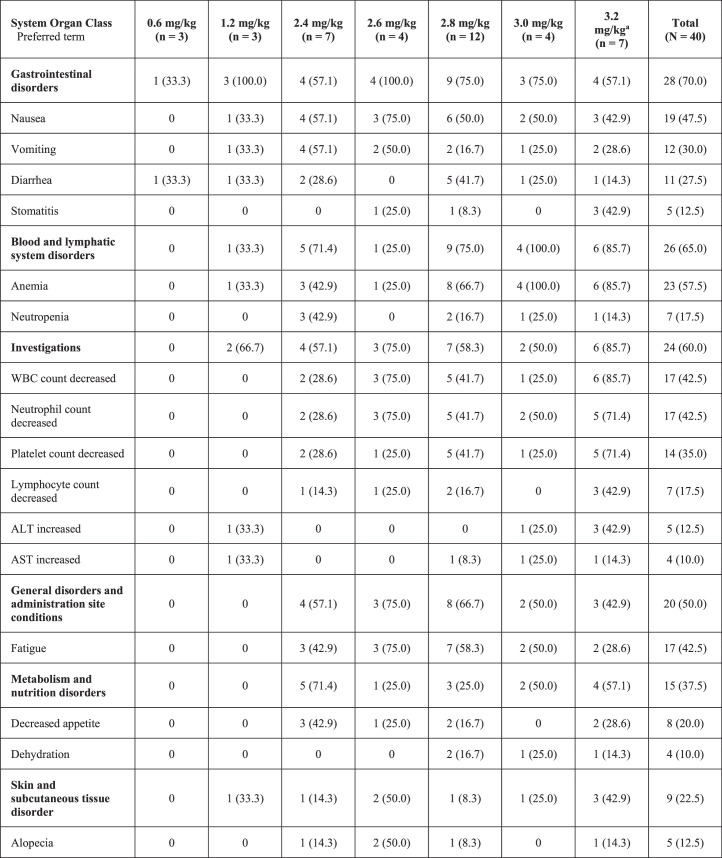
Related TEAEs (reported in ≥10% of patients, overall) by Primary System Organ Class and Preferred Term (Safety Analysis Set)

^a^Including three patients with primary G-CSF prophylaxis

WBC, white blood cell.

**Extended Data Table 2:**
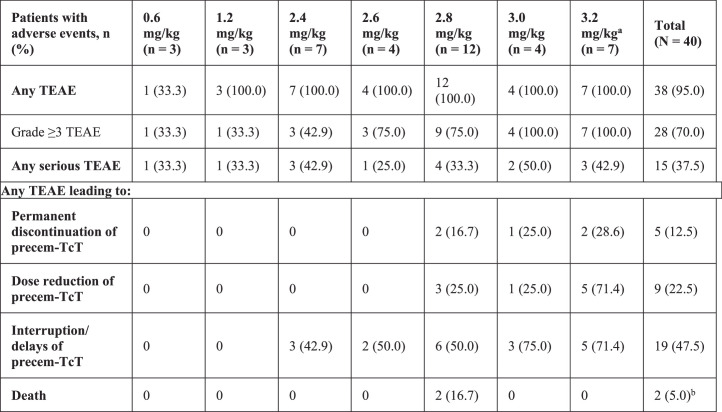
Safety overview

^a^Including three patients with primary G-CSF prophylaxis

^b^Death due to sepsis (*n* = 1) and gastrointestinal hemorrhage (*n* = 1; not related to Precem-TcT; deemed to be related to myelosuppression)

## Data Availability

Any requests for data by qualified scientific and medical researchers for legitimate research purposes will be subject to the data-sharing policy of the healthcare business of Merck KGaA, Darmstadt, Germany. After completion of the study and finalization of the clinical study reports, deidentified patient data and the statistical analysis plan can be provided upon request from qualified researchers. Access to the data is controlled for privacy, ethical and compliance reasons. All requests should be submitted in writing to the data sharing portal for the healthcare business of Merck KGaA, Darmstadt, Germany (https://www.emdgroup.com/en/research/our-approach-to-research-and-development/healthcare/clinical-trials/commitment-responsible-data-sharing.html). When the healthcare business of Merck KGaA, Darmstadt, Germany has a co-research, co-development, co-marketing or co-promotion agreement, or when the product has been out-licensed, the responsibility for disclosure might be dependent on the agreement between parties. Under these circumstances, the healthcare business of Merck KGaA, Darmstadt, Germany will endeavor to gain agreement to share data in response to requests. The antibody sequence of Precem-TcT was previously published^57^ and is included in the Supplementary Information file. A patent application describing Precem-TcT has been filed (published international patent application WO 2022/048883 A1). Source data are provided with this paper.
