# Supplementary material for: Precemtabart tocentecan, an anti-CEACAM5 antibody–drug conjugate, in metastatic colorectal cancer: a phase 1 trial
Source: Nat Med. 2025 Jul 30;31(10):3504–13. doi: 10.1038/s41591-025-03843-z (PMC12532702; doi:10.1038/s41591-025-03843-z)
Supplement: Supplementary file 1 — Supplementary Table 1 and Single-letter amino acid sequence of Precem-TcT, along with redacted protocol. [file 41591_2025_3843_MOESM1_ESM.pdf]

nature medicine

Supplementary information

https://doi.org/10.1038/s41591-025-03843-z

Precemtabart tocentecan, an anti-CEACAM5 antibody–drug conjugate, in metastatic colorectal cancer: a phase 1 trial

In the format provided by the authors and unedited

Supplementary Table 1: TEAEs by Worst Grade, SOC and PT - SAF Analysis Set

**Primary System Organ Class**<br>Preferred Term	**0.6 mg/kg**<br>**n=3 (100%)**					**1.2 mg/kg**<br>**n=3 (100%)**				
	**Any Grade**<br>**n (%)**	**Grade ≥2**<br>**n (%)**	**Grade ≥3**<br>**n (%)**	**Grade ≥4**<br>**n (%)**	**Grade =5**<br>**n (%)**	**Any Grade**<br>**n (%)**	**Grade ≥2**<br>**n (%)**	**Grade ≥3**<br>**n (%)**	**Grade ≥4**<br>**n (%)**	**Grade =5**<br>**n (%)**
**Subjects with at least one Event**	1 (33.3)	1 (33.3)	1 (33.3)	0 (0.0)	0 (0.0)	3 (100.0)	2 (66.7)	1 (33.3)	0 (0.0)	0 (0.0)
**Gastrointestinal disorders**	1 (33.3)	1 (33.3)	0 (0.0)	0 (0.0)	0 (0.0)	3 (100.0)	0 (0.0)	0 (0.0)	0 (0.0)	0 (0.0)
Nausea	0 (0.0)	0 (0.0)	0 (0.0)	0 (0.0)	0 (0.0)	1 (33.3)	0 (0.0)	0 (0.0)	0 (0.0)	0 (0.0)
Vomiting	0 (0.0)	0 (0.0)	0 (0.0)	0 (0.0)	0 (0.0)	1 (33.3)	0 (0.0)	0 (0.0)	0 (0.0)	0 (0.0)
Diarrhea	1 (33.3)	0 (0.0)	0 (0.0)	0 (0.0)	0 (0.0)	1 (33.3)	0 (0.0)	0 (0.0)	0 (0.0)	0 (0.0)
Constipation	1 (33.3)	1 (33.3)	0 (0.0)	0 (0.0)	0 (0.0)	0 (0.0)	0 (0.0)	0 (0.0)	0 (0.0)	0 (0.0)
Stomatitis	0 (0.0)	0 (0.0)	0 (0.0)	0 (0.0)	0 (0.0)	0 (0.0)	0 (0.0)	0 (0.0)	0 (0.0)	0 (0.0)
Abdominal pain	0 (0.0)	0 (0.0)	0 (0.0)	0 (0.0)	0 (0.0)	0 (0.0)	0 (0.0)	0 (0.0)	0 (0.0)	0 (0.0)
Abdominal pain upper	0 (0.0)	0 (0.0)	0 (0.0)	0 (0.0)	0 (0.0)	0 (0.0)	0 (0.0)	0 (0.0)	0 (0.0)	0 (0.0)
Rectal hemorrhage	0 (0.0)	0 (0.0)	0 (0.0)	0 (0.0)	0 (0.0)	0 (0.0)	0 (0.0)	0 (0.0)	0 (0.0)	0 (0.0)
Abdominal distension	0 (0.0)	0 (0.0)	0 (0.0)	0 (0.0)	0 (0.0)	0 (0.0)	0 (0.0)	0 (0.0)	0 (0.0)	0 (0.0)
Anal hemorrhage	0 (0.0)	0 (0.0)	0 (0.0)	0 (0.0)	0 (0.0)	0 (0.0)	0 (0.0)	0 (0.0)	0 (0.0)	0 (0.0)
Ascites	0 (0.0)	0 (0.0)	0 (0.0)	0 (0.0)	0 (0.0)	0 (0.0)	0 (0.0)	0 (0.0)	0 (0.0)	0 (0.0)
Flatulence	0 (0.0)	0 (0.0)	0 (0.0)	0 (0.0)	0 (0.0)	0 (0.0)	0 (0.0)	0 (0.0)	0 (0.0)	0 (0.0)
Gastrointestinal hemorrhage	0 (0.0)	0 (0.0)	0 (0.0)	0 (0.0)	0 (0.0)	0 (0.0)	0 (0.0)	0 (0.0)	0 (0.0)	0 (0.0)
Hematochezia	0 (0.0)	0 (0.0)	0 (0.0)	0 (0.0)	0 (0.0)	0 (0.0)	0 (0.0)	0 (0.0)	0 (0.0)	0 (0.0)
Hemorrhoidal hemorrhage	0 (0.0)	0 (0.0)	0 (0.0)	0 (0.0)	0 (0.0)	0 (0.0)	0 (0.0)	0 (0.0)	0 (0.0)	0 (0.0)
Ileus	0 (0.0)	0 (0.0)	0 (0.0)	0 (0.0)	0 (0.0)	0 (0.0)	0 (0.0)	0 (0.0)	0 (0.0)	0 (0.0)
Intestinal obstruction	0 (0.0)	0 (0.0)	0 (0.0)	0 (0.0)	0 (0.0)	0 (0.0)	0 (0.0)	0 (0.0)	0 (0.0)	0 (0.0)
Upper gastrointestinal hemorrhage	0 (0.0)	0 (0.0)	0 (0.0)	0 (0.0)	0 (0.0)	0 (0.0)	0 (0.0)	0 (0.0)	0 (0.0)	0 (0.0)
**Blood and lymphatic system disorders**	0 (0.0)	0 (0.0)	0 (0.0)	0 (0.0)	0 (0.0)	1 (33.3)	0 (0.0)	0 (0.0)	0 (0.0)	0 (0.0)
Anemia	0 (0.0)	0 (0.0)	0 (0.0)	0 (0.0)	0 (0.0)	1 (33.3)	0 (0.0)	0 (0.0)	0 (0.0)	0 (0.0)
Neutropenia	0 (0.0)	0 (0.0)	0 (0.0)	0 (0.0)	0 (0.0)	0 (0.0)	0 (0.0)	0 (0.0)	0 (0.0)	0 (0.0)
Febrile neutropenia	0 (0.0)	0 (0.0)	0 (0.0)	0 (0.0)	0 (0.0)	0 (0.0)	0 (0.0)	0 (0.0)	0 (0.0)	0 (0.0)
Thrombocytopenia	0 (0.0)	0 (0.0)	0 (0.0)	0 (0.0)	0 (0.0)	0 (0.0)	0 (0.0)	0 (0.0)	0 (0.0)	0 (0.0)
**General disorder and administration site condition**	1 (33.3)	0 (0.0)	0 (0.0)	0 (0.0)	0 (0.0)	1 (33.3)	0 (0.0)	0 (0.0)	0 (0.0)	0 (0.0)
Fatigue	1 (33.3)	0 (0.0)	0 (0.0)	0 (0.0)	0 (0.0)	1 (33.3)	0 (0.0)	0 (0.0)	0 (0.0)	0 (0.0)
Asthenia	0 (0.0)	0 (0.0)	0 (0.0)	0 (0.0)	0 (0.0)	0 (0.0)	0 (0.0)	0 (0.0)	0 (0.0)	0 (0.0)

Supplementary Table 1: TEAEs by Worst Grade, SOC and PT - SAF Analysis Set (continued)

**Primary System Organ Class**<br>Preferred Term	**0.6 mg/kg n=3 (100%)**					**1.2 mg/kg n=3 (100%)**				
	**Any Grade n (%)**	**Grade ≥2 n (%)**	**Grade ≥3 n (%)**	**Grade ≥4 n (%)**	**Grade =5 n (%)**	**Any Grade n (%)**	**Grade ≥2 n (%)**	**Grade ≥3 n (%)**	**Grade ≥4 n (%)**	**Grade =5 n (%)**
Pyrexia	0 (0.0)	0 (0.0)	0 (0.0)	0 (0.0)	0 (0.0)	0 (0.0)	0 (0.0)	0 (0.0)	0 (0.0)	0 (0.0)
Oedema peripheral	0 (0.0)	0 (0.0)	0 (0.0)	0 (0.0)	0 (0.0)	0 (0.0)	0 (0.0)	0 (0.0)	0 (0.0)	0 (0.0)
Malaise	0 (0.0)	0 (0.0)	0 (0.0)	0 (0.0)	0 (0.0)	0 (0.0)	0 (0.0)	0 (0.0)	0 (0.0)	0 (0.0)
Medical device site fistula	0 (0.0)	0 (0.0)	0 (0.0)	0 (0.0)	0 (0.0)	0 (0.0)	0 (0.0)	0 (0.0)	0 (0.0)	0 (0.0)
Pain	0 (0.0)	0 (0.0)	0 (0.0)	0 (0.0)	0 (0.0)	0 (0.0)	0 (0.0)	0 (0.0)	0 (0.0)	0 (0.0)
Peripheral swelling	0 (0.0)	0 (0.0)	0 (0.0)	0 (0.0)	0 (0.0)	0 (0.0)	0 (0.0)	0 (0.0)	0 (0.0)	0 (0.0)
**Investigations**	0 (0.0)	0 (0.0)	0 (0.0)	0 (0.0)	0 (0.0)	3 (100.0)	2 (66.7)	1 (33.3)	0 (0.0)	0 (0.0)
Neutrophil count decreased	0 (0.0)	0 (0.0)	0 (0.0)	0 (0.0)	0 (0.0)	0 (0.0)	0 (0.0)	0 (0.0)	0 (0.0)	0 (0.0)
White blood cell count decreased	0 (0.0)	0 (0.0)	0 (0.0)	0 (0.0)	0 (0.0)	0 (0.0)	0 (0.0)	0 (0.0)	0 (0.0)	0 (0.0)
Platelet count decreased	0 (0.0)	0 (0.0)	0 (0.0)	0 (0.0)	0 (0.0)	0 (0.0)	0 (0.0)	0 (0.0)	0 (0.0)	0 (0.0)
Lymphocyte count decreased	0 (0.0)	0 (0.0)	0 (0.0)	0 (0.0)	0 (0.0)	1 (33.3)	1 (33.3)	1 (33.3)	0 (0.0)	0 (0.0)
Alanine aminotransferase increased	0 (0.0)	0 (0.0)	0 (0.0)	0 (0.0)	0 (0.0)	1 (33.3)	0 (0.0)	0 (0.0)	0 (0.0)	0 (0.0)
Lipase increased	0 (0.0)	0 (0.0)	0 (0.0)	0 (0.0)	0 (0.0)	0 (0.0)	0 (0.0)	0 (0.0)	0 (0.0)	0 (0.0)
Weight decreased	0 (0.0)	0 (0.0)	0 (0.0)	0 (0.0)	0 (0.0)	2 (66.7)	1 (33.3)	0 (0.0)	0 (0.0)	0 (0.0)
Aspartate aminotransferase increased	0 (0.0)	0 (0.0)	0 (0.0)	0 (0.0)	0 (0.0)	1 (33.3)	0 (0.0)	0 (0.0)	0 (0.0)	0 (0.0)
Gamma-glutamyltransferase increased	0 (0.0)	0 (0.0)	0 (0.0)	0 (0.0)	0 (0.0)	0 (0.0)	0 (0.0)	0 (0.0)	0 (0.0)	0 (0.0)
Amylase increased	0 (0.0)	0 (0.0)	0 (0.0)	0 (0.0)	0 (0.0)	0 (0.0)	0 (0.0)	0 (0.0)	0 (0.0)	0 (0.0)
Blood alkaline phosphatase increased	0 (0.0)	0 (0.0)	0 (0.0)	0 (0.0)	0 (0.0)	0 (0.0)	0 (0.0)	0 (0.0)	0 (0.0)	0 (0.0)
Blood bilirubin increased	0 (0.0)	0 (0.0)	0 (0.0)	0 (0.0)	0 (0.0)	0 (0.0)	0 (0.0)	0 (0.0)	0 (0.0)	0 (0.0)
Blood creatine increased	0 (0.0)	0 (0.0)	0 (0.0)	0 (0.0)	0 (0.0)	0 (0.0)	0 (0.0)	0 (0.0)	0 (0.0)	0 (0.0)
Blood creatinine increased	0 (0.0)	0 (0.0)	0 (0.0)	0 (0.0)	0 (0.0)	0 (0.0)	0 (0.0)	0 (0.0)	0 (0.0)	0 (0.0)
Blood creatinine decreased	0 (0.0)	0 (0.0)	0 (0.0)	0 (0.0)	0 (0.0)	0 (0.0)	0 (0.0)	0 (0.0)	0 (0.0)	0 (0.0)
**Metabolism and nutrition disorders**	0 (0.0)	0 (0.0)	0 (0.0)	0 (0.0)	0 (0.0)	1 (33.3)	0 (0.0)	0 (0.0)	0 (0.0)	0 (0.0)
Decreased appetite	0 (0.0)	0 (0.0)	0 (0.0)	0 (0.0)	0 (0.0)	1 (33.3)	0 (0.0)	0 (0.0)	0 (0.0)	0 (0.0)
Dehydration	0 (0.0)	0 (0.0)	0 (0.0)	0 (0.0)	0 (0.0)	0 (0.0)	0 (0.0)	0 (0.0)	0 (0.0)	0 (0.0)
Hypokalemia	0 (0.0)	0 (0.0)	0 (0.0)	0 (0.0)	0 (0.0)	0 (0.0)	0 (0.0)	0 (0.0)	0 (0.0)	0 (0.0)
Hypoalbuminemia	0 (0.0)	0 (0.0)	0 (0.0)	0 (0.0)	0 (0.0)	0 (0.0)	0 (0.0)	0 (0.0)	0 (0.0)	0 (0.0)
Hyponatremia	0 (0.0)	0 (0.0)	0 (0.0)	0 (0.0)	0 (0.0)	0 (0.0)	0 (0.0)	0 (0.0)	0 (0.0)	0 (0.0)
Hyperphosphatemia	0 (0.0)	0 (0.0)	0 (0.0)	0 (0.0)	0 (0.0)	0 (0.0)	0 (0.0)	0 (0.0)	0 (0.0)	0 (0.0)
Hypocalcemia	0 (0.0)	0 (0.0)	0 (0.0)	0 (0.0)	0 (0.0)	0 (0.0)	0 (0.0)	0 (0.0)	0 (0.0)	0 (0.0)

Supplementary Table 1: TEAEs by Worst Grade, SOC and PT - SAF Analysis Set (continued)

**Primary System Organ Class**<br>Preferred Term	**0.6 mg/kg<br>n=3 (100%)**					**1.2 mg/kg<br>n=3 (100%)**				
	**Any Grade<br>n (%)**	**Grade ≥2<br>n (%)**	**Grade ≥3<br>n (%)**	**Grade ≥4<br>n (%)**	**Grade =5<br>n (%)**	**Any Grade<br>n (%)**	**Grade ≥2<br>n (%)**	**Grade ≥3<br>n (%)**	**Grade ≥4<br>n (%)**	**Grade =5<br>n (%)**
Hypomagnesaemia	0 (0.0)	0 (0.0)	0 (0.0)	0 (0.0)	0 (0.0)	0 (0.0)	0 (0.0)	0 (0.0)	0 (0.0)	0 (0.0)
Hypophosphatemia	0 (0.0)	0 (0.0)	0 (0.0)	0 (0.0)	0 (0.0)	0 (0.0)	0 (0.0)	0 (0.0)	0 (0.0)	0 (0.0)
Vitamin D deficiency	0 (0.0)	0 (0.0)	0 (0.0)	0 (0.0)	0 (0.0)	0 (0.0)	0 (0.0)	0 (0.0)	0 (0.0)	0 (0.0)
**Infections and infestations**	1 (33.3)	1 (33.3)	0 (0.0)	0 (0.0)	0 (0.0)	0 (0.0)	0 (0.0)	0 (0.0)	0 (0.0)	0 (0.0)
COVID-19	0 (0.0)	0 (0.0)	0 (0.0)	0 (0.0)	0 (0.0)	0 (0.0)	0 (0.0)	0 (0.0)	0 (0.0)	0 (0.0)
Upper respiratory tract infection	0 (0.0)	0 (0.0)	0 (0.0)	0 (0.0)	0 (0.0)	0 (0.0)	0 (0.0)	0 (0.0)	0 (0.0)	0 (0.0)
Herpes zoster	0 (0.0)	0 (0.0)	0 (0.0)	0 (0.0)	0 (0.0)	0 (0.0)	0 (0.0)	0 (0.0)	0 (0.0)	0 (0.0)
Liver abscess	0 (0.0)	0 (0.0)	0 (0.0)	0 (0.0)	0 (0.0)	0 (0.0)	0 (0.0)	0 (0.0)	0 (0.0)	0 (0.0)
Metapneumovirus infection	0 (0.0)	0 (0.0)	0 (0.0)	0 (0.0)	0 (0.0)	0 (0.0)	0 (0.0)	0 (0.0)	0 (0.0)	0 (0.0)
Respiratory tract infection	0 (0.0)	0 (0.0)	0 (0.0)	0 (0.0)	0 (0.0)	0 (0.0)	0 (0.0)	0 (0.0)	0 (0.0)	0 (0.0)
Rhinitis	0 (0.0)	0 (0.0)	0 (0.0)	0 (0.0)	0 (0.0)	0 (0.0)	0 (0.0)	0 (0.0)	0 (0.0)	0 (0.0)
Sepsis	0 (0.0)	0 (0.0)	0 (0.0)	0 (0.0)	0 (0.0)	0 (0.0)	0 (0.0)	0 (0.0)	0 (0.0)	0 (0.0)
Septic shock	0 (0.0)	0 (0.0)	0 (0.0)	0 (0.0)	0 (0.0)	0 (0.0)	0 (0.0)	0 (0.0)	0 (0.0)	0 (0.0)
Skin infection	1 (33.3)	1 (33.3)	0 (0.0)	0 (0.0)	0 (0.0)	0 (0.0)	0 (0.0)	0 (0.0)	0 (0.0)	0 (0.0)
Systemic candida	0 (0.0)	0 (0.0)	0 (0.0)	0 (0.0)	0 (0.0)	0 (0.0)	0 (0.0)	0 (0.0)	0 (0.0)	0 (0.0)
Tooth infection	0 (0.0)	0 (0.0)	0 (0.0)	0 (0.0)	0 (0.0)	0 (0.0)	0 (0.0)	0 (0.0)	0 (0.0)	0 (0.0)
Vascular device infection	0 (0.0)	0 (0.0)	0 (0.0)	0 (0.0)	0 (0.0)	0 (0.0)	0 (0.0)	0 (0.0)	0 (0.0)	0 (0.0)
**Nervous system disorders**	0 (0.0)	0 (0.0)	0 (0.0)	0 (0.0)	0 (0.0)	0 (0.0)	0 (0.0)	0 (0.0)	0 (0.0)	0 (0.0)
Headache	0 (0.0)	0 (0.0)	0 (0.0)	0 (0.0)	0 (0.0)	0 (0.0)	0 (0.0)	0 (0.0)	0 (0.0)	0 (0.0)
Dizziness	0 (0.0)	0 (0.0)	0 (0.0)	0 (0.0)	0 (0.0)	0 (0.0)	0 (0.0)	0 (0.0)	0 (0.0)	0 (0.0)
Dysgeusia	0 (0.0)	0 (0.0)	0 (0.0)	0 (0.0)	0 (0.0)	0 (0.0)	0 (0.0)	0 (0.0)	0 (0.0)	0 (0.0)
Cerebrovascular accident	0 (0.0)	0 (0.0)	0 (0.0)	0 (0.0)	0 (0.0)	0 (0.0)	0 (0.0)	0 (0.0)	0 (0.0)	0 (0.0)
Cervical radiculopathy	0 (0.0)	0 (0.0)	0 (0.0)	0 (0.0)	0 (0.0)	0 (0.0)	0 (0.0)	0 (0.0)	0 (0.0)	0 (0.0)
Disturbance in attention	0 (0.0)	0 (0.0)	0 (0.0)	0 (0.0)	0 (0.0)	0 (0.0)	0 (0.0)	0 (0.0)	0 (0.0)	0 (0.0)
Hypoesthesia	0 (0.0)	0 (0.0)	0 (0.0)	0 (0.0)	0 (0.0)	0 (0.0)	0 (0.0)	0 (0.0)	0 (0.0)	0 (0.0)
Paresthesia	0 (0.0)	0 (0.0)	0 (0.0)	0 (0.0)	0 (0.0)	0 (0.0)	0 (0.0)	0 (0.0)	0 (0.0)	0 (0.0)
Peripheral sensory neuropathy	0 (0.0)	0 (0.0)	0 (0.0)	0 (0.0)	0 (0.0)	0 (0.0)	0 (0.0)	0 (0.0)	0 (0.0)	0 (0.0)
Seizure	0 (0.0)	0 (0.0)	0 (0.0)	0 (0.0)	0 (0.0)	0 (0.0)	0 (0.0)	0 (0.0)	0 (0.0)	0 (0.0)
Syncope	0 (0.0)	0 (0.0)	0 (0.0)	0 (0.0)	0 (0.0)	0 (0.0)	0 (0.0)	0 (0.0)	0 (0.0)	0 (0.0)

Supplementary Table 1: TEAEs by Worst Grade, SOC and PT - SAF Analysis Set (continued)

**Primary System Organ Class**<br>Preferred Term	**0.6 mg/kg**<br>**n=3 (100%)**					**1.2 mg/kg**<br>**n=3 (100%)**				
	**Any Grade**<br>**n (%)**	**Grade ≥2**<br>**n (%)**	**Grade ≥3**<br>**n (%)**	**Grade ≥4**<br>**n (%)**	**Grade =5**<br>**n (%)**	**Any Grade**<br>**n (%)**	**Grade ≥2**<br>**n (%)**	**Grade ≥3**<br>**n (%)**	**Grade ≥4**<br>**n (%)**	**Grade =5**<br>**n (%)**
**Respiratory, thoracic and mediastinal disorders**	0 (0.0)	0 (0.0)	0 (0.0)	0 (0.0)	0 (0.0)	2 (66.7)	0 (0.0)	0 (0.0)	0 (0.0)	0 (0.0)
Epistaxis	0 (0.0)	0 (0.0)	0 (0.0)	0 (0.0)	0 (0.0)	1 (33.3)	0 (0.0)	0 (0.0)	0 (0.0)	0 (0.0)
Cough	0 (0.0)	0 (0.0)	0 (0.0)	0 (0.0)	0 (0.0)	1 (33.3)	0 (0.0)	0 (0.0)	0 (0.0)	0 (0.0)
Dyspnea	0 (0.0)	0 (0.0)	0 (0.0)	0 (0.0)	0 (0.0)	0 (0.0)	0 (0.0)	0 (0.0)	0 (0.0)	0 (0.0)
Oropharyngeal pain	0 (0.0)	0 (0.0)	0 (0.0)	0 (0.0)	0 (0.0)	0 (0.0)	0 (0.0)	0 (0.0)	0 (0.0)	0 (0.0)
Dry throat	0 (0.0)	0 (0.0)	0 (0.0)	0 (0.0)	0 (0.0)	0 (0.0)	0 (0.0)	0 (0.0)	0 (0.0)	0 (0.0)
Hiccups	0 (0.0)	0 (0.0)	0 (0.0)	0 (0.0)	0 (0.0)	0 (0.0)	0 (0.0)	0 (0.0)	0 (0.0)	0 (0.0)
Nasal congestion	0 (0.0)	0 (0.0)	0 (0.0)	0 (0.0)	0 (0.0)	0 (0.0)	0 (0.0)	0 (0.0)	0 (0.0)	0 (0.0)
Oropharyngeal discomfort	0 (0.0)	0 (0.0)	0 (0.0)	0 (0.0)	0 (0.0)	0 (0.0)	0 (0.0)	0 (0.0)	0 (0.0)	0 (0.0)
Pleural effusion	0 (0.0)	0 (0.0)	0 (0.0)	0 (0.0)	0 (0.0)	0 (0.0)	0 (0.0)	0 (0.0)	0 (0.0)	0 (0.0)
Pneumonitis	0 (0.0)	0 (0.0)	0 (0.0)	0 (0.0)	0 (0.0)	0 (0.0)	0 (0.0)	0 (0.0)	0 (0.0)	0 (0.0)
Respiratory failure	0 (0.0)	0 (0.0)	0 (0.0)	0 (0.0)	0 (0.0)	0 (0.0)	0 (0.0)	0 (0.0)	0 (0.0)	0 (0.0)
**Musculoskeletal and connective tissue disorders**	0 (0.0)	0 (0.0)	0 (0.0)	0 (0.0)	0 (0.0)	1 (33.3)	0 (0.0)	0 (0.0)	0 (0.0)	0 (0.0)
Back pain	0 (0.0)	0 (0.0)	0 (0.0)	0 (0.0)	0 (0.0)	1 (33.3)	0 (0.0)	0 (0.0)	0 (0.0)	0 (0.0)
Arthralgia	0 (0.0)	0 (0.0)	0 (0.0)	0 (0.0)	0 (0.0)	0 (0.0)	0 (0.0)	0 (0.0)	0 (0.0)	0 (0.0)
Arthritis	0 (0.0)	0 (0.0)	0 (0.0)	0 (0.0)	0 (0.0)	0 (0.0)	0 (0.0)	0 (0.0)	0 (0.0)	0 (0.0)
Flank pain	0 (0.0)	0 (0.0)	0 (0.0)	0 (0.0)	0 (0.0)	0 (0.0)	0 (0.0)	0 (0.0)	0 (0.0)	0 (0.0)
Joint swelling	0 (0.0)	0 (0.0)	0 (0.0)	0 (0.0)	0 (0.0)	0 (0.0)	0 (0.0)	0 (0.0)	0 (0.0)	0 (0.0)
Limb discomfort	0 (0.0)	0 (0.0)	0 (0.0)	0 (0.0)	0 (0.0)	0 (0.0)	0 (0.0)	0 (0.0)	0 (0.0)	0 (0.0)
Muscle spasms	0 (0.0)	0 (0.0)	0 (0.0)	0 (0.0)	0 (0.0)	0 (0.0)	0 (0.0)	0 (0.0)	0 (0.0)	0 (0.0)
Musculoskeletal pain	0 (0.0)	0 (0.0)	0 (0.0)	0 (0.0)	0 (0.0)	0 (0.0)	0 (0.0)	0 (0.0)	0 (0.0)	0 (0.0)
Neck pain	0 (0.0)	0 (0.0)	0 (0.0)	0 (0.0)	0 (0.0)	0 (0.0)	0 (0.0)	0 (0.0)	0 (0.0)	0 (0.0)
**Skin and subcutaneous tissue disorders**	0 (0.0)	0 (0.0)	0 (0.0)	0 (0.0)	0 (0.0)	2 (66.7)	0 (0.0)	0 (0.0)	0 (0.0)	0 (0.0)
Alopecia	0 (0.0)	0 (0.0)	0 (0.0)	0 (0.0)	0 (0.0)	0 (0.0)	0 (0.0)	0 (0.0)	0 (0.0)	0 (0.0)
Dry skin	0 (0.0)	0 (0.0)	0 (0.0)	0 (0.0)	0 (0.0)	0 (0.0)	0 (0.0)	0 (0.0)	0 (0.0)	0 (0.0)
Pruritus	0 (0.0)	0 (0.0)	0 (0.0)	0 (0.0)	0 (0.0)	1 (33.3)	0 (0.0)	0 (0.0)	0 (0.0)	0 (0.0)
Rash	0 (0.0)	0 (0.0)	0 (0.0)	0 (0.0)	0 (0.0)	0 (0.0)	0 (0.0)	0 (0.0)	0 (0.0)	0 (0.0)
Dermatitis	0 (0.0)	0 (0.0)	0 (0.0)	0 (0.0)	0 (0.0)	0 (0.0)	0 (0.0)	0 (0.0)	0 (0.0)	0 (0.0)

Supplementary Table 1: TEAEs by Worst Grade, SOC and PT - SAF Analysis Set (continued)

**Primary System Organ Class** Preferred Term	**0.6 mg/kg n=3 (100%)**					**1.2 mg/kg n=3 (100%)**				
	**Any Grade n (%)**	**Grade ≥2 n (%)**	**Grade ≥3 n (%)**	**Grade ≥4 n (%)**	**Grade =5 n (%)**	**Any Grade n (%)**	**Grade ≥2 n (%)**	**Grade ≥3 n (%)**	**Grade ≥4 n (%)**	**Grade =5 n (%)**
Eczema	0 (0.0)	0 (0.0)	0 (0.0)	0 (0.0)	0 (0.0)	0 (0.0)	0 (0.0)	0 (0.0)	0 (0.0)	0 (0.0)
Petechiae	0 (0.0)	0 (0.0)	0 (0.0)	0 (0.0)	0 (0.0)	0 (0.0)	0 (0.0)	0 (0.0)	0 (0.0)	0 (0.0)
Photosensitivity reaction	0 (0.0)	0 (0.0)	0 (0.0)	0 (0.0)	0 (0.0)	1 (33.3)	0 (0.0)	0 (0.0)	0 (0.0)	0 (0.0)
**Injury, poisoning and procedural complications**	1 (33.3)	1 (33.3)	0 (0.0)	0 (0.0)	0 (0.0)	0 (0.0)	0 (0.0)	0 (0.0)	0 (0.0)	0 (0.0)
Stoma site hemorrhage	0 (0.0)	0 (0.0)	0 (0.0)	0 (0.0)	0 (0.0)	0 (0.0)	0 (0.0)	0 (0.0)	0 (0.0)	0 (0.0)
Back injury	0 (0.0)	0 (0.0)	0 (0.0)	0 (0.0)	0 (0.0)	0 (0.0)	0 (0.0)	0 (0.0)	0 (0.0)	0 (0.0)
Cervical vertebral fracture	0 (0.0)	0 (0.0)	0 (0.0)	0 (0.0)	0 (0.0)	0 (0.0)	0 (0.0)	0 (0.0)	0 (0.0)	0 (0.0)
Contusion	0 (0.0)	0 (0.0)	0 (0.0)	0 (0.0)	0 (0.0)	0 (0.0)	0 (0.0)	0 (0.0)	0 (0.0)	0 (0.0)
Fracture	0 (0.0)	0 (0.0)	0 (0.0)	0 (0.0)	0 (0.0)	0 (0.0)	0 (0.0)	0 (0.0)	0 (0.0)	0 (0.0)
Infusion related reaction	1 (33.3)	1 (33.3)	0 (0.0)	0 (0.0)	0 (0.0)	0 (0.0)	0 (0.0)	0 (0.0)	0 (0.0)	0 (0.0)
Postoperative wound complication	0 (0.0)	0 (0.0)	0 (0.0)	0 (0.0)	0 (0.0)	0 (0.0)	0 (0.0)	0 (0.0)	0 (0.0)	0 (0.0)
Procedural pain	0 (0.0)	0 (0.0)	0 (0.0)	0 (0.0)	0 (0.0)	0 (0.0)	0 (0.0)	0 (0.0)	0 (0.0)	0 (0.0)
Thermal burn	0 (0.0)	0 (0.0)	0 (0.0)	0 (0.0)	0 (0.0)	0 (0.0)	0 (0.0)	0 (0.0)	0 (0.0)	0 (0.0)
**Renal and urinary disorders**	0 (0.0)	0 (0.0)	0 (0.0)	0 (0.0)	0 (0.0)	1 (33.3)	1 (33.3)	1 (33.3)	0 (0.0)	0 (0.0)
Hematuria	0 (0.0)	0 (0.0)	0 (0.0)	0 (0.0)	0 (0.0)	1 (33.3)	1 (33.3)	1 (33.3)	0 (0.0)	0 (0.0)
Dysuria	0 (0.0)	0 (0.0)	0 (0.0)	0 (0.0)	0 (0.0)	0 (0.0)	0 (0.0)	0 (0.0)	0 (0.0)	0 (0.0)
Acute kidney injury	0 (0.0)	0 (0.0)	0 (0.0)	0 (0.0)	0 (0.0)	0 (0.0)	0 (0.0)	0 (0.0)	0 (0.0)	0 (0.0)
Pollakiuria	0 (0.0)	0 (0.0)	0 (0.0)	0 (0.0)	0 (0.0)	0 (0.0)	0 (0.0)	0 (0.0)	0 (0.0)	0 (0.0)
Proteinuria	0 (0.0)	0 (0.0)	0 (0.0)	0 (0.0)	0 (0.0)	0 (0.0)	0 (0.0)	0 (0.0)	0 (0.0)	0 (0.0)
**Neoplasms benign, malignant an unspecified (incl cysts and polyps)**	1 (33.3)	1 (33.3)	1 (33.3)	0 (0.0)	0 (0.0)	2 (66.7)	2 (66.7)	0 (0.0)	0 (0.0)	0 (0.0)
Tumor pain	1 (33.3)	1 (33.3)	1 (33.3)	0 (0.0)	0 (0.0)	1 (33.3)	1 (33.3)	0 (0.0)	0 (0.0)	0 (0.0)
Cancer pain	0 (0.0)	0 (0.0)	0 (0.0)	0 (0.0)	0 (0.0)	1 (33.3)	1 (33.3)	0 (0.0)	0 (0.0)	0 (0.0)
Colorectal adenoma	0 (0.0)	0 (0.0)	0 (0.0)	0 (0.0)	0 (0.0)	0 (0.0)	0 (0.0)	0 (0.0)	0 (0.0)	0 (0.0)
**Vascular disorders**	0 (0.0)	0 (0.0)	0 (0.0)	0 (0.0)	0 (0.0)	0 (0.0)	0 (0.0)	0 (0.0)	0 (0.0)	0 (0.0)
Hypotension	0 (0.0)	0 (0.0)	0 (0.0)	0 (0.0)	0 (0.0)	0 (0.0)	0 (0.0)	0 (0.0)	0 (0.0)	0 (0.0)
Hypertension	0 (0.0)	0 (0.0)	0 (0.0)	0 (0.0)	0 (0.0)	0 (0.0)	0 (0.0)	0 (0.0)	0 (0.0)	0 (0.0)

Supplementary Table 1: TEAEs by Worst Grade, SOC and PT - SAF Analysis Set (continued)

**Primary System Organ Class**<br>Preferred Term	**0.6 mg/kg**<br>**n=3 (100%)**					**1.2 mg/kg**<br>**n=3 (100%)**				
	**Any Grade n (%)**	**Grade ≥2 n (%)**	**Grade ≥3 n (%)**	**Grade ≥4 n (%)**	**Grade =5 n (%)**	**Any Grade n (%)**	**Grade ≥2 n (%)**	**Grade ≥3 n (%)**	**Grade ≥4 n (%)**	**Grade =5 n (%)**
Hypovolemic shock	0 (0.0)	0 (0.0)	0 (0.0)	0 (0.0)	0 (0.0)	0 (0.0)	0 (0.0)	0 (0.0)	0 (0.0)	0 (0.0)
**Psychiatric disorders**	0 (0.0)	0 (0.0)	0 (0.0)	0 (0.0)	0 (0.0)	0 (0.0)	0 (0.0)	0 (0.0)	0 (0.0)	0 (0.0)
Insomnia	0 (0.0)	0 (0.0)	0 (0.0)	0 (0.0)	0 (0.0)	0 (0.0)	0 (0.0)	0 (0.0)	0 (0.0)	0 (0.0)
Nervousness	0 (0.0)	0 (0.0)	0 (0.0)	0 (0.0)	0 (0.0)	0 (0.0)	0 (0.0)	0 (0.0)	0 (0.0)	0 (0.0)
Sleep disorder	0 (0.0)	0 (0.0)	0 (0.0)	0 (0.0)	0 (0.0)	0 (0.0)	0 (0.0)	0 (0.0)	0 (0.0)	0 (0.0)
**Reproductive system and breast disorders**	0 (0.0)	0 (0.0)	0 (0.0)	0 (0.0)	0 (0.0)	0 (0.0)	0 (0.0)	0 (0.0)	0 (0.0)	0 (0.0)
Intermenstrual bleeding	0 (0.0)	0 (0.0)	0 (0.0)	0 (0.0)	0 (0.0)	0 (0.0)	0 (0.0)	0 (0.0)	0 (0.0)	0 (0.0)
Pelvic pain	0 (0.0)	0 (0.0)	0 (0.0)	0 (0.0)	0 (0.0)	0 (0.0)	0 (0.0)	0 (0.0)	0 (0.0)	0 (0.0)
Perineal pain	0 (0.0)	0 (0.0)	0 (0.0)	0 (0.0)	0 (0.0)	0 (0.0)	0 (0.0)	0 (0.0)	0 (0.0)	0 (0.0)
**Cardiac disorders**	0 (0.0)	0 (0.0)	0 (0.0)	0 (0.0)	0 (0.0)	0 (0.0)	0 (0.0)	0 (0.0)	0 (0.0)	0 (0.0)
Palpitations	0 (0.0)	0 (0.0)	0 (0.0)	0 (0.0)	0 (0.0)	0 (0.0)	0 (0.0)	0 (0.0)	0 (0.0)	0 (0.0)
**Ear and labyrinth disorders**	0 (0.0)	0 (0.0)	0 (0.0)	0 (0.0)	0 (0.0)	0 (0.0)	0 (0.0)	0 (0.0)	0 (0.0)	0 (0.0)
Ear pain	0 (0.0)	0 (0.0)	0 (0.0)	0 (0.0)	0 (0.0)	0 (0.0)	0 (0.0)	0 (0.0)	0 (0.0)	0 (0.0)
**Endocrine disorders**	0 (0.0)	0 (0.0)	0 (0.0)	0 (0.0)	0 (0.0)	0 (0.0)	0 (0.0)	0 (0.0)	0 (0.0)	0 (0.0)
Hyperthyroidism	0 (0.0)	0 (0.0)	0 (0.0)	0 (0.0)	0 (0.0)	0 (0.0)	0 (0.0)	0 (0.0)	0 (0.0)	0 (0.0)
**Hepatobiliary disorders**	0 (0.0)	0 (0.0)	0 (0.0)	0 (0.0)	0 (0.0)	0 (0.0)	0 (0.0)	0 (0.0)	0 (0.0)	0 (0.0)
Cholangitis	0 (0.0)	0 (0.0)	0 (0.0)	0 (0.0)	0 (0.0)	0 (0.0)	0 (0.0)	0 (0.0)	0 (0.0)	0 (0.0)

Supplementary Table 1: TEAEs by Worst Grade, SOC and PT - SAF Analysis Set (continued)

**Primary System Organ Class**<br>Preferred Term	**2.4 mg/kg<br>n=7 (100%)**					**2.6 mg/kg<br>n=4 (100%)**				
	**Any Grade<br>n (%)**	**Grade ≥2<br>n (%)**	**Grade ≥3<br>n (%)**	**Grade ≥4<br>n (%)**	**Grade =5<br>n (%)**	**Any Grade<br>n (%)**	**Grade ≥2<br>n (%)**	**Grade ≥3<br>n (%)**	**Grade ≥4<br>n (%)**	**Grade =5<br>n (%)**
**Subjects with at least one Event**	7 (100.0)	6 (85.7)	3 (42.9)	2 (28.6)	0 (0.0)	4 (100.0)	4 (100.0)	3 (75.0)	2 (50.0)	0 (0.0)
**Gastrointestinal disorders**	6 (85.7)	2 (28.6)	0 (0.0)	0 (0.0)	0 (0.0)	4 (100.0)	3 (75.0)	1 (25.0)	0 (0.0)	0 (0.0)
Nausea	4 (57.1)	1 (14.3)	0 (0.0)	0 (0.0)	0 (0.0)	3 (75.0)	2 (50.0)	0 (0.0)	0 (0.0)	0 (0.0)
Vomiting	4 (57.1)	1 (14.3)	0 (0.0)	0 (0.0)	0 (0.0)	2 (50.0)	0 (0.0)	0 (0.0)	0 (0.0)	0 (0.0)
Diarrhea	2 (28.6)	1 (14.3)	0 (0.0)	0 (0.0)	0 (0.0)	0 (0.0)	0 (0.0)	0 (0.0)	0 (0.0)	0 (0.0)
Constipation	2 (28.6)	0 (0.0)	0 (0.0)	0 (0.0)	0 (0.0)	0 (0.0)	0 (0.0)	0 (0.0)	0 (0.0)	0 (0.0)
Stomatitis	0 (0.0)	0 (0.0)	0 (0.0)	0 (0.0)	0 (0.0)	1 (25.0)	1 (25.0)	0 (0.0)	0 (0.0)	0 (0.0)
Abdominal pain	1 (14.3)	0 (0.0)	0 (0.0)	0 (0.0)	0 (0.0)	0 (0.0)	0 (0.0)	0 (0.0)	0 (0.0)	0 (0.0)
Abdominal pain upper	0 (0.0)	0 (0.0)	0 (0.0)	0 (0.0)	0 (0.0)	0 (0.0)	0 (0.0)	0 (0.0)	0 (0.0)	0 (0.0)
Rectal hemorrhage	1 (14.3)	0 (0.0)	0 (0.0)	0 (0.0)	0 (0.0)	0 (0.0)	0 (0.0)	0 (0.0)	0 (0.0)	0 (0.0)
Abdominal distension	0 (0.0)	0 (0.0)	0 (0.0)	0 (0.0)	0 (0.0)	0 (0.0)	0 (0.0)	0 (0.0)	0 (0.0)	0 (0.0)
Anal hemorrhage	0 (0.0)	0 (0.0)	0 (0.0)	0 (0.0)	0 (0.0)	0 (0.0)	0 (0.0)	0 (0.0)	0 (0.0)	0 (0.0)
Ascites	0 (0.0)	0 (0.0)	0 (0.0)	0 (0.0)	0 (0.0)	0 (0.0)	0 (0.0)	0 (0.0)	0 (0.0)	0 (0.0)
Flatulence	0 (0.0)	0 (0.0)	0 (0.0)	0 (0.0)	0 (0.0)	0 (0.0)	0 (0.0)	0 (0.0)	0 (0.0)	0 (0.0)
Gastrointestinal hemorrhage	0 (0.0)	0 (0.0)	0 (0.0)	0 (0.0)	0 (0.0)	0 (0.0)	0 (0.0)	0 (0.0)	0 (0.0)	0 (0.0)
Hematochezia	0 (0.0)	0 (0.0)	0 (0.0)	0 (0.0)	0 (0.0)	0 (0.0)	0 (0.0)	0 (0.0)	0 (0.0)	0 (0.0)
Hemorrhoidal hemorrhage	1 (14.3)	0 (0.0)	0 (0.0)	0 (0.0)	0 (0.0)	0 (0.0)	0 (0.0)	0 (0.0)	0 (0.0)	0 (0.0)
Ileus	0 (0.0)	0 (0.0)	0 (0.0)	0 (0.0)	0 (0.0)	0 (0.0)	0 (0.0)	0 (0.0)	0 (0.0)	0 (0.0)
Intestinal obstruction	0 (0.0)	0 (0.0)	0 (0.0)	0 (0.0)	0 (0.0)	0 (0.0)	0 (0.0)	0 (0.0)	0 (0.0)	0 (0.0)
Upper gastrointestinal hemorrhage	0 (0.0)	0 (0.0)	0 (0.0)	0 (0.0)	0 (0.0)	1 (25.0)	1 (25.0)	1 (25.0)	0 (0.0)	0 (0.0)
**Blood and lymphatic system disorders**	6 (85.7)	4 (57.1)	3 (42.9)	1 (14.3)	0 (0.0)	2 (50.0)	2 (50.0)	1 (25.0)	0 (0.0)	0 (0.0)
Anemia	4 (57.1)	2 (28.6)	2 (28.6)	0 (0.0)	0 (0.0)	2 (50.0)	2 (50.0)	1 (25.0)	0 (0.0)	0 (0.0)
Neutropenia	3 (42.9)	3 (42.9)	2 (28.6)	1 (14.3)	0 (0.0)	0 (0.0)	0 (0.0)	0 (0.0)	0 (0.0)	0 (0.0)
Febrile neutropenia	1 (14.3)	1 (14.3)	1 (14.3)	0 (0.0)	0 (0.0)	0 (0.0)	0 (0.0)	0 (0.0)	0 (0.0)	0 (0.0)
Thrombocytopenia	0 (0.0)	0 (0.0)	0 (0.0)	0 (0.0)	0 (0.0)	0 (0.0)	0 (0.0)	0 (0.0)	0 (0.0)	0 (0.0)
**General disorder and administration site condition**	5 (71.4)	3 (42.9)	0 (0.0)	0 (0.0)	0 (0.0)	4 (100.0)	1 (25.0)	0 (0.0)	0 (0.0)	0 (0.0)
Fatigue	3 (42.9)	2 (28.6)	0 (0.0)	0 (0.0)	0 (0.0)	3 (75.0)	1 (25.0)	0 (0.0)	0 (0.0)	0 (0.0)
Asthenia	3 (42.9)	1 (14.3)	0 (0.0)	0 (0.0)	0 (0.0)	0 (0.0)	0 (0.0)	0 (0.0)	0 (0.0)	0 (0.0)

Supplementary Table 1: TEAEs by Worst Grade, SOC and PT - SAF Analysis Set (continued)

**Primary System Organ Class**<br>Preferred Term	**2.4 mg/kg**<br>**n=7 (100%)**					**2.6 mg/kg**<br>**n=4 (100%)**				
	**Any Grade n (%)**	**Grade ≥2 n (%)**	**Grade ≥3 n (%)**	**Grade ≥4 n (%)**	**Grade =5 n (%)**	**Any Grade n (%)**	**Grade ≥2 n (%)**	**Grade ≥3 n (%)**	**Grade ≥4 n (%)**	**Grade =5 n (%)**
Pyrexia	0 (0.0)	0 (0.0)	0 (0.0)	0 (0.0)	0 (0.0)	0 (0.0)	0 (0.0)	0 (0.0)	0 (0.0)	0 (0.0)
Oedema peripheral	0 (0.0)	0 (0.0)	0 (0.0)	0 (0.0)	0 (0.0)	1 (25.0)	0 (0.0)	0 (0.0)	0 (0.0)	0 (0.0)
Malaise	0 (0.0)	0 (0.0)	0 (0.0)	0 (0.0)	0 (0.0)	0 (0.0)	0 (0.0)	0 (0.0)	0 (0.0)	0 (0.0)
Medical device site fistula	0 (0.0)	0 (0.0)	0 (0.0)	0 (0.0)	0 (0.0)	0 (0.0)	0 (0.0)	0 (0.0)	0 (0.0)	0 (0.0)
Pain	1 (14.3)	0 (0.0)	0 (0.0)	0 (0.0)	0 (0.0)	0 (0.0)	0 (0.0)	0 (0.0)	0 (0.0)	0 (0.0)
Peripheral swelling	0 (0.0)	0 (0.0)	0 (0.0)	0 (0.0)	0 (0.0)	0 (0.0)	0 (0.0)	0 (0.0)	0 (0.0)	0 (0.0)
**Investigations**	5 (71.4)	4 (57.1)	2 (28.6)	1 (14.3)	0 (0.0)	3 (75.0)	3 (75.0)	2 (50.0)	2 (50.0)	0 (0.0)
Neutrophil count decreased	2 (28.6)	1 (14.3)	1 (14.3)	1 (14.3)	0 (0.0)	3 (75.0)	2 (50.0)	2 (50.0)	2 (50.0)	0 (0.0)
White blood cell count decreased	2 (28.6)	2 (28.6)	1 (14.3)	1 (14.3)	0 (0.0)	3 (75.0)	3 (75.0)	1 (25.0)	0 (0.0)	0 (0.0)
Platelet count decreased	2 (28.6)	2 (28.6)	2 (28.6)	1 (14.3)	0 (0.0)	2 (50.0)	1 (25.0)	1 (25.0)	0 (0.0)	0 (0.0)
Lymphocyte count decreased	1 (14.3)	1 (14.3)	1 (14.3)	0 (0.0)	0 (0.0)	1 (25.0)	1 (25.0)	0 (0.0)	0 (0.0)	0 (0.0)
Alanine aminotransferase increased	1 (14.3)	0 (0.0)	0 (0.0)	0 (0.0)	0 (0.0)	0 (0.0)	0 (0.0)	0 (0.0)	0 (0.0)	0 (0.0)
Lipase increased	2 (28.6)	1 (14.3)	0 (0.0)	0 (0.0)	0 (0.0)	0 (0.0)	0 (0.0)	0 (0.0)	0 (0.0)	0 (0.0)
Weight decreased	2 (28.6)	1 (14.3)	0 (0.0)	0 (0.0)	0 (0.0)	0 (0.0)	0 (0.0)	0 (0.0)	0 (0.0)	0 (0.0)
Aspartate aminotransferase increased	0 (0.0)	0 (0.0)	0 (0.0)	0 (0.0)	0 (0.0)	0 (0.0)	0 (0.0)	0 (0.0)	0 (0.0)	0 (0.0)
Gamma-glutamyltransferase increased	1 (14.3)	1 (14.3)	0 (0.0)	0 (0.0)	0 (0.0)	0 (0.0)	0 (0.0)	0 (0.0)	0 (0.0)	0 (0.0)
Amylase increased	0 (0.0)	0 (0.0)	0 (0.0)	0 (0.0)	0 (0.0)	0 (0.0)	0 (0.0)	0 (0.0)	0 (0.0)	0 (0.0)
Blood alkaline phosphatase increased	0 (0.0)	0 (0.0)	0 (0.0)	0 (0.0)	0 (0.0)	1 (25.0)	0 (0.0)	0 (0.0)	0 (0.0)	0 (0.0)
Blood bilirubin increased	0 (0.0)	0 (0.0)	0 (0.0)	0 (0.0)	0 (0.0)	0 (0.0)	0 (0.0)	0 (0.0)	0 (0.0)	0 (0.0)
Blood creatine increased	1 (14.3)	0 (0.0)	0 (0.0)	0 (0.0)	0 (0.0)	0 (0.0)	0 (0.0)	0 (0.0)	0 (0.0)	0 (0.0)
Blood creatinine increased	1 (14.3)	1 (14.3)	1 (14.3)	0 (0.0)	0 (0.0)	0 (0.0)	0 (0.0)	0 (0.0)	0 (0.0)	0 (0.0)
Blood creatinine decreased	0 (0.0)	0 (0.0)	0 (0.0)	0 (0.0)	0 (0.0)	1 (25.0)	0 (0.0)	0 (0.0)	0 (0.0)	0 (0.0)
**Metabolism and nutrition disorders**	5 (71.4)	3 (42.9)	0 (0.0)	0 (0.0)	0 (0.0)	3 (75.0)	1 (25.0)	0 (0.0)	0 (0.0)	0 (0.0)
Decreased appetite	4 (57.1)	1 (14.3)	0 (0.0)	0 (0.0)	0 (0.0)	2 (50.0)	1 (25.0)	0 (0.0)	0 (0.0)	0 (0.0)
Dehydration	0 (0.0)	0 (0.0)	0 (0.0)	0 (0.0)	0 (0.0)	1 (25.0)	1 (25.0)	0 (0.0)	0 (0.0)	0 (0.0)
Hypokalemia	2 (28.6)	0 (0.0)	0 (0.0)	0 (0.0)	0 (0.0)	0 (0.0)	0 (0.0)	0 (0.0)	0 (0.0)	0 (0.0)
Hypoalbuminemia	2 (28.6)	1 (14.3)	0 (0.0)	0 (0.0)	0 (0.0)	0 (0.0)	0 (0.0)	0 (0.0)	0 (0.0)	0 (0.0)
Hyponatremia	0 (0.0)	0 (0.0)	0 (0.0)	0 (0.0)	0 (0.0)	0 (0.0)	0 (0.0)	0 (0.0)	0 (0.0)	0 (0.0)
Hyperphosphatemia	0 (0.0)	0 (0.0)	0 (0.0)	0 (0.0)	0 (0.0)	1 (25.0)	0 (0.0)	0 (0.0)	0 (0.0)	0 (0.0)
Hypocalcemia	1 (14.3)	1 (14.3)	0 (0.0)	0 (0.0)	0 (0.0)	0 (0.0)	0 (0.0)	0 (0.0)	0 (0.0)	0 (0.0)

Supplementary Table 1: TEAEs by Worst Grade, SOC and PT - SAF Analysis Set (continued)

**Primary System Organ Class**<br>Preferred Term	**2.4 mg/kg**<br>**n=7 (100%)**					**2.6 mg/kg**<br>**n=4 (100%)**				
	**Any Grade**<br>**n (%)**	**Grade ≥2**<br>**n (%)**	**Grade ≥3**<br>**n (%)**	**Grade ≥4**<br>**n (%)**	**Grade =5**<br>**n (%)**	**Any Grade**<br>**n (%)**	**Grade ≥2**<br>**n (%)**	**Grade ≥3**<br>**n (%)**	**Grade ≥4**<br>**n (%)**	**Grade =5**<br>**n (%)**
Hypomagnesaemia	0 (0.0)	0 (0.0)	0 (0.0)	0 (0.0)	0 (0.0)	0 (0.0)	0 (0.0)	0 (0.0)	0 (0.0)	0 (0.0)
Hypophosphatemia	1 (14.3)	1 (14.3)	0 (0.0)	0 (0.0)	0 (0.0)	0 (0.0)	0 (0.0)	0 (0.0)	0 (0.0)	0 (0.0)
Vitamin D deficiency	0 (0.0)	0 (0.0)	0 (0.0)	0 (0.0)	0 (0.0)	0 (0.0)	0 (0.0)	0 (0.0)	0 (0.0)	0 (0.0)
**Infections and infestations**	3 (42.9)	2 (28.6)	1 (14.3)	0 (0.0)	0 (0.0)	0 (0.0)	0 (0.0)	0 (0.0)	0 (0.0)	0 (0.0)
COVID-19	1 (14.3)	1 (14.3)	0 (0.0)	0 (0.0)	0 (0.0)	0 (0.0)	0 (0.0)	0 (0.0)	0 (0.0)	0 (0.0)
Upper respiratory tract infection	0 (0.0)	0 (0.0)	0 (0.0)	0 (0.0)	0 (0.0)	0 (0.0)	0 (0.0)	0 (0.0)	0 (0.0)	0 (0.0)
Herpes zoster	0 (0.0)	0 (0.0)	0 (0.0)	0 (0.0)	0 (0.0)	0 (0.0)	0 (0.0)	0 (0.0)	0 (0.0)	0 (0.0)
Liver abscess	0 (0.0)	0 (0.0)	0 (0.0)	0 (0.0)	0 (0.0)	0 (0.0)	0 (0.0)	0 (0.0)	0 (0.0)	0 (0.0)
Metapneumovirus infection	0 (0.0)	0 (0.0)	0 (0.0)	0 (0.0)	0 (0.0)	0 (0.0)	0 (0.0)	0 (0.0)	0 (0.0)	0 (0.0)
Respiratory tract infection	1 (14.3)	0 (0.0)	0 (0.0)	0 (0.0)	0 (0.0)	0 (0.0)	0 (0.0)	0 (0.0)	0 (0.0)	0 (0.0)
Rhinitis	1 (14.3)	0 (0.0)	0 (0.0)	0 (0.0)	0 (0.0)	0 (0.0)	0 (0.0)	0 (0.0)	0 (0.0)	0 (0.0)
Sepsis	0 (0.0)	0 (0.0)	0 (0.0)	0 (0.0)	0 (0.0)	0 (0.0)	0 (0.0)	0 (0.0)	0 (0.0)	0 (0.0)
Septic shock	0 (0.0)	0 (0.0)	0 (0.0)	0 (0.0)	0 (0.0)	0 (0.0)	0 (0.0)	0 (0.0)	0 (0.0)	0 (0.0)
Skin infection	0 (0.0)	0 (0.0)	0 (0.0)	0 (0.0)	0 (0.0)	0 (0.0)	0 (0.0)	0 (0.0)	0 (0.0)	0 (0.0)
Systemic candida	0 (0.0)	0 (0.0)	0 (0.0)	0 (0.0)	0 (0.0)	0 (0.0)	0 (0.0)	0 (0.0)	0 (0.0)	0 (0.0)
Tooth infection	1 (14.3)	1 (14.3)	0 (0.0)	0 (0.0)	0 (0.0)	0 (0.0)	0 (0.0)	0 (0.0)	0 (0.0)	0 (0.0)
Vascular device infection	1 (14.3)	1 (14.3)	1 (14.3)	0 (0.0)	0 (0.0)	0 (0.0)	0 (0.0)	0 (0.0)	0 (0.0)	0 (0.0)
**Nervous system disorders**	4 (57.1)	1 (14.3)	0 (0.0)	0 (0.0)	0 (0.0)	1 (25.0)	0 (0.0)	0 (0.0)	0 (0.0)	0 (0.0)
Headache	0 (0.0)	0 (0.0)	0 (0.0)	0 (0.0)	0 (0.0)	0 (0.0)	0 (0.0)	0 (0.0)	0 (0.0)	0 (0.0)
Dizziness	2 (28.6)	0 (0.0)	0 (0.0)	0 (0.0)	0 (0.0)	0 (0.0)	0 (0.0)	0 (0.0)	0 (0.0)	0 (0.0)
Dysgeusia	1 (14.3)	0 (0.0)	0 (0.0)	0 (0.0)	0 (0.0)	0 (0.0)	0 (0.0)	0 (0.0)	0 (0.0)	0 (0.0)
Cerebrovascular accident	0 (0.0)	0 (0.0)	0 (0.0)	0 (0.0)	0 (0.0)	0 (0.0)	0 (0.0)	0 (0.0)	0 (0.0)	0 (0.0)
Cervical radiculopathy	1 (14.3)	1 (14.3)	0 (0.0)	0 (0.0)	0 (0.0)	0 (0.0)	0 (0.0)	0 (0.0)	0 (0.0)	0 (0.0)
Disturbance in attention	0 (0.0)	0 (0.0)	0 (0.0)	0 (0.0)	0 (0.0)	0 (0.0)	0 (0.0)	0 (0.0)	0 (0.0)	0 (0.0)
Hypoesthesia	0 (0.0)	0 (0.0)	0 (0.0)	0 (0.0)	0 (0.0)	0 (0.0)	0 (0.0)	0 (0.0)	0 (0.0)	0 (0.0)
Paresthesia	0 (0.0)	0 (0.0)	0 (0.0)	0 (0.0)	0 (0.0)	1 (25.0)	0 (0.0)	0 (0.0)	0 (0.0)	0 (0.0)
Peripheral sensory neuropathy	0 (0.0)	0 (0.0)	0 (0.0)	0 (0.0)	0 (0.0)	0 (0.0)	0 (0.0)	0 (0.0)	0 (0.0)	0 (0.0)
Seizure	0 (0.0)	0 (0.0)	0 (0.0)	0 (0.0)	0 (0.0)	0 (0.0)	0 (0.0)	0 (0.0)	0 (0.0)	0 (0.0)
Syncope	0 (0.0)	0 (0.0)	0 (0.0)	0 (0.0)	0 (0.0)	0 (0.0)	0 (0.0)	0 (0.0)	0 (0.0)	0 (0.0)

Supplementary Table 1: TEAEs by Worst Grade, SOC and PT - SAF Analysis Set (continued)

**Primary System Organ Class** Preferred Term	**2.4 mg/kg n=7 (100%)**					**2.6 mg/kg n=4 (100%)**				
	**Any Grade n (%)**	**Grade ≥2 n (%)**	**Grade ≥3 n (%)**	**Grade ≥4 n (%)**	**Grade =5 n (%)**	**Any Grade n (%)**	**Grade ≥2 n (%)**	**Grade ≥3 n (%)**	**Grade ≥4 n (%)**	**Grade =5 n (%)**
**Respiratory, thoracic and mediastina disorders**	4 (57.1)	2 (28.6)	0 (0.0)	0 (0.0)	0 (0.0)	0 (0.0)	0 (0.0)	0 (0.0)	0 (0.0)	0 (0.0)
Epistaxis	3 (42.9)	0 (0.0)	0 (0.0)	0 (0.0)	0 (0.0)	0 (0.0)	0 (0.0)	0 (0.0)	0 (0.0)	0 (0.0)
Cough	2 (28.6)	1 (14.3)	0 (0.0)	0 (0.0)	0 (0.0)	0 (0.0)	0 (0.0)	0 (0.0)	0 (0.0)	0 (0.0)
Dyspnea	2 (28.6)	1 (14.3)	0 (0.0)	0 (0.0)	0 (0.0)	0 (0.0)	0 (0.0)	0 (0.0)	0 (0.0)	0 (0.0)
Oropharyngeal pain	0 (0.0)	0 (0.0)	0 (0.0)	0 (0.0)	0 (0.0)	0 (0.0)	0 (0.0)	0 (0.0)	0 (0.0)	0 (0.0)
Dry throat	0 (0.0)	0 (0.0)	0 (0.0)	0 (0.0)	0 (0.0)	0 (0.0)	0 (0.0)	0 (0.0)	0 (0.0)	0 (0.0)
Hiccups	0 (0.0)	0 (0.0)	0 (0.0)	0 (0.0)	0 (0.0)	0 (0.0)	0 (0.0)	0 (0.0)	0 (0.0)	0 (0.0)
Nasal congestion	1 (14.3)	0 (0.0)	0 (0.0)	0 (0.0)	0 (0.0)	0 (0.0)	0 (0.0)	0 (0.0)	0 (0.0)	0 (0.0)
Oropharyngeal discomfort	0 (0.0)	0 (0.0)	0 (0.0)	0 (0.0)	0 (0.0)	0 (0.0)	0 (0.0)	0 (0.0)	0 (0.0)	0 (0.0)
Pleural effusion	1 (14.3)	1 (14.3)	0 (0.0)	0 (0.0)	0 (0.0)	0 (0.0)	0 (0.0)	0 (0.0)	0 (0.0)	0 (0.0)
Pneumonitis	1 (14.3)	0 (0.0)	0 (0.0)	0 (0.0)	0 (0.0)	0 (0.0)	0 (0.0)	0 (0.0)	0 (0.0)	0 (0.0)
Respiratory failure	0 (0.0)	0 (0.0)	0 (0.0)	0 (0.0)	0 (0.0)	0 (0.0)	0 (0.0)	0 (0.0)	0 (0.0)	0 (0.0)
**Musculoskeletal and connective tissue disorders**	2 (28.6)	0 (0.0)	0 (0.0)	0 (0.0)	0 (0.0)	1 (25.0)	0 (0.0)	0 (0.0)	0 (0.0)	0 (0.0)
Back pain	1 (14.3)	0 (0.0)	0 (0.0)	0 (0.0)	0 (0.0)	0 (0.0)	0 (0.0)	0 (0.0)	0 (0.0)	0 (0.0)
Arthralgia	0 (0.0)	0 (0.0)	0 (0.0)	0 (0.0)	0 (0.0)	0 (0.0)	0 (0.0)	0 (0.0)	0 (0.0)	0 (0.0)
Arthritis	0 (0.0)	0 (0.0)	0 (0.0)	0 (0.0)	0 (0.0)	0 (0.0)	0 (0.0)	0 (0.0)	0 (0.0)	0 (0.0)
Flank pain	0 (0.0)	0 (0.0)	0 (0.0)	0 (0.0)	0 (0.0)	0 (0.0)	0 (0.0)	0 (0.0)	0 (0.0)	0 (0.0)
Joint swelling	0 (0.0)	0 (0.0)	0 (0.0)	0 (0.0)	0 (0.0)	0 (0.0)	0 (0.0)	0 (0.0)	0 (0.0)	0 (0.0)
Limb discomfort	0 (0.0)	0 (0.0)	0 (0.0)	0 (0.0)	0 (0.0)	1 (25.0)	0 (0.0)	0 (0.0)	0 (0.0)	0 (0.0)
Muscle spasms	0 (0.0)	0 (0.0)	0 (0.0)	0 (0.0)	0 (0.0)	0 (0.0)	0 (0.0)	0 (0.0)	0 (0.0)	0 (0.0)
Musculoskeletal pain	0 (0.0)	0 (0.0)	0 (0.0)	0 (0.0)	0 (0.0)	0 (0.0)	0 (0.0)	0 (0.0)	0 (0.0)	0 (0.0)
Neck pain	1 (14.3)	0 (0.0)	0 (0.0)	0 (0.0)	0 (0.0)	0 (0.0)	0 (0.0)	0 (0.0)	0 (0.0)	0 (0.0)
**Skin and subcutaneous tissue disorders**	1 (14.3)	0 (0.0)	0 (0.0)	0 (0.0)	0 (0.0)	2 (50.0)	0 (0.0)	0 (0.0)	0 (0.0)	0 (0.0)
Alopecia	1 (14.3)	0 (0.0)	0 (0.0)	0 (0.0)	0 (0.0)	2 (50.0)	0 (0.0)	0 (0.0)	0 (0.0)	0 (0.0)
Dry skin	0 (0.0)	0 (0.0)	0 (0.0)	0 (0.0)	0 (0.0)	1 (25.0)	0 (0.0)	0 (0.0)	0 (0.0)	0 (0.0)
Pruritus	1 (14.3)	0 (0.0)	0 (0.0)	0 (0.0)	0 (0.0)	0 (0.0)	0 (0.0)	0 (0.0)	0 (0.0)	0 (0.0)
Rash	1 (14.3)	0 (0.0)	0 (0.0)	0 (0.0)	0 (0.0)	0 (0.0)	0 (0.0)	0 (0.0)	0 (0.0)	0 (0.0)
Dermatitis	0 (0.0)	0 (0.0)	0 (0.0)	0 (0.0)	0 (0.0)	0 (0.0)	0 (0.0)	0 (0.0)	0 (0.0)	0 (0.0)

Supplementary Table 1: TEAEs by Worst Grade, SOC and PT - SAF Analysis Set (continued)

**Primary System Organ Class**<br>Preferred Term	**2.4 mg/kg n=7 (100%)**					**2.6 mg/kg n=4 (100%)**				
	**Any Grade n (%)**	**Grade ≥2 n (%)**	**Grade ≥3 n (%)**	**Grade ≥4 n (%)**	**Grade =5 n (%)**	**Any Grade n (%)**	**Grade ≥2 n (%)**	**Grade ≥3 n (%)**	**Grade ≥4 n (%)**	**Grade =5 n (%)**
Eczema	0 (0.0)	0 (0.0)	0 (0.0)	0 (0.0)	0 (0.0)	0 (0.0)	0 (0.0)	0 (0.0)	0 (0.0)	0 (0.0)
Petechiae	0 (0.0)	0 (0.0)	0 (0.0)	0 (0.0)	0 (0.0)	0 (0.0)	0 (0.0)	0 (0.0)	0 (0.0)	0 (0.0)
Photosensitivity reaction	0 (0.0)	0 (0.0)	0 (0.0)	0 (0.0)	0 (0.0)	0 (0.0)	0 (0.0)	0 (0.0)	0 (0.0)	0 (0.0)
**Injury, poisoning and procedural complications**	2 (28.6)	1 (14.3)	0 (0.0)	0 (0.0)	0 (0.0)	2 (50.0)	0 (0.0)	0 (0.0)	0 (0.0)	0 (0.0)
Stoma site hemorrhage	1 (14.3)	0 (0.0)	0 (0.0)	0 (0.0)	0 (0.0)	0 (0.0)	0 (0.0)	0 (0.0)	0 (0.0)	0 (0.0)
Back injury	0 (0.0)	0 (0.0)	0 (0.0)	0 (0.0)	0 (0.0)	1 (25.0)	0 (0.0)	0 (0.0)	0 (0.0)	0 (0.0)
Cervical vertebral fracture	1 (14.3)	1 (14.3)	0 (0.0)	0 (0.0)	0 (0.0)	0 (0.0)	0 (0.0)	0 (0.0)	0 (0.0)	0 (0.0)
Contusion	0 (0.0)	0 (0.0)	0 (0.0)	0 (0.0)	0 (0.0)	0 (0.0)	0 (0.0)	0 (0.0)	0 (0.0)	0 (0.0)
Fracture	0 (0.0)	0 (0.0)	0 (0.0)	0 (0.0)	0 (0.0)	0 (0.0)	0 (0.0)	0 (0.0)	0 (0.0)	0 (0.0)
Infusion related reaction	0 (0.0)	0 (0.0)	0 (0.0)	0 (0.0)	0 (0.0)	0 (0.0)	0 (0.0)	0 (0.0)	0 (0.0)	0 (0.0)
Postoperative wound complication	0 (0.0)	0 (0.0)	0 (0.0)	0 (0.0)	0 (0.0)	0 (0.0)	0 (0.0)	0 (0.0)	0 (0.0)	0 (0.0)
Procedural pain	0 (0.0)	0 (0.0)	0 (0.0)	0 (0.0)	0 (0.0)	0 (0.0)	0 (0.0)	0 (0.0)	0 (0.0)	0 (0.0)
Thermal burn	0 (0.0)	0 (0.0)	0 (0.0)	0 (0.0)	0 (0.0)	1 (25.0)	0 (0.0)	0 (0.0)	0 (0.0)	0 (0.0)
**Renal and urinary disorders**	2 (28.6)	0 (0.0)	0 (0.0)	0 (0.0)	0 (0.0)	0 (0.0)	0 (0.0)	0 (0.0)	0 (0.0)	0 (0.0)
Hematuria	1 (14.3)	0 (0.0)	0 (0.0)	0 (0.0)	0 (0.0)	0 (0.0)	0 (0.0)	0 (0.0)	0 (0.0)	0 (0.0)
Dysuria	1 (14.3)	0 (0.0)	0 (0.0)	0 (0.0)	0 (0.0)	0 (0.0)	0 (0.0)	0 (0.0)	0 (0.0)	0 (0.0)
Acute kidney injury	0 (0.0)	0 (0.0)	0 (0.0)	0 (0.0)	0 (0.0)	0 (0.0)	0 (0.0)	0 (0.0)	0 (0.0)	0 (0.0)
Pollakiuria	1 (14.3)	0 (0.0)	0 (0.0)	0 (0.0)	0 (0.0)	0 (0.0)	0 (0.0)	0 (0.0)	0 (0.0)	0 (0.0)
Proteinuria	0 (0.0)	0 (0.0)	0 (0.0)	0 (0.0)	0 (0.0)	0 (0.0)	0 (0.0)	0 (0.0)	0 (0.0)	0 (0.0)
**Neoplasms benign, malignant an unspecified (incl cysts and polyps)**	1 (14.3)	0 (0.0)	0 (0.0)	0 (0.0)	0 (0.0)	0 (0.0)	0 (0.0)	0 (0.0)	0 (0.0)	0 (0.0)
Tumor pain	0 (0.0)	0 (0.0)	0 (0.0)	0 (0.0)	0 (0.0)	0 (0.0)	0 (0.0)	0 (0.0)	0 (0.0)	0 (0.0)
Cancer pain	0 (0.0)	0 (0.0)	0 (0.0)	0 (0.0)	0 (0.0)	0 (0.0)	0 (0.0)	0 (0.0)	0 (0.0)	0 (0.0)
Colorectal adenoma	1 (14.3)	0 (0.0)	0 (0.0)	0 (0.0)	0 (0.0)	0 (0.0)	0 (0.0)	0 (0.0)	0 (0.0)	0 (0.0)
**Vascular disorders**	1 (14.3)	1 (14.3)	1 (14.3)	0 (0.0)	0 (0.0)	0 (0.0)	0 (0.0)	0 (0.0)	0 (0.0)	0 (0.0)
Hypotension	1 (14.3)	0 (0.0)	0 (0.0)	0 (0.0)	0 (0.0)	0 (0.0)	0 (0.0)	0 (0.0)	0 (0.0)	0 (0.0)
Hypertension	1 (14.3)	1 (14.3)	1 (14.3)	0 (0.0)	0 (0.0)	0 (0.0)	0 (0.0)	0 (0.0)	0 (0.0)	0 (0.0)

Supplementary Table 1: TEAEs by Worst Grade, SOC and PT - SAF Analysis Set (continued)

**Primary System Organ Class**<br>Preferred Term	**2.4 mg/kg<br>n=7 (100%)**					**2.6 mg/kg<br>n=4 (100%)**				
	**Any Grade n (%)**	**Grade ≥2 n (%)**	**Grade ≥3 n (%)**	**Grade ≥4 n (%)**	**Grade =5 n (%)**	**Any Grade n (%)**	**Grade ≥2 n (%)**	**Grade ≥3 n (%)**	**Grade ≥4 n (%)**	**Grade =5 n (%)**
Hypovolemic shock	0 (0.0)	0 (0.0)	0 (0.0)	0 (0.0)	0 (0.0)	0 (0.0)	0 (0.0)	0 (0.0)	0 (0.0)	0 (0.0)
**Psychiatric disorders**	0 (0.0)	0 (0.0)	0 (0.0)	0 (0.0)	0 (0.0)	0 (0.0)	0 (0.0)	0 (0.0)	0 (0.0)	0 (0.0)
Insomnia	0 (0.0)	0 (0.0)	0 (0.0)	0 (0.0)	0 (0.0)	0 (0.0)	0 (0.0)	0 (0.0)	0 (0.0)	0 (0.0)
Nervousness	0 (0.0)	0 (0.0)	0 (0.0)	0 (0.0)	0 (0.0)	0 (0.0)	0 (0.0)	0 (0.0)	0 (0.0)	0 (0.0)
Sleep disorder	0 (0.0)	0 (0.0)	0 (0.0)	0 (0.0)	0 (0.0)	0 (0.0)	0 (0.0)	0 (0.0)	0 (0.0)	0 (0.0)
**Reproductive system and breast disorders**	1 (14.3)	0 (0.0)	0 (0.0)	0 (0.0)	0(0.0)	1 (25.0)	1 (25.0)	0 (0.0)	0 (0.0)	0 (0.0)
Intermenstrual bleeding	1 (14.3)	0 (0.0)	0 (0.0)	0 (0.0)	0 (0.0)	0 (0.0)	0 (0.0)	0 (0.0)	0 (0.0)	0 (0.0)
Pelvic pain	0 (0.0)	0 (0.0)	0 (0.0)	0 (0.0)	0 (0.0)	1 (25.0)	1 (25.0)	0 (0.0)	0 (0.0)	0 (0.0)
Perineal pain	0 (0.0)	0 (0.0)	0 (0.0)	0 (0.0)	0 (0.0)	0 (0.0)	0 (0.0)	0 (0.0)	0 (0.0)	0 (0.0)
**Cardiac disorders**	0 (0.0)	0 (0.0)	0 (0.0)	0 (0.0)	0 (0.0)	0 (0.0)	0 (0.0)	0 (0.0)	0 (0.0)	0 (0.0)
Palpitations	0 (0.0)	0 (0.0)	0 (0.0)	0 (0.0)	0 (0.0)	0 (0.0)	0 (0.0)	0 (0.0)	0 (0.0)	0 (0.0)
**Ear and labyrinth disorders**	0 (0.0)	0 (0.0)	0 (0.0)	0 (0.0)	0 (0.0)	0 (0.0)	0 (0.0)	0 (0.0)	0 (0.0)	0 (0.0)
Ear pain	0 (0.0)	0 (0.0)	0 (0.0)	0 (0.0)	0 (0.0)	0 (0.0)	0 (0.0)	0 (0.0)	0 (0.0)	0 (0.0)
**Endocrine disorders**	0 (0.0)	0 (0.0)	0 (0.0)	0 (0.0)	0 (0.0)	0 (0.0)	0 (0.0)	0 (0.0)	0 (0.0)	0 (0.0)
Hyperthyroidism	0 (0.0)	0 (0.0)	0 (0.0)	0 (0.0)	0 (0.0)	0 (0.0)	0 (0.0)	0 (0.0)	0 (0.0)	0 (0.0)
**Hepatobiliary disorders**	0 (0.0)	0 (0.0)	0 (0.0)	0 (0.0)	0 (0.0)	0 (0.0)	0 (0.0)	0 (0.0)	0 (0.0)	0 (0.0)
Cholangitis	0 (0.0)	0 (0.0)	0 (0.0)	0 (0.0)	0 (0.0)	0 (0.0)	0 (0.0)	0 (0.0)	0 (0.0)	0 (0.0)

Supplementary Table 1: TEAEs by Worst Grade, SOC and PT - SAF Analysis Set (continued)

**Primary System Organ Class**<br>Preferred Term	**2.8 mg/kg**<br>**n=12 (100%)**					**3.0 mg/kg**<br>**n=4 (100%)**				
	**Any Grade n (%)**	**Grade ≥2 n (%)**	**Grade ≥3 n (%)**	**Grade ≥4 n (%)**	**Grade =5 n (%)**	**Any Grade n (%)**	**Grade ≥2 n (%)**	**Grade ≥3 n (%)**	**Grade ≥4 n (%)**	**Grade =5 n (%)**
**Subjects with at least one Event**	12 (100.0)	11 (91.7)	9 (75.0)	5 (41.7)	2 (16.7)	4 (100.0)	4 (100.0)	4 (100.0)	2 (50.0)	0 (0.0)
**Gastrointestinal disorders**	11 (91.7)	4 (33.3)	1 (8.3)	1 (8.3)	1 (8.3)	4 (100.0)	3 (75.0)	0 (0.0)	0 (0.0)	0 (0.0)
Nausea	7 (58.3)	3 (25.0)	0 (0.0)	0 (0.0)	0 (0.0)	2 (50.0)	2 (50.0)	0 (0.0)	0 (0.0)	0 (0.0)
Vomiting	2 (16.7)	0 (0.0)	0 (0.0)	0 (0.0)	0 (0.0)	1 (25.0)	0 (0.0)	0 (0.0)	0 (0.0)	0 (0.0)
Diarrhea	5 (41.7)	0 (0.0)	0 (0.0)	0 (0.0)	0 (0.0)	1 (25.0)	1 (25.0)	0 (0.0)	0 (0.0)	0 (0.0)
Constipation	3 (25.0)	1 (8.3)	0 (0.0)	0 (0.0)	0 (0.0)	1 (25.0)	0 (0.0)	0 (0.0)	0 (0.0)	0 (0.0)
Stomatitis	1 (8.3)	0 (0.0)	0 (0.0)	0 (0.0)	0 (0.0)	0 (0.0)	0 (0.0)	0 (0.0)	0 (0.0)	0 (0.0)
Abdominal pain	1 (8.3)	1 (8.3)	0 (0.0)	0 (0.0)	0 (0.0)	1 (25.0)	1 (25.0)	0 (0.0)	0 (0.0)	0 (0.0)
Abdominal pain upper	0 (0.0)	0 (0.0)	0 (0.0)	0 (0.0)	0 (0.0)	1 (25.0)	0 (0.0)	0 (0.0)	0 (0.0)	0 (0.0)
Rectal hemorrhage	1 (8.3)	0 (0.0)	0 (0.0)	0 (0.0)	0 (0.0)	0 (0.0)	0 (0.0)	0 (0.0)	0 (0.0)	0 (0.0)
Abdominal distension	0 (0.0)	0 (0.0)	0 (0.0)	0 (0.0)	0 (0.0)	0 (0.0)	0 (0.0)	0 (0.0)	0 (0.0)	0 (0.0)
Anal hemorrhage	0 (0.0)	0 (0.0)	0 (0.0)	0 (0.0)	0 (0.0)	0 (0.0)	0 (0.0)	0 (0.0)	0 (0.0)	0 (0.0)
Ascites	0 (0.0)	0 (0.0)	0 (0.0)	0 (0.0)	0 (0.0)	1 (25.0)	1 (25.0)	0 (0.0)	0 (0.0)	0 (0.0)
Flatulence	1 (8.3)	0 (0.0)	0 (0.0)	0 (0.0)	0 (0.0)	0 (0.0)	0 (0.0)	0 (0.0)	0 (0.0)	0 (0.0)
Gastrointestinal hemorrhage	1 (8.3)	1 (8.3)	1 (8.3)	1 (8.3)	1 (8.3)	0 (0.0)	0 (0.0)	0 (0.0)	0 (0.0)	0 (0.0)
Hematochezia	1 (8.3)	0 (0.0)	0 (0.0)	0 (0.0)	0 (0.0)	0 (0.0)	0 (0.0)	0 (0.0)	0 (0.0)	0 (0.0)
Hemorrhoidal hemorrhage	0 (0.0)	0 (0.0)	0 (0.0)	0 (0.0)	0 (0.0)	0 (0.0)	0 (0.0)	0 (0.0)	0 (0.0)	0 (0.0)
Ileus	0 (0.0)	0 (0.0)	0 (0.0)	0 (0.0)	0 (0.0)	1 (25.0)	1 (25.0)	0 (0.0)	0 (0.0)	0 (0.0)
Intestinal obstruction	0 (0.0)	0 (0.0)	0 (0.0)	0 (0.0)	0 (0.0)	0 (0.0)	0 (0.0)	0 (0.0)	0 (0.0)	0 (0.0)
Upper gastrointestinal hemorrhage	0 (0.0)	0 (0.0)	0 (0.0)	0 (0.0)	0 (0.0)	0 (0.0)	0 (0.0)	0 (0.0)	0 (0.0)	0 (0.0)
**Blood and lymphatic system disorders**	10 (83.3)	10 (83.3)	8 (66.7)	1 (8.3)	0 (0.0)	4 (100.0)	4 (100.0)	2 (50.0)	0 (0.0)	0 (0.0)
Anemia	9 (75.0)	9 (75.0)	6 (50.0)	0 (0.0)	0 (0.0)	4 (100.0)	4 (100.0)	1 (25.0)	0 (0.0)	0 (0.0)
Neutropenia	2 (16.7)	2 (16.7)	2 (16.7)	1 (8.3)	0 (0.0)	1 (25.0)	1 (25.0)	1 (25.0)	0 (0.0)	0 (0.0)
Febrile neutropenia	0 (0.0)	0 (0.0)	0 (0.0)	0 (0.0)	0 (0.0)	0 (0.0)	0 (0.0)	0 (0.0)	0 (0.0)	0 (0.0)
Thrombocytopenia	0 (0.0)	0 (0.0)	0 (0.0)	0 (0.0)	0 (0.0)	0 (0.0)	0 (0.0)	0 (0.0)	0 (0.0)	0 (0.0)
**General disorder and administration site condition**	9 (75.0)	4 (33.3)	1 (8.3)	0 (0.0)	0 (0.0)	3 (75.0)	0 (0.0)	0 (0.0)	0 (0.0)	0 (0.0)
Fatigue	8 (66.7)	3 (25.0)	0 (0.0)	0 (0.0)	0 (0.0)	2 (50.0)	0 (0.0)	0 (0.0)	0 (0.0)	0 (0.0)
Asthenia	1 (8.3)	1 (8.3)	1 (8.3)	0 (0.0)	0 (0.0)	0 (0.0)	0 (0.0)	0 (0.0)	0 (0.0)	0 (0.0)

Supplementary Table 1: TEAEs by Worst Grade, SOC and PT - SAF Analysis Set (continued)

**Primary System Organ Class**<br>Preferred Term	**2.8 mg/kg<br>n=12 (100%)**					**3.0 mg/kg<br>n=4 (100%)**				
	**Any Grade<br>n (%)**	**Grade ≥2<br>n (%)**	**Grade ≥3<br>n (%)**	**Grade ≥4<br>n (%)**	**Grade =5<br>n (%)**	**Any Grade<br>n (%)**	**Grade ≥2<br>n (%)**	**Grade ≥3<br>n (%)**	**Grade ≥4<br>n (%)**	**Grade =5<br>n (%)**
Pyrexia	2 (16.7)	0 (0.0)	0 (0.0)	0 (0.0)	0 (0.0)	0 (0.0)	0 (0.0)	0 (0.0)	0 (0.0)	0 (0.0)
Oedema peripheral	0 (0.0)	0 (0.0)	0 (0.0)	0 (0.0)	0 (0.0)	1 (25.0)	0 (0.0)	0 (0.0)	0 (0.0)	0 (0.0)
Malaise	0 (0.0)	0 (0.0)	0 (0.0)	0 (0.0)	0 (0.0)	0 (0.0)	0 (0.0)	0 (0.0)	0 (0.0)	0 (0.0)
Medical device site fistula	0 (0.0)	0 (0.0)	0 (0.0)	0 (0.0)	0 (0.0)	0 (0.0)	0 (0.0)	0 (0.0)	0 (0.0)	0 (0.0)
Pain	0 (0.0)	0 (0.0)	0 (0.0)	0 (0.0)	0 (0.0)	0 (0.0)	0 (0.0)	0 (0.0)	0 (0.0)	0 (0.0)
Peripheral swelling	1 (8.3)	0 (0.0)	0 (0.0)	0 (0.0)	0 (0.0)	0 (0.0)	0 (0.0)	0 (0.0)	0 (0.0)	0 (0.0)
**Investigations**	7 (58.3)	6 (50.0)	5 (41.7)	5 (41.7)	0 (0.0)	4 (100.0)	4 (100.0)	4 (100.0)	2 (50.0)	0 (0.0)
Neutrophil count decreased	5 (41.7)	5 (41.7)	4 (33.3)	3 (25.0)	0 (0.0)	2 (50.0)	2 (50.0)	2 (50.0)	1 (25.0)	0 (0.0)
White blood cell count decreased	5 (41.7)	5 (41.7)	4 (33.3)	4 (33.3)	0 (0.0)	1 (25.0)	1 (25.0)	1 (25.0)	0 (0.0)	0 (0.0)
Platelet count decreased	5 (41.7)	3 (25.0)	3 (25.0)	2 (16.7)	0 (0.0)	2 (50.0)	2 (50.0)	2 (50.0)	1 (25.0)	0 (0.0)
Lymphocyte count decreased	2 (16.7)	2 (16.7)	2 (16.7)	0 (0.0)	0 (0.0)	2 (50.0)	2 (50.0)	2 (50.0)	1 (25.0)	0 (0.0)
Alanine aminotransferase increased	0 (0.0)	0 (0.0)	0 (0.0)	0 (0.0)	0 (0.0)	1 (25.0)	0 (0.0)	0 (0.0)	0 (0.0)	0 (0.0)
Lipase increased	1 (8.3)	0 (0.0)	0 (0.0)	0 (0.0)	0 (0.0)	1 (25.0)	1 (25.0)	0 (0.0)	0 (0.0)	0 (0.0)
Weight decreased	1 (8.3)	1 (8.3)	0 (0.0)	0 (0.0)	0 (0.0)	0 (0.0)	0 (0.0)	0 (0.0)	0 (0.0)	0 (0.0)
Aspartate aminotransferase increased	1 (8.3)	0 (0.0)	0 (0.0)	0 (0.0)	0 (0.0)	1 (25.0)	0 (0.0)	0 (0.0)	0 (0.0)	0 (0.0)
Gamma-glutamyltransferase increased	1 (8.3)	0 (0.0)	0 (0.0)	0 (0.0)	0 (0.0)	1 (25.0)	0 (0.0)	0 (0.0)	0 (0.0)	0 (0.0)
Amylase increased	1 (8.3)	0 (0.0)	0 (0.0)	0 (0.0)	0 (0.0)	0 (0.0)	0 (0.0)	0 (0.0)	0 (0.0)	0 (0.0)
Blood alkaline phosphatase increased	0 (0.0)	0 (0.0)	0 (0.0)	0 (0.0)	0 (0.0)	0 (0.0)	0 (0.0)	0 (0.0)	0 (0.0)	0 (0.0)
Blood bilirubin increased	1 (8.3)	0 (0.0)	0 (0.0)	0 (0.0)	0 (0.0)	0 (0.0)	0 (0.0)	0 (0.0)	0 (0.0)	0 (0.0)
Blood creatine increased	0 (0.0)	0 (0.0)	0 (0.0)	0 (0.0)	0 (0.0)	0 (0.0)	0 (0.0)	0 (0.0)	0 (0.0)	0 (0.0)
Blood creatinine increased	0 (0.0)	0 (0.0)	0 (0.0)	0 (0.0)	0 (0.0)	0 (0.0)	0 (0.0)	0 (0.0)	0 (0.0)	0 (0.0)
Blood creatinine decreased	0 (0.0)	0 (0.0)	0 (0.0)	0 (0.0)	0 (0.0)	0 (0.0)	0 (0.0)	0 (0.0)	0 (0.0)	0 (0.0)
**Metabolism and nutrition disorders**	5 (41.7)	3 (25.0)	0 (0.0)	0 (0.0)	0 (0.0)	3 (75.0)	2 (50.0)	1 (25.0)	0 (0.0)	0 (0.0)
Decreased appetite	2 (16.7)	1 (8.3)	0 (0.0)	0 (0.0)	0 (0.0)	0 (0.0)	0 (0.0)	0 (0.0)	0 (0.0)	0 (0.0)
Dehydration	3 (25.0)	3 (25.0)	0 (0.0)	0 (0.0)	0 (0.0)	1 (25.0)	1 (25.0)	0 (0.0)	0 (0.0)	0 (0.0)
Hypokalemia	1 (8.3)	1 (8.3)	0 (0.0)	0 (0.0)	0 (0.0)	1 (25.0)	0 (0.0)	0 (0.0)	0 (0.0)	0 (0.0)
Hypoalbuminemia	0 (0.0)	0 (0.0)	0 (0.0)	0 (0.0)	0 (0.0)	0 (0.0)	0 (0.0)	0 (0.0)	0 (0.0)	0 (0.0)
Hyponatremia	2 (16.7)	0 (0.0)	0 (0.0)	0 (0.0)	0 (0.0)	1 (25.0)	1 (25.0)	1 (25.0)	0 (0.0)	0 (0.0)
Hyperphosphatemia	0 (0.0)	0 (0.0)	0 (0.0)	0 (0.0)	0 (0.0)	0 (0.0)	0 (0.0)	0 (0.0)	0 (0.0)	0 (0.0)
Hypocalcemia	0 (0.0)	0 (0.0)	0 (0.0)	0 (0.0)	0 (0.0)	0 (0.0)	0 (0.0)	0 (0.0)	0 (0.0)	0 (0.0)

Supplementary Table 1: TEAEs by Worst Grade, SOC and PT - SAF Analysis Set (continued)

**Primary System Organ Class**<br>Preferred Term	**2.8 mg/kg**<br>**n=12 (100%)**					**3.0 mg/kg**<br>**n=4 (100%)**				
	**Any Grade n (%)**	**Grade ≥2 n (%)**	**Grade ≥3 n (%)**	**Grade ≥4 n (%)**	**Grade =5 n (%)**	**Any Grade n (%)**	**Grade ≥2 n (%)**	**Grade ≥3 n (%)**	**Grade ≥4 n (%)**	**Grade =5 n (%)**
Hypomagnesaemia	1 (8.3)	0 (0.0)	0 (0.0)	0 (0.0)	0 (0.0)	0 (0.0)	0 (0.0)	0 (0.0)	0 (0.0)	0 (0.0)
Hypophosphatemia	0 (0.0)	0 (0.0)	0 (0.0)	0 (0.0)	0 (0.0)	0 (0.0)	0 (0.0)	0 (0.0)	0 (0.0)	0 (0.0)
Vitamin D deficiency	0 (0.0)	0 (0.0)	0 (0.0)	0 (0.0)	0 (0.0)	0 (0.0)	0 (0.0)	0 (0.0)	0 (0.0)	0 (0.0)
**Infections and infestations**	4 (33.3)	3 (25.0)	1 (8.3)	1 (8.3)	1 (8.3)	3 (75.0)	3 (75.0)	2 (50.0)	0 (0.0)	0 (0.0)
COVID-19	0 (0.0)	0 (0.0)	0 (0.0)	0 (0.0)	0 (0.0)	2 (50.0)	2 (50.0)	1 (25.0)	0 (0.0)	0 (0.0)
Upper respiratory tract infection	1 (8.3)	1 (8.3)	0 (0.0)	0 (0.0)	0 (0.0)	0 (0.0)	0 (0.0)	0 (0.0)	0 (0.0)	0 (0.0)
Herpes zoster	1 (8.3)	1 (8.3)	0 (0.0)	0 (0.0)	0 (0.0)	0 (0.0)	0 (0.0)	0 (0.0)	0 (0.0)	0 (0.0)
Liver abscess	0 (0.0)	0 (0.0)	0 (0.0)	0 (0.0)	0 (0.0)	0 (0.0)	0 (0.0)	0 (0.0)	0 (0.0)	0 (0.0)
Metapneumovirus infection	1 (8.3)	0 (0.0)	0 (0.0)	0 (0.0)	0 (0.0)	0 (0.0)	0 (0.0)	0 (0.0)	0 (0.0)	0 (0.0)
Respiratory tract infection	0 (0.0)	0 (0.0)	0 (0.0)	0 (0.0)	0 (0.0)	0 (0.0)	0 (0.0)	0 (0.0)	0 (0.0)	0 (0.0)
Rhinitis	0 (0.0)	0 (0.0)	0 (0.0)	0 (0.0)	0 (0.0)	0 (0.0)	0 (0.0)	0 (0.0)	0 (0.0)	0 (0.0)
Sepsis	1 (8.3)	1 (8.3)	1 (8.3)	1 (8.3)	1 (8.3)	0 (0.0)	0 (0.0)	0 (0.0)	0 (0.0)	0 (0.0)
Septic shock	0 (0.0)	0 (0.0)	0 (0.0)	0 (0.0)	0 (0.0)	0 (0.0)	0 (0.0)	0 (0.0)	0 (0.0)	0 (0.0)
Skin infection	0 (0.0)	0 (0.0)	0 (0.0)	0 (0.0)	0 (0.0)	0 (0.0)	0 (0.0)	0 (0.0)	0 (0.0)	0 (0.0)
Systemic candida	0 (0.0)	0 (0.0)	0 (0.0)	0 (0.0)	0 (0.0)	1 (25.0)	1 (25.0)	1 (25.0)	0 (0.0)	0 (0.0)
Tooth infection	0 (0.0)	0 (0.0)	0 (0.0)	0 (0.0)	0 (0.0)	0 (0.0)	0 (0.0)	0 (0.0)	0 (0.0)	0 (0.0)
Vascular device infection	0 (0.0)	0 (0.0)	0 (0.0)	0 (0.0)	0 (0.0)	0 (0.0)	0 (0.0)	0 (0.0)	0 (0.0)	0 (0.0)
**Nervous system disorders**	5 (41.7)	2 (16.7)	0 (0.0)	0 (0.0)	0 (0.0)	1 (25.0)	0 (0.0)	0 (0.0)	0 (0.0)	0 (0.0)
Headache	2 (16.7)	0 (0.0)	0 (0.0)	0 (0.0)	0 (0.0)	0 (0.0)	0 (0.0)	0 (0.0)	0 (0.0)	0 (0.0)
Dizziness	0 (0.0)	0 (0.0)	0 (0.0)	0 (0.0)	0 (0.0)	0 (0.0)	0 (0.0)	0 (0.0)	0 (0.0)	0 (0.0)
Dysgeusia	1 (8.3)	0 (0.0)	0 (0.0)	0 (0.0)	0 (0.0)	0 (0.0)	0 (0.0)	0 (0.0)	0 (0.0)	0 (0.0)
Cerebrovascular accident	1 (8.3)	1 (8.3)	0 (0.0)	0 (0.0)	0 (0.0)	0 (0.0)	0 (0.0)	0 (0.0)	0 (0.0)	0 (0.0)
Cervical radiculopathy	0 (0.0)	0 (0.0)	0 (0.0)	0 (0.0)	0 (0.0)	0 (0.0)	0 (0.0)	0 (0.0)	0 (0.0)	0 (0.0)
Disturbance in attention	0 (0.0)	0 (0.0)	0 (0.0)	0 (0.0)	0 (0.0)	1 (25.0)	0 (0.0)	0 (0.0)	0 (0.0)	0 (0.0)
Hypoesthesia	0 (0.0)	0 (0.0)	0 (0.0)	0 (0.0)	0 (0.0)	0 (0.0)	0 (0.0)	0 (0.0)	0 (0.0)	0 (0.0)
Paresthesia	0 (0.0)	0 (0.0)	0 (0.0)	0 (0.0)	0 (0.0)	0 (0.0)	0 (0.0)	0 (0.0)	0 (0.0)	0 (0.0)
Peripheral sensory neuropathy	1 (8.3)	1 (8.3)	0 (0.0)	0 (0.0)	0 (0.0)	0 (0.0)	0 (0.0)	0 (0.0)	0 (0.0)	0 (0.0)
Seizure	0 (0.0)	0 (0.0)	0 (0.0)	0 (0.0)	0 (0.0)	0 (0.0)	0 (0.0)	0 (0.0)	0 (0.0)	0 (0.0)
Syncope	1 (8.3)	0 (0.0)	0 (0.0)	0 (0.0)	0 (0.0)	0 (0.0)	0 (0.0)	0 (0.0)	0 (0.0)	0 (0.0)

Supplementary Table 1: TEAEs by Worst Grade, SOC and PT - SAF Analysis Set (continued)

**Primary System Organ Class** Preferred Term	**2.8 mg/kg n=12 (100%)**					**3.0 mg/kg n=4 (100%)**				
	**Any Grade n (%)**	**Grade ≥2 n (%)**	**Grade ≥3 n (%)**	**Grade ≥4 n (%)**	**Grade =5 n (%)**	**Any Grade n (%)**	**Grade ≥2 n (%)**	**Grade ≥3 n (%)**	**Grade ≥4 n (%)**	**Grade =5 n (%)**
**Respiratory, thoracic and mediastina disorders**	2 (16.7)	1 (8.3)	1 (8.3)	0 (0.0)	0 (0.0)	1 (25.0)	0 (0.0)	0 (0.0)	0 (0.0)	0 (0.0)
Epistaxis	0 (0.0)	0 (0.0)	0 (0.0)	0 (0.0)	0 (0.0)	1 (25.0)	0 (0.0)	0 (0.0)	0 (0.0)	0 (0.0)
Cough	1 (8.3)	0 (0.0)	0 (0.0)	0 (0.0)	0 (0.0)	0 (0.0)	0 (0.0)	0 (0.0)	0 (0.0)	0 (0.0)
Dyspnea	1 (8.3)	1 (8.3)	0 (0.0)	0 (0.0)	0 (0.0)	0 (0.0)	0 (0.0)	0 (0.0)	0 (0.0)	0 (0.0)
Oropharyngeal pain	0 (0.0)	0 (0.0)	0 (0.0)	0 (0.0)	0 (0.0)	0 (0.0)	0 (0.0)	0 (0.0)	0 (0.0)	0 (0.0)
Dry throat	0 (0.0)	0 (0.0)	0 (0.0)	0 (0.0)	0 (0.0)	0 (0.0)	0 (0.0)	0 (0.0)	0 (0.0)	0 (0.0)
Hiccups	0 (0.0)	0 (0.0)	0 (0.0)	0 (0.0)	0 (0.0)	0 (0.0)	0 (0.0)	0 (0.0)	0 (0.0)	0 (0.0)
Nasal congestion	0 (0.0)	0 (0.0)	0 (0.0)	0 (0.0)	0 (0.0)	0 (0.0)	0 (0.0)	0 (0.0)	0 (0.0)	0 (0.0)
Oropharyngeal discomfort	1 (8.3)	0 (0.0)	0 (0.0)	0 (0.0)	0 (0.0)	0 (0.0)	0 (0.0)	0 (0.0)	0 (0.0)	0 (0.0)
Pleural effusion	0 (0.0)	0 (0.0)	0 (0.0)	0 (0.0)	0 (0.0)	0 (0.0)	0 (0.0)	0 (0.0)	0 (0.0)	0 (0.0)
Pneumonitis	0 (0.0)	0 (0.0)	0 (0.0)	0 (0.0)	0 (0.0)	0 (0.0)	0 (0.0)	0 (0.0)	0 (0.0)	0 (0.0)
Respiratory failure	1 (8.3)	1 (8.3)	1 (8.3)	0 (0.0)	0 (0.0)	0 (0.0)	0 (0.0)	0 (0.0)	0 (0.0)	0 (0.0)
**Musculoskeletal and connective tissue disorders**	4 (33.3)	2 (16.7)	0 (0.0)	0 (0.0)	0 (0.0)	1 (25.0)	0 (0.0)	0 (0.0)	0 (0.0)	0 (0.0)
Back Pain	1 (8.3)	1 (8.3)	0 (0.0)	0 (0.0)	0 (0.0)	1 (25.0)	0 (0.0)	0 (0.0)	0 (0.0)	0 (0.0)
Arthralgia	0 (0.0)	0 (0.0)	0 (0.0)	0 (0.0)	0 (0.0)	0 (0.0)	0 (0.0)	0 (0.0)	0 (0.0)	0 (0.0)
Arthritis	1 (8.3)	0 (0.0)	0 (0.0)	0 (0.0)	0 (0.0)	0 (0.0)	0 (0.0)	0 (0.0)	0 (0.0)	0 (0.0)
Flank pain	1 (8.3)	1 (8.3)	0 (0.0)	0 (0.0)	0 (0.0)	0 (0.0)	0 (0.0)	0 (0.0)	0 (0.0)	0 (0.0)
Joint swelling	1 (8.3)	0 (0.0)	0 (0.0)	0 (0.0)	0 (0.0)	0 (0.0)	0 (0.0)	0 (0.0)	0 (0.0)	0 (0.0)
Limb discomfort	0 (0.0)	0 (0.0)	0 (0.0)	0 (0.0)	0 (0.0)	0 (0.0)	0 (0.0)	0 (0.0)	0 (0.0)	0 (0.0)
Muscle spasms	0 (0.0)	0 (0.0)	0 (0.0)	0 (0.0)	0 (0.0)	1 (25.0)	0 (0.0)	0 (0.0)	0 (0.0)	0 (0.0)
Musculoskeletal pain	1 (8.3)	0 (0.0)	0 (0.0)	0 (0.0)	0 (0.0)	0 (0.0)	0 (0.0)	0 (0.0)	0 (0.0)	0 (0.0)
Neck pain	0 (0.0)	0 (0.0)	0 (0.0)	0 (0.0)	0 (0.0)	0 (0.0)	0 (0.0)	0 (0.0)	0 (0.0)	0 (0.0)
**Skin and subcutaneous tissue disorders**	1 (8.3)	0 (0.0)	0 (0.0)	0 (0.0)	0 (0.0)	1 (25.0)	0 (0.0)	0 (0.0)	0 (0.0)	0 (0.0)
Alopecia	1 (8.3)	0 (0.0)	0 (0.0)	0 (0.0)	0 (0.0)	0 (0.0)	0 (0.0)	0 (0.0)	0 (0.0)	0 (0.0)
Dry skin	0 (0.0)	0 (0.0)	0 (0.0)	0 (0.0)	0 (0.0)	0 (0.0)	0 (0.0)	0 (0.0)	0 (0.0)	0 (0.0)
Pruritus	0 (0.0)	0 (0.0)	0 (0.0)	0 (0.0)	0 (0.0)	0 (0.0)	0 (0.0)	0 (0.0)	0 (0.0)	0 (0.0)
Rash	0 (0.0)	0 (0.0)	0 (0.0)	0 (0.0)	0 (0.0)	0 (0.0)	0 (0.0)	0 (0.0)	0 (0.0)	0 (0.0)
Dermatitis	0 (0.0)	0 (0.0)	0 (0.0)	0 (0.0)	0 (0.0)	0 (0.0)	0 (0.0)	0 (0.0)	0 (0.0)	0 (0.0)

Supplementary Table 1: TEAEs by Worst Grade, SOC and PT - SAF Analysis Set (continued)

**Primary System Organ Class** Preferred Term	**2.8 mg/kg n=12 (100%)**					**3.0 mg/kg n=4 (100%)**				
	**Any Grade n (%)**	**Grade ≥2 n (%)**	**Grade ≥3 n (%)**	**Grade ≥4 n (%)**	**Grade =5 n (%)**	**Any Grade n (%)**	**Grade ≥2 n (%)**	**Grade ≥3 n (%)**	**Grade ≥4 n (%)**	**Grade =5 n (%)**
Eczema	0 (0.0)	0 (0.0)	0 (0.0)	0 (0.0)	0 (0.0)	0 (0.0)	0 (0.0)	0 (0.0)	0 (0.0)	0 (0.0)
Petechiae	0 (0.0)	0 (0.0)	0 (0.0)	0 (0.0)	0 (0.0)	1 (25.0)	0 (0.0)	0 (0.0)	0 (0.0)	0 (0.0)
Photosensitivity reaction	0 (0.0)	0 (0.0)	0 (0.0)	0 (0.0)	0 (0.0)	0 (0.0)	0 (0.0)	0 (0.0)	0 (0.0)	0 (0.0)
**Injury, poisoning and procedural complications**	2 (16.7)	1 (8.3)	1 (8.3)	0 (0.0)	0 (0.0)	0 (0.0)	0 (0.0)	0 (0.0)	0 (0.0)	0 (0.0)
Stoma site hemorrhage	1 (8.3)	0 (0.0)	0 (0.0)	0 (0.0)	0 (0.0)	0 (0.0)	0 (0.0)	0 (0.0)	0 (0.0)	0 (0.0)
Back injury	0 (0.0)	0 (0.0)	0 (0.0)	0 (0.0)	0 (0.0)	0 (0.0)	0 (0.0)	0 (0.0)	0 (0.0)	0 (0.0)
Cervical vertebral fracture	0 (0.0)	0 (0.0)	0 (0.0)	0 (0.0)	0 (0.0)	0 (0.0)	0 (0.0)	0 (0.0)	0 (0.0)	0 (0.0)
Contusion	1 (8.3)	0 (0.0)	0 (0.0)	0 (0.0)	0 (0.0)	0 (0.0)	0 (0.0)	0 (0.0)	0 (0.0)	0 (0.0)
Fracture	1 (8.3)	1 (8.3)	1 (8.3)	0 (0.0)	0 (0.0)	0 (0.0)	0 (0.0)	0 (0.0)	0 (0.0)	0 (0.0)
Infusion related reaction	0 (0.0)	0 (0.0)	0 (0.0)	0 (0.0)	0 (0.0)	0 (0.0)	0 (0.0)	0 (0.0)	0 (0.0)	0 (0.0)
Postoperative wound complication	0 (0.0)	0 (0.0)	0 (0.0)	0 (0.0)	0 (0.0)	0 (0.0)	0 (0.0)	0 (0.0)	0 (0.0)	0 (0.0)
Procedural pain	0 (0.0)	0 (0.0)	0 (0.0)	0 (0.0)	0 (0.0)	0 (0.0)	0 (0.0)	0 (0.0)	0 (0.0)	0 (0.0)
Thermal burn	0 (0.0)	0 (0.0)	0 (0.0)	0 (0.0)	0 (0.0)	0 (0.0)	0 (0.0)	0 (0.0)	0 (0.0)	0 (0.0)
**Renal and urinary disorders**	1 (8.3)	0 (0.0)	0 (0.0)	0 (0.0)	0 (0.0)	1 (25.0)	1 (25.0)	1 (25.0)	0 (0.0)	0 (0.0)
Hematuria	1 (8.3)	0 (0.0)	0 (0.0)	0 (0.0)	0 (0.0)	0 (0.0)	0 (0.0)	0 (0.0)	0 (0.0)	0 (0.0)
Dysuria	0 (0.0)	0 (0.0)	0 (0.0)	0 (0.0)	0 (0.0)	1 (25.0)	0 (0.0)	0 (0.0)	0 (0.0)	0 (0.0)
Acute kidney injury	0 (0.0)	0 (0.0)	0 (0.0)	0 (0.0)	0 (0.0)	1 (25.0)	1 (25.0)	1 (25.0)	0 (0.0)	0 (0.0)
Pollakiuria	0 (0.0)	0 (0.0)	0 (0.0)	0 (0.0)	0 (0.0)	0 (0.0)	0 (0.0)	0 (0.0)	0 (0.0)	0 (0.0)
Proteinuria	0 (0.0)	0 (0.0)	0 (0.0)	0 (0.0)	0 (0.0)	0 (0.0)	0 (0.0)	0 (0.0)	0 (0.0)	0 (0.0)
**Neoplasms benign, malignant an unspecified (incl cysts and polyps)**	0 (0.0)	0 (0.0)	0 (0.0)	0 (0.0)	0 (0.0)	0 (0.0)	0 (0.0)	0 (0.0)	0 (0.0)	0 (0.0)
Tumor pain	0 (0.0)	0 (0.0)	0 (0.0)	0 (0.0)	0 (0.0)	0 (0.0)	0 (0.0)	0 (0.0)	0 (0.0)	0 (0.0)
Cancer pain	0 (0.0)	0 (0.0)	0 (0.0)	0 (0.0)	0 (0.0)	0 (0.0)	0 (0.0)	0 (0.0)	0 (0.0)	0 (0.0)
Colorectal adenoma	0 (0.0)	0 (0.0)	0 (0.0)	0 (0.0)	0 (0.0)	0 (0.0)	0 (0.0)	0 (0.0)	0 (0.0)	0 (0.0)
**Vascular disorders**	2 (16.7)	1 (8.3)	1 (8.3)	1 (8.3)	1 (8.3)	0 (0.0)	0 (0.0)	0 (0.0)	0 (0.0)	0 (0.0)
Hypotension	1 (8.3)	0 (0.0)	0 (0.0)	0 (0.0)	0 (0.0)	0 (0.0)	0 (0.0)	0 (0.0)	0 (0.0)	0 (0.0)
Hypertension	0 (0.0)	0 (0.0)	0 (0.0)	0 (0.0)	0 (0.0)	0 (0.0)	0 (0.0)	0 (0.0)	0 (0.0)	0 (0.0)

Supplementary Table 1: TEAEs by Worst Grade, SOC and PT - SAF Analysis Set (continued)

**Primary System Organ Class**<br>Preferred Term	**2.8 mg/kg**<br>**n=12 (100%)**					**3.0 mg/kg**<br>**n=4 (100%)**				
	**Any Grade n (%)**	**Grade ≥2 n (%)**	**Grade ≥3 n (%)**	**Grade ≥4 n (%)**	**Grade =5 n (%)**	**Any Grade n (%)**	**Grade ≥2 n (%)**	**Grade ≥3 n (%)**	**Grade ≥4 n (%)**	**Grade =5 n (%)**
Hypovolemic shock	1 (8.3)	1 (8.3)	1 (8.3)	1 (8.3)	1 (8.3)	0 (0.0)	0 (0.0)	0 (0.0)	0 (0.0)	0 (0.0)
**Psychiatric disorders**	1 (8.3)	0 (0.0)	0 (0.0)	0 (0.0)	0 (0.0)	0 (0.0)	0 (0.0)	0 (0.0)	0 (0.0)	0 (0.0)
Insomnia	0 (0.0)	0 (0.0)	0 (0.0)	0 (0.0)	0 (0.0)	0 (0.0)	0 (0.0)	0 (0.0)	0 (0.0)	0 (0.0)
Nervousness	1 (8.3)	0 (0.0)	0 (0.0)	0 (0.0)	0 (0.0)	0 (0.0)	0 (0.0)	0 (0.0)	0 (0.0)	0 (0.0)
Sleep disorder	0 (0.0)	0 (0.0)	0 (0.0)	0 (0.0)	0 (0.0)	0 (0.0)	0 (0.0)	0 (0.0)	0 (0.0)	0 (0.0)
**Reproductive system and breast disorders**	1 (8.3)	0 (0.0)	0 (0.0)	0 (0.0)	0 (0.0)	0 (0.0)	0 (0.0)	0 (0.0)	0 (0.0)	0 (0.0)
Intermenstrual bleeding	0 (0.0)	0 (0.0)	0 (0.0)	0 (0.0)	0 (0.0)	0 (0.0)	0 (0.0)	0 (0.0)	0 (0.0)	0 (0.0)
Pelvic pain	0 (0.0)	0 (0.0)	0 (0.0)	0 (0.0)	0 (0.0)	0 (0.0)	0 (0.0)	0 (0.0)	0 (0.0)	0 (0.0)
Perineal pain	1 (8.3)	0 (0.0)	0 (0.0)	0 (0.0)	0 (0.0)	0 (0.0)	0 (0.0)	0 (0.0)	0 (0.0)	0 (0.0)
**Cardiac disorders**	0 (0.0)	0 (0.0)	0 (0.0)	0 (0.0)	0 (0.0)	0 (0.0)	0 (0.0)	0 (0.0)	0 (0.0)	0 (0.0)
Palpitations	0 (0.0)	0 (0.0)	0 (0.0)	0 (0.0)	0 (0.0)	0 (0.0)	0 (0.0)	0 (0.0)	0 (0.0)	0 (0.0)
**Ear and labyrinth disorders**	0 (0.0)	0 (0.0)	0 (0.0)	0 (0.0)	0 (0.0)	0 (0.0)	0 (0.0)	0 (0.0)	0 (0.0)	0 (0.0)
Ear pain	0 (0.0)	0 (0.0)	0 (0.0)	0 (0.0)	0 (0.0)	0 (0.0)	0 (0.0)	0 (0.0)	0 (0.0)	0 (0.0)
**Endocrine disorders**	0 (0.0)	0 (0.0)	0 (0.0)	0 (0.0)	0 (0.0)	0 (0.0)	0 (0.0)	0 (0.0)	0 (0.0)	0 (0.0)
Hyperthyroidism	0 (0.0)	0 (0.0)	0 (0.0)	0 (0.0)	0 (0.0)	0 (0.0)	0 (0.0)	0 (0.0)	0 (0.0)	0 (0.0)
**Hepatobiliary disorders**	0 (0.0)	0 (0.0)	0 (0.0)	0 (0.0)	0 (0.0)	0 (0.0)	0 (0.0)	0 (0.0)	0 (0.0)	0 (0.0)
Cholangitis	0 (0.0)	0 (0.0)	0 (0.0)	0 (0.0)	0 (0.0)	0 (0.0)	0 (0.0)	0 (0.0)	0 (0.0)	0 (0.0)

Supplementary Table 1: TEAEs by Worst Grade, SOC and PT - SAF Analysis Set (continued)

**Primary System Organ Class**<br>Preferred Term	**3.2 mg/kg**<br>**n=7 (100%)**					**Total**<br>**N=40 (100%)**				
	**Any Grade n (%)**	**Grade ≥2 n (%)**	**Grade ≥3 n (%)**	**Grade ≥4 n (%)**	**Grade =5 n (%)**	**Any Grade n (%)**	**Grade ≥2 n (%)**	**Grade ≥3 n (%)**	**Grade ≥4 n (%)**	**Grade =5 n (%)**
**Subjects with at least one Event**	7 (100)	7 (100)	7(100)	6 (85.7)	0 (0.0)	38 (95.0)	35 (87.5)	28 (70.0)	17 (42.5)	2 (5.0)
**Gastrointestinal disorders**	6 (85.7)	3 (42.9)	0 (0.0)	0 (0.0)	0 (0.0)	35 (87.5)	16 (40.0)	2 (5.0)	1 (2.5)	1 (2.5)
Nausea	3 (42.9)	2 (28.6)	0 (0.0)	0 (0.0)	0 (0.0)	20 (50.0)	10 (25.0)	0 (0.0)	0 (0.0)	0 (0.0)
Vomiting	2 (28.6)	1 (14.3)	0 (0.0)	0 (0.0)	0 (0.0)	12 (30.0)	2 (5.0)	0 (0.0)	0 (0.0)	0 (0.0)
Diarrhea	1 (14.3)	0 (0.0)	0 (0.0)	0 (0.0)	0 (0.0)	11 (27.5)	2 (5.0)	0 (0.0)	0 (0.0)	0 (0.0)
Constipation	1 (14.3)	0 (0.0)	0 (0.0)	0 (0.0)	0 (0.0)	8 (20.0)	2 (5.0)	0 (0.0)	0 (0.0)	0 (0.0)
Stomatitis	3 (42.9)	1 (14.3)	0 (0.0)	0 (0.0)	0 (0.0)	5 (12.5)	2 (5.0)	0 (0.0)	0 (0.0)	0 (0.0)
Abdominal pain	1 (14.3)	0 (0.0)	0 (0.0)	0 (0.0)	0 (0.0)	4 (10.0)	2 (5.0)	0 (0.0)	0 (0.0)	0 (0.0)
Abdominal pain upper	1 (14.3)	0 (0.0)	0 (0.0)	0 (0.0)	0 (0.0)	2 (5.0)	0 (0.0)	0 (0.0)	0 (0.0)	0 (0.0)
Rectal hemorrhage	0 (0.0)	0 (0.0)	0 (0.0)	0 (0.0)	0 (0.0)	2 (5.0)	0 (0.0)	0 (0.0)	0 (0.0)	0 (0.0)
Abdominal distension	1 (14.3)	0 (0.0)	0 (0.0)	0 (0.0)	0 (0.0)	1 (2.5)	0 (0.0)	0 (0.0)	0 (0.0)	0 (0.0)
Anal hemorrhage	1 (14.3)	0 (0.0)	0 (0.0)	0 (0.0)	0 (0.0)	1 (2.5)	0 (0.0)	0 (0.0)	0 (0.0)	0 (0.0)
Ascites	0 (0.0)	0 (0.0)	0 (0.0)	0 (0.0)	0 (0.0)	1 (2.5)	1 (2.5)	0 (0.0)	0 (0.0)	0 (0.0)
Flatulence	0 (0.0)	0 (0.0)	0 (0.0)	0 (0.0)	0 (0.0)	1 (2.5)	0 (0.0)	0 (0.0)	0 (0.0)	0 (0.0)
Gastrointestinal hemorrhage	0 (0.0)	0 (0.0)	0 (0.0)	0 (0.0)	0 (0.0)	1 (2.5)	1 (2.5)	1 (2.5)	1 (2.5)	1 (2.5)
Hematochezia	0 (0.0)	0 (0.0)	0 (0.0)	0 (0.0)	0 (0.0)	1 (2.5)	0 (0.0)	0 (0.0)	0 (0.0)	0 (0.0)
Hemorrhoidal hemorrhage	0 (0.0)	0 (0.0)	0 (0.0)	0 (0.0)	0 (0.0)	1 (2.5)	0 (0.0)	0 (0.0)	0 (0.0)	0 (0.0)
Ileus	0 (0.0)	0 (0.0)	0 (0.0)	0 (0.0)	0 (0.0)	1 (2.5)	1 (2.5)	0 (0.0)	0 (0.0)	0 (0.0)
Intestinal obstruction	1 (14.3)	1(14.3)	0 (0.0)	0 (0.0)	0 (0.0)	1 (2.5)	1 (2.5)	0 (0.0)	0 (0.0)	0 (0.0)
Upper gastrointestinal hemorrhage	0 (0.0)	0 (0.0)	0 (0.0)	0 (0.0)	0 (0.0)	1 (2.5)	1 (2.5)	1 (2.5)	0 (0.0)	0 (0.0)
**Blood and lymphatic system disorders**	6 (85.7)	6 (85.7)	6 (85.7)	2 (28.6)	0 (0.0)	29 (72.5)	26 (65.0)	20 (50.0)	4 (10.0)	0 (0.0)
Anemia	6 (85.7)	6 (85.7)	6 (85.7)	0 (0.0)	0 (0.0)	26 (65.0)	23 (57.5)	16 (40.0)	0 (0.0)	0 (0.0)
Neutropenia	1 (14.3)	1 (14.3)	1 (14.3)	1 (14.3)	0 (0.0)	7 (17.5)	7 (17.5)	6 (15.0)	3 (7.5)	0 (0.0)
Febrile neutropenia	2 (28.6)	2 (28.6)	2 (28.6)	0 (0.0)	0 (0.0)	3 (7.5)	3 (7.5)	3 (7.5)	0 (0.0)	0 (0.0)
Thrombocytopenia	2 (28.6)	2 (28.6)	2 (28.6)	1 (14.3)	0 (0.0)	2 (5.0)	2 (5.0)	2 (5.0)	1 (2.5)	0 (0.0)
**General disorder and administration site condition**	6 (85.7)	4 (57.1)	1 (14.3)	0 (0.0)	0 (0.0)	29 (72.5)	12 (30.0)	2 (5.0)	0 (0.0)	0 (0.0)
Fatigue	2 (28.6)	2 (28.6)	0 (0.0)	0 (0.0)	0 (0.0)	20 (50.0)	8 (20.0)	0 (0.0)	0 (0.0)	0 (0.0)
Asthenia	0 (0.0)	0 (0.0)	0 (0.0)	0 (0.0)	0 (0.0)	4 (10.0)	2 (5.0)	1 (2.5)	0 (0.0)	0 (0.0)

Supplementary Table 1: TEAEs by Worst Grade, SOC and PT - SAF Analysis Set (continued)

**Primary System Organ Class**<br>Preferred Term	**3.2 mg/kg**<br>**n=7 (100%)**					**Total**<br>**N=40 (100%)**				
	**Any Grade n (%)**	**Grade ≥2 n (%)**	**Grade ≥3 n (%)**	**Grade ≥4 n (%)**	**Grade =5 n (%)**	**Any Grade n (%)**	**Grade ≥2 n (%)**	**Grade ≥3 n (%)**	**Grade ≥4 n (%)**	**Grade =5 n (%)**
Pyrexia	2 (28.6)	1 (14.3)	1 (14.3)	0 (0.0)	0 (0.0)	4 (10.0)	1 (2.5)	1 (2.5)	0 (0.0)	0 (0.0)
Oedema peripheral	0 (0.0)	0 (0.0)	0 (0.0)	0 (0.0)	0 (0.0)	2 (5.0)	0 (0.0)	0 (0.0)	0 (0.0)	0 (0.0)
Malaise	1 (14.3)	0 (0.0)	0 (0.0)	0 (0.0)	0 (0.0)	1 (2.5)	0 (0.0)	0 (0.0)	0 (0.0)	0 (0.0)
Medical device site fistula	1 (14.3)	1 (14.3)	0 (0.0)	0 (0.0)	0 (0.0)	1 (2.5)	1 (2.5)	0 (0.0)	0 (0.0)	0 (0.0)
Pain	0 (0.0)	0 (0.0)	0 (0.0)	0 (0.0)	0 (0.0)	1 (2.5)	0 (0.0)	0 (0.0)	0 (0.0)	0 (0.0)
Peripheral swelling	0 (0.0)	0 (0.0)	0 (0.0)	0 (0.0)	0 (0.0)	1 (2.5)	0 (0.0)	0 (0.0)	0 (0.0)	0 (0.0)
**Investigations**	6 (85.7)	6 (85.7)	6 (85.7)	5 (71.4)	0 (0.0)	28 (70.0)	25 (62.5)	20 (50.0)	15 (37.5)	0 (0.0)
Neutrophil count decreased	5 (71.4)	5 (71.4)	5 (71.4)	3 (42.9)	0 (0.0)	17 (42.5)	15 (37.5)	14 (35.0)	10 (25.0)	0 (0.0)
White blood cell count decreased	6 (85.7)	6 (85.7)	5 (71.4)	1 (14.3)	0 (0.0)	17 (42.5)	17 (42.5)	12 (30.0)	7 (17.5)	0 (0.0)
Platelet count decreased	5 (71.4)	4 (57.1)	3 (42.9)	2 (28.6)	0 (0.0)	16 (40.0)	12 (30.0)	11 (27.5)	7 (17.5)	0 (0.0)
Lymphocyte count decreased	3 (42.9)	3 (42.9)	3 (42.9)	3 (42.9)	0 (0.0)	10 (25.0)	10 (25.0)	9 (22.5)	2 (5.0)	0 (0.0)
Alanine aminotransferase increased	3 (42.9)	0 (0.0)	0 (0.0)	1 (14.3)	0 (0.0)	6 (15.0)	0 (0.0)	0 (0.0)	0 (0.0)	0 (0.0)
Lipase increased	2 (28.6)	1 (14.3)	0 (0.0)	0 (0.0)	0 (0.0)	6 (15.0)	3 (7.5)	0 (0.0)	0 (0.0)	0 (0.0)
Weight decreased	1 (14.3)	1 (14.3)	0 (0.0)	0 (0.0)	0 (0.0)	6 (15.0)	4 (10.0)	0 (0.0)	0 (0.0)	0 (0.0)
Aspartate aminotransferase increased	2 (28.6)	1 (14.3)	1 (14.3)	0 (0.0)	0 (0.0)	5 (12.5)	1 (2.5)	1 (2.5)	0 (0.0)	0 (0.0)
Gamma-glutamyltransferase increased	0 (0.0)	0 (0.0)	0 (0.0)	0 (0.0)	0 (0.0)	3 (7.5)	1 (2.5)	0 (0.0)	0 (0.0)	0 (0.0)
Amylase increased	1 (14.3)	0 (0.0)	0 (0.0)	0 (0.0)	0 (0.0)	2 (5.0)	0 (0.0)	0 (0.0)	0 (0.0)	0 (0.0)
Blood alkaline phosphatase increased	1 (14.3)	0 (0.0)	0 (0.0)	0 (0.0)	0 (0.0)	2 (5.0)	0 (0.0)	0 (0.0)	0 (0.0)	0 (0.0)
Blood bilirubin increased	1 (14.3)	1 (14.3)	0 (0.0)	0 (0.0)	0 (0.0)	2 (5.0)	1 (2.5)	0 (0.0)	0 (0.0)	0 (0.0)
Blood creatine increased	1 (14.3)	0 (0.0)	0 (0.0)	0 (0.0)	0 (0.0)	2 (5.0)	0 (0.0)	0 (0.0)	0 (0.0)	0 (0.0)
Blood creatinine increased	1 (14.3)	0 (0.0)	0 (0.0)	0 (0.0)	0 (0.0)	2 (5.0)	1 (2.5)	1 (2.5)	0 (0.0)	0 (0.0)
Blood creatinine decreased	0 (0.0)	0 (0.0)	0 (0.0)	0 (0.0)	0 (0.0)	1 (2.5)	0 (0.0)	0 (0.0)	0 (0.0)	0 (0.0)
**Metabolism and nutrition disorders**	5 (71.4)	2 (28.6)	1 (14.3)	0 (0.0)	0 (0.0)	22 (55.0)	11 (27.5)	2 (5.0)	0 (0.0)	0 (0.0)
Decreased appetite	2 (28.6)	0 (0.0)	0 (0.0)	0 (0.0)	0 (0.0)	11 (27.5)	3 (7.5)	0 (0.0)	0 (0.0)	0 (0.0)
Dehydration	1 (14.3)	1 (14.3)	0 (0.0)	0 (0.0)	0 (0.0)	6 (15.0)	6 (15.0)	0 (0.0)	0 (0.0)	0 (0.0)
Hypokalemia	1 (14.3)	1 (14.3)	1 (14.3)	0 (0.0)	0 (0.0)	5 (12.5)	2 (5.0)	1 (2.5)	0 (0.0)	0 (0.0)
Hypoalbuminemia	1 (14.3)	1 (14.3)	0 (0.0)	0 (0.0)	0 (0.0)	3 (7.5)	2 (5.0)	0 (0.0)	0 (0.0)	0 (0.0)
Hyponatremia	0 (0.0)	0 (0.0)	0 (0.0)	0 (0.0)	0 (0.0)	3 (7.5)	1 (2.5)	1 (2.5)	0 (0.0)	0 (0.0)
Hyperphosphatemia	0 (0.0)	0 (0.0)	0 (0.0)	0 (0.0)	0 (0.0)	1 (2.5)	0 (0.0)	0 (0.0)	0 (0.0)	0 (0.0)
Hypocalcemia	0 (0.0)	0 (0.0)	0 (0.0)	0 (0.0)	0 (0.0)	1 (2.5)	1 (2.5)	0 (0.0)	0 (0.0)	0 (0.0)

Supplementary Table 1: TEAEs by Worst Grade, SOC and PT - SAF Analysis Set (continued)

**Primary System Organ Class**<br>Preferred Term	**3.2 mg/kg**<br>**n=7 (100%)**					**Total**<br>**N=40 (100%)**				
	**Any Grade n (%)**	**Grade ≥2 n (%)**	**Grade ≥3 n (%)**	**Grade ≥4 n (%)**	**Grade =5 n (%)**	**Any Grade n (%)**	**Grade ≥2 n (%)**	**Grade ≥3 n (%)**	**Grade ≥4 n (%)**	**Grade =5 n (%)**
Hypomagnesaemia	0 (0.0)	0 (0.0)	0 (0.0)	0 (0.0)	0 (0.0)	1 (2.5)	0 (0.0)	0 (0.0)	0 (0.0)	0 (0.0)
Hypophosphatemia	0 (0.0)	0 (0.0)	0 (0.0)	0 (0.0)	0 (0.0)	1 (2.5)	1 (2.5)	0 (0.0)	0 (0.0)	0 (0.0)
Vitamin D deficiency	1 (14.3)	0 (0.0)	0 (0.0)	0 (0.0)	0 (0.0)	1 (2.5)	0 (0.0)	0 (0.0)	0 (0.0)	0 (0.0)
**Infections and infestations**	2 (28.6)	2 (28.6)	1 (14.3)	0 (0.0)	0 (0.0)	13 (32.5)	11 (27.5)	5 (12.5)	1 (2.5)	1 (2.5)
COVID-19	0 (0.0)	0 (0.0)	0 (0.0)	0 (0.0)	0 (0.0)	3 (7.5)	3 (7.5)	1 (2.5)	0 (0.0)	0 (0.0)
Upper respiratory tract infection	1 (14.3)	1 (14.3)	0 (0.0)	0 (0.0)	0 (0.0)	2 (5.0)	2 (5.0)	0 (0.0)	0 (0.0)	0 (0.0)
Herpes zoster	0 (0.0)	0 (0.0)	0 (0.0)	0 (0.0)	0 (0.0)	1 (2.5)	1 (2.5)	0 (0.0)	0 (0.0)	0 (0.0)
Liver abscess	1 (14.3)	1 (14.3)	1 (14.3)	0 (0.0)	0 (0.0)	1 (2.5)	1 (2.5)	1 (2.5)	0 (0.0)	0 (0.0)
Metapneumovirus infection	0 (0.0)	0 (0.0)	0 (0.0)	0 (0.0)	0 (0.0)	1 (2.5)	0 (0.0)	0 (0.0)	0 (0.0)	0 (0.0)
Respiratory tract infection	0 (0.0)	0 (0.0)	0 (0.0)	0 (0.0)	0 (0.0)	1 (2.5)	0 (0.0)	0 (0.0)	0 (0.0)	0 (0.0)
Rhinitis	0 (0.0)	0 (0.0)	0 (0.0)	0 (0.0)	0 (0.0)	1 (2.5)	0 (0.0)	0 (0.0)	0 (0.0)	0 (0.0)
Sepsis	0 (0.0)	0 (0.0)	0 (0.0	0 (0.0)	0 (0.0)	1 (2.5)	1 (2.5)	1 (2.5)	1 (2.5)	1 (2.5)
Septic shock	1 (14.3)	1 (14.3)	1 (14.3)	0 (0.0)	0 (0.0)	1 (2.5)	1 (2.5)	1 (2.5)	0 (0.0)	0 (0.0)
Skin infection	0 (0.0)	0 (0.0)	0 (0.0)	0 (0.0)	0 (0.0)	1 (2.5)	1 (2.5)	0 (0.0)	0 (0.0)	0 (0.0)
Systemic candida	0 (0.0)	0 (0.0)	0 (0.0)	0 (0.0)	0 (0.0)	1 (2.5)	1 (2.5)	1 (2.5)	0 (0.0)	0 (0.0)
Tooth infection	0 (0.0)	0 (0.0)	0 (0.0)	0 (0.0)	0 (0.0)	1 (2.5)	1 (2.5)	0 (0.0)	0 (0.0)	0 (0.0)
Vascular device infection	0 (0.0)	0 (0.0)	0 (0.0)	0 (0.0)	0 (0.0)	1 (2.5)	1 (2.5)	1 (2.5)	0 (0.0)	0 (0.0)
**Nervous system disorders**	2 (28.6)	0 (0.0)	0 (0.0)	0 (0.0)	0 (0.0)	13 (32.5)	3 (7.5)	0 (0.0)	0 (0.0)	0 (0.0)
Headache	2 (28.6)	0 (0.0)	0 (0.0)	0 (0.0)	0 (0.0)	4 (10.0)	0 (0.0)	0 (0.0)	0 (0.0)	0 (0.0)
Dizziness	0 (0.0)	0 (0.0)	0 (0.0)	0 (0.0)	0 (0.0)	2 (5.0)	0 (0.0)	0 (0.0)	0 (0.0)	0 (0.0)
Dysgeusia	0 (0.0)	0 (0.0)	0 (0.0)	0 (0.0)	0 (0.0)	2 (5.0)	0 (0.0)	0 (0.0)	0 (0.0)	0 (0.0)
Cerebrovascular accident	0 (0.0)	0 (0.0)	0 (0.0)	0 (0.0)	0 (0.0)	1 (2.5)	1 (2.5)	0 (0.0)	0 (0.0)	0 (0.0)
Cervical radiculopathy	0 (0.0)	0 (0.0)	0 (0.0)	0 (0.0)	0 (0.0)	1 (2.5)	1 (2.5)	0 (0.0)	0 (0.0)	0 (0.0)
Disturbance in attention	0 (0.0)	0 (0.0)	0 (0.0)	0 (0.0)	0 (0.0)	1 (2.5)	0 (0.0)	0 (0.0)	0 (0.0)	0 (0.0)
Hypoesthesia	1 (14.3)	0 (0.0)	0 (0.0)	0 (0.0)	0 (0.0)	1 (2.5)	0 (0.0)	0 (0.0)	0 (0.0)	0 (0.0)
Paresthesia	0 (0.0)	0 (0.0)	0 (0.0)	0 (0.0)	0 (0.0)	1 (2.5)	0 (0.0)	0 (0.0)	0 (0.0)	0 (0.0)
Peripheral sensory neuropathy	0 (0.0)	0 (0.0)	0 (0.0)	0 (0.0)	0 (0.0)	1 (2.5)	1 (2.5)	0 (0.0)	0 (0.0)	0 (0.0)
Seizure	1 (14.3)	0 (0.0)	0 (0.0)	0 (0.0)	0 (0.0)	1 (2.5)	0 (0.0)	0 (0.0)	0 (0.0)	0 (0.0)
Syncope	0 (0.0)	0 (0.0)	0 (0.0)	0 (0.0)	0 (0.0)	1 (2.5)	0 (0.0)	0 (0.0)	0 (0.0)	0 (0.0)

Supplementary Table 1: TEAEs by Worst Grade, SOC and PT - SAF Analysis Set (continued)

**Primary System Organ Class** Preferred Term	**3.2 mg/kg n=7 (100%)**					**Total N=40 (100%)**				
	**Any Grade n (%)**	**Grade ≥2 n (%)**	**Grade ≥3 n (%)**	**Grade ≥4 n (%)**	**Grade =5 n (%)**	**Any Grade n (%)**	**Grade ≥2 n (%)**	**Grade ≥3 n (%)**	**Grade ≥4 n (%)**	**Grade =5 n (%)**
**Respiratory, thoracic and mediastina disorders**	4 (57.1)	0 (0.0)	0 (0.0)	0 (0.0)	0 (0.0)	13 (32.5)	3 (7.5)	1 (2.5)	0 (0.0)	0 (0.0)
Epistaxis	1 (14.3)	0 (0.0)	0 (0.0)	0 (0.0)	0 (0.0)	6 (15.0)	0 (0.0)	0 (0.0)	0 (0.0)	0 (0.0)
Cough	1 (14.3)	0 (0.0)	0 (0.0)	0 (0.0)	0 (0.0)	5 (12.5)	1 (2.5)	0 (0.0)	0 (0.0)	0 (0.0)
Dyspnea	1 (14.3)	0 (0.0)	0 (0.0)	0 (0.0)	0 (0.0)	4 (10.0)	2 (5.0)	0 (0.0)	0 (0.0)	0 (0.0)
Oropharyngeal pain	2 (28.6)	0 (0.0)	0 (0.0)	0 (0.0)	0 (0.0)	2 (5.0)	0 (0.0)	0 (0.0)	0 (0.0)	0 (0.0)
Dry throat	1 (14.3)	0 (0.0)	0 (0.0)	0 (0.0)	0 (0.0)	1 (2.5)	0 (0.0)	0 (0.0)	0 (0.0)	0 (0.0)
Hiccups	1 (14.3)	0 (0.0)	0 (0.0)	0 (0.0)	0 (0.0)	1 (2.5)	0 (0.0)	0 (0.0)	0 (0.0)	0 (0.0)
Nasal congestion	0 (0.0)	0 (0.0)	0 (0.0)	0 (0.0)	0 (0.0)	1 (2.5)	0 (0.0)	0 (0.0)	0 (0.0)	0 (0.0)
Oropharyngeal discomfort	0 (0.0)	0 (0.0)	0 (0.0)	0 (0.0)	0 (0.0)	1 (2.5)	0 (0.0)	0 (0.0)	0 (0.0)	0 (0.0)
Pleural effusion	0 (0.0)	0 (0.0)	0 (0.0)	0 (0.0)	0 (0.0)	1 (2.5)	1 (2.5)	0 (0.0)	0 (0.0)	0 (0.0)
Pneumonitis	0 (0.0)	0 (0.0)	0 (0.0)	0 (0.0)	0 (0.0)	1 (2.5)	0 (0.0)	0 (0.0)	0 (0.0)	0 (0.0)
Respiratory failure	0 (0.0)	0 (0.0)	0 (0.0)	0 (0.0)	0 (0.0)	1 (2.5)	1 (2.5)	1 (2.5)	0 (0.0)	0 (0.0)
**Musculoskeletal and connective tissue disorders**	3 (42.9)	0 (0.0)	0 (0.0)	0 (0.0)	0 (0.0)	12 (30.0)	2 (5.0)	0 (0.0)	0 (0.0)	0 (0.0)
Back Pain	2 (28.6)	0 (0.0)	0 (0.0)	0 (0.0)	0 (0.0)	6 (15.0)	1 (2.5)	0 (0.0)	0 (0.0)	0 (0.0)
Arthralgia	1 (14.3)	0 (0.0)	0 (0.0)	0 (0.0)	0 (0.0)	1 (2.5)	0 (0.0)	0 (0.0)	0 (0.0)	0 (0.0)
Arthritis	0 (0.0)	0 (0.0)	0 (0.0)	0 (0.0)	0 (0.0)	1 (2.5)	0 (0.0)	0 (0.0)	0 (0.0)	0 (0.0)
Flank pain	0 (0.0)	0 (0.0)	0 (0.0)	0 (0.0)	0 (0.0)	1 (2.5)	1 (2.5)	0 (0.0)	0 (0.0)	0 (0.0)
Joint swelling	0 (0.0)	0 (0.0)	0 (0.0)	0 (0.0)	0 (0.0)	1 (2.5)	0 (0.0)	0 (0.0)	0 (0.0)	0 (0.0)
Limb discomfort	0 (0.0)	0 (0.0)	0 (0.0)	0 (0.0)	0 (0.0)	1 (2.5)	0 (0.0)	0 (0.0)	0 (0.0)	0 (0.0)
Muscle spasms	0 (0.0)	0 (0.0)	0 (0.0)	0 (0.0)	0 (0.0)	1 (2.5)	0 (0.0)	0 (0.0)	0 (0.0)	0 (0.0)
Musculoskeletal pain	0 (0.0)	0 (0.0)	0 (0.0)	0 (0.0)	0 (0.0)	1 (2.5)	0 (0.0)	0 (0.0)	0 (0.0)	0 (0.0)
Neck pain	0 (0.0)	0 (0.0)	0 (0.0)	0 (0.0)	0 (0.0)	1 (2.5)	0 (0.0)	0 (0.0)	0 (0.0)	0 (0.0)
**Skin and subcutaneous tissue disorders**	3 (42.9)	0 (0.0)	0 (0.0)	0 (0.0)	0 (0.0)	10 (25.0)	0 (0.0)	0 (0.0)	0 (0.0)	0 (0.0)
Alopecia	1 (14.3)	0 (0.0)	0 (0.0)	0 (0.0)	0 (0.0)	5 (12.5)	0 (0.0)	0 (0.0)	0 (0.0)	0 (0.0)
Dry skin	1 (14.3)	0 (0.0)	0 (0.0)	0 (0.0)	0 (0.0)	2 (5.0)	0 (0.0)	0 (0.0)	0 (0.0)	0 (0.0)
Pruritus	0 (0.0)	0 (0.0)	0 (0.0)	0 (0.0)	0 (0.0)	2 (5.0)	0 (0.0)	0 (0.0)	0 (0.0)	0 (0.0)
Rash	1 (14.3)	0 (0.0)	0 (0.0)	0 (0.0)	0 (0.0)	2 (5.0)	0 (0.0)	0 (0.0)	0 (0.0)	0 (0.0)
Dermatitis	1 (14.3)	0 (0.0)	0 (0.0)	0 (0.0)	0 (0.0)	1 (2.5)	0 (0.0)	0 (0.0)	0 (0.0)	0 (0.0)

Supplementary Table 1: TEAEs by Worst Grade, SOC and PT - SAF Analysis Set (continued)

**Primary System Organ Class** Preferred Term	**3.2 mg/kg n=7 (100%)**					**Total n=40 (100%)**				
	**Any Grade n (%)**	**Grade ≥2 n (%)**	**Grade ≥3 n (%)**	**Grade ≥4 n (%)**	**Grade =5 n (%)**	**Any Grade n (%)**	**Grade ≥2 n (%)**	**Grade ≥3 n (%)**	**Grade ≥4 n (%)**	**Grade =5 n (%)**
Eczema	1 (14.3)	0 (0.0)	0 (0.0)	0 (0.0)	0 (0.0)	1 (2.5)	0 (0.0)	0 (0.0)	0 (0.0)	0 (0.0)
Petechiae	0 (0.0)	0 (0.0)	0 (0.0)	0 (0.0)	0 (0.0)	1 (2.5)	0 (0.0)	0 (0.0)	0 (0.0)	0 (0.0)
Photosensitivity reaction	0 (0.0)	0 (0.0)	0 (0.0)	0 (0.0)	0 (0.0)	1 (2.5)	0 (0.0)	0 (0.0)	0 (0.0)	0 (0.0)
**Injury, poisoning and procedural complications**	2 (28.6)	0 (0.0)	0 (0.0)	0 (0.0)	0 (0.0)	9 (22.5)	3 (7.5)	1 (2.5)	0 (0.0)	0 (0.0)
Stoma site hemorrhage	0 (0.0)	0 (0.0)	0 (0.0)	0 (0.0)	0 (0.0)	2 (5.0)	0 (0.0)	0 (0.0)	0 (0.0)	0 (0.0)
Back injury	0 (0.0)	0 (0.0)	0 (0.0)	0 (0.0)	0 (0.0)	1 (2.5)	0 (0.0)	0 (0.0)	0 (0.0)	0 (0.0)
Cervical vertebral fracture	0 (0.0)	0 (0.0)	0 (0.0)	0 (0.0)	0 (0.0)	1 (2.5)	1 (2.5)	0 (0.0)	0 (0.0)	0 (0.0)
Contusion	0 (0.0)	0 (0.0)	0 (0.0)	0 (0.0)	0 (0.0)	1 (2.5)	0 (0.0)	0 (0.0)	0 (0.0)	0 (0.0)
Fracture	0 (0.0)	0 (0.0)	0 (0.0)	0 (0.0)	0 (0.0)	1 (2.5)	1 (2.5)	1 (2.5)	0 (0.0)	0 (0.0)
Infusion related reaction	0 (0.0)	0 (0.0)	0 (0.0)	0 (0.0)	0 (0.0)	1 (2.5)	1 (2.5)	0 (0.0)	0 (0.0)	0 (0.0)
Postoperative wound complication	1 (14.3)	0 (0.0)	0 (0.0)	0 (0.0)	0 (0.0)	1 (2.5)	0 (0.0)	0 (0.0)	0 (0.0)	0 (0.0)
Procedural pain	1 (14.3)	0 (0.0)	0 (0.0)	0 (0.0)	0 (0.0)	1 (2.5)	0 (0.0)	0 (0.0)	0 (0.0)	0 (0.0)
Thermal burn	0 (0.0)	0 (0.0)	0 (0.0)	0 (0.0)	0 (0.0)	1 (2.5)	0 (0.0)	0 (0.0)	0 (0.0)	0 (0.0)
**Renal and urinary disorders**	3 (42.9)	1 (14.3)	0 (0.0)	0 (0.0)	0 (0.0)	8 (20.0)	3 (7.5)	2 (5.0)	0 (0.0)	0 (0.0)
Hematuria	1 (14.3)	0 (0.0)	0 (0.0)	0 (0.0)	0 (0.0)	4 (10.0)	1 (2.5)	1 (2.5)	0 (0.0)	0 (0.0)
Dysuria	1 (14.3)	0 (0.0)	0 (0.0)	0 (0.0)	0 (0.0)	3 (7.5)	0 (0.0)	0 (0.0)	0 (0.0)	0 (0.0)
Acute kidney injury	0 (0.0)	0 (0.0)	0 (0.0)	0 (0.0)	0 (0.0)	1 (2.5)	1 (2.5)	1 (2.5)	0 (0.0)	0 (0.0)
Pollakiuria	0 (0.0)	0 (0.0)	0 (0.0)	0 (0.0)	0 (0.0)	1 (2.5)	0 (0.0)	0 (0.0)	0 (0.0)	0 (0.0)
Proteinuria	1 (14.3)	1 (14.3)	0 (0.0)	0 (0.0)	0 (0.0)	1 (2.5)	1 (2.5)	0 (0.0)	0 (0.0)	0 (0.0)
**Neoplasms benign, malignant an unspecified (incl cysts and polyps)**	1 (14.3)	1 (14.3)	0 (0.0)	0 (0.0)	0 (0.0)	5 (12.5)	4 (10.0)	1 (2.5)	0 (0.0)	0 (0.0)
Tumor pain	1 (14.3)	1 (14.3)	0 (0.0)	0 (0.0)	0 (0.0)	3 (7.5)	3 (7.5)	1 (2.5)	0 (0.0)	0 (0.0)
Cancer pain	0 (0.0)	0 (0.0)	0 (0.0)	0 (0.0)	0 (0.0)	1 (2.5)	1 (2.5)	0 (0.0)	0 (0.0)	0 (0.0)
Colorectal adenoma	0 (0.0)	0 (0.0)	0 (0.0)	0 (0.0)	0 (0.0)	1 (2.5)	0 (0.0)	0 (0.0)	0 (0.0)	0 (0.0)
**Vascular disorders**	1 (14.3)	1 (14.3)	0 (0.0)	0 (0.0)	0 (0.0)	4 (10.0)	3 (7.5)	2 (5.0)	1 (2.5)	1 (2.5)
Hypotension	1 (14.3)	1 (14.3)	0 (0.0)	0 (0.0)	0 (0.0)	3 (7.5)	1 (2.5)	0 (0.0)	0 (0.0)	0 (0.0)
Hypertension	0 (0.0)	0 (0.0)	0 (0.0)	0 (0.0)	0 (0.0)	1 (2.5)	1 (2.5)	1 (2.5)	0 (0.0)	0 (0.0)

Supplementary Table 1: TEAEs by Worst Grade, SOC and PT - SAF Analysis Set (continued)

**Primary System Organ Class**<br>Preferred Term	**3.2 mg/kg<br>n=7 (100%)**					**Total<br>N=40 (100%)**				
	**Any Grade<br>n (%)**	**Grade ≥2<br>n (%)**	**Grade ≥3<br>n (%)**	**Grade ≥4<br>n (%)**	**Grade =5<br>n (%)**	**Any Grade<br>n (%)**	**Grade ≥2<br>n (%)**	**Grade ≥3<br>n (%)**	**Grade ≥4<br>n (%)**	**Grade =5<br>n (%)**
Hypovolemic shock	0 (0.0)	0 (0.0)	0 (0.0)	0 (0.0)	0 (0.0)	1 (2.5)	1 (2.5)	1 (2.5)	1 (2.5)	1 (2.5)
**Psychiatric disorders**	2 (28.6)	0 (0.0)	0 (0.0)	0 (0.0)	0 (0.0)	3 (7.5)	0 (0.0)	0 (0.0)	0 (0.0)	0 (0.0)
Insomnia	1 (14.3)	0 (0.0)	0 (0.0)	0 (0.0)	0 (0.0)	1 (2.5)	0 (0.0)	0 (0.0)	0 (0.0)	0 (0.0)
Nervousness	0 (0.0)	0 (0.0)	0 (0.0)	0 (0.0)	0 (0.0)	1 (2.5)	0 (0.0)	0 (0.0)	0 (0.0)	0 (0.0)
Sleep disorder	1 (14.3)	0 (0.0)	0 (0.0)	0 (0.0)	0 (0.0)	1 (2.5)	0 (0.0)	0 (0.0)	0 (0.0)	0 (0.0)
**Reproductive system and breast disorders**	0 (0.0)	0 (0.0)	0 (0.0)	0 (0.0)	0 (0.0)	3 (7.5)	1 (2.5)	0 (0.0)	0 (0.0)	0 (0.0)
Intermenstrual bleeding	0 (0.0)	0 (0.0)	0 (0.0)	0 (0.0)	0 (0.0)	1 (2.5)	0 (0.0)	0 (0.0)	0 (0.0)	0 (0.0)
Pelvic pain	0 (0.0)	0 (0.0)	0 (0.0)	0 (0.0)	0 (0.0)	1 (2.5)	1 (2.5)	0 (0.0)	0 (0.0)	0 (0.0)
Perineal pain	0 (0.0)	0 (0.0)	0 (0.0)	0 (0.0)	0 (0.0)	1 (2.5)	0 (0.0)	0 (0.0)	0 (0.0)	0 (0.0)
**Cardiac disorders**	2 (28.6)	0 (0.0)	0 (0.0)	0 (0.0)	0 (0.0)	2 (5.0)	0 (0.0)	0 (0.0)	0 (0.0)	0 (0.0)
Palpitations	2 (28.6)	0 (0.0)	0 (0.0)	0 (0.0)	0 (0.0)	2 (5.0)	0 (0.0)	0 (0.0)	0 (0.0)	0 (0.0)
**Ear and labyrinth disorders**	1 (14.3)	0 (0.0)	0 (0.0)	0 (0.0)	0 (0.0)	1 (2.5)	0 (0.0)	0 (0.0)	0 (0.0)	0 (0.0)
Ear pain	1 (14.3)	0 (0.0)	0 (0.0)	0 (0.0)	0 (0.0)	1 (2.5)	0 (0.0)	0 (0.0)	0 (0.0)	0 (0.0)
**Endocrine disorders**	1 (14.3)	0 (0.0)	0 (0.0)	0 (0.0)	0 (0.0)	1 (2.5)	0 (0.0)	0 (0.0)	0 (0.0)	0 (0.0)
Hyperthyroidism	1 (14.3)	0 (0.0)	0 (0.0)	0 (0.0)	0 (0.0)	1 (2.5)	0 (0.0)	0 (0.0)	0 (0.0)	0 (0.0)
**Hepatobiliary disorders**	1 (14.3)	1 (14.3)	0 (0.0)	0 (0.0)	0 (0.0)	1 (2.5)	1 (2.5)	0 (0.0)	0 (0.0)	0 (0.0)
Cholangitis	1 (14.3)	1 (14.3)	0 (0.0)	0 (0.0)	0 (0.0)	1 (2.5)	1 (2.5)	0 (0.0)	0 (0.0)	0 (0.0)

MedDRA version 27.0, NCI-CTCAE version 5.0. Any grade includes ‘Missing’ grade

The precem-TcT antibody sequence is as follows:

Heavy chain

EVQLQESGPG LVKPSQTLSL TCTVSDGSVS RGGYYLTWIR QHPGKGLEWI 50
GYIYYSGSTY FNPSLRSRVT MSVDTSKNQF SLKLSSVTAA DTAVYYCARG 100
IAVAPFDYWG QGTLVTVSSA STKGPSVFPL APSSKSTSGG TAALGCLVKD 150
YFPEPVTVSW NSGALTSGVH TFPAVLQSSG LYSLSSVVTV PSSSLGTQTY 200
ICNVNHKPSN TKVDKRVEPK SCDKTHTCPP CPAPPVAGPS VFLFPPKPKD 250
TLMISRTPEV TCVVVDVSHE DPEVKFNWYV DGVEVHNAKT KPREEQYNST 300
YRVVSVLTVL HQDWLNGKEY KCKVSNKALP SSIEKTISKA KGQPREPQVY 350
TLPPSREEMT KNQVSLTCLV KGFYPSDIAV EWESNGQPEN NYKTTPPVLD 400
SDGSFFLYSK LTVDKSRWQQ GNVFSCSVMH EALHNHYTQK SLSLSPG 447

Light chain

EIVLTQSPAT LSVSPGERAT LSCRTSQSVR SNLAWYQQKP GQAPRLLIYA 50
ASTRATGIPA RFSGSGSGTE FTLTISSLQS EDFAVYYCQQ YTNWPFTFGP 100
GTKVDIKRTV AAPSVFIFPP SDEQLKSGTA SVVCLLNNFY PREAKVQWKV 150
DNALQSGNSQ ESVTEQDSKD STYSLSSTLT LSKADYEKHK VYACEVTHQG 200
LSSPVTKSFN RGEC 214

Clinical Study Protocol

Title Page

**Clinical Study Protocol Title:**	A Phase 1, Two-Part, Multicenter, Open-Label First-in-Human Study of anti-CEACAM5 Antibody-Drug Conjugate M9140 in Participants with Advanced Solid Tumors
**Study Number:**	MS202329_0001
**Protocol Version:**	29 July 2024/Version 5.0
**Merck Compound:**	M9140
**Merck Registered Compound Name in Japan:**	Not Applicable
**Study Phase:**	1
**Short Title:**	Anti-CEACAM5 ADC M9140 in Advanced Solid Tumors
**Acronym or Abbreviation**	PROCEADE-CRC-01
**Coordinating Investigator:**	PPD, M.D, Ph.D, FACP<br>MD Anderson Cancer Center, Houston, Texas, US
**Sponsor Name and Legal Registered Address:**	Sponsor:<br>Affiliates of Merck KGaA, Darmstadt, Germany<br><br>For all countries, except the US and Canada:<br>Merck Healthcare KGaA, Darmstadt, Germany<br>an affiliate of Merck KGaA, Darmstadt, Germany<br>Frankfurter Str. 250<br>64293, Darmstadt, Germany<br><br>In the US and Canada:<br>EMD Serono Research & Development Institute, Inc.<br>an affiliate of Merck KGaA, Darmstadt, Germany<br>45A Middlesex Turnpike<br>Billerica, MA, 01821, USA<br><br>Local Sponsor for Sites in Japan:<br>Merck Biopharma Co., Ltd. Japan<br>an affiliate of Merck KGaA, Darmstadt, Germany<br>Arco Tower, 1-8-1 Shimomeguro<br>Meguro-ku, Tokyo 153-8926, Japan

**Regulatory Agency Identifying Numbers:**	US FDA IND: 156588<br>EU trial number: 2022-500508-23-00

Protocol Amendment Summary of Changes

Protocol History

Version Number	Type	Version Date
5.0	Global Amendment	29-July-2024
3.3 EU	Local Amendment	18-July-2024
4.0	Global Amendment	20-June-2024; released to CROs but not submitted
3.2 KR	Local Amendment	22-May-2024
3.1 US	Local Amendment	03-April-2024
3.0	Global Amendment	17-January-2024
2.8 JP	Local Amendment	23-August-2023
2.7 US	Local Amendment	23-August-2023
2.6 ES	Local Amendment	23-August-2023
2.5 JP	Local Amendment	27-April-2023
2.4 US	Local Amendment	28-April-2023
2.3 ES	Local Amendment	25-April-2023
2.2 JP	Local Amendment	27-June-2022
2.1 US	Local Amendment	24-May-2022
2.0	Global Amendment	24-May-2022
1.0	Original Protocol	12-April-2022

Protocol Version 5.0 (29 July 2024)

Overall Rationale for the Amendment

The protocol has been revised to incorporate changes from local amendments based on feedback from regulatory authorities in a new global version of the protocol and to introduce a new Part 2D that combines M9140 with 5-FU and bevacizumab. In addition, clarification of verbiage on eligibility criteria (past treatment lines, brain metastases) and concomitant medication use was added, based on interactions with Investigators.

The protocol v4.0, dated 20 June 2024, was planned to introduce a new Part 2D that combines M9140 with 5-FU and bevacizumab. Protocol v4.0 was finalized and released but not submitted to any Health Authority. Meanwhile, feedback from European regulatory authorities triggered a local protocol amendment v3.3 EU. To achieve a harmonized protocol version once again, all local changes and revised content from protocol v4.0 (introduction of Part 2D) were merged and

integrated into a new protocol v5.0. A summary of these combined changes is shown in the table below.

Section # and Name	Description of Change	Brief Rationale
1.1 Synopsis<br>1.2 Schema<br>1.3.5 Part 2D – M9140 [redacted] in Combination with 5-FU plus Bevacizumab<br>3.3 Part 2B (M9140 [redacted]), Part 2C (M9140 Q3W in Combination with Bevacizumab or Bevacizumab plus Capecitabine), and Part 2D (M9140 [redacted] in Combination with 5-FU plus Bevacizumab)<br>4.1 Overall Design<br>4.2.2 Parts 2A, 2B, 2C, and 2D – Dose Expansion<br>4.3.2.4 Part 2D – M9140 [redacted] in Combination with 5-FU plus Bevacizumab<br>5.2 Exclusion Criteria<br>6.1 Study Intervention Administration<br>6.4 Study Intervention Compliance<br>6.5.2 Dose Selection<br>6.5.3 Safety Monitoring Committee<br>6.5.4 Definition of Dose-limiting Toxicity<br>6.5.5 Dose Modification<br>6.8.2 Permitted Medicines<br>6.8.3 Prohibited Medicines<br>8.1 Efficacy Assessments and Procedures<br>8.2.4 Clinical Safety Laboratory Assessments<br>8.4 Pharmacokinetics<br>9.2 Sample Size Determination<br>9.4.2.2 Dose Recommendations for Expansion<br>9.4.4 Sequence of Analyses<br>10 References<br>Appendix 6	Addition of Part 2D with M9140 in combination with 5-FU, folinic acid, and bevacizumab.	To evaluate the potential of M9140 to be combined with standard of care agents in the treatment of mCRC.

Section # and Name	Description of Change	Brief Rationale
1.1 Synopsis<br>3 Objectives and Endpoints<br>9.4.1 Efficacy Analyses	Inclusion of a secondary endpoint to assess disease control rate in Part 2.	The dose escalation part demonstrated that a significant percentage of participants experienced a benefit from the treatment in terms of long-term disease stabilization that was also reflected in the mPFS observed. For that reason, analyzing disease control rate is a relevant endpoint that would give a more complete picture of the anti-tumor activity of M9140.
1.1 Synopsis<br>1.3.2 Schedule of Activities Part 2A<br>4.1 Overall Design<br>4.4.2 End of Study Definition	Prolongation of maximum survival follow-up up to 24 months.	More mature data from the dose escalation part of the study have demonstrated treatment duration of > 12 cycles for some participants. Longer OS follow up will be needed to capture OS duration for those participants.
1.1 Synopsis	Removal of justification #2.	Legacy text removed to be consistent with main text.
1.3 Schedule of Activities	Removed the specification for chest and abdomen from the CT scan or MRI (Table 3, Table 5, Table 7, Table 9)<br>Specified that all sampling timepoints are based on the start of M9140 infusion (Table 4, Table 6, Table 8, and Table 10)	Removed as full details are given in Section 8.1.<br>To provide clarity on timepoints of sampling.
1.3.4 Part 2C – M9140 Q3W in Combination with Bevacizumab or Bevacizumab plus Capecitabine<br>Appendix 6	Additions to Part 2C urinalysis: If dipstick proteinuria ≥ 2+, a 24-hour urine collection is required.	For quantitative assessment of proteinuria due to increased risk of proteinuria when treated with bevacizumab.
1.3.5 Part 2D – M9140 [redacted] in Combination with 5-FU plus Bevacizumab	Table 10: On C3D1 EOI no bevacizumab concentrations are to be measured.	Correction of error detected for bevacizumab PK sampling.
2.3.1 Risk Assessment	Benefit-risk assessment updated with details applicable for 5-FU and folinic acid.<br>Other risks updated with phototoxicity.	Modified to align with risks for the study interventions to be administered in Part 2D.<br>To include sun protection measures as used in other studies involving exatecan as suggested by HA.
4.1 Overall Design	Clarified that enrollment in Part 2C2 can only start after dose level 1 in Part 2C1 has been deemed safe and tolerable by the safety monitoring committee.	To clarify sequence of enrollment.

Section # and Name	Description of Change	Brief Rationale
	Text added to describe that for Part 2B, the Sponsor upon recommendation by the SMC and using emerging data from Part 2A may evaluate other doses “as long as they do not exceed the time-averaged dose intensity corresponding to the MTD defined with Q3W regimen.”	To clarify for Part 2B the highest dose tested cannot exceed the time-averaged dose intensity corresponding to the MTD in the Q3W regimen.
4.1 Overall Design (Part 2C2)	• Paragraph revision on sentinel dosing in Parts 2C1 and 2C2<br>• Clarification that enrollment in Part 2C2 can only start after dose level 1 in Part 2C1 has been deemed safe and tolerable by the SMC.	• Implementation of sentinel dosing verbiage between corresponding dose levels of different parts (2C1 and 2C2) in Part 2C<br>• To clarify sequence of enrollment in Part 2C.
4.1 Overall Design<br>4.3.2.3 Part 2C Justification for Dose<br>9.4.2.2 Dose Recommendations for Expansion	Dose level 1 in Part 2C2 will include capecitabine at its lower approved dose (800 mg/m^2). If the dose is deemed tolerable by the SMC, capecitabine can be escalated to 1000 mg/m^2.	For participant safety purposes, dosing will start with the lower approved dose of capecitabine.
3 Objectives and Endpoints (Part 2: Table 13 and Table 14) 4.1 Overall Design<br>5 Study Population<br>5.1 Inclusion Criteria	Description of the study population updated to clarify prior lines of therapy.	Updated for clarity about previous lines of treatments (including experimental treatments) and to provide additional guidance about differentiation between adjuvant/neoadjuvant and metastatic lines.
4.2.3 Rationale for Treatment of CRC with anti CEACAM5 ADC	Text added to describe the all-comers approach without the need of participant selection given the high prevalence of CEACAM5 expression in mCRC.	To justify that participants can be included in the study without prospectively testing for CEACAM5 expression.
4.3.2 Parts 2A, 2B, 2C, and 2D – Dose Expansion	Updated with end of dose escalation data from Part 1.	To provide the most updated information available.
	Text added to provide additional information regarding the preliminary clinical data from the dose escalation part of the study to include occurrence of a Grade 5 adverse event.	Health Authority request to provide additional information.
5 Study Population<br>5.1 Inclusion Criteria	Pasted details for participants with known BRAF mutation.	Aligned with text in Section 4.1.
Appendix 2	ICF signing by legal representative removed.	Participant must be able to sign the ICF.
5.1 Inclusion Criteria	The time period for avoiding breastfeeding is 3 months in Part 1, 2A, and 2B, and 6 months in Part 2C and 2D.	To align with the product information for the respective standard of care agents.
5.1 Inclusion criteria	Clarification about wash-out period of prior anticancer therapies	Updated for clarity and completeness

Section # and Name	Description of Change	Brief Rationale
5.2 Exclusion Criteria	Legacy text about GC/[redacted] removed.	As per previous amendment, GC/[redacted] is no longer part of study population. Text removed to align.
	Exclusion criteria added for Part 2C and 2D.	To conform with the use of bevacizumab, capecitabine and 5-FU in these parts.
	Clarification on exclusion of participants with brain metastases	To provide clarity about exceptions of this criterion.
Section 5.3.4 Other Lifestyle Considerations	New section added.	To provide instructions on sunlight exposure of participant.
6.1 Study Intervention Administration<br>Appendix 11 Country-specific Requirements	• Clarified that each vial will be labeled per country-specific requirements (Table 17 and Table 18).<br>• Changed Appendix 11 title to 'Country-specific Requirements' and text added to describe EU Regulation 536/2024 (Annex VI, A1, paragraph 3).	• To clarify labeling requirements<br>• To clarify that as per EU Regulation 536/2024 (Annex VI, A1, paragraph 3), the address and telephone number of the main contact for information on the product and clinical study is not on the label but on the Patient ID card.
6.5.3 Safety Monitoring Committee	Text added to describe criteria that would trigger an ad hoc SMC meeting to decide continuation or discontinuation of a participant, dose level, or study part in case of unexpected toxicity.	To define criteria that would trigger an ad hoc SMC meeting as requested by HA.
6.5.4 Definition of Dose-limiting Toxicity	Sentence on study accrual hold for ad hoc SMC meeting following Grade 5 event was moved to Section 6.5.3.	Information now combined with changes made in Section 6.5.3 for clarity.
6.5.5 Dose Modification	Included reference to local prescribing information of bevacizumab.	To conform with the use of bevacizumab.
	Table 19:<br>• Clarification that consideration on dose reduction applies only in cases of Grade 4 thrombocytopenia events with no complications that last ≥ 7 days.<br>• Separated thrombocytopenia with and without clinically significant bleeding and long-lasting thrombocytopenia without clinically significant bleeding.	• Modified for clarity and consistency<br>• Modified for better readability and clearer separation.

Section # and Name	Description of Change	Brief Rationale
	• Added recommendation for second recurrence of Grade 4 ‘neutrophil count decreased’ and first recurrence of other Grade 4 non-hematological toxicities (except GI)<br>• Addition of anaphylaxis/anaphylactic reactions specifically within IRR	• Modified for clarity and completeness.<br>• To provide guidance on the management of anaphylaxis/anaphylactic reactions with M9140
	• Dose-modification for capecitabine included only in Table 20.<br>• Added text to clarify that once dose of capecitabine has been reduced, it should not be increased at a later time.<br>• Added text on dose modification of capecitabine in case of decrease in neutrophil or platelet counts or creatinine clearance.	To clarify which guidelines to apply for managing adverse reactions to capecitabine.
	Addition of Table 21 for management (temporary/permanent discontinuation) of adverse reactions that may occur when administering bevacizumab.	To clarify management of adverse reactions to bevacizumab.
	Addition of Table 22 for management of adverse reactions that may occur when administering 5-FU.	To clarify management of adverse reactions to 5-FU.
	Modified text and tables on individual dose modifications, interruptions, or discontinuations for the combination drugs.	To clarify the dose modification with respect to the different SoC compounds.
6.7 Treatment of Overdose	Clarified that for any overdose of approved therapy, reference should be made to the local prescribing information on handling and side effects for the respective treatment.	To avoid listing details for each approved therapy regarding overdose symptoms and any local treatment details in the protocol.
6.8.2 Permitted Medicines<br>6.8.3 Prohibited Medicines	Text adapted/moved to clarify the guidance on use of live and non-live vaccines before first M9140 dose and during the study.	To provide clearer guidance on vaccination prior and during the study.
6.8.2 Permitted Medicines	Updated to permit limited use of steroids.	To provide clearer guidance on the use of steroids.
6.8.3 Prohibited Medicines	Clarification that the period for prohibition of medicines is valid until Safety Follow-up Visit only, unless otherwise noted.	To clarify the end of restriction.

Section # and Name	Description of Change	Brief Rationale
	Use of anticoagulants or thrombolytic agents is not allowed in Part 2C and 2D.	Modified in line with the newly added exclusion criterion conforming with the use of bevacizumab.
	Use of brivudine, sorivudine or their analogues is not allowed in Part 2C2 and 2D.	Modified in line with the newly added exclusion criterion conforming with the use of capecitabine.
	Clarification that also herbal/natural products which are potential CYP3A inhibitors/inducers are prohibited.	To clarify the extent of prohibition of CYP3A inhibitors/inducers.
	Text added to refer to the local product labels for details on drug-drug interactions with capecitabine, 5-FU, or bevacizumab or folinic acid.	Modified in line with reference to drug-drug interactions described in local product labels.
	Addition of prohibited dose and duration thresholds of corticosteroids.	Updated for clarity and completeness.
	Clarification about types of anticancer therapies prohibited during the study.	Updated for clarity and completeness
6.8.4 Other Interventions	Treatment with bevacizumab should be interrupted for elective surgery.	To conform with the use of bevacizumab.
	Clarification added that concurrent participation in other clinical studies is not permitted until Safety Follow-up Visit.	To clarify restrictions in terms of study participation.
7.1 Discontinuation of Study Intervention	Study intervention stopping criteria for liver events have been added.	Included for patient-safety purposes.
	Clarified that a participant must be withdrawn from any study intervention at the participant's request.	For clarity.
8.1 Efficacy Assessments and Procedures	Updated to clarify that imaging of chest/abdomen/pelvis and other anatomical regions (as clinically indicated) should be performed with other approved techniques in case CT/MRI is insufficient.	For clarity and completeness.
8.3.3 Regulatory Reporting Requirements for Serious Adverse Events<br>Appendix 4	Update of reporting procedure for SAEs and SUSARs.	To align with current Sponsor process.
8.3.3 Regulatory Reporting Requirements for Serious Adverse Events<br>Appendix 11 Country-specific Requirements	Added SUSAR reporting requirements according to EU Regulation 536/2014.	To clarify the SUSAR reporting requirements procedures according to EU Regulation 536/2014.
8.3.7 Adverse Event of Special Interest (Part 2)	Updated definition of AESIs.	Updated for clarity.

Section # and Name	Description of Change	Brief Rationale
8.3.7 Adverse Event of Special Interest<br>Appendix 4	Update of reporting procedure for AESIs.	To align with current Sponsor process.
8.4 Pharmacokinetics<br>8.6 Biomarkers<br>8.7 Immunogenicity Assessments	Removal of specific volumes to be collected.	To avoid local protocol amendments in case of differences in local standards.
9.4.1 Efficacy Analyses	Addition of details for analysis and of DoR and TTR.	To clearly define the planned analyses.
9.4.3 Other Analyses	Removal of non-renal clearance assessment for urine sample	Only renal clearance can be assessed in urine sample
9.4.4 Sequence of Analyses	Added option to perform exploratory analysis at end of dose optimization (Part 2A).	To ensure establishing optimal dose for future studies in a timely manner.
Appendix 2 Study Governance	Removal of language concerning the participant's legally authorized representative.	To align with current Sponsor protocol template
Appendix 6 Clinical Laboratory Tests	Clarified that coagulation profile testing is required also at End of Treatment and Safety Follow-up visits.	To clarify timepoints for assessment of coagulation.
Appendix 7 List of Strong and Moderate CYP Inhibitors and Inducers	Added link to website for more details on CYP inhibitors and inducers.	To make information easier accessible.
Appendix 11 Country-specific Requirements	Added definition of new fact.	Included definition of new fact as requested by HA.
Appendix 11 Japan-specific Requirements	Addition of oral progesterone-only containing contraceptives to list of prohibited contraceptive methods in Japan.	To align with contraceptive methods permitted in Japan.
10 References	Alignment of references with text.	Updated for consistency.
Throughout document	Minor editorial, typographical, and document-formatting changes	

CCI [redacted]

Table of Contents

Title Page 1
Table of Contents 10
Table of Tables 14
Table of Figures 16
1 Protocol Summary 17
1.1 Synopsis 17
1.2 Schema 23
1.3 Schedule of Activities 26
1.3.1 Part 1 – M9140 Q3W Dose Escalation 26
1.3.2 Part 2A – M9140 Q3W Dose Optimization 32
1.3.3 Part 2B – M9140 [redacted] Regimen 41
1.3.4 Part 2C – M9140 Q3W in Combination with Bevacizumab or Bevacizumab plus Capecitabine 45
1.3.5 Part 2D – M9140 [redacted] in Combination with 5-FU plus Bevacizumab 52
2 Introduction 58
2.1 Study Rationale 58
2.2 Background 58
2.3 Benefit/Risk Assessment 59
2.3.1 Risk Assessment 60
2.3.2 Benefit Assessment 64
2.3.3 Overall Benefit/Risk Conclusion 65
3 Objectives and Endpoints 65
3.1 Part 1 – M9140 Q3W Dose Escalation 65
3.2 Part 2A – M9140 Q3W Dose Optimization 67
3.3 Part 2B (M9140 [redacted]), Part 2C (M9140 Q3W in Combination with Bevacizumab or Bevacizumab plus Capecitabine), and Part 2D (M9140 [redacted] in Combination with 5-FU plus Bevacizumab).....70
4 Study Design 73
4.1 Overall Design 73
4.2 Scientific Rationale for Study Design 82
4.2.1 Part 1 – M9140 Dose Escalation 82

4.2.2 Parts 2A, 2B, 2C, and 2D – Dose Expansion 83
4.2.3 Rationale for Treatment of CRC with anti-CEACAM5 ADC....83
4.2.4 Participant Input into Design....84
4.3 Justification for Dose....84
4.3.1 Part 1 – M9140 Dose Escalation 84
4.3.2 Parts 2A, 2B, 2C, and 2D – Dose Expansion 86
4.3.2.1 Part 2A – M9140 Q3W Dose Optimization 87
4.3.2.2 Part 2B – M9140 [redacted] Regimen....87
4.3.2.3 Part 2C – M9140 Q3W in Combination with Bevacizumab (Part 2C1) or Bevacizumab plus Capecitabine (Part 2C2)....87
4.3.2.4 Part 2D – M9140 [redacted] in Combination with 5-FU plus Bevacizumab....88
4.4 End of Study Definition....88
4.4.1 Part 1 – Dose Escalation....88
4.4.2 Part 2 – Dose Expansion....88
5 Study Population....89
5.1 Inclusion Criteria 91
5.2 Exclusion Criteria 96
5.3 Lifestyle Considerations 99
5.3.1 Meals and Dietary Restrictions....99
5.3.2 Caffeine, Alcohol, Tobacco, and Cannabinoid....99
5.3.3 Activity 99
5.3.4 Other Lifestyle Considerations....99
5.4 Screen Failures....99
6 Study Intervention and Concomitant Therapies 99
6.1 Study Intervention Administration 100
6.2 Study Intervention Preparation, Handling, Storage, and Accountability....103
6.3 Measures to Minimize Bias: Study Intervention Assignment and Blinding 104
6.3.1 Study Intervention Assignment 104
6.3.2 Blinding 105
6.3.3 Emergency Unblinding....105

6.4 Study Intervention Compliance 105
6.5 Dose Modification 106
6.5.1 Retreatment Criteria...106
6.5.2 Dose Selection 106
6.5.3 Safety Monitoring Committee 107
6.5.4 Definition of Dose-limiting Toxicity..108
6.5.5 Dose Modification 110
6.5.5.1 Switching Between M9140 Dose Levels: Part 2A Dose Optimization 117
6.6 Continued Access to Study Intervention After the End of the Study 117
6.7 Treatment of Overdose 117
6.8 Concomitant Therapy 118
6.8.1 Rescue Medicine...118
6.8.2 Permitted Medicines 118
6.8.3 Prohibited Medicines 119
6.8.4 Other Interventions 120
7 Discontinuation of Study Intervention and Participant Discontinuation/Withdrawal...121
7.1 Discontinuation of Study Intervention..121
7.2 Participant Discontinuation/Withdrawal from the Study 122
7.3 Lost to Follow-Up..122
8 Study Assessments and Procedures 122
8.1 Efficacy Assessments and Procedures..123
8.2 Safety Assessments and Procedures 124
8.2.1 Physical Examinations...124
8.2.2 Vital Signs 124
8.2.3 Electrocardiograms 125
8.2.3.1 Safety ECGs..125
8.2.3.2 QT/QTc Evaluation 126
8.2.4 Clinical Safety Laboratory Assessments 126
8.2.5 Patient-Reported Symptomatic Adverse Events..............................126
8.2.5.1 PRO-CTCAE Item Library...127

8.2.6 Suicidal Ideation and Behavior Risk Monitoring 127
8.3 Adverse Events, Serious Adverse Events, and Other Safety Reporting 127
8.3.1 Method of Detecting Adverse Events and Serious Adverse Events .128
8.3.2 Follow-up of Adverse Events and Serious Adverse Events 128
8.3.3 Regulatory Reporting Requirements for Serious Adverse Events ...128
8.3.4 Pregnancy 129
8.3.5 Cardiovascular and Death Events 130
8.3.6 Disease-Related Events and/or Disease-Related Outcomes Not Qualifying as AEs or SAEs 130
8.3.7 Adverse Events of Special Interest (Part 2) 130
8.4 Pharmacokinetics 131
CCI [redacted]
8.7 Immunogenicity Assessments 134
9 Statistical Considerations 135
9.1 Statistical Hypotheses 135
9.2 Sample Size Determination 135
9.2.1 Part 2A: Interim Futility Analyses 136
9.2.2 Part 2A: Dose Optimization 136
9.3 Analyses Sets 138
9.4 Statistical Analyses 139
9.4.1 Efficacy Analyses 139
9.4.2 Safety Analyses 140
9.4.2.1 Dose Escalation 141
9.4.2.2 Dose Recommendations for Expansion 143
9.4.3 Other Analyses 147
9.4.4 Sequence of Analyses 149
10 References 151
11 Appendices 156
Appendix 1 Abbreviations 156
Appendix 2 Study Governance 160
Appendix 3 Contraception and Barrier Requirements 166

Appendix 4 Adverse Events: Definitions and Procedures for Recording, Evaluating, Follow-up, and Reporting 168
Appendix 5 Liver Safety: Suggested Actions and Follow-up Assessments 175
Appendix 6 Clinical Laboratory Tests 176
Appendix 7 List of Strong and Moderate CYP Inhibitors and Inducers 178
Appendix 8 Model for Bayesian Dose Escalation 179
Appendix 9 Response Evaluation Criteria in Solid Tumors (RECIST) Version 1.1 182
Appendix 10 Protocol Amendment History 192
Appendix 11 Country-specific Requirements 224
Appendix 12 NCI-PRO-CTCAE® Custom Survey 227
Appendix 13 Sponsor Signature Page 228
Appendix 14 Coordinating Investigator Signature Page 229
Appendix 15 Principal Investigator Signature Page 230

Table of Tables

Table 1 Schedule of Activities for Part 1A and Part 1B – Dose Escalation (Q3W) 26
Table 2 Schedule of ECG, PK, [redacted], ADA Assessments, and Urine Collection – Part 1 (Dose Escalation Q3W) 31
Table 3 Schedule of Activities for Part 2A– Dose Optimization (Q3W) 32
Table 4 Schedule of ECG, PK, [redacted], and ADA Assessments during Part 2A (M9140 Dose Optimization Q3W) 40
Table 5 Schedule of Activities for Part 2B – M9140 [redacted] regimen 41
Table 6 Schedule of PK, [redacted], and ADA Assessments – Part 2B M9140 [redacted] regimen 44
Table 7 Schedule of Activities for Part 2C – M9140 Q3W in Combination with Bevacizumab or Bevacizumab plus Capecitabine 45
Table 8 Schedule of PK, [redacted], and ADA Assessments during Part 2C M9140 Q3W in Combination with Bevacizumab or Bevacizumab plus Capecitabine 51
Table 9 Schedule of Activities for Part 2D – M9140 [redacted] in Combination with 5-FU plus Bevacizumab 52

Table 10	Schedule of PK, [redacted], and ADA Assessments during Part 2D M9140 [redacted] in Combination with 5-FU plus Bevacizumab 57
Table 11	Identified and Potential Risks and Their Management (Data Cutoff Date 20-November-2023) 60
Table 12	Objectives and Endpoints: Part 1A and Part 1B -- Dose Escalation ..65
Table 13	Objectives and Endpoints/Estimands: Part 2A – M9140 Q3W Dose Optimization 67
Table 14	Objectives and Endpoints/Estimands: Part 2B, Part 2C, and Part 2D 70
[redacted]	
Table 16	Comparison of Main PK Parameters for Exatecan Between Caucasian and Japanese Populations (Median, Range) Based on Published Data 90
Table 17	Study Intervention Administered in Part 1A and Part 1B 100
Table 18	Study Interventions Administered in Part 2A, Part 2B, Part 2C, and Part 2D 101
Table 19	Dose Modifications and Temporary/Permanent Treatment Discontinuation of M9140 111
Table 20	Dose Modifications for Capecitabine According to Toxicity Grades 114
Table 21	Temporary/Permanent Discontinuation of Bevacizumab for Bevacizumab-related Toxicities (Part 2C and 2D) 115
Table 22	Dose Modification and Temporary/Permanent Discontinuation of 5-FU for related Toxicities (Part 2D) 115
Table 23	Symptomatic Toxicity Items 127
Table 24	Decision Criteria at Interim Analysis for Part 2A 136
Table 25	Posterior Probabilities for ORR Difference Between Higher And Lower Dose Given Observed Data. 137
Table 26	Posterior Probabilities for Difference In Grade ≥ 3 AEoI Or Dose Modification Rates Between Higher and Lower Dose Given Observed Data. 137
Table 27	Description of the Analysis Sets 138
Table 28	Efficacy Analyses 139
Table 29	Safety Analyses 140
[redacted]	

Table of Figures

Figure 1 Overall Study Design ... 23

Figure 2 Overall Study Design for Part 2C – M9140 Q3W in Combination with Bevacizumab or Bevacizumab plus Capecitabine ... 24

Figure 3 Overall Study Design for Part 2D – M9140 [redacted] in Combination with 5-FU plus Bevacizumab ... 25

[redacted]

Figure 5 Flowchart for trial conduct using the iBOIN design. ... 145

1 Protocol Summary

1.1 Synopsis

Protocol Title: A Phase 1, Two-Part, Multicenter, Open-Label First-in-Human Study of anti-CEACAM5 Antibody-Drug Conjugate M9140 in Participants with Advanced Solid Tumors

Short Title: Anti-CEACAM5 ADC M9140 in Advanced Solid Tumors

Rationale:

The administration of M9140 to participants with specified locally advanced or metastatic solid tumors, is justified by the following: 1) Carcinoembryonic Antigen-Related Cell Adhesion Molecule 5 (CEACAM5) has limited expression in adult normal tissues, but is expressed at mid to high levels in various adenocarcinomas such as in colorectal cancer (CRC) and gastric cancer (GC), [redacted] providing an attractive target for the development of antibody-drug conjugates (ADCs). 2) In patient-derived xenograft (PDX) models, M9140 demonstrated strong efficacy in CEACAM5-expressing tumors, including CRC, GC, [redacted]. 3) M9140 induced dose-dependent reversible adverse events (AEs) in the hematolymphoid and gastrointestinal systems of monkeys that resemble exatecan toxicity. These risks can be adequately managed with the proposed study design. 4) Preliminary clinical data from the dose escalation Part 1 of the study have demonstrated a manageable safety profile consistent with exatecan toxicity with no new, unexpected AEs observed and encouraging preliminary antitumor activity of M9140 in heavily pretreated patients with colorectal cancer.

Objectives and Endpoints:

Objectives and Endpoints: Part 1A and Part 1B – M9140 Q3W Dose Escalation

Objectives	Endpoints
**Primary**	
To determine dose-toxicity relationship and MTD (if reached) of M9140 as monotherapy (Part 1A) and of M9140 monotherapy with pegfilgrastim prophylaxis (Part 1B) for investigated regimens in patients with locally advanced or metastatic CRC, respectively.	• Occurrence of DLTs.<br>• Occurrence of AEs.
To determine the RDE of M9140 as monotherapy (Part 1A) and of M9140 monotherapy with pegfilgrastim prophylaxis (Part 1B) for investigated regimens, respectively.	In addition to AEs and DLTs, the PK profile and preliminary clinical activity will be considered to determine the RDE.
**Secondary**	
To characterize the PK profile of M9140 (conjugated antibody, total antibody, and unconjugated exatecan payload)	PK parameters of M9140 conjugated antibody, total antibody, and unconjugated exatecan using noncompartmental analysis, e.g., AUC_{0-t}, AUC_{τ}, C_{max}, C_{trough}, Cl, V_d, $t_{½}$, CLr.
To evaluate indicators of clinical activity of M9140 in terms of OR using RECIST v1.1.	OR according to RECIST v1.1 as assessed by Investigators.

Objectives	Endpoints
To evaluate indicators of clinical activity of M9140 in terms of DoR using RECIST v1.1.	DoR according to RECIST v1.1 as assessed by Investigators, defined as time from first documentation of OR to PD or death, occurring within 2 scheduled tumor assessments after last evaluable assessment or start of treatment.
To evaluate indicators of clinical activity of M9140 in terms of PFS (RECIST v1.1).	PFS as defined from date of first study intervention to PD according to RECIST v1.1 as assessed by Investigators or death. Death events are considered only if occurring within 2 scheduled tumor assessments after last evaluable assessment or start of treatment.
To characterize the immunogenicity of M9140.	ADA against M9140 occurrence and titer, as measured by ADA assay.
To assess the effect of M9140 on QTc interval.	Triplicate digital ECG measures, change from the baseline QTc (ΔQTc) over predefined timepoints.

AE: adverse event, ADA: anti-drug-antibodies, CRC: colorectal cancer, DLT: dose-limiting toxicity, DoR: duration of response, ECG: electrocardiogram, MTD: maximum tolerated dose, OR: objective response, PD: progressive disease, PFS: progression-free survival, PK: pharmacokinetic, QTc: corrected QT interval, RDE: recommended dose for expansion, RECIST: Response Evaluation Criteria in Solid Tumors.

Objectives and Endpoints: Part 2A – M9140 Q3W Dose Optimization

Objectives	Endpoints
**Primary**	
To determine clinical activity of two M9140 dose levels in terms of OR and DoR to inform selection of the RP2D of M9140 monotherapy Q3W in patients with locally advanced or metastatic CRC.	• OR according to RECIST v1.1 as assessed by Investigators.<br>• DoR according to RECIST v1.1 as assessed by Investigators, defined as time from first documentation of objective response to PD or death, occurring within 2 scheduled tumor assessments after last evaluable assessment or start of treatment.
To determine safety and tolerability of two M9140 dose levels to inform selection of RP2D of M9140 in patients with locally advanced or metastatic CRC.	Occurrence of AEs.
**Secondary**	
To evaluate indicators of clinical activity of M9140 in terms of DC at 12 weeks.	DC defined as CR, PR, SD, or non-CR/non-PD at week 12 visit or later prior to documented PD.
To evaluate indicators of clinical activity of M9140 in terms of time to response and PFS using RECIST v1.1.	Time to response defined as time from date of randomization to first documentation of objective response according to RECIST v1.1 as assessed by investigators.<br>PFS as defined from date of randomization to PD according to RECIST v1.1 as assessed by Investigators or death.
To evaluate indicators of clinical activity of M9140 in terms of OS.	OS defined as time from date of randomization to death.
To assess the effect of M9140 on QTc interval.	Triplicate digital ECG measures, change from baseline QTc (ΔQTc) over predefined timepoints to contribute to concentration-QTc analysis.
To characterize the PK profile of M9140 (conjugated antibody, total antibody and unconjugated exatecan payload).	PK parameters of M9140 conjugated antibody, total antibody, and unconjugated exatecan using

Objectives	Endpoints
	noncompartmental analysis, e.g., AUC_{0-t}, AUC_{τ}, C_{max}, C_{trough}, Cl, V_d, $t_{½}$.
To characterize the immunogenicity of M9140.	ADA against M9140: occurrence and titer, as measured by ADA assay.
To describe patient-reported symptomatic AEs of M9140 by dose levels in Arm A1 and Arm A2.	Symptomatic AEs and related impacts as measured by selected questions addressing fatigue, nausea, vomiting, diarrhea, mouth sores.

AE: adverse event, ADA: anti-drug-antibodies, CEACAM5: Carcinoembryonic Antigen-Related Cell Adhesion Molecule 5, CRC: colorectal cancer, DoR: duration of response, ECG: electrocardiogram, OR: objective response, OS: overall survival, PFS: progression-free survival, PK: pharmacokinetics, QTc: corrected QT interval, RECIST: Response Evaluation Criteria in Solid Tumors, RP2D: recommended Phase 2 dose.

Objectives and Endpoints: Part 2B M9140 [redacted] Regimen, Part 2C M9140 Q3W in Combination with Bevacizumab or Bevacizumab plus Capecitabine, and Part 2D M9140 [redacted] in Combination with 5-FU plus Bevacizumab

Objectives	Endpoints
**Primary**	
To assess safety to inform selection of the RP2D of M9140 as monotherapy given [redacted] (Part 2B), of M9140 Q3W in combination with bevacizumab and bevacizumab plus capecitabine (Part 2C), and of M9140 [redacted] in combination with 5-FU plus bevacizumab (Part 2D), in patients with locally advanced or metastatic CRC.	• Occurrence of DLTs<br>• Occurrence of AEs
**Secondary**	
To evaluate indicators of clinical activity of M9140 as monotherapy given [redacted] (Part 2B), of M9140 Q3W in combination with bevacizumab and bevacizumab plus capecitabine (Part 2C), and of M9140 [redacted] in combination with 5-FU plus bevacizumab (Part 2D) in terms of OR and DC at 12 weeks using RECIST v1.1.	• OR according to RECIST v1.1 as assessed by Investigators.<br>• DC defined as CR, PR, SD, or non-CR/non-PD at week 12 visit or later prior to documented PD
To evaluate indicators of clinical activity of M9140 in terms of time to response using RECIST v1.1.	Time to response defined as time from date of first study intervention to first documentation of OR according to RECIST v1.1 as assessed by Investigators.
To evaluate indicators of clinical activity of M9140 in terms of DoR using RECIST v1.1.	DoR according to RECIST v1.1 as assessed by Investigators, defined as time from first documentation of OR to PD or death, occurring within 2 scheduled tumor assessments after last evaluable assessment or start of treatment.
To evaluate indicators of clinical activity of M9140 in terms of PFS (RECIST v1.1).	PFS as defined from date of first study intervention to PD according to RECIST v1.1 as assessed by Investigators or death.
To characterize the PK of M9140 (conjugated antibody, total antibody and unconjugated exatecan payload) in a [redacted] administration schedule as monotherapy and in combination regimen or Q3W in combination regimens.	PK parameters of M9140 conjugated antibody, total antibody, and unconjugated exatecan using noncompartmental analysis, e.g., AUC_{0-t}, AUC_{τ}, C_{max}, C_{trough}, Cl, V_d, $t_{½}$
To characterize the immunogenicity of M9140	ADA against M9140: occurrence and titer, as measured by ADA assay.

5-FU: 5-fluorouracil; ADA: anti-drug-antibodies, AE: adverse event, CEACAM5: Carcinoembryonic Antigen-Related Cell Adhesion Molecule 5, CR: complete response, CRC: colorectal cancer, DC: disease control, DoR: duration of response, OR: objective response, PFS: progression-free survival, PD: progressive disease, PK: pharmacokinetics, PR: partial response, RECIST: Response Evaluation Criteria in Solid Tumors, RP2D: recommended Phase 2 dose, SD: stable disease.

Overall Design:

This is a Phase 1, open-label, first-in-human (FIH), 2-part (dose escalation and expansion), noncontrolled, multicenter clinical study designed to determine the safety, tolerability, PK, and early signs of clinical activity of M9140. Part 1 (dose escalation) is aimed at determining the MTD and/or RDE of M9140 monotherapy (Part 1A) and of M9140 monotherapy with pegfilgrastim prophylaxis (Part 1B) based on safety, tolerability, PK, and preliminary clinical activity of M9140 as a single agent in participants with locally advanced or metastatic CRC for whom no effective standard therapy exists. Following Part 1, early clinical activity, safety, tolerability, and PK of M9140 will be further investigated in Part 2 of the study in participants with locally advanced or metastatic CRC.

- Part 2A will randomize participants, in a 1:1 ratio, between two M9140 doses (2.8 mg/kg and 2.4 mg/kg, every 3 weeks; Arm A1 and Arm A2, respectively).
- Part 2B will study M9140 administered CCI [redacted].
- Part 2C will study M9140 administered every 3 weeks in combination with bevacizumab (Part 2C1) and in combination with bevacizumab plus capecitabine (Part 2C2).
- Part 2D will study M9140 administered CCI [redacted] in combination with 5-FU plus bevacizumab.

Brief Summary:

The purpose of this first-in-human study is to evaluate the safety, tolerability, pharmacokinetics, and preliminary clinical activity of M9140 in participants with locally advanced or metastatic colorectal cancer. Study details include:

- Study Duration (per participant): Approximately 4 months for Part 1 and 8 months for Part 2.
- Visit Frequency: Approximately every 3 weeks (Parts 1, 2A and 2C) and CCI [redacted] for Parts 2B and 2D.
- M9140 is not available through an expanded access program.

Number of Participants:

The total number of participants in Part 1 will depend on the number of cohorts to be evaluated. It is anticipated that approximately 21 to 30 participants (5 projected dose levels with 3 to 9 participants each) will be exposed to the study intervention in Part 1A (cohorts without pegfilgrastim prophylaxis) and 9 to 15 participants (3 projected dose levels with 3 to 9 participants each) in Part 1B (cohorts with pegfilgrastim prophylaxis).

Part 2 will include approximately 135 to 170 participants. Part 2A will randomize 60 participants to 2.8 mg/kg (Arm A1) and 2.4 mg/kg (Arm A2) (30 participants in each). Approximately 20 to 30 participants will be enrolled in Part 2B and approximately 35 to 50 participants in Part 2C (approximately 15 to 20 in Part 2C1 and approximately 20 to 30 in Part 2C2). Approximately 20 to 30 participants will be enrolled in Part 2D.

Study Intervention Groups and Duration:

Study duration per participant is on an average approximately 4 months for Part 1 and 8 months for Part 2 (without Survival Follow-up). This includes a 28-day Screening period, infusion (approximately 1 hour) on Day 1 of every cycle, and Safety Follow-up Visit 30 (±3) days after the last dose of M9140. Furthermore, for Part 2A only, Survival Follow-up will be conducted every 3 months (± 2 weeks) until End of Study or up to 24 months after first dose, whichever comes first.

Involvement of Special Committee: Yes, Safety Monitoring Committee.

1.2 Schema

The overall study design is presented in Figure 1. Detailed schema for Part 2C is presented in Figure 2 and for Part 2D in Figure 3.

Figure 1 Overall Study Design

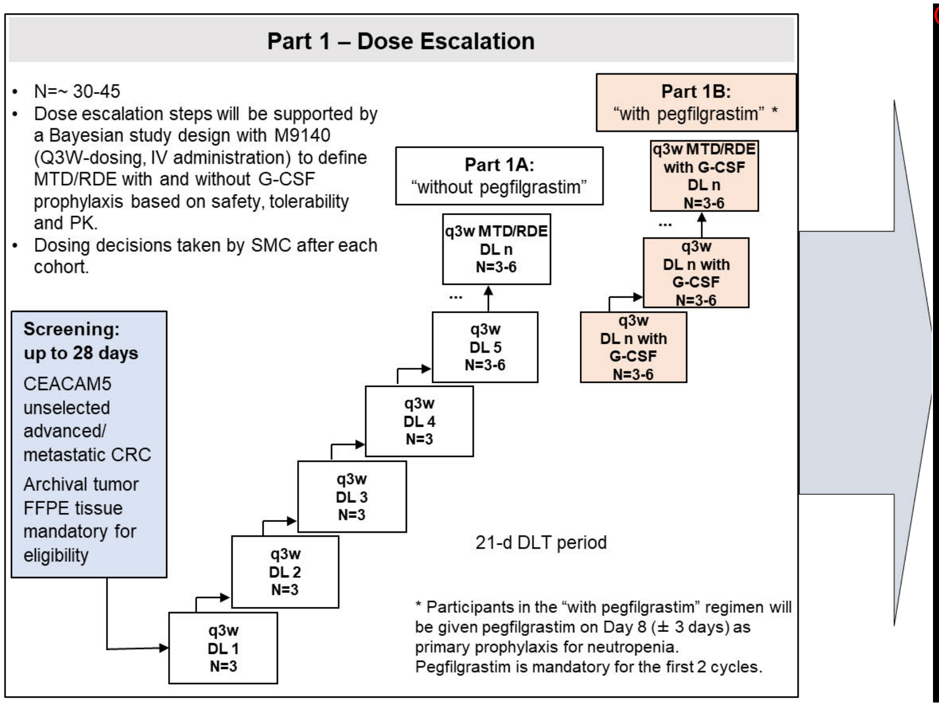

[redacted]

3L: 3rd line, CEACAM5: carcinoembryonic antigen-related cell adhesion molecule 5, [redacted], d: days, DL: dose level, DLT: dose-limiting toxicity, FFPE: formalin-fixed paraffin-embedded, iv: intravenous, MTD: maximum tolerated dose, N: number of participants, PK: pharmacokinetics, [redacted], Q3W: every 3 weeks, RDE: recommended dose for expansion, SMC: Safety Monitoring Committee.

Figure 2 **Overall Study Design for Part 2C – M9140 Q3W in Combination with Bevacizumab or Bevacizumab plus Capecitabine**

Part 2C – Planned Dose Levels

CCI [redacted]

Figure 3 Overall Study Design for Part 2D – M9140 CCI [redacted] in Combination with 5-FU plus Bevacizumab

Part 2D - Planned Dose Levels

CCI [redacted]

1.3 Schedule of Activities

1.3.1 Part 1 – M9140 Q3W Dose Escalation

Table 1 Schedule of Activities for Part 1A and Part 1B – Dose Escalation (Q3W)

Assessments & Procedures	Screening	Part 1: M9140 Dose Escalation Intervention Period (1 Cycle=21 Days)													End of M9140 Treatment (EOT)	Safety Follow-up/ Discontinuation	Notes
	Day -28	[redacted]													Within 7 days after decision of treatment discontinuation	30 days after last dose	
	Day																
	Visit Window (±h)														32	72	In case of bank holidays, a wider visit window is allowed.
Informed Consent	X																
Inclusion and Exclusion Criteria	X	X															Recheck clinical status before first dose of study intervention.
Study Intervention **M9140**		X					X			X		X		X			M9140 dosing is every 3 weeks (Q3W).
Pegfilgrastim administration					X			X			(X)		(X)				Applies to participants in Part 1B only. ± 3 days window is allowed.
Demography	X																
Physical examination	X	X			X	X	X	X		X	X	X	X	X	X		48-h window allowed prior to C1D1.
Past & Current Medical History (includes substance usage)	X																Substances: Drugs, alcohol, caffeine, tobacco usage.
Prior anticancer therapies	X																

Assessments & Procedures	Screening	Part 1: M9140 Dose Escalation Intervention Period (1 Cycle=21 Days)													End of M9140 Treatment (EOT)	Safety Follow-up/ Discontinuation	Notes
	Day -28	[redacted]													Within 7 days after decision of treatment discontinuation	30 days after last dose	
	Day																
	Visit Window (±h)														32	72	In case of bank holidays, a wider visit window is allowed.
Archival tumor tissue collection	X																Availability of archival tumor material is required. If no archival tumor tissue is available, it can be substituted with a fresh biopsy sample. [redacted]
Pregnancy test (WOCBP only)	X	X					X			X		X		X	X	X	Serum only at Screening and urine or serum on all other visits is allowed.
HIV, Hepatitis B and C screening	X																HIV: optional unless locally required.
Clinical Laboratory Tests (hematology, serum chemistry, including coagulation)	X	X			X	X	X	X	X	X	X	X	X	X	X	X	72-h window allowed prior to C1D1. Hematology includes reticulocyte count besides routine CBC. See Appendix 6 for details. On C2D15 only CBC is needed. C2D15 visit may be performed at participant's local physician's practice. Results need to be reported to the Principal Investigator for review and entered in the eCRF.
Iron deficiency (ferritin, TSAT) testing	X																

Assessments & Procedures	Screening	Part 1: M9140 Dose Escalation Intervention Period (1 Cycle=21 Days)													End of M9140 Treatment (EOT)	Safety Follow-up/ Discontinuation	Notes
	Day -28<br>Day	CCI [redacted]													Within 7 days after decision of treatment discontinuation	30 days after last dose	
	Visit Window (±h)														32	72	In case of bank holidays, a wider visit window is allowed.
Routine urinalysis	X	X					X			X		X		X	X		72-h window allowed prior to C1D1. Local urinalysis per dipstick testing and microscopic examination if blood or protein abnormality, if locally applicable based on institutional guidelines (see Appendix 6).
ECOG Performance Status	X	X					X			X		X		X	X	X	48-h window allowed prior to C1D1.
Vital signs	X	X	X	X	X	X	X	X		X	X	X	X	X	X	X	Weight and BMI will be assessed on D1 of each cycle. Height is only assessed at Screening (see Section 8.2.2 for details).
12-lead Safety ECG	X	X			X	X	X			X		X		X	X	X	Safety ECGs to be performed/read and interpreted locally (see Section 8.2.3 for details).

Assessments & Procedures	Screening	Part 1: M9140 Dose Escalation Intervention Period (1 Cycle=21 Days)													End of M9140 Treatment (EOT)	Safety Follow-up/ Discontinuation	Notes
	Day -28	[redacted]													Within 7 days after decision of treatment discontinuation	30 days after last dose	
	Day																
	Visit Window (±h)														32	72	In case of bank holidays, a wider visit window is allowed.
CT scan or MRI	X									X		X		X	X		At Screening and for response assessment via RECIST v1.1; tumors will be assessed every 6 weeks (±7 days) following the C1D1 visit. After the 3rd tumor scan (Screening, Evaluation 1, Evaluation 2) the following tumor assessments will be done every 9 weeks (±7 days). Tumor assessment at EOT if clinically applicable. See Section 8.1 for details.
Triplicate ECG for QTc evaluation	See Table 2 for details on collection timepoints.																
[redacted]																	
PK																	
ADA																	
Urine collection (M9140 renal clearance)																	
Subsequent anticancer therapies																X	
AE & SAE review	<===>																From the time of signing ICF to Safety Follow-Up Visit (See Section 8.3 and Section 6.8).
Concomitant medication and procedures review	<===>																

ADA: anti-drug antibodies, AE: adverse event, C: cycle, CBC: complete blood count, CT: computed tomography, D: day, EOT: end of treatment, ECG: electrocardiogram, ECOG: Eastern Cooperative Oncology Group, eCRF: electronic case report form, HIV: human immunodeficiency virus, ICF: informed consent form, MRI: magnetic resonance imaging, PK: pharmacokinetics, QTc: corrected QT interval, RECIST: Response Evaluation Criteria in Solid Tumors, SAE: serious adverse event, CCI [redacted], TSAT: transferrin saturation, WOCBP: Women of childbearing potential.

Table 2 Schedule of ECG, PK, [redacted], ADA Assessments, and Urine Collection – Part 1 (Dose Escalation Q3W)

Treatment Day	Time h (± h) [a,b]	Triplicate ECG for QTc evaluation[c]	PK M9140	[redacted]	ADA	Urine collection [d]	Notes
Screening							
[redacted]		X	X		X		[a] At visits where assessment time points (vital signs, ECG, and PK) coincide with each other:<br>1. Perform vital signs assessments first<br>2. ECG assessments slightly before the specific collection time point and<br>3. PK assessments at scheduled collection time point<br>[b] Actual collection times should be recorded in eCRF along with the times of start and EOI. All timepoints are based on the start of infusion.<br>[c] Triplicate digital acquisition of ECG after a 10-min rest and within 2 min. ECG to be taken before any blood sampling. QTc evaluation requires central evaluation (see Section 8.2.3 for details).<br>[d] Urine collection between start of infusion and 6 h after start of infusion on C1D1. On C1D2-C1D15, collect urine upon arrival and record the time of last urination prior to arrival at the clinic, starting from DL3 and through the projected DLs (i.e., DL4, DL5, DLn). Urine volume and collection time needs to be recorded in the eCRF. In case of hospitalization (expected for sites in Japan, see Appendix 11), collect all urine during the days specified in the schedule and record collection times during the stay in the clinic.
		X	X				
		X	X			X	
		X	X			X	
		X	X			X	
		X	X			X	
			X		X	X	
			X		X		
		X	X		X		
		X	X				
		X	X				
			X				
			X		X		PK, [redacted], and ADA sampling at predose every 2 cycles from C4 until treatment discontinuation (no samples are needed at the EOT visit).

ADA: anti-drug antibodies, C: cycle, D: day, DL: dose-escalation level, eCRF: electronic case report form, EOI: end of infusion, EOT: end of treatment, ECG: electrocardiogram, PK: Pharmacokinetics, QTc: corrected QT interval.

1.3.2 Part 2A – M9140 Q3W Dose Optimization

Table 3 Schedule of Activities for Part 2A– Dose Optimization (Q3W)

Assessments & Procedures	Screening	PART 2A: Dose Optimization Q3W Intervention Period (1 Cycle=21 Days)											End of M9140 Treatment (EOT)	Safety Follow-up/ Discontinuation	Survival Follow-up	Notes
	Day -28															
	CCI [redacted]												Within 7 days after decision of treatment discontinuation	30 days after last dose	Every 90 days until EOS or 24 months post First Dose	*C1D2 visit is applicable only for N ≈ 12 participants per arm (i.e., N ≈ 24 in total) See also Table 4.
	Visit Window (± h)	-	-6/+36	32	32	32	32	32	32	72	72	72	32	72	14 days	In case of bank holidays, a wider visit window is allowed.
Informed Consent	X															
Inclusion and Exclusion Criteria	X	X														Recheck clinical status before first dose of study intervention.
Study Intervention **M9140**		X				X		X		X		X				Dosing is every 3 weeks (Q3W).
Demography	X															
Physical examination	X	X			X	X		X		X		X	X			48-h window allowed prior to C1D1.
Past & Current Medical History	X															
Prior Anticancer Therapies	X															

Assessments & Procedures	Screening Day -28	PART 2A: Dose Optimization Q3W Intervention Period (1 Cycle=21 Days)											End of M9140 Treatment (EOT)	Safety Follow-up/ Discontinuation	Survival Follow-up	Notes
	CCI [redacted]												Within 7 days after decision of treatment discontinuation	30 days after last dose	Every 90 days until EOS or 24 months post First Dose	*C1D2 visit is applicable only for N ≈ 12 participants per arm (i.e., N ≈ 24 in total) See also Table 4.
	Visit Window (± h)	-	-6/+36	32	32	32	32	32	32	72	72	72	32	72	14 days	In case of bank holidays, a wider visit window is allowed.
Archival Tumor Tissue Collection	X															Availability of archival tumor material is required. If no archival tumor tissue is available, it can be substituted with a fresh biopsy sample. CCI [redacted]
Optional Fresh Tumor Biopsy	X															Sample acquisition should be conducted after participant's study eligibility has been confirmed and before participant receives the first dose.
Pregnancy Test (WOCBP only)	X	X				X		X		X		X	X	X		Serum only at Screening and urine or serum on all other visits is allowed.

Assessments & Procedures	Screening	PART 2A: Dose Optimization Q3W Intervention Period (1 Cycle=21 Days)											End of M9140 Treatment (EOT)	Safety Follow-up/ Discontinuation	Survival Follow-up	Notes
	Day -28															
	CCI [redacted]												Within 7 days after decision of treatment discontinuation	30 days after last dose	Every 90 days until EOS or 24 months post First Dose	*C1D2 visit is applicable only for N ≈ 12 participants per arm (i.e., N ≈ 24 in total) See also Table 4.
	Visit Window (± h)	-	-6/+36	32	32	32	32	32	32	72	72	72	32	72	14 days	In case of bank holidays, a wider visit window is allowed.
HIV, Hepatitis B and C Screening	X															HIV: optional unless locally required.
Clinical Laboratory Tests (Hematology, Serum Chemistry, including Coagulation)	X	X		X	X	X	X	X	X	X	X	X	X	X		At D15 hematology includes reticulocyte count besides routine CBC. See Appendix 6. 72-h window allowed prior to C1D1. Coagulation to be done only on D1 of each cycle, EOT and Safety Follow-up.
Iron deficiency testing (Ferritin, TSAT)	X							X		X*		X*				* Tests will be done on C3D1, C6D1, and C9D1.

Assessments & Procedures	Screening Day -28	PART 2A: Dose Optimization Q3W Intervention Period (1 Cycle=21 Days)											End of M9140 Treatment (EOT)	Safety Follow-up/ Discontinuation	Survival Follow-up	Notes
	[redacted]												Within 7 days after decision of treatment discontinuation	30 days after last dose	Every 90 days until EOS or 24 months post First Dose	*C1D2 visit is applicable only for N ≈ 12 participants per arm (i.e., N ≈ 24 in total) See also Table 4.
	Visit Window (± h)	-	-6/+36	32	32	32	32	32	32	72	72	72	32	72	14 days	In case of bank holidays, a wider visit window is allowed.
Routine urinalysis	X	X				X		X		X		X	X			72 h window allowed prior to C1D1. Local urinalysis per dipstick testing and microscopic examination if blood or protein abnormality, if locally applicable based on the institutional guidelines (see Appendix 6).
ECOG Performance Status	X	X				X		X		X		X	X	X		48-h window allowed prior to C1D1.
Vital signs	X	X		X	X	X	X	X	X	X	X	X	X	X		Weight and BMI will be assessed only on D1 of each cycle. Height is only assessed at Screening. See Section 8.2.2 for details.

Assessments & Procedures	Screening	PART 2A: Dose Optimization Q3W Intervention Period (1 Cycle=21 Days)											End of M9140 Treatment (EOT)	Safety Follow-up/ Discontinuation	Survival Follow-up	Notes
	Day -28 CCI [redacted]												Within 7 days after decision of treatment discontinuation	30 days after last dose	Every 90 days until EOS or 24 months post First Dose	*C1D2 visit is applicable only for N ≈ 12 participants per arm (i.e., N ≈ 24 in total) See also Table 4.
	Visit Window (± h)	-	-6/+36	32	32	32	32	32	32	72	72	72	32	72	14 days	In case of bank holidays, a wider visit window is allowed.
12-lead Safety ECG	X	X						X		X		X	X			Safety ECGs to be performed, read, and interpreted locally on Day 1 of every other cycle starting on C1D1. See Section 8.2.3 for details.

Assessments & Procedures	Screening Day -28	PART 2A: Dose Optimization Q3W Intervention Period (1 Cycle=21 Days)											End of M9140 Treatment (EOT)	Safety Follow-up/ Discontinuation	Survival Follow-up	Notes
	CCI [redacted]												Within 7 days after decision of treatment discontinuation	30 days after last dose	Every 90 days until EOS or 24 months post First Dose	*C1D2 visit is applicable only for N ≈ 12 participants per arm (i.e., N ≈ 24 in total) See also Table 4.
	Visit Window (± h)	-	-6/+36	32	32	32	32	32	32	72	72	72	32	72	14 days	In case of bank holidays, a wider visit window is allowed.
CT Scan or MRI	X							X		X		X	X			At Screening and for response assessment via RECIST v1.1; tumors will be assessed every 6 weeks (± 7 days) following the C1D1 Visit. After the 4th tumor scan (Screening, Evaluation 1 and Evaluation 2, Evaluation 3) the following tumor assessments will be done every 12 weeks (± 7 days) until disease progression or start of new anti-cancer therapy. Tumor assessment at EOT if clinically applicable (see Section 8.1 for details).

Assessments & Procedures	Screening Day -28	PART 2A: Dose Optimization Q3W Intervention Period (1 Cycle=21 Days)											End of M9140 Treatment (EOT)	Safety Follow-up/ Discontinuation	Survival Follow-up	Notes
	CCI [redacted]												Within 7 days after decision of treatment discontinuation	30 days after last dose	Every 90 days until EOS or 24 months post First Dose	*C1D2 visit is applicable only for N ≈ 12 participants per arm (i.e., N ≈ 24 in total) See also Table 4.
	Visit Window (± h)	-	-6/+36	32	32	32	32	32	32	72	72	72	32	72	14 days	In case of bank holidays, a wider visit window is allowed.
CCI [redacted]																
Triplicate ECG for QTc evaluation	See Table 4 for details on collection timepoints.															
CCI [redacted]																CCI [redacted]
PK																
ADA																
Patient reported symptomatic adverse events (PRO-CTCAE)		X		X	X	X	X	X	X	X	X					To be completed before dosing on D1 (see Appendix 12).
Survival Follow-up															X	Follow-up for survival every 90 ± 14 days (e.g., by phone) until EOS or up to 24 months after the first dose. See also Section 4.4.
Subsequent Anticancer Therapies													X	X	X	

Assessments & Procedures	Screening Day -28	PART 2A: Dose Optimization Q3W Intervention Period (1 Cycle=21 Days)											End of M9140 Treatment (EOT)	Safety Follow-up/ Discontinuation	Survival Follow-up	Notes
	[redacted]												Within 7 days after decision of treatment discontinuation	30 days after last dose	Every 90 days until EOS or 24 months post First Dose	*C1D2 visit is applicable only for N ≈ 12 participants per arm (i.e., N ≈ 24 in total) See also Table 4.
	Visit Window (± h)	-	-6/+36	32	32	32	32	32	32	72	72	72	32	72	14 days	In case of bank holidays, a wider visit window is allowed.
AE & SAE review	<==>															From the time of signing ICF to Safety Follow-Up Visit.
Concomitant medication and procedures review	<==>															

ADA: anti-drug antibodies, AE: adverse event, C: cycle, [redacted], CT: computed tomography, D: day, EOS: end of study, EOT: end of treatment, ECG: electrocardiogram, ECOG: Eastern Cooperative Oncology Group, HIV: human immunodeficiency virus, ICF: informed consent form, MRI: magnetic resonance imaging, PK: pharmacokinetics, QTc: corrected QT interval, RECIST: Response Evaluation Criteria in Solid Tumors, SAE: serious adverse event, [redacted], TSAT: transferrin saturation, WOCBP: Women of childbearing potential.

Table 4 Schedule of ECG, PK, [redacted], and ADA Assessments during Part 2A (M9140 Dose Optimization Q3W)

	PART 2A					
**Treatment Day**	**Time h (± h) [a,b]**	**Triplicate ECG for QTc evaluation[c]**	**PK M9140**	[redacted]	**ADA**	**Notes**
[redacted]	[redacted]			[redacted]		
		X	X		X	[a] At visits where assessment time points (vital signs, ECG, and PK) coincide with each other:<br>• Perform vital signs assessments first<br>• ECG assessments slightly before the specific collection timepoint and<br>• PK assessments at scheduled collection timepoint<br>[b] Actual collection times should be recorded in the eCRF along with the times of start and EOI. All timepoints are based on the start of M9140 infusion.<br>[c] Triplicate, digital acquisition of ECG after a 10-min rest and within 2 min. ECG to be taken before any blood sampling. QTc evaluation requires central evaluation (see Section 8.2.3 for details). Triplicate ECG will be performed only for approximately 24 participants (n≈12 for each Arm A1 and Arm A2)<br>[d] C1D2 visit will be performed only for the same approximately 24 participants (n≈12 for each Arm A1 and Arm A2) mentioned above.
		X	X			
		X	X			
		X	X			
		X	X			
		X	X			
			X		X	
		X	X		X	
		X	X			
		X	X			
			X			
			X		X	
			X		X	Samples are needed at the EOT visit for M9140 PK, ADA, and biomarkers.

ADA: anti-drug antibodies, C: cycle, D: day, ECG: electrocardiogram, eCRF: electronic case report form, EOI: end of infusion, EOT: end of treatment, PK: Pharmacokinetics, QTc: corrected QT interval.

1.3.3 Part 2B – M9140 [redacted] Regimen

Table 5 Schedule of Activities for Part 2B – M9140 [redacted] regimen

Assessments & Procedures	Screening Day -28	Part 2B – M9140 [redacted] Intervention Period ([redacted])									End of M9140 Treatment (EOT)	Safety Follow-up/ Discontinuation	Notes
	[redacted]										Within 7 days after decision of treatment discontinuation	30 days after last dose	
	Visit Window (± h)	-	32	+32	32	32	32	72	72	72	32	72	In case of bank holidays, a wider visit window is allowed.
Informed Consent	X												
Inclusion and Exclusion Criteria	X	X											Recheck clinical status before first dose of study intervention.
[redacted]													
Demography	X												
Physical examination	X	X	X	X	X	X	X	X	X	X	X		48-hour window allowed prior to C1D1.
Past & Current Medical History	X												
Prior Anticancer Therapy	X												
Archival Tumor Tissue Collection	X												Availability of archival tumor material is required. If no archival tumor tissue is available, it can be substituted with a fresh biopsy sample. [redacted]
Pregnancy Test (WOCBP only)	X	X		X		X		X		X	X	X	Serum only at Screening and urine or serum on all other visits is allowed.
HIV, Hepatitis B, and C Screening	X												HIV: optional unless locally required.

Assessments & Procedures	Screening Day -28	Part 2B – M9140 [redacted] Intervention Period ([redacted])									End of M9140 Treatment (EOT)	Safety Follow-up/ Discontinuation	Notes
	[redacted]										Within 7 days after decision of treatment discontinuation	30 days after last dose	
	Visit Window (± h)	-	32	+32	32	32	32	72	72	72	32	72	In case of bank holidays, a wider visit window is allowed.
Clinical Laboratory Tests (hematology, serum chemistry, including coagulation)	X	X	X	X	X	X	X	X	X	X	X	X	At D8 hematology includes reticulocyte count besides routine CBC. See Appendix 6 for details. 72-h window allowed prior to C1D1. Coagulation to be done only on D1 of each cycle, EOT and Safety Follow-up.
Iron deficiency (Ferritin, TSAT) testing	X					X		X*		X*			* Tests to be done on C3D1, C6D1, and C9D1.
Routine urinalysis	X	X		X		X		X		X	X		72-h window allowed prior to C1D1. Local urinalysis per dipstick testing and microscopic examination if blood or protein abnormality, if locally applicable based on the institutional guidelines (see Appendix 6).
ECOG Performance Status	X	X		X		X		X		X	X	X	48-h window allowed prior to C1D1.
Vital signs	X	X	X	X	X	X	X	X	X	X	X	X	Weight and BMI will be assessed only on D1 of each cycle. Height is only assessed at Screening (see Section 8.2.2 for details).
12-lead Safety ECG	X	X				X		X		X	X		Safety ECGs to be performed, read, and interpreted locally on D1 of every other cycle starting on C1D1 (see Section 8.2.3 for details).

Assessments & Procedures	Screening Day -28	Part 2B – M9140 [redacted] Intervention Period ([redacted])									End of M9140 Treatment (EOT)	Safety Follow-up/ Discontinuation	Notes
	[redacted]										Within 7 days after decision of treatment discontinuation	30 days after last dose	
	Visit Window (± h)	-	32	+32	32	32	32	72	72	72	32	72	In case of bank holidays, a wider visit window is allowed.
CT Scan or MRI	X							X		X	X		At Screening and for response assessment via RECIST v1.1; tumors will be assessed every 6 weeks (± 7 days) following C1D1 visit. After the 4th tumor scan (Screening, Evaluation 1, Evaluation 2, Evaluation 3) the following tumor assessments will be done every 12 weeks (± 7 days). Tumor assessment at EOT if clinically applicable. See Section 8.1 for details.
[redacted]													
[redacted]	See Table 6 for details on collection timepoints												[redacted]
PK													
ADA													
Subsequent Anticancer Therapy											X	X	
AE & SAE review	< == >												
Concomitant medication and procedures review	< == >												From the time of signing ICF to Safety Follow-Up Visit.

ADA: anti-drug antibodies, AE: adverse event, C: cycle, CBC: complete blood count, CT: computed tomography, D: day, EOI: end of infusion, EOT: end of treatment, HIV: human immunodeficiency virus, ECG: electrocardiogram, ECOG: Eastern Cooperative of Oncology Group, eCRF: electronic case report form, FFPE: formalin-fixed paraffin-embedded, ICF: informed consent form, MRI: magnetic resonance imaging, PK: Pharmacokinetics, QTc: corrected QT interval, RECIST: Response Evaluation Criteria in Solid Tumors, SAE: serious adverse event, [redacted], WOCBP: Women of childbearing potential.

Table 6 Schedule of PK, [redacted], and ADA Assessments – Part 2B M9140 [redacted] regimen

PART 2B: M9140 [redacted] Regimen					
**Treatment Day**	**Time h [a,b]**	**PK M9140**	[redacted]	**ADA**	**Notes**
[redacted]					[a] At visits where assessment time points (PK, and vital signs) coincide with each other:<br>• Perform vital signs assessments first<br>• ECG assessments slightly before the specific collection timepoint and<br>• PK assessments at scheduled collection timepoint<br>[b] Actual collection times should be collected in the eCRF along with the times of start and EOI. All timepoints are based on the start of infusion.
		X		X	
		X			
		X			
		X			
		X		X	
		X		X	
		X			
		X			
		X			
		X		X	
		X		X	Samples are needed at the EOT visit for M9140 PK, ADA, and [redacted].

ADA: antidrug antibodies, C: cycle, D: day, ECG: electrocardiogram, eCRF: electronic case report form, EOI: end of infusion, EOT: end of treatment, PK: Pharmacokinetics.

1.3.4 Part 2C – M9140 Q3W in Combination with Bevacizumab or Bevacizumab plus Capecitabine

Table 7 Schedule of Activities for Part 2C – M9140 Q3W in Combination with Bevacizumab or Bevacizumab plus Capecitabine

Assessments & Procedures	Screening Day -28	Part 2C – M9140 Q3W in Combination with Bevacizumab or Bevacizumab plus Capecitabine Intervention Period (1 Cycle=21 Days)													End of M9140 Treatment (EOT)	Safety Follow-up/ Discontinuation	Notes
	[redacted]														Within 7 days after decision of treatment discontinuation	30 days after last dose	*C1D2 visit will be performed only for ≈24 participants (n≈12 for each Part C1 and Part C2)
	Visit Window (± h)	-	-6/+36	32	32	+32	32	32	32	32	32	72	72	72	32	72	In case of bank holidays, a wider visit window is allowed.
Informed Consent	X																
Inclusion and Exclusion Criteria	X	X															Recheck clinical status before first dose of study intervention.
Study Intervention **M9140** (+ bevacizumab in Part 2C1; +bevacizumab and capecitabine in Part 2C2)		X				X			X			X		X			Dosing is every 3 weeks (Q3W). For capecitabine dosing, see Section 6.1
Demography	X																
Physical examination	X	X		X	X	X			X			X		X	X		48-h window allowed prior to C1D1.
Past & Current Medical History	X																
Prior Anticancer Therapies	X																

Assessments & Procedures	Screening Day -28	Part 2C – M9140 Q3W in Combination with Bevacizumab or Bevacizumab plus Capecitabine Intervention Period (1 Cycle=21 Days)													End of M9140 Treatment (EOT)	Safety Follow-up/ Discontinuation	Notes
	CCI [redacted]														Within 7 days after decision of treatment discontinuation	30 days after last dose	*C1D2 visit will be performed only for ≈24 participants (n≈12 for each Part C1 and Part C2)
	Visit Window (± h)	-	-6/+36	32	32	+32	32	32	32	32	32	72	72	72	32	72	In case of bank holidays, a wider visit window is allowed.
Archival Tumor Tissue Collection	X																Availability of archival tumor material is required. If no archival tumor tissue is available, it can be substituted with a fresh biopsy sample. CCI [redacted]
Optional Fresh Tumor Biopsy	X																Sample acquisition should be conducted after participant's study eligibility has been confirmed and before participant receives the first dose.
Pregnancy Test (WOCBP only)	X	X				X			X			X		X	X	X	Serum only at Screening and urine or serum on all other visits is allowed.
HIV, Hepatitis B, and C Screening	X																HIV: optional unless locally required.

Assessments & Procedures	Screening Day -28	Part 2C – M9140 Q3W in Combination with Bevacizumab or Bevacizumab plus Capecitabine Intervention Period (1 Cycle=21 Days)													End of M9140 Treatment (EOT)	Safety Follow-up/ Discontinuation	Notes
	[redacted]														Within 7 days after decision of treatment discontinuation	30 days after last dose	*C1D2 visit will be performed only for ≈24 participants (n≈12 for each Part C1 and Part C2)
	Visit Window (± h)	-	-6/+36	32	32	+32	32	32	32	32	32	72	72	72	32	72	In case of bank holidays, a wider visit window is allowed.
Clinical Laboratory Tests (Hematology, Serum Chemistry, including Coagulation)	X	X		X	X	X	X	X	X	X	X	X	X	X	X	X	At D15 hematology includes reticulocyte count besides routine CBC. See Appendix 6. 72-h window allowed prior to C1D1. Coagulation to be done only on D1 of each cycle, EOT and Safety Follow-up.
Iron deficiency testing (Ferritin, TSAT)	X								X			X*		X*			* Tests to be done on C3D1, C6D1, and C9D1

**Assessments & Procedures**	**Screening Day -28**	**Part 2C – M9140 Q3W in Combination with Bevacizumab or Bevacizumab plus Capecitabine Intervention Period (1 Cycle=21 Days)**													**End of M9140 Treatment (EOT)**	**Safety Follow-up/ Discontinuation**	**Notes**
	[redacted]														**Within 7 days after decision of treatment discontinuation**	**30 days after last dose**	*C1D2 visit will be performed only for ≈24 participants (n≈12 for each Part C1 and Part C2)
	**Visit Window (± h)**	**-**	**-6/+36**	**32**	**32**	**+32**	**32**	**32**	**32**	**32**	**32**	**72**	**72**	**72**	**32**	**72**	In case of bank holidays, a wider visit window is allowed.
Routine urinalysis	X	X				X			X			X		X	X		72-h window allowed prior to C1D1. Local urinalysis per dipstick testing and microscopic examination if blood or protein abnormality, if locally applicable based on the institutional guidelines. If dipstick proteinuria ≥ 2+, a 24-hour urine collection is required for quantitative assessment of proteinuria (see Appendix 6).
ECOG Performance Status	X	X				X			X			X		X	X	X	48-h window allowed prior to C1D1.
Vital signs	X	X		X	X	X	X	X	X	X	X	X	X	X	X	X	Weight and BMI will be assessed only on D1 of each cycle. BSA will be assessed on D1 of each cycle (Part 2C2 only). Height is only assessed at Screening. See Section 8.2.2 for details.

Assessments & Procedures	Screening Day -28	Part 2C – M9140 Q3W in Combination with Bevacizumab or Bevacizumab plus Capecitabine Intervention Period (1 Cycle=21 Days)													End of M9140 Treatment (EOT)	Safety Follow-up/ Discontinuation	Notes
	[redacted]														Within 7 days after decision of treatment discontinuation	30 days after last dose	*C1D2 visit will be performed only for ≈24 participants (n≈12 for each Part C1 and Part C2)
	Visit Window (± h)	-	-6/+36	32	32	+32	32	32	32	32	32	72	72	72	32	72	In case of bank holidays, a wider visit window is allowed.
12-lead Safety ECG	X	X							X			X		X	X		Safety ECGs to be performed/read and interpreted locally on D1 of every other cycle starting on C1D1. See Section 8.2.3 for details.
CT Scan or MRI	X								X			X		X	X		At Screening and for response assessment via RECIST v1.1; tumors will be assessed every 6 weeks (± 7 days) following the C1D1 Visit. After the 4th tumor scan (Screening, Evaluation 1 and Evaluation 2, Evaluation 3) the following tumor assessments will be done every12 weeks (± 7 days). Tumor assessment at EOT if clinically applicable (see Section 8.1 for details).

Assessments & Procedures	Screening Day -28	Part 2C – M9140 Q3W in Combination with Bevacizumab or Bevacizumab plus Capecitabine Intervention Period (1 Cycle=21 Days)													End of M9140 Treatment (EOT)	Safety Follow-up/ Discontinuation	Notes
	[redacted]														Within 7 days after decision of treatment discontinuation	30 days after last dose	*C1D2 visit will be performed only for ≈24 participants (n≈12 for each Part C1 and Part C2)
	Visit Window (± h)	-	-6/+36	32	32	+32	32	32	32	32	32	72	72	72	32	72	In case of bank holidays, a wider visit window is
[redacted]																	
[redacted]	See Table 8 for details on collection timepoints.																[redacted]
PK																	
ADA																	
Subsequent Anticancer Therapies															X	X	
AE & SAE review	< === >																From the time of signing ICF to Safety Follow-Up Visit.
Concomitant medication and procedures review	< === >																

ADA: anti-drug antibodies, AE: adverse event, BMI: body mass index, BSA: body surface area, C: cycle, [redacted], CT: computed tomography, D: day, EOS: end of study, EOT: end of treatment, ECG: electrocardiogram, ECOG: Eastern Cooperative Oncology Group, HIV: human immunodeficiency virus, ICF: informed consent form, MRI: magnetic resonance imaging, PK: pharmacokinetics, QTc: corrected QT interval, RECIST: Response Evaluation Criteria in Solid Tumors, SAE: serious adverse event, [redacted], TSAT: transferrin saturation, WOCBP: Women of childbearing potential.

Table 8 Schedule of PK, CCI [redacted], and ADA Assessments during Part 2C M9140 Q3W in Combination with Bevacizumab or Bevacizumab plus Capecitabine

Part 2C: M9140 Q3W in Combination with Bevacizumab or Bevacizumab plus Capecitabine					
**Treatment Day**	**Time h (± h)** [a,b,c]	**PK M9140**	CCI [redacted]	**ADA**[g]	**Notes**
CCI [redacted]					
		X[e,f]		X	[a] At visits where assessment time points (vital signs, and PK) coincide with each other:<br>• Perform vital signs assessments first<br>• ECG assessments slightly before the specific collection timepoint and<br>• PK assessments at scheduled collection timepoint<br>[b] Actual collection times should be recorded in the eCRF along with the times of start and EOI.<br>[c] All times refer to M9140 administration and are based on the start of M9140 infusion.<br>[d] It is recommended to complete PK sampling in the morning<br>[e] Bevacizumab concentrations to be measured in addition to M9140<br>[f] Part 2C2: Capecitabine and 5-FU concentrations to be measured in addition to M9140<br>[g] only ADA against M9140 will be measured<br>[h] C1D2 visit will be performed only for approximately 24 participants (n≈12 for each Part C1 and Part C2)
		X			
		X[e]			
		X[e,f]			
		X[e,f]			
		X[e,f]			
		X[e]		X	
		X[e]		X	
		X			
		X[e]			
		X[e]			
		X[e]		X	
		X[e]		X	Samples are needed at the EOT visit for M9140 PK, ADA, CCI [redacted].

ADA: anti-drug antibodies, C: cycle, D: day, ECG: electrocardiogram, eCRF: electronic case report form, EOI: end of infusion, EOT: end of treatment, PK: Pharmacokinetics.

1.3.5 Part 2D – M9140 [redacted] in Combination with 5-FU plus Bevacizumab

Table 9 Schedule of Activities for Part 2D – M9140 [redacted] in Combination with 5-FU plus Bevacizumab

Assessments & Procedures	Screening Day -28	Part 2D – M9140 [redacted] in Combination with 5-FU plus Bevacizumab Intervention Period ([redacted])									End of M9140 Treatment (EOT)	Safety Follow-up/ Discontinuation	Notes
	[redacted]										Within 7 days after decision of treatment discontinuation	30 days after last dose	
	Visit Window (± h)	-	32	+32	32	32	32	72	72	72	32	72	In case of bank holidays, a wider visit window is allowed.
Informed Consent	X												
Inclusion and Exclusion Criteria	X	X											Recheck clinical status before first dose of study intervention.
[redacted]													
Demography	X												
Physical examination	X	X	X	X	X	X	X	X	X	X	X		48-hour window allowed prior to C1D1.
Past & Current Medical History	X												
Prior Anticancer Therapy	X												

Assessments & Procedures	Screening Day -28	Part 2D – M9140 [redacted] in Combination with 5-FU plus Bevacizumab Intervention Period ([redacted])									End of M9140 Treatment (EOT)	Safety Follow-up/ Discontinuation	Notes
		[redacted]									Within 7 days after decision of treatment discontinuation	30 days after last dose	
	Visit Window (± h)	-	32	+32	32	32	32	72	72	72	32	72	In case of bank holidays, a wider visit window is allowed.
Archival Tumor Tissue Collection	X												Availability of archival tumor material is required. If no archival tumor tissue is available, it can be substituted with a fresh biopsy sample. [redacted]
Pregnancy Test (WOCBP only)	X	X		X		X		X		X	X	X	Serum only at Screening and urine or serum on all other visits is allowed.
HIV, Hepatitis B, and C Screening	X												HIV: optional unless locally required.

Assessments & Procedures	Screening Day -28	Part 2D – M9140 [redacted] in Combination with 5-FU plus Bevacizumab Intervention Period ([redacted])									End of M9140 Treatment (EOT)	Safety Follow-up/ Discontinuation	Notes
	[redacted]										Within 7 days after decision of treatment discontinuation	30 days after last dose	
	**Visit Window (± h)**	**-**	**32**	**+32**	**32**	**32**	**32**	**72**	**72**	**72**	**32**	**72**	In case of bank holidays, a wider visit window is allowed.
Clinical Laboratory Tests (hematology, serum chemistry, including coagulation)	X	X	X	X	X	X	X	X	X	X	X	X	At D8 hematology includes reticulocyte count besides routine CBC. See Appendix 6 for details. 72-h window allowed prior to C1D1. Coagulation to be done only on D1 of each cycle, EOT and Safety Follow-up.
Routine urinalysis	X	X		X		X		X		X	X		72-h window allowed prior to C1D1. Local urinalysis per dipstick testing and microscopic examination if blood or protein abnormality, if locally applicable based on the institutional guidelines. If dipstick proteinuria ≥ 2+, a 24-hour urine collection is required for quantitative assessment of proteinuria (see Appendix 6).
ECOG Performance Status	X	X		X		X		X		X	X	X	48-h window allowed prior to C1D1.
Vital signs	X	X	X	X	X	X	X	X	X	X	X	X	Weight, BMI, and BSA will be assessed only on D1 of each cycle. Height is only assessed at Screening (see Section 8.2.2 for details).

Assessments & Procedures	Screening Day -28	Part 2D – M9140 [redacted] in Combination with 5-FU plus Bevacizumab Intervention Period ([redacted])									End of M9140 Treatment (EOT)	Safety Follow-up/ Discontinuation	Notes
	[redacted]										Within 7 days after decision of treatment discontinuation	30 days after last dose	
	Visit Window (± h)	-	32	+32	32	32	32	72	72	72	32	72	In case of bank holidays, a wider visit window is allowed.
12-lead Safety ECG	X	X				X		X		X	X		Safety ECGs to be performed, read, and interpreted locally on D1 of every other cycle starting on C1D1 (see Section 8.2.3 for details).
CT Scan or MRI	X							X		X	X		At Screening and for response assessment via RECIST v1.1; tumors will be assessed every 6 weeks (± 7 days) following C1D1 visit. After the 4th tumor scan (Screening, Evaluation 1, Evaluation 2, Evaluation 3) the following tumor assessments will be done every 12 weeks (± 7 days). Tumor assessment at EOT if clinically applicable. See Section 8.1 for details.
[redacted]													
[redacted]	See Table 10 for details on collection timepoints												[redacted]
PK													
ADA													

**Assessments & Procedures**	**Screening Day -28**	**Part 2D – M9140 [redacted] in Combination with 5-FU plus Bevacizumab Intervention Period ([redacted])**									**End of M9140 Treatment (EOT)**	**Safety Follow-up/ Discontinuation**	**Notes**
	[redacted]										**Within 7 days after decision of treatment discontinuation**	**30 days after last dose**	
	**Visit Window (± h)**	**-**	**32**	**+32**	**32**	**32**	**32**	**72**	**72**	**72**	**32**	**72**	In case of bank holidays, a wider visit window is allowed.
Subsequent Anticancer Therapy											X	X	
AE & SAE review	< == >												
Concomitant medication and procedures review	< == >												From the time of signing ICF to Safety Follow-Up Visit.

5-FU: 5-fluorouracil, ADA: anti-drug antibodies, AE: adverse event, BMI: body mass index, BSA: body surface area, C: cycle, CBC: complete blood count, CT: computed tomography, [redacted], D: day, ECG: electrocardiogram, ECOG: Eastern Cooperative of Oncology Group, EOT: end of treatment, HIV: human immunodeficiency virus, ICF=informed consent form, MRI: magnetic resonance imaging, PK: pharmacokinetics, RECIST: Response Evaluation Criteria in Solid Tumors, SAE: serious adverse event, [redacted], WOCBP: Women of childbearing potential.

5-FU is administered via a portable pump as continuous infusion over 46 hours. Participants in Part 2D may be required to return to the investigational site for pump removal after completion of the 5-FU infusion, typically on D3 of each cycle, depending on local guidelines and practices. No study assessments will take place during this visit.

Table 10 Schedule of PK, [redacted], and ADA Assessments during Part 2D M9140 [redacted] in Combination with 5-FU plus Bevacizumab

PART 2D: M9140 [redacted] in Combination with 5-FU plus Bevacizumab					
**Treatment Day**	**Time h [a,b,c]**	**PK M9140**	[redacted]	**ADA[g]**	**Notes**
[redacted]					[a] At visits where assessment time points (PK, and vital signs) coincide with each other:<br>• Perform vital signs assessments first<br>• ECG assessments slightly before the specific collection timepoint and<br>• PK assessments at scheduled collection timepoint<br>[b] Actual collection times should be collected in the eCRF along with the times of start and EOI.<br>[c] All times refer to M9140 administration and are based on the start of M9140 infusion.<br>[d] Bevacizumab concentrations to be measured in addition to M9140<br>[e] 5-FU concentrations to be measured in addition to M9140.<br>[f] Sample to be taken after start of 5-FU infusion.<br>[g] Only ADA against M9140 will be measured
		$X^{d,e}$		X	
		X			
		$X^{d,e,f}$			
		X^{d}			
		X^{d}		X	
		X^{d}		X	
		X			
		$X^{d,e,f}$			
		X^{d}			
		X^{d}		X	
		X^{d}		X	Samples are needed at the EOT visit for M9140 PK, ADA, and [redacted].

ADA: antidrug antibodies, C: cycle, D: day, ECG: electrocardiogram, eCRF: electronic case report form, EOI: end of infusion, EOT: end of treatment, PK: Pharmacokinetics.

2 Introduction

M9140 is an ADC specific to CEACAM5 (also CEA or CD66) with the TOP1 inhibitor exatecan as payload. The molecule is a fully homogenous product comprising 8 exatecan molecules per antibody, linked via maleimide chemistry to the interchain cysteines.

M9140 is being developed for the treatment of participants with advanced/metastatic solid tumors.

Detailed information on the chemistry, pharmacology, efficacy, and safety of M9140 is in the IB.

2.1 Study Rationale

The administration of M9140 to participants with specified locally advanced or metastatic solid tumors, is justified for the following reasons:

- CEACAM5 is a well-known member of the CEACAM family. CEACAM5 has limited expression in adult normal tissues, but is expressed at high levels in various adenocarcinomas, particularly in CRC, GC, [redacted], NSCLC, and pancreatic cancers; therefore, it provides an attractive target for the development of ADCs, such as M9140.
- In PDX models, administration of M9140 demonstrated strong efficacy in CRC, GC, [redacted], and NSCLC tumors expressing CEACAM5.
- M9140 induced dose-dependent reversible AEs in the hematolymphoid and gastrointestinal systems of monkeys that resemble exatecan toxicity. These risks can be adequately managed with the proposed study design.
- Bystander effect observed with M9140 further enhances antitumor activity.
- Preliminary clinical data from the dose escalation Part 1 of the study have demonstrated a manageable safety profile consistent with exatecan toxicity with no new, unexpected AEs observed and encouraging preliminary antitumor activity of M9140 in heavily pretreated participants with colorectal cancer.

Overall, a positive benefit-risk ratio justifies the administration of M9140 iv either as monotherapy or in combination regimens in unselected participants with locally advanced or metastatic CRC.

2.2 Background

CEACAMs are a family of 12 immunoglobulin-related proteins physiologically expressed on many epithelial tissues, where they act as modulators of different processes such as cell adhesion, differentiation, proliferation, and survival (Tchoupa 2014). The link between CEACAMs and cancer was established many years ago with the identification of CEA (CEACAM5) as tumor biomarker and later with the description of CEACAM5's role in cancer progression and metastasis due to its own functions as an adhesion molecule or in association with signaling

receptors (Beauchemin 2013). CEACAM5 is highly expressed in CRC, GC/GEJC, and NSCLC tumors whereas its expression in normal tissue is limited (Decary 2020). CEACAM5 was demonstrated to be an independent prognostic factor for CRC and late-stage GC (Zhou 2015). Thus, CEACAM5 is an attractive target for antibody-based therapies designed to selectively deliver cytotoxic drugs to tumors expressing CEACAM5.

M9140 is a recombinant human immunoglobulin G1 (IgG1) monoclonal antibody [redacted], coupled to a potent TOP1i payload, exatecan, with a [redacted] drug-to-antibody ratio (DAR) of 8. M9140 has high specificity and binding affinity for CEACAM5+ tumors and induces tumor cell killing after lysosomal release of the payload into tumor cells. Furthermore, preclinical data have shown killing of bystander tumor cells that lack CEACAM5 expression.

The primary mechanism of action of M9140 is targeted killing of tumor cells expressing CEACAM5 by affecting DNA replication and transcription. M9140 belongs to a class of highly potent biopharmaceutical ADCs, designed as targeted therapy for the treatment of solid cancer. Upon CEACAM5 binding, the target-ADC complex is internalized into the cell and translocated to the lysosomal compartment where the linker is enzymatically cleaved and the cytotoxic substance exatecan is released from the ADC to induce tumor cell death. Exatecan is a camptothecin derivative that acts as an inhibitor of the enzyme TOP1, comparable to known chemotherapeutics like irinotecan and topotecan. TOP1 relaxes supercoiled DNA during replication and transcription by DNA nicking. TOP1 inhibitors are extensively used to treat a diverse range of cancers and act by trapping the TOP1-DNA cleavage complexes, which results in DNA damage during replication or transcription, thereby leading to tumor cell death (Pommier 2006; Mitsui 1995).

2.3 Benefit/Risk Assessment

More detailed information about the known and expected benefits and risks and reasonably expected adverse events of M9140 may be found in Section 4.2 and the IB.

Based on the available nonclinical and clinical data to date, the conduct of the study, as specified in this protocol, is considered justifiable.

2.3.1 Risk Assessment

Table 11 Identified and Potential Risks and Their Management (Data Cutoff Date 20-November-2023)

Identified and Potential Risks of Clinical Significance	Summary of Data/Rationale for Risk	Mitigation Strategy
**Study Intervention: M9140**		
**Identified Risks**		
**Hematologic toxicity** (e.g., anemia, neutropenia, leukopenia, febrile neutropenia and neutropenic infectious complications, thrombocytopenia, lymphopenia)	Toxicity studies in cynomolgus monkeys with M9140 showed changes of the hematolymphoid system (bone marrow, thymus, spleen, and lymph nodes) at/from 30 mg/kg and transient decrease of reticulocytes and neutrophils from 10 mg/kg, as well as RBC mass reduction (RBC, Ht, Hb) in females only from 24 mg/kg.<br>In oncology patients treated with exatecan (Rowinsky 2005, De Jager 2000), myelosuppression, particularly neutropenia, was the principal DLT. Anemia, thrombocytopenia, lymphopenia may also occur less frequently.<br>In Part 1 of the study (dose escalation), the most common hematologic Grade ≥3 TEAEs were neutrophil count decreased/neutropenia, anemia, white blood cell count decreased/leukopenia, and platelet count decreased/thrombocytopenia.	• Inclusion criteria: adequate hematologic function.<br>• BMI cap-based dosing to protect obese participants from excessive exposure and potential increased risk for treatment related adverse events.<br>• Hematology labs at baseline and monitoring over study.<br>• Dose escalation regimen with the use of pegfilgrastim as primary prophylaxis for neutropenia (Part 1B).<br>• Recommendation for use of G-CSF for subsequent dose administrations after neutropenia Grade ≥3 or febrile neutropenia is observed.<br>• These events should be managed and treated appropriately (hematopoietic growth factors or blood transfusion treatment [except DLT period] based on Investigator's judgment) or by M9140 dose interruption, dose modification or discontinuation (see Sections 6.5 and 7.1).

Identified and Potential Risks of Clinical Significance	Summary of Data/Rationale for Risk	Mitigation Strategy
**Gastrointestinal toxicity** (e.g., diarrhea, constipation, nausea, vomiting, stomatitis)	In cynomolgus monkeys treated with M9140, changes of the digestive tract (e.g., mucosal atrophy of colon and larynx, atrophy of acinar cells of the exocrine pancreas) were observed at/from 30 mg/kg, occasionally with diarrhea. In oncology patients treated with exatecan (Rowinsky 2005, De Jager 2000), GI toxicity was very common and generally mild to moderate (nausea and vomiting).<br>In Part 1 of the study (dose escalation), the most common GI TEAEs were nausea and vomiting. These GI toxicities were mild and moderate (Grade 1 and 2).	• Inclusion criteria: adequate organ function (liver, renal).<br>• Biochemistry, urinalysis, physical examination, and weight at baseline and monitoring over study.<br>• Exclusion criteria:<br>• Participants with diarrhea (liquid stool) or ileus > Grade 1.<br>• Participants with active chronic inflammatory bowel disease (e.g., ulcerative colitis, Crohn's disease, intestinal perforation) and/or bowel obstruction.<br>These events should be treated if necessary (antiemetic agents, antidiarrhea agents, [e.g., loperamide]) and treatment for stomatitis at discretion of the Investigator and according to institutional guidelines or by M9140 dose interruption, dose modification or discontinuation (see Sections 6.5 and 7.1).
**Fatigue**	CCI [redacted].	Management by M9140 dose interruption/dose modification or discontinuation based on Investigator's clinical judgment.
**Potential Risks**		
**Infusion-related reactions** (e.g., itching, flushing, hives, chills, fever, back pain, hypotension, anaphylactic shock)	CCI [redacted].	Exclusion of participants with history of severe hypersensitivity to M9140 or to one or more of the excipients used.<br>Severe hypersensitivity requires immediate discontinuation of study intervention, treatment according to the institutional guidelines and monitoring until the condition resolves.
**Genotoxicity and Teratogenicity**	Exatecan is regarded as genotoxic and teratogenic.	Contraception/barrier requirements included in eligibility criteria. Pregnancy information collection, monitoring, and reporting (see Section 8.3.3).
**Drug-drug interactions (DDIs)**	M9140 payload is metabolized by CYP450 (3A4 and to a minor extent 1A2 isozymes) and is expected to be cleared mainly via metabolism and to a minor extent by renal clearance.	Strong CYP3A4 inhibitors/inducers are prohibited (see Section 6.8.3 and Appendix 7).

Identified and Potential Risks of Clinical Significance	Summary of Data/Rationale for Risk	Mitigation Strategy
**Skin and subcutaneous: Alopecia**	CCI [redacted].	Education and supportive care (e.g., use of cosmetic and hair growth topical treatment) for patient comfort.<br>Management by M9140 dose interruption/dose modification or discontinuation based on Investigator's clinical judgment.
**Risk Associated with Study Procedures**		
**Blood sampling**	Blood sampling is required for participants as detailed in the schedule of activities. These are considered essential for the study's scientific objectives. Blood sampling carries a risk of adverse events including pain, bruising, bleeding, redness and swelling of the site/vein, and infection.	Minimization of blood sampling was thoughtfully considered during protocol development weighing risk to participants versus achievement of the study's scientific objectives.
**Tumor biopsy**	Fresh tumor biopsies are optional for the study during Part 2A and 2C as detailed in the schedule of activities and inclusion criteria. Biopsies carry a risk of adverse events including bleeding and infection.	Investigators are expected to use clinical judgment and not to proceed with the biopsy if clinically not indicated.
**Other**		
Allergic reactions to imaging contrast agents (e.g., rash, itching, kidney function impairment)	Reported for MRI with Gadolinium contrast agent.	Contraindicated for participants with renal dysfunction and skin hypersensitivity.
Phototoxicity	CCI [redacted].	Advice for participants to avoid direct sun exposure and to wear sunscreen (Section 5.3.4)

CYP: cytochrome 450, DLT: dose-limiting toxicity, GI: gastrointestinal, Hb: hemoglobin, Ht: hematocrit, RBC: red blood cells, MRI: magnetic resonance imaging.

Hematologic toxicity, GI toxicity, and fatigue are identified risks for M9140, while IRRs, genotoxicity and teratogenicity, DDIs, and alopecia are potential risks. Risk mitigation measures are built into the study design. These include use of appropriate inclusion/exclusion criteria, robust rationale for choice of starting dose, limiting exposure to small number of participants at each dose in the dose escalation cohorts and delay/period of observation between 1st and 2nd participant in each dose cohort in the DLT period. Moreover, participants in Part 1B of the study, will be given pegfilgrastim as primary prophylaxis for neutropenia (mandatory for the first 2 cycles and after Cycle 2 according to physician's discretion). Depending on the safety findings and SMC recommendation the same dose levels may be tested in Part 1A and Part 1B. Further, lifestyle restrictions, regular monitoring (clinical and labs), guidance on dose modification in the event of an adverse reaction and use of a Bayesian model to assist the SMC with dose selection in each subsequent cohort following the review of emerging data at specified timepoints will ensure that the risks are adequately managed and acceptable. For additional risk management measures specific to Japan, see Appendix 11.

The most common TEAEs observed in the dose escalation Part 1 of the study were anemia, nausea, fatigue, and white blood cell count decreased, while the most common severe (Grade ≥3) TEAEs were neutrophil count decreased/neutropenia, anemia, white blood cell count decreased, lymphocyte count decreased, and platelet count decreased.

Several Phase 1 studies have evaluated exatecan dosing in different administration schedules and all administration schedules were associated with the same type of DLTs (hematological toxicities) (De Jager 2000). Therefore, same type of TEAEs is expected with [redacted] administration schedule of M9140 as the one that was observed with the Q3W administration schedule used in the dose escalation Part 1 of the study.

Some of the most frequently observed adverse reactions across clinical trials, based on data from over 5,700 patients with various malignancies receiving bevacizumab, were hypertension, fatigue or asthenia, diarrhea, and abdominal pain. The most serious adverse reactions across clinical trials using bevacizumab are GI perforations, hemorrhage, and arterial thromboembolism. Please refer to the applicable local prescribing information for the full list of adverse reactions, as well as further information on the safety profile of bevacizumab.

The most commonly reported and/or clinically relevant treatment-related adverse drug reactions of capecitabine, based on data from over 3,000 patients treated with capecitabine as monotherapy or in combination with different chemotherapy regimens in multiple indications were GI disorders (especially diarrhea, nausea, vomiting, abdominal pain, stomatitis), hand-foot syndrome (palmar-plantar erythrodysesthesia), fatigue, asthenia, anorexia, cardiotoxicity, increased renal dysfunction on those with preexisting compromised renal function, and thrombosis/embolism. Please refer to the applicable local prescribing information for further information on the safety profile of capecitabine.

The most common toxicities associated with 5-FU treatment are myelosuppression, neutropenia, thrombocytopenia, leukopenia, agranulocytosis, anemia, pancytopenia, bronchospasm, immunosuppression, infections, pharyngitis, hyperuricemia, ECG signs of myocardial ischemia, mucosal inflammation, stomatitis, esophagitis, proctitis, anorexia, diarrhea, nausea, vomiting, palmar-plantar erythrodysesthesia syndrome, alopecia, delayed wound healing, epistaxis, malaise, asthenia and fatigue. Please refer to the applicable local prescribing information for further information on the safety profile of 5-FU. A lack of superiority but a more favorable benefit-risk profile for continuous infusion 5-FU over bolus 5-FU plus folinic acid has been shown in several clinical trials (Chau 2005, Poplin 2005, Köhne 2013). The Pan-European Trials in Adjuvant Colon Cancer (PETACC) 02 trial has observed no differences in relapse-free or overall survival for the high-dose infusion versus bolus 5-FU. Compared with bolus 5-FU, treatment with high-dose infusional 5-FU caused less mucositis and neutropenia, similar rates of diarrhea, and more hand-foot syndrome (Köhne 2013).

Generally, the safety profile of folinic acid depends on the applied regimen of 5-FU due to enhancement of the 5-FU induced toxicities. The most commonly reported toxicities associated with the use of folinic acid, when used in combination with 5-FU are bone marrow failure including fatal cases, leukopenia, neutropenia, thrombocytopenia and anemia, Palmar-Plantar Erythrodysesthesia, mucositis including stomatitis and cheilitis, vomiting, nausea, diarrhea (with

higher grades of toxicities), and dehydration resulting in hospital admission for treatment and even death.

The most common severe AEs observed with M9140 are primarily hematological toxicities and GI AEs are mild and moderate. The toxicity profiles of all 3 anti-tumor components of the combination regimens (5-FU, capecitabine, bevacizumab) show limited overlapping toxicities, however, considering the dose modification guidelines provided, the risk of a significant increase in the incidence or severity of AEs is considered relatively low and manageable. Moreover, risk minimization measures have been put in place to ensure participant's safety (see Section 2.3.1, Table 11, Section 6.5.5, and Table 18, Section 8.2). Additionally, the SMC will review safety data from Parts 2B, 2C, and 2D on a regular basis and may decide to modify or stop any of the study parts if unexpected AEs are observed or in SMC's opinion participants' safety is jeopardized (see Sections 4.1 and 6.5.3).

2.3.2 Benefit Assessment

The study will provide clinical data on the safety profile of M9140 in the dose escalation Part 1 in CRC. Then, in Part 2 it will investigate the clinical efficacy, safety, and tolerability of two M9140 dose levels in CRC as single agent when administered Q3W to inform selection of the optimal RP2D (dose optimization). It will also evaluate clinical efficacy, safety, and tolerability of an alternative [redacted] dosing regimen, and of M9140 in combination with CRC standard of care agents. It is expected that the [redacted] schedule and combination regimens assessed in Part 2 of the study will facilitate the future evaluation of M9140 as potential therapeutic option in earlier CRC treatment lines. The target populations of this study have high unmet medical need due to limited established therapeutic options in the late lines setting (see Section 4.1). M9140 may meet this unmet medical need due to its properties to selectively target CEACAM5-expressing tumor cells to deliver its cytotoxic payload to tumor cells by direct binding and bystander effect.

Nonclinical in vivo pharmacology studies indicate that M9140 is a highly potent ADC with strong antitumor activity in CDX and PDX tumor models. The antitumor activity of M9140 observed in CEACAM5-expressing CRC, GC and NSCLC PDX models indicates that M9140 may have the potential to deliver an efficacy benefit to patients with cancer in these indications whose tumors express CEACAM5.

Preliminary clinical data from the dose escalation Part 1 of the study have demonstrated a manageable safety profile consistent with exatecan toxicity with no new, unexpected AEs observed and encouraging preliminary antitumor activity of M9140 in heavily pretreated patients with colorectal cancer.

Bevacizumab, 5-FU, and capecitabine are approved agents for the treatment of CRC and are part of various SoC combination regimens for the treatment of CRC. Their use for mCRC is recommended by scientific organizations (NCCN guidelines 2024, ESMO guidelines 2023).

The high prevalence of high CEACAM5 expression in mCRC allows to investigate a CEACAM5-unselected CRC patient population in this study.

2.3.3 Overall Benefit/Risk Conclusion

Considering the measures taken to minimize risks to participants in this study, the potential risks identified in association with M9140 are justified by the anticipated benefits that may be afforded to participants with locally advanced or metastatic CRC, who were intolerant/refractory to or had progressive disease after 2 or 3 prior lines of SoC therapy depending on local standards and availability in Part 1. Furthermore, a positive benefit-risk ratio is seen for Part 2 with participants with locally advanced or metastatic CRC who were intolerant/refractory to or had progressive disease after 2 prior lines of SoC therapy depending on local standards and availability.

3 Objectives and Endpoints

3.1 Part 1 – M9140 Q3W Dose Escalation

Table 12 Objectives and Endpoints: Part 1A and Part 1B -- Dose Escalation

Objectives	Endpoints/Estimands	Ref. #
**Primary**		
To determine dose-toxicity relationship and MTD (if reached) of M9140 as monotherapy (Part 1A) and of M9140 monotherapy with pegfilgrastim prophylaxis (Part 1B) for investigated regimens in patients with locally advanced or metastatic CRC, respectively.	**Endpoint:**<br>• Occurrence of DLTs.<br>• Occurrence of AEs.<br>**Population:** Patients with locally advanced or metastatic CRC, who were intolerant/refractory to or had progressive disease after 2 or 3 prior lines of SoC therapy depending on local standards and availability.<br>**Strategies for handling intercurrent events:**<br>For DLTs:<br>• Discontinuation/interruption/delay of treatment (> 20% of planned cumulative dose in DLT period missed) to prevent a DLT: composite strategy (to be considered a DLT).<br>• Treatment with erythropoietin and /or blood transfusions during DLT period: composite strategy (to be considered a DLT).<br>• Only in Part 1A (without G-CSF prophylaxis): Administration of G-CSF within the DLT period: composite strategy (to be considered a DLT).<br>For AEs:<br>The endpoint will be analyzed regardless of whether the following intercurrent events had occurred (treatment policy strategy):<br>• Treatment discontinuation.<br>• Start of subsequent anticancer therapy.<br>**Population level summary**:<br>• DLT probabilities and associated credibility intervals as estimated using the BLRM model (for DLTs only).<br>• Incidence proportions.	1

Objectives	Endpoints/Estimands	Ref. #
To determine the RDE of M9140 as monotherapy (Part 1A) and of M9140 monotherapy with pegfilgrastim prophylaxis (Part 1B) for investigated regimens, respectively.	**Endpoint:** In addition to AEs and DLTs, the PK profile and preliminary clinical activity will be considered to determine the RDE.<br>**Population:** As defined for Objective #1.<br>**Strategy for handling intercurrent events:**<br>The endpoint will be analyzed regardless of whether the following intercurrent events had occurred (treatment policy strategy):<br>• Discontinuation of treatment.<br>• Start of subsequent anticancer therapy.<br>**Population level summary:**<br>1. Standard summary statistics.<br>2. Dose/exposure - Toxicity modeling as data permit.<br>3. Exposure-Response results as data permit.	2
**Secondary**		
To characterize the PK profile of M9140 (conjugated antibody, total antibody, and unconjugated exatecan payload)	**Endpoint:** PK parameters of M9140 conjugated antibody, total antibody, and unconjugated exatecan using noncompartmental analysis, e.g., AUC$_{0-t}$, AUC$_{\tau}$, C$_{max}$, C$_{trough}$, Cl, V$_{d}$, t$_{½}$.	3
To evaluate indicators of clinical activity of M9140 in terms of OR using RECIST v1.1.	**Endpoint:** OR according to RECIST v1.1 as assessed by Investigators.<br>**Population:** As defined for Objective #1.<br>**Strategy for handling intercurrent events:**<br>The endpoint will be analyzed regardless of whether the following intercurrent events had occurred:<br>• Discontinuation of treatment (treatment policy strategy, i.e., regardless of the intercurrent event).<br>• Start of subsequent anticancer therapy (while not treated with subsequent anticancer therapy strategy, i.e., ignoring tumor assessments after the intercurrent event).<br>• Progression according to RECIST v1.1 (while not progressed strategy).<br>**Population level summary:** OR rate.	4
To evaluate indicators of clinical activity of M9140 in terms of DoR using RECIST v1.1.	**Endpoint:** DoR according to RECIST v1.1 as assessed by Investigators, defined as time from first documentation of OR to PD or death, occurring within 2 scheduled tumor assessments after last evaluable assessment or start of treatment.<br>**Strategy for handling intercurrent events:**<br>• Death within 2 scheduled tumor assessments after last evaluable assessment or first study intervention will be considered as event (composite strategy).<br>The endpoint will be analyzed regardless of whether the following intercurrent events had occurred (treatment policy strategy):<br>• Discontinuation of treatment.	5

Objectives	Endpoints/Estimands	Ref. #
	• Start of subsequent anticancer therapy.<br>**Population:** Patients as defined for Objective #1, with confirmed OR according to RECIST v1.1.<br>**Population level summary:** Median DoR.	
To evaluate indicators of clinical activity of M9140 in terms of PFS (RECIST v1.1).	**Endpoint:** PFS as defined from date of first study intervention to PD according to RECIST v1.1 as assessed by Investigators or death.<br>**Strategy for handling intercurrent events:** As defined for DoR (Objective #5).<br>**Population:** As defined for Objective #1.<br>**Population level summary:** Median and rate.	6
To characterize the immunogenicity of M9140.	**Endpoint:** ADA against M9140 occurrence and titer, as measured by ADA assay.	7
To assess the effect of M9140 on QTc interval.	**Endpoint:** Triplicate digital ECG measures, change from the baseline QTc (ΔQTc) over predefined timepoints.	8

CCI [redacted]

ADA: anti-drug antibodies, AE: adverse event, BLRM: Bayesian logistic regression model, CCI [redacted], CRC: colorectal cancer, DLT: dose-limiting toxicity, DoR: duration of response, ECG: electrocardiogram, MTD: maximum tolerated dose, OR: objective response, PD: progressive disease, PFS: progression-free survival, PK: pharmacokinetic, QTc: corrected QT interval, RDE: recommended dose for expansion, RECIST: Response Evaluation Criteria in Solid Tumors, SoC: standard of care.

3.2 Part 2A – M9140 Q3W Dose Optimization

Table 13 Objectives and Endpoints/Estimands: Part 2A – M9140 Q3W Dose Optimization

Objectives	Endpoints/Estimands	Ref. #
**Primary**		
To determine clinical activity in terms of OR and DoR of two M9140 dose levels to inform selection of RP2D of M9140 monotherapy Q3W in patients with locally advanced or metastatic CRC.	**Endpoint:**<br>• OR according to RECIST v1.1 as assessed by Investigators.<br>• DoR according to RECIST v1.1 as assessed by Investigators, defined as time from first documentation of objective response to PD or death, occurring within 2 scheduled tumor assessments after last evaluable assessment or start of treatment.	11

Objectives	Endpoints/Estimands	Ref. #
	**Population:**<br>For OR: Patients with locally advanced or metastatic CRC who were intolerant/refractory to or had progressive disease after 2 prior lines of therapy depending on local standards and availability.<br>For DoR: Patients as defined for Objective #11, with confirmed OR according to RECIST v1.1.<br>**Strategy for handling intercurrent events:**<br>**OR:** As defined for Objective #4.<br>**DoR:** Death within 2 scheduled tumor assessments after last evaluable assessment or first study intervention will be considered as event (composite strategy).<br>The endpoints will be analyzed regardless of whether the following intercurrent events had occurred (treatment policy strategy):<br>• Discontinuation of treatment.<br>• Start of subsequent anticancer therapy.<br>**Population level summary:** OR difference between Arm A1 and Arm A2, Median DoR.	
To determine safety and tolerability of two M9140 dose levels to inform selection of RP2D of M9140 in patients with locally advanced or metastatic CRC.	**Endpoint:**<br>• Occurrence of AEs.<br>**Population:** As defined for Objective #11.<br>**Strategy for handling intercurrent events:**<br>The endpoints will be analyzed regardless of whether the following intercurrent events had occurred (treatment policy strategy):<br>• Discontinuation of treatment.<br>• Start of subsequent anticancer therapy.<br>**Population level summary:** Incidence proportions and difference in proportions for Arm A1 and Arm A2.	12
**Secondary**		
To evaluate indicators of clinical activity of M9140 in terms of DC at 12 weeks	**Endpoint:** DC defined as CR, PR, SD, or non-CR/non-PD at week 12 visit or later prior to documented PD.<br>**Population:** As defined for Objective #11.<br>**Strategy for handling intercurrent events:** As defined for OR (Objective #4).<br>**Population-level summary:** DCR as proportion of participants with disease control at week 12 visit.	13
To evaluate indicators of clinical activity of M9140 in terms of time to response and PFS using RECIST v1.1.	**Endpoint:**<br>• Time to response defined as time from date of randomization to first documentation of objective response according to RECIST v1.1 as assessed by investigators.<br>• PFS as defined from date of randomization to PD according to RECIST v1.1 as assessed by Investigators or death.<br>**Strategy for handling intercurrent events:**<br>For PFS, as defined for DoR (Objective #11).<br>For TTR, as defined for OR (Objective #4).<br>**Population:** As defined for Objective #11.<br>**Population level summary:** Median TTR, median PFS, and PFS rate at 3, 6, 9, and 12 months.	14

Objectives	Endpoints/Estimands	Ref. #
To evaluate indicators of clinical activity of M9140 in terms of OS.	**Endpoint:** OS defined as time from date of randomization to death.<br>**Population:** As defined for Objective #11.<br>**Strategy for handling intercurrent events:**<br>• The endpoint will be analyzed regardless of whether the following intercurrent events had occurred (treatment policy strategy):<br>• Treatment discontinuation.<br>• Start of subsequent anticancer therapy.<br>**Population level summary:** Median and rate.	15
To assess the effect of M9140 on QTc interval.	**Endpoint:** Triplicate digital ECG measures, change from baseline QTc (ΔQTc) over predefined timepoints to contribute to concentration-QTc analysis.	16
To characterize the PK profile of M9140 (conjugated antibody, total antibody and unconjugated exatecan payload).	**Endpoint:** PK parameters of M9140 (conjugated antibody, total antibody, and unconjugated exatecan) using noncompartmental analysis, e.g. AUC_{0-t}, AUC_{τ}, C_{max}, C_{trough}, Cl, V_d, $t_{½}$.	17
To characterize the immunogenicity of M9140.	**Endpoint:** ADA against M9140: occurrence and titer, as measured by ADA assay.	18
To describe patient-reported symptomatic AEs of M9140 by dose levels in Arm A1 and Arm A2	**Endpoint:** Symptomatic AEs and related impacts as measured by selected questions addressing fatigue, nausea, vomiting, diarrhea, mouth sores	19

CCI [redacted]

AE: adverse event, ADA: anti-drug-antibodies, CCI [redacted], CRC: colorectal cancer, CCI [redacted], DoR: duration of response, ECG: electrocardiogram, OR: objective response, OS: overall survival, PFS: progression-free survival, PD: progressive disease, PK: pharmacokinetic, QTc: corrected QT interval, RECIST: Response Evaluation Criteria in Solid Tumors, SoC: standard of care.

3.3 Part 2B (M9140 [redacted]), Part 2C (M9140 Q3W in Combination with Bevacizumab or Bevacizumab plus Capecitabine), and Part 2D (M9140 [redacted] in Combination with 5-FU plus Bevacizumab)

Table 14 Objectives and Endpoints/Estimands: Part 2B, Part 2C, and Part 2D

Objectives	Endpoints/Estimands	Ref. #
**Primary**		
To assess safety to inform selection of the RP2D of M9140 as monotherapy given [redacted] (Part 2B), of M9140 Q3W in combination with bevacizumab and bevacizumab plus capecitabine (Part 2C), and of M9140 [redacted] in combination with 5-FU plus bevacizumab (Part 2D), in patients with locally advanced or metastatic CRC.	**Endpoint:**<br>• Occurrence of DLTs<br>• Occurrence of AEs<br>**Population:** Patients with locally advanced or metastatic CRC who were intolerant/refractory to or had progressive disease after 2 prior lines of therapy depending on local standards and availability.<br>**Strategies for handling intercurrent events:**<br>For DLTs:<br>• Discontinuation/interruption/delay of treatment (> 20% of planned cumulative dose in DLT period missed) to prevent a DLT: composite strategy (to be considered a DLT).<br>• Treatment with erythropoietin and /or blood transfusions during DLT period: composite strategy (to be considered a DLT).<br>• Administration of G-CSF within the DLT period: composite strategy (to be considered a DLT).<br>For AEs:<br>The endpoint will be analyzed regardless of whether the following intercurrent events had occurred (treatment policy strategy):<br>• Treatment discontinuation.<br>• Start of subsequent anticancer therapy.<br>**Population level summary**:<br>• DLT probabilities and associated credibility intervals as estimated using the BLRM model (for DLTs only).<br>• Incidence proportions.	25
**Secondary**		
To evaluate indicators of clinical activity of M9140 as monotherapy given [redacted] (Part 2B), of M9140 Q3W in combination with bevacizumab and bevacizumab plus capecitabine (Part 2C), and of M9140 [redacted] in combination with 5-FU plus bevacizumab (Part 2D), in terms of OR and DC at 12 weeks using RECIST v1.1.	**Endpoint:**<br>• OR according to RECIST v1.1 as assessed by Investigators.<br>• DC defined as CR, PR, SD, or non-CR/non-PD at week 12 visit or later prior to documented PD<br>**Population:** As defined for Objective #25<br>**Strategy for handling intercurrent events:**<br>The endpoint will be analyzed regardless of whether the following intercurrent events had occurred:<br>• Discontinuation of treatment (treatment policy strategy, i.e., regardless of the intercurrent event).<br>• Start of subsequent anticancer therapy (while not treated with subsequent anticancer therapy strategy, i.e., ignoring tumor assessments after the intercurrent event).<br>• Progression according to RECIST v1.1 (while not progressed strategy).	26

Objectives	Endpoints/Estimands	Ref. #
	**Population level summary:**<br>• OR rate.<br>• DCR as proportion of participants with disease control at week 12 visit.	
To evaluate indicators of clinical activity of M9140 in terms of time to response using RECIST v1.1.	**Endpoint** Time to response defined as time from date of first study intervention to first documentation of objective response according to RECIST v1.1 as assessed by Investigators.<br>**Population:** As defined for Objective #25.<br>**Strategy for handling intercurrent events:**<br>The endpoint will be analyzed regardless of whether the following intercurrent events had occurred:<br>• Discontinuation of treatment (treatment policy strategy, i.e., regardless of the intercurrent event).<br>• Start of subsequent anticancer therapy (while not treated with subsequent anticancer therapy strategy, i.e., ignoring tumor assessments after the intercurrent event). Progression according to RECIST v1.1 (while not progressed strategy).<br>**Population level summary:** OR rate.	27
To evaluate indicators of clinical activity of M9140 in terms of DoR using RECIST v1.1.	**Endpoint:** DoR according to RECIST v1.1 as assessed by Investigators, defined as time from first documentation of OR to PD or death, occurring within 2 scheduled tumor assessments after last evaluable assessment or start of treatment.<br>**Population:** Patients as defined for Objective #25, with confirmed OR according to RECIST v1.1.<br>**Strategy for handling intercurrent events:**<br>• Death within 2 scheduled tumor assessments after last evaluable assessment or first study intervention will be considered as event (composite strategy).<br>The endpoint will be analyzed regardless of whether the following intercurrent events had occurred (treatment policy strategy):<br>• Discontinuation of treatment.<br>• Start of subsequent anticancer therapy.<br>**Population level summary:** Median DoR	28
To evaluate indicators of clinical activity of M9140 in terms of PFS (RECIST v1.1).	**Endpoint:** PFS as defined from date of first study intervention to PD according to RECIST v1.1 as assessed by Investigators, or death.<br>**Strategy for handling intercurrent events:** As defined for DoR (Objective #28).<br>**Population:** As defined for Objective #2.<br>**Population level summary:** Median and rate.	29
To characterize the PK profile of M9140 (conjugated antibody, total antibody and unconjugated exatecan payload), in a [redacted] administration schedule as monotherapy or in combination regimen, or Q3W in combination regimens.	PK parameters of M9140 (conjugated antibody, total antibody, and unconjugated exatecan) using noncompartmental analysis, e.g., $AUC_{0\text{-}t}$, AUC_{τ}, C_{max}, C_{trough}, CI, V_d, $t_{½}$.	30
To characterize the immunogenicity of M9140.	ADA against M9140: occurrence and titer, as measured by ADA assay.	31

Objectives	Endpoints/Estimands	Ref. #
CCI [redacted]		

4 Study Design

4.1 Overall Design

Study Design	FIH, 2-part (dose escalation and expansion), clinical study of M9140 ADC
Control Method	Uncontrolled
Single or Multicenter	Multicenter<br>Part 1: US/Spain/Japan<br>Part 2: North America/EU/Asia
Control Group	Not applicable
Study Population Type	**<u>Part 1 - Dose Escalation</u>**<br>Participants with documented histopathological diagnosis of locally advanced or metastatic CRC, who were intolerant/refractory to or progressed after systemic therapies in the advanced/metastatic setting that included and are restricted to a [redacted], if locally indicated and available to the participant. Participants with a known MSI-H status must have received treatment with an immune checkpoint inhibitor (if locally indicated and available) unless contraindicated. See Appendix 11 for additional requirements in Japan.<br>**<u>Part 2 - Dose Expansion</u>**<br>**Parts 2A, 2B, 2C, and 2D:**<br>• Participants with documented histopathological diagnosis of locally advanced or metastatic CRC.<br>• Participants must have demonstrated progressive disease according to RECIST v1.1 during or after the most recent regimen.<br>• Participants must have received at least 1 previous systemic treatment regimen in the advanced/metastatic disease setting but no more than 2.<br><u>Exception:</u> patients with MSI-H disease or BRAF positive disease are allowed to have had 3 previous regimens.<br>• Previous systemic regimens must have included the following (provided no medical contraindication and agent is locally available):<br>○ [redacted].<br>○ [redacted].

	○ treatment with an immune checkpoint inhibitor for participants with a known MSI-H status.<br>○ previous treatment with [redacted] for participants with known BRAF gene mutations<br><br>Notes:<br>• [redacted].<br>• Participants may have previously received experimental treatments, as long as the maximum number of lines is respected, and no protocol-prohibited agents were administered.<br>• Changes of regimen components, due to unacceptable toxicity without signs of progression, will not be counted as a line.<br>See Appendix 11 for additional requirements in Japan.
Level and Method of Blinding	Open label
Bias Minimalization Method(s)	Not applicable
Study Intervention Assignment Method	Not applicable for Part 1 and Parts 2B, 2C, and 2D.<br>In Part 2A dose optimization, after confirmation of participant's eligibility and at the last practical moment prior to study intervention administration, participants will be centrally allocated to either 2.8 mg/kg of M9140 (Arm A1) or 2.4 mg/kg (Arm A2) of M9140 in a 1:1 ratio, stratified by BMI (BMI ≤ 30 vs BMI > 30) to ensure a balanced ratio of participants with a dosing cap due to their BMI across both arms, using an IRT randomization system and per a computer-generated randomization list.
Number of Participants	The total number of participants in Part 1 will depend on the number of cohorts to be evaluated.<br>It is anticipated that approximately 21 to 30 participants (5 projected dose levels with 3 to 9 participants each) will be exposed to study intervention in cohorts without pegfilgrastim prophylaxis (Part 1A) and 9-15 participants (3 projected dose levels with 3 to 9 participants each) in cohorts with pegfilgrastim prophylaxis (Part 1B).<br><br>Part 2 will include approximately 135 to 170 participants. Part 2A will randomize 60 participants to 2.8 mg/kg (Arm A1) and 2.4 mg/kg (Arm A2) (30 participants in each). Approximately 20 to 30 participants will be enrolled in Part 2B and approximately 35 to 50 participants in Part 2C (approximately 15 to 20 in Part 2C1

	and approximately 20 to 30 in Part 2C2). For Part 2D, approximately 20 to 30 participants will be enrolled.<br>The Sponsor will monitor enrollment in the study and may make determinations to limit enrollment in certain countries and/or regions in order to obtain a study population representative across the participating global regions (for example Asian versus non-Asian countries).
Total Duration of Study Participation per Participant	Study duration per participant is on average approximately 4 months for Part 1 and 8 months for Part 2 (without Survival follow-up). This includes a 28-day Screening period, infusion (approximately 1 hour) on Day 1 of every cycle, and Safety Follow-up Visit 30 (± 3) days after the last dose of M9140 administration. Furthermore, for Part 2A, Survival follow-up will be conducted every 3 months (± 2 weeks) until End of Study or up to 24 months after first dose, whichever comes first (see Section 4.4).
Parts of the Study	Part 1 – Dose Escalation<br>Part 2 – Dose Expansion
Method Used for Dose Escalation	Methods to support SMC recommendation:<br>• Bayesian 2-parameter logistic regression model (Part 1 and Part 2B)<br>• iBOIN (Part 2C1)<br>• Continual Reassessment Method (Part 2C2 and 2D)
Adaptive Aspects of Study Design	SMC recommendation of dose/regimen for escalation cohorts in Part 1 and Part 2. The number of participants per cohort and dose level may be flexible based on SMC recommendation.<br>Choice of starting dose for escalation with pegfilgrastim support (Part 1B).<br>Choice of dose(s) for the expansion parts after dose escalation part reached RDE suggestion.<br>Changes to doses in the expansion parts per SMC recommendation.
Screening, Treatment, and Follow-up Periods:	**Screening**: Screening will be performed within 28 days prior to Day 1 of M9140 administration. If there are no clinically significant findings at Screening and the participant meets all the protocol-defined inclusion and none of the exclusion criteria, the participant will be considered eligible for participation in the study. Screening for the next cohort should usually start before the decision on the next dose has been made. The eligible participants screened for the next cohort will, however, only be treated following SMC recommendation.<br>**Treatment period:** It will begin at the first dose of M9140 in Cycle 1 Day 1 (C1D1) and consist of consecutive 21-day cycles of M9140 study intervention, except Parts 2B and 2D in which participants will receive consecutive [redacted]-day cycles.

	DLT observation period will be 21 days starting on the day of the first administration of M9140 for Part 1 and Part 2C and [redacted] days starting on the day of the first administration of M9140 for Parts 2B and 2D.<br>The dose (in mg) to be administered to each participant will be calculated based on the participant's weight rounded to the nearest kilogram, i.e., assigned cohort dose level in mg/kg x body weight in kg.<br>For participants whose BMI is greater than 30 kg/m^2, on the administration day the Investigator should use a weight that, based on the participant's height, corresponds to a maximum BMI of 30 kg/m^2. The dose is calculated according to the following formula if BMI is greater than 30 kg/m^2:<br>Dose (mg) = Dose Level (mg/kg) x 30 (kg/m^2) x (height [m])^2<br>BMI should be calculated based on the following formula: weight in kilograms (kg) divided by height in meters (m) squared and rounded to the nearest single decimal (e.g., for a participant with weight of 70 kg and height of 1.75 m the BMI would be calculated as follows: 70 kg/(1.75 m x 1.75 m) = 22.9 kg/m^2).<br>**Part 1: Dose Escalation**<br>The dose escalation explores primarily the following 2 regimens:<br>• Part 1A: M9140 will be administered iv Q3W with no pegfilgrastim prophylaxis during the DLT observation period. Use of G-CSF outside the DLT observation period is allowed according to physician's discretion if needed to treat a neutropenic and/or febrile neutropenia event or as secondary prophylaxis after a neutropenia of Grade ≥3 or febrile neutropenia event has been observed in previous cycles (see Section 6.5.5, Table 19).<br>• Part 1B: M9140 will be administered iv Q3W with mandatory pegfilgrastim administration as primary prophylaxis for neutropenia on day 8 (±3 days) of the first 2 cycles. Use of G-CSF for all cycles after cycle 2 is optional according to physician's discretion.<br>The first dose escalation cohort of 3 participants will receive M9140 at the starting dose of 0.6 mg/kg Q3W. The term "cohort" with regard to dose escalation in this protocol is referring to a group of participants that are enrolled at the same dose level and evaluated by an SMC before the dosing decision for the next cohort is made. There may be more than one cohort on the same dose level, if the SMC recommends that a further cohort on the same dose level is needed.<br>A preselected set of doses (the first 5 projected escalation steps [DL1 to DL5] with doses of 0.6, 1.2, 1.8, 2.4, and 3.0 mg/kg and subsequent escalation steps with 20% dose increases) was considered by the Bayesian model, although doses that are not part of the prespecified set may be chosen as well. Depending on the observed safety profile

	and available PK data the SMC may recommend doses that are different from what was prespecified.<br>Enrollment to cohorts in Part 1B (the escalation part with mandatory pegfilgrastim prophylaxis) may start after SMC recommendation (and Sponsor agreement) and will then proceed in parallel to the cohorts in Part 1A (without pegfilgrastim prophylaxis) escalation. The SMC will recommend the starting dose of the escalation in Part 1B based on the observed safety and available PK data until then.<br>Potential doses to be tested in Part 1B are 3.2, 3.6, and 4.0 mg/kg, and subsequent escalation steps with dose increases that cannot be >20% are planned. Depending on the observed safety profile and available PK data the SMC may recommend doses that are different from what was prespecified. Depending on the safety findings and SMC recommendation the same dose levels may also be tested in Part 1A.<br>In order to ensure that an adequate period of time exists between the administration of treatment of the first participant on each dose level in each regimen (Part 1A and Part 1B) and the second participant in the same cohort, to observe if any reactions and AEs occur, the first participant enrolled will be observed for DLTs for at least 7 days before the second participant is enrolled in this cohort. In cases where a dose has already been tested in Part 1A and the same dose is initiated in Part 1B, there is no need for the 7-day observation period.<br>Safety and tolerability data (including but not limited to TEAEs, SAEs, DLTs) of the first participant will be discussed between the treating investigator and the Sponsor, before additional participants are treated on that dose level to ensure that relevant safety information of the sentinel participant is received and reviewed. In case the sentinel patient develops a DLT then an ad hoc SMC meeting consultation should be held to evaluate the totality of safety and tolerability data of this participant and recommend the appropriate steps. Until the SMC issues its recommendation no additional participants can be enrolled.<br>In principle, dose escalation in each regimen (Part 1A and Part 1B) will proceed according to the SMC recommendation until the MTD for this regimen and/or RDE for this regimen is determined and/or the SMC recommends ending dose escalation in the concerned regimen. The SMC recommends the MTD. Target DLT probability for the suggested MTD by the Bayesian model is 30%.<br>At least 6 evaluable participants need to be treated at the MTD/RDE, of whom at least 4 received ≥ 80% of the uncapped dose.<br>If further data on the RDE dose(s) are needed, additional individuals can be enrolled in parallel to further escalation.<br>The SMC recommends an RDE for each regimen, but the final selection of the RDE is made by the Sponsor.<br>The RDE dose(s) cannot exceed the MTD. If the suggested RDE dose(s) are below the MTD, dose escalation will continue until the

	MTD is reached (if applicable) or the SMC recommends ending dose escalation.<br>**<u>Part 2 Dose Expansion</u>:**<br>Once an RDE/MTD of M9140 has been defined which is considered safe by the SMC, Part 2 dose expansion may begin. Initiation of Part 2 may proceed at the RDE dose(s) (recommended by SMC based on Part 1 dose escalation) in parallel with continued dose escalation toward the MTD in Part 1.<br>It is aimed to investigate M9140 antitumor activity, tolerability, and safety in 4 expansion parts (Parts 2A, 2B, 2C, and 2D) in unselected participants with mCRC.<br>**Part 2A**, aimed at dose optimization, will evaluate two different dose levels of M9140 to support the selection of the RP2D to be used for further development. Approximately 60 participants will be randomized in a 1:1 ratio to receive either 2.8 mg/kg (Arm A1) or 2.4 mg/kg (Arm A2) of M9140, both given Q3W. A dose selection analysis will be performed to evaluate both safety and efficacy of these two doses and to support the selection of the RP2D for the Q3W administration schedule.<br>An interim analysis to assess futility will be performed after 15 participants in each arm of Part 2A are evaluable for response, i.e. have had at least one on-study tumor assessment or have dropped out, to consider stopping one or both of the two dose groups. Moreover, the SMC will evaluate the totality of safety data to determine the tolerability of each dosing regimen after 8 and 15 participants have completed Cycle 1 in each arm, and as needed.<br>If one dose regimen is stopped (for either safety or efficacy reasons) participants in that group will be offered the option to move to the other dose group. This refers to both participants who have already received treatment in the stopped dose regimen and participants who have been randomized to that dose regimen and have not yet started treatment.<br>For details on the decision criteria of the interim analyses, see Section 9.2.<br>**Part 2B** will evaluate the safety and tolerability of M9140 on a [redacted] dosing regimen. Using preliminary modeling and simulation analyses and the totality of evidence integrating PK, tumor dynamics, and safety data, and based on the low M9140 dose used in Part 2A (2.4 mg/kg), unless otherwise recommended by the SMC, the anticipated starting dose (DL1) of Part 2B will be [redacted]. Approximately 3 participants will be treated in DL1. The SMC will evaluate the totality of data available for those participants and decide on the dose for the next cohort(s). [redacted]

CCI [redacted]

The Sponsor upon recommendation by the SMC and using emerging data from Part 2A may:

- Evaluate other doses as well, as long as they do not exceed the time-averaged dose intensity corresponding to the MTD defined with Q3W regimen.
- Decide to add more participants in any DL to better determine the safety and tolerability profile, without exceeding the prespecified maximum number of participants in Part 2B (n=30).

Part 2C will evaluate M9140 when given in combination with agents that are standard of care in the treatment of mCRC.

Part 2C1 will evaluate M9140 in combination with bevacizumab 7.5 mg/kg given Q3W. DL1 will be comprised of M9140 at the dose of 2.4 mg/kg and bevacizumab 7.5 mg/kg both administered in consecutive 21-day cycles. Approximately 3 participants will be treated in DL1. The SMC will evaluate the totality of data available for those participants and decide on the dose for the next cohort(s). The planned DL2 is M9140 at the dose of 2.8 mg/kg and bevacizumab 7.5 mg/kg. Other M9140 doses may be chosen as well, as long as they do not exceed the highest dose level declared safe in Part 1.

Part 2C2 will evaluate M9140 in combination with bevacizumab plus capecitabine.

Enrolment into Part 2C2 can only start after DL1 in Part 2C1 has been deemed safe and tolerable by the SMC.

DL1 of Part 2C2 will be comprised of M9140 at the dose of 2.4 mg/kg Q3W, bevacizumab 7.5 mg/kg Q3W and capecitabine at 800 mg/m^2 bid (D1-14, q21 days). Approximately 3 participants will be treated in DL1. The SMC will evaluate the totality of data available for those participants and decide on the dose for the next cohort(s). The planned DL2 is M9140 at the dose of 2.4 mg/kg, combined with bevacizumab 7.5 mg/kg and capecitabine at 1,000 mg/m^2 bid. If this dose level is deemed tolerable by the SMC, participants in the next dose level will be treated with M9140 at 2.8 mg/kg together with bevacizumab 7.5 mg/kg and capecitabine at 1,000 mg/m^2 bid. If DL1 is not deemed tolerable by the SMC, then a DL -1 will be considered comprising of M9140 at the dose of 2.0 mg/kg, bevacizumab 7.5 mg/kg and capecitabine at 800 mg/m^2 bid. Other M9140 doses may be chosen as well, as long as they do not exceed the highest dose level declared safe in Part 1.

For both Parts 2C1 and 2C2, if additional data are required for any DL to better determine the safety and tolerability profile, more participants may be added based on SMC recommendation. If Arm A1 or Arm A2 is terminated for safety reasons, then the corresponding DL in

	Parts 2C1 and 2C2 will be terminated. The Sponsor in collaboration with the SMC may decide to add more participants in any DL based on emerging data from Part 2A, not exceeding the prespecified maximum number of participants in each part (n=20 for Part 2C1 and n=30 for Part 2C2). Additionally, the Sponsor in collaboration with the SMC may decide to evaluate additional DLs.<br><br>**Part 2D** will evaluate M9140 in combination with 5-FU plus bevacizumab given [redacted]. Enrolment into this part can only start after DL1 of Part 2B [redacted] and DL1 of Part 2C1 (M9140 at 2.4 mg/kg plus bevacizumab at 7.5mg/kg Q3W) have been deemed safe and tolerable by the SMC.<br><br>[redacted]. Approximately 3 participants will be treated in DL1. The SMC will evaluate the totality of data available for those participants and decide on the dose for the next cohort(s). [redacted]. If DL2 is deemed safe and tolerable by the SMC, participants in the next dose level will be treated with M9140 at the dose of [redacted]. At all dose levels, infusion of 5-FU is always preceded by an infusion of folinic acid at 400 mg/m^2.<br><br>Other doses of M9140 [redacted] may be chosen as well, as long as they do not exceed the highest dose level declared safe in Part 2B.<br><br>For Part 2D, if additional data are required for any DL to better determine the safety and tolerability profile, more participants may be added based on SMC recommendation. If any DL in Part 2B is terminated for safety reasons, then the corresponding DL in Part 2D will be terminated. The Sponsor in collaboration with the SMC may decide to add more participants in any DL based on emerging data from other study parts, not exceeding the prespecified maximum number of participants (n=30). Additionally, the Sponsor in collaboration with the SMC may decide to evaluate additional DLs based on the DLT rate and the overall safety profile.<br><br>To ensure that an adequate period of time exists between the administration of treatment for the first participant on each DL in Part 2C and 2D and the second participant in the same DL, to observe if any reactions and AEs occur, the first participant enrolled will be observed for DLTs for at least 7 days before the second participant is enrolled in this cohort. Additionally, the first participant in each DL of Part 2C2 will only be dosed once the first participant in the corresponding DL of Part 2C1 has been dosed and completed an observation period of at least 7 days.

	Safety and tolerability data (including but not limited to TEAEs, SAEs, DLTs) of the first participant will be discussed between the treating Investigator and the Sponsor, before additional participants are treated on that DL to ensure that relevant safety information of the sentinel participant is received and reviewed. In case the sentinel participant develops a DLT then an ad hoc SMC meeting consultation should be held to evaluate the totality of safety and tolerability data of this participant and recommend the appropriate steps. Until the SMC issues its recommendation, no additional participants can be enrolled.<br>**For all Parts 2A, 2B, 2C, and 2D**, participants will receive M9140 until disease progression, unacceptable toxicity, withdrawal of consent, or any criterion for withdrawal from the study (Section 7.2).<br>A total of at least 9 evaluable participants need to be treated at each RP2D (Parts 2B, 2C, and 2D), of whom at least 6 received ≥ 80% of the uncapped dose, provided that the maximum number of participants to be enrolled in that part is not exceeded. The SMC recommends an RP2D for each regimen, but the final selection of the RP2Ds is made by the Sponsor.<br>During the expansion part of the study (Part 2), SMC meetings will be held as described in Section 6.5.3. The SMC may recommend by consensus on continuation at the same dose, change in dose, or stop of expansion parts. Decision making of the SMC will be supported by results of a Bayesian 2-parameter logistic model (Part 2B), an iBOIN design (Part 2C1), or a Continual Reassessment Method (CRM) model (Part 2C2 and Part 2D). Enrollment will continue during preparation of the SMC. Medical monitoring will include observation of AEs meeting the DLT criteria as described in Section 6.5.4.<br>**End of Treatment Visit**: Occurs within 7 days of decision of M9140 treatment discontinuation (to be performed before starting of subsequent anticancer therapies).<br>**Safety Follow-up Visit**: Occurs 30 ± 3 days after the last M9140 administration.<br>Safety Follow-up visit is not required in case the EOT visit falls on the same time window (30 ± 3 days after last M9140 dose) as the Safety Follow-up visit.<br>**Survival Follow-up Period** (only Part 2A): Participants will be followed up for survival every 3 months (± 2 weeks) via a phone call until end of study or up to 24 months after first dose, whichever comes first (see Section 4.4).
Involvement of Special Committee:	Yes.<br>The SMC will monitor safety and recommend on dosing during the dose escalation phase and expansion phase of the study (Section 6.5.2 and Appendix 8).

Provisions for Study Extension or Entry into Rollover Studies	Not applicable.

4.2 Scientific Rationale for Study Design

The Phase 1, two-part (dose escalation and dose expansion), study is aiming to establish a safe and tolerable dose and to investigate PK and the primary clinical efficacy of M9140 in monotherapy (Part 1 and Parts 2A and 2B) and in combination with SoC agents (Part 2C and 2D) in participants with locally advanced or metastatic CRC.

4.2.1 Part 1 – M9140 Dose Escalation

The **dose escalation part** will initially evaluate M9140 administered by iv infusion Q3W as the primary regimen. This regimen is intended to be investigated for M9140 monotherapy as well as for potential later combination therapy trials of M9140 with Q3W SoC regimens.

Neutropenia is a well described and common AE for exatecan and has been reported as a DLT in participants treated with exatecan in various Phase 1 studies (de Jager 2000). Mandatory pegfilgrastim (administered on D8 [±3 days] of first 2 cycles) in Part 1B is expected to reduce the risk of treatment-related neutropenia/decreased neutrophil count as well as febrile neutropenia at higher dose, to improve participant convenience, and to allow higher doses to be tested that may result into earlier and more deep responses. Finally, it may also facilitate potential combinations with other anticancer agents in future studies. Pegfilgrastim is commonly used in routine clinical practice, has a well described safety profile, and is recommended by scientific communities (Aapro 2011; Smith 2015). Therefore, its introduction in the current study is not expected to negatively impact participants' wellbeing.

Thus, the study will evaluate a regimen with (Part 1B) and without prophylactic pegfilgrastim administration (Part 1A) to define the most appropriate administration strategy for M9140 and determine the dose(s) that will be evaluated in the expansion part of this study. Based on additional data generated, such as antitumor activity observed in CDX and PDX tumor models, and/or compelling clinical efficacy demonstrated in Part 1 or at the interim analyses, further expansion cohorts in different indications known to express CEACAM5 may be considered for Part 2. The potential cohorts for exploring additional indications or/and combination treatment in Part 2 will be detailed in a protocol amendment to be approved by Regulatory Authorities.

Current clinical experience indicates dose-related neutropenia that appears to be associated with the exposures of unconjugated payload exatecan, based on preliminary PK data. In order to protect obese participants (BMI > 30 kg/m^2) from excessive exposures and potential increased risk for treatment related adverse events that could potentially result from mg/kg dosing, a maximum absolute dose limit (dose cap) corresponding to a weight that, based on the participant's height, corresponds to a BMI of 30 kg/m^2 will be implemented for participants whose BMI is greater than 30 kg/m^2.

4.2.2 Parts 2A, 2B, 2C, and 2D – Dose Expansion

In the dose optimization Part 2A, it is planned to randomize in a 1:1 ratio approximately 60 participants with locally advanced or metastatic CRC to M9140 2.8 mg/kg (Arm A1) and 2.4 mg/kg (Arm A2), Q3W, to identify the RP2D dose to be used for further development.

The study design also includes an independent cohort (Part 2B) of approximately 20 to 30 additional participants with locally advanced or metastatic CRC who will receive M9140 at a [redacted] administration schedule.

The encouraging preliminary anti-tumor activity observed with single agent M9140 in participants with advanced and metastatic CRC in the dose escalation Part 1 of the study also provides a strong rationale for investigating M9140 in combination with SoC agents (Kopetz 2024). Thus, Part 2C of the study will evaluate M9140 in combination with bevacizumab (Part 2C1) and bevacizumab plus capecitabine (Part 2C2) in Q3W administration in participants with advanced or metastatic CRC, and Part 2D of the study will evaluate M9140 in combination with 5-FU plus bevacizumab in [redacted] administration.

Alternative administration schedules, e.g., [redacted] might be evaluated in the future, to be implemented via a protocol amendment.

4.2.3 Rationale for Treatment of CRC with anti-CEACAM5 ADC

CEACAM5 is a very well characterized cancer antigen target and is highly expressed by several tumor types, in particular such as CRC, GC/GEJC, and NSCLC. M9140 is a novel anti-CEACAM5 ADC designed to have improved properties compared to approved ADCs with TOP1i payloads, such as trastuzumab deruxtecan (Enhertu®) and sacituzumab govitecan (Trodelvy®), which have demonstrated clinical activity in various indications.

There are currently no CEACAM5-targeted agents approved in CRC or GC/GEJC. Labetuzumab govitecan (IMMU-130), an anti-CEACAM5 ADC in which the antibody labetuzumab is conjugated to SN 38 showed some activity in irinotecan-refractory mCRC but there are currently no ongoing studies with this ADC (Criscitiello 2021). The development of tusamitamab ravtansine (formerly SAR408701, an ADC in which an anti-CEACAM5 antibody is conjugated to DM4, a maytansinoid cytotoxic agent) was discontinued in CRC indication because of its low sensitivity to microtubulin-inhibitors payload (Criscitiello 2021). A Phase 2 study of tusamitamab ravtansine in combination with ramucirumab in GC/GEJC is ongoing (NCT05071053).

Approximately 25% of patients with CRC are diagnosed with metastatic disease and 50% will develop metastasis (Van Cutsem 2014) and have a poor prognosis with a 5-year OS rate of only 15.7% (SEER 2014-2020) and a median OS of 24 to 36 months (Prasanna 2018). Patients with mCRC are currently treated with a combination of chemotherapy (e.g., fluoropyrimidine, irinotecan, oxaliplatin) and an anti-VEGF compound or anti-EGFR monoclonal antibody depending on RAS mutational status. RAS activating mutations (KRAS/NRAS) are present in 30% to 45% of mCRC and have proven to be a negative predictive biomarker of response to anti-EGFR (Sorich 2015).

Therapies such as regorafenib and trifluridine/tipiracil are recommended as 3L+ treatments for mCRC. The reported ORR is less than 5%, with a median OS benefit of only 1 to 2 months compared with placebo (Weinberg 2016). Trifluridine/tipiracil (FTD/TPI, TAS-102) is recommended in patients pretreated with fluoropyrimidines, oxaliplatin, irinotecan, and biologics if available or in earlier lines of therapy following oxaliplatin and irinotecan regimen failure (Xu 2018; Yoshino 2018). Another option is to administer cetuximab or panitumumab in patients with RASwt mCRC if they have not previously received it, which is rarely the case because they are indicated in 1L or 2L therapy (Fernández-Montes 2020). Encorafenib in combination with cetuximab is also approved for the treatment of adult patients with metastatic CRC with a BRAFV600E mutation, who have received prior systemic therapy, based on the results of the BEACON study (Tabernero 2021).

A further 3L alternative is to rechallenge with chemotherapy or biologicals previously discontinued owing to toxicity or PD (Masuishi 2020). High-quality evidence for this strategy is limited. Another option is to use specific treatments for very selected populations such as trastuzumab + lapatinib in mCRC HER2+, immunotherapy in MSI-H, or intrahepatic therapies in limited disease or primarily located in the liver. The main recommendation is to include patients in clinical trials (Minami 2001, Arnold 2018) and also as per NCCN Clinical Practice Guidelines recommendation for CRC (NCCN CRC 2024).

M9140 showed an acceptable safety profile and strong antitumoral activity leading to in vivo efficacy in various animal models representing a variety of solid tumors expressing CEACAM5 including but not limited to CRC and thus presents an attractive therapy for patients with limited therapeutic options and high unmet medical needs (Raab-Westphal 2024).

The high prevalence of CEACAM5 in patients with CRC (> 95% of patients exhibit high CEACAM5 expression; unpublished Sponsor data), justifies an all-comers approach for participants with advanced CRC, without the need for prospective CEACAM5 expression testing.

Clinical data from the dose escalation Part 1 of Study MS202329_0001 has demonstrated encouraging preliminary anti-tumor activity with a manageable safety profile consistent with exatecan toxicity, with no new unexpected AEs observed, in participants with heavily pretreated mCRC.

4.2.4 Participant Input into Design

Not applicable.

4.3 Justification for Dose

4.3.1 Part 1 – M9140 Dose Escalation

The proposed clinical study is a FIH Phase 1 dose escalation study with expansion cohorts aiming to explore the safety and tolerability of M9140 in participants with locally advanced or metastatic solid tumors known to express CEACAM5 who have limited therapeutic options. Therefore,

ICH S9 has been considered for the determination of the FIH starting dose, (i.e., a dose that is expected to have pharmacologic effects and is reasonably safe to use).

In addition, several published clinical trials with exatecan mesylate allow for human risk assessment of exatecan (Braybrooke 2003, De Jager 2000, Garrison 2003, Minami 2001, Rowinsky 2000, Royce 2001, Sharma 2001). Exatecan is gradually released in small amount from M9140 after iv infusion and is considered the driver for antigen-independent toxicity as observed mainly in the monkey toxicity studies with M9140 (up to 30 mg/kg).

Human risk assessment of exatecan mesylate and associated plasma exatecan exposure based on several clinical Phase 1 safety and PK studies with oncology patients is offering a safety margin of [redacted] against exatecan exposure at NOAEL of 24 mg/kg Q3W of M9140 in monkeys. No or limited hematotoxicity (Grade 1 or 2) was observed in these patients with cancer. In terms of the PK profile corresponding to the exatecan release from M9140, the study by Royce 2001 applying 24-h infusion Q3W may be regarded as the most representative clinical Phase 1 study with exatecan mesylate providing at least a [redacted] safety margin (against the exposure at the dose of 1.2 mg/m^2 Q3W exatecan). Therefore, the exposure to exatecan at NOAEL is considered as safe to start a clinical study with M9140 in advanced-stage oncology patients.

The NOAEL for M9140 in monkeys was determined at the highest applied dose of 24 mg/kg Q3W in the pivotal toxicity study. Since no HNSTD was determined, following the recommendations for assessing ADCs in oncology (Saber 2015) and ICH S9 (Q&A Step 5 Section 4.6), the initial clinical starting dose of [redacted].

[redacted].

Exatecan exposure in clinical study with exatecan at 24-h infusion at tolerated dose of 2.4 mg/m^2 Q3W (Royce 2001) and predicted unconjugated exatecan exposure at M9140 dose of 0.6 mg/kg: more than [redacted] exposure margin (AUC).

[redacted]

A preselected set of doses of 0.6, 1.2, 1.8, 2.4, and 3.0 mg/kg (subsequent escalation steps with 20% dose increases) are planned for dose escalation. The specific escalation steps and maximum dose will depend on the observed safety, tolerability, and other emerging data. At the predicted tumor regression dose of 2.33 mg/kg, the M9140 exposure (AUC) is predicted to be [redacted] lower than at the NOAEL in monkey, while unconjugated exatecan exposure is predicted to be [redacted]% lower than the average exposure at 2.4 mg/m^2 as described by Royce 2001. Hematological toxicities driven by unconjugated exatecan, e.g., neutropenia, are expected to determine the MTD and RDE. At the starting dose of 0.6 mg/kg, no clinically meaningful decrease in neutrophils is expected.

Current clinical experience indicates dose-related neutropenia that appears to be associated with the exposure of unconjugated payload exatecan, based on preliminary PK data. Information on dose calculations for each participant, including a dose cap for participants whose BMI is greater than 30 kg/m^2, is provided in Sections 4.1 and 4.2

4.3.2 Parts 2A, 2B, 2C, and 2D – Dose Expansion

Preliminary clinical data from the dose escalation Part 1 of the study have demonstrated an encouraging antitumor activity with participants in 2.8 mg/kg and 2.4 mg/kg dose levels experiencing PRs. At the cut-off date (20 February 2024), overall median PFS was 5.0 months (95% CI: 2.8, 8.4) and DCR was 62.5% (confirmed PR per RECIST v1.1: n=3 plus SD: n=22 including 1 unconfirmed PR, with overall n=40) (Sponsor data from end of dose escalation analysis). Moreover, preliminary clinical data from the dose escalation Part 1 of the study have demonstrated a manageable safety profile; the most frequent TEAEs were anemia, nausea, fatigue, white blood cell count decreased, neutrophil count decreased, vomiting, and platelet count decreased which were mostly low grade and manageable with supportive measures.

12 participants were treated at 2.8 mg/kg. 1 participant at this DL experienced an AE (sepsis) leading to death and the causality was attributed both to M9140 treatment as well as to the disease under study. The SMC evaluated the totality of safety data for this DL, including this participant, and declared this dose to be tolerable and safe for participants. No other M9140 related Grade 5 event was reported across all tested DLs.

4.3.2.1 Part 2A – M9140 Q3W Dose Optimization

The planned dose levels of M9140 for Part 2A are 2.4 mg/kg and 2.8 mg/kg, Q3W, based on preliminary clinical data from Part 1 of the study and subsequent PK/PD modeling analysis. The selection of the two doses was based on the preliminary PK, safety, tolerability, and antitumor activity data using a totality of evidence approach, supported by PK/PD analyses of hematological safety and tumor dynamics. Based on the integrated assessment of tumor growth inhibition using pharmacology studies in PDX mouse models and clinical data, a dose range of 2.4 mg/kg to 2.8 mg/kg was expected to result in tumor growth inhibition in patients by translational modeling.

In addition, PRs were observed in the dose escalation (Part 1) at the DL 2.4 mg/kg or higher. From a safety perspective, DLs ≥ 3.0 mg/kg (with and without primary G-CSF prophylaxis) were considered poorly tolerated. PK, efficacy, and safety data were leveraged in PK/PD analyses to increase our understanding of the therapeutic window of M9140.

Based on this integrated assessment, DLs 2.4 mg/kg and 2.8 mg/kg are expected to demonstrate antitumor activity with an acceptable safety profile and were selected to further evaluate the benefit/risk profile of M9140. Two doses will be assessed in a larger population, with a focus on dose and exposure-response analyses for safety and clinical activity endpoints, in order to support the determination of the RP2D of M9140 for this and future clinical trials.

4.3.2.2 Part 2B – M9140 [redacted] Regimen

Initial calculations of the doses using a [redacted] regimen were based on time-averaged dose intensity and enabled the selection of [redacted]. In addition, modeling and simulation analyses based on emerging data with a Q3W regimen in Parts 1 and 2A will be leveraged to support the [redacted] doses to be evaluated in Part 2B. By integrating PK/PD data, the models will enable dose-exposure-response assessments and inform the selection of an appropriate dose for the [redacted] regimen in order to achieve comparable antitumor activity (i.e. longitudinal tumor size) with similar acceptable toxicity (neutrophil counts and platelets).

4.3.2.3 Part 2C – M9140 Q3W in Combination with Bevacizumab (Part 2C1) or Bevacizumab plus Capecitabine (Part 2C2)

Bevacizumab is approved in combination with fluoropyrimidine-based chemotherapy for patients with mCRC at the dose of 7.5 mg/kg Q3W. For Part 2C1 dosing will start from the lower M9140 dose of 2.4 mg/kg (DL1) with bevacizumab at its approved dose of 7.5 mg/kg Q3W. If no safety and tolerability issues are observed, then the planned DL2 will be M9140 at 2.8 mg/kg and bevacizumab 7.5 mg/kg. Recent publications have demonstrated that combinations of other ADCs at their single agent recommended dose with bevacizumab at 7.5 mg/kg are feasible with no safety concerns (DESTINY Gastric-03 [Janjigian 2022] and TROPION PanTumor 03 [Janjigian 2023]).

The combination of TOP-1 inhibitors with capecitabine and bevacizumab is a well-established regimen used in the treatment of colorectal cancer (Xu 2018). Additionally, ADCs with TOP1

inhibitors as payload are currently being evaluated in combination with capecitabine plus bevacizumab (Janjigian 2023). In combination treatment, the recommended starting dose of capecitabine is 800 to 1,000 mg/m^2 when administered bid for 14 days followed by a 7-day rest period. Therefore, in Part 2C2, dosing will commence from DL1 with M9140 at the dose of 2.4 mg/kg Q3W, bevacizumab at its approved dose of 7.5 mg/kg Q3W and capecitabine at its lower dose of 800 mg/m^2 bid (D1-14, q21 days). If DL1 is not tolerable then DL-1 will be considered with M9140 at the dose of 2.0 mg/kg, bevacizumab at 7.5 mg/kg and capecitabine 800 mg/m^2 bid, given Q3W. If DL1 is deemed tolerable by the SMC, capecitabine dose can be escalated to 1,000 mg/m^2 in DL2 and M9140 dose can be escalated up to 2.8 mg/kg in the subsequent planned dose level (DL3).

4.3.2.4 Part 2D – M9140 [redacted] in Combination with 5-FU plus Bevacizumab

5-FU is a SoC agent used along with folinic acid in the treatment of colon and rectal cancers in a number of treatment regimens. Commonly used treatment regimens combine 5-FU and folinic acid with other chemotherapeutic agents such as irinotecan (FOLFIRI), oxaliplatin (FOLFOX) or both irinotecan and oxaliplatin (FOLFIRINOX) (Dekker 2019). The FOLFIRI regimen is administered [redacted] and typically consists of the following components: [redacted] (Heinemann 2014). The addition of bevacizumab to FOLFIRI is considered safe and efficacious (Aparicio 2018, Modest 2018).

The dose for [redacted] administration of M9140 monotherapy will first be evaluated in Part 2B. The intended starting dose for Part 2D (DL1) is [redacted]. If DL1 is not tolerable then lower starting dose for M9140 will be considered based on emerging data from Part 2B and 2C1. If DL1 is deemed safe and tolerable by the SMC, the next planned dose level is M9140 at a dose of [redacted]. If DL2 is deemed tolerable and depending on data from Part 2B and Part 2C1, in DL3, [redacted]. At all dose levels, infusion of 5-FU is always preceded by an infusion of folinic acid at 400 mg/m^2.

4.4 End of Study Definition

4.4.1 Part 1 – Dose Escalation

The end of the study is defined as the date when the last participant has completed the last Safety follow-up Visit after the last M9140 administration.

4.4.2 Part 2 – Dose Expansion

The end of study is defined as the date of study treatment discontinuation by all participants (due to disease progression, death, toxicity, withdrawal of consent/study, lost to follow-up or participants are not likely to benefit from the study intervention any longer)

AND

At least two thirds of the participants in Part 2A have completed Survival Follow-up for at least 12 months after first dose of M9140 or have died, withdrawn, or were lost to follow-up.

AND

All of the participants in Parts 2B, 2C, and 2D have completed Safety Follow-up Visit 30 (±3) days after last M9140 administration or have died, withdrawn, or were lost to follow-up.

A participant has completed the study if he/she has completed all study parts including the Safety Follow-up Visit 30 (±3) days after the last dose of M9140 administration and for Part 2A, including the Survival Follow-up up to 24 months after first dose or until study end.

The Sponsor may terminate the study at any time once access to M9140 for participants still benefiting is provided via a rollover study, expanded access, marketed product, or another mechanism of access as appropriate.

At the time of the Sponsor's decision to discontinue the trial early based on SMC recommendation or other reasons, Investigators, in collaboration with the Sponsor will evaluate if participants may still benefit from study treatment and will continue until appropriate mechanisms of access are available (as described above). However, in such an event, collection of further data from participants still on treatment (e.g., PRO questionnaires, PK/ADA samples and central imaging reads) and long-term Survival follow-up interviews may be stopped to reduce the burden for the participants.

5 Study Population

Part 1 – M9140 Dose Escalation

Participants with documented histopathological diagnosis of locally advanced or metastatic CRC, who were intolerant/refractory to, or progressed after systemic therapies in the advanced/metastatic setting that included and are restricted to a CCI [redacted]. Patients may have received prior treatment with CCI [redacted], if locally indicated and available to the patient. Participants with a known MSI-H status must have received treatment with an immune checkpoint inhibitor (if locally indicated and available) unless contraindicated. Japanese patients will be included in the dose escalation. See Appendix 11 for additional requirements in Japan. Low ethnic sensitivities are expected for M9140, based on literature data (Table 16):

- Monoclonal antibody-based therapies are not sensitive to ethnic factors and thus the antibody component is expected to have conserved PK and safety across populations (Chiba 2013).
- For approved tecan-based ADCs sacituzumab govitecan and trastuzumab deruxtecan, no clinically significant differences in PK were observed between Asian and non-Asian population. For sacituzumab govitecan the main driver for PK variability was due to genetic

variants of the UGT1A1 gene, but exatecan is not a UGT1A1 substrate. Mahmood 2021 described that no meaningful ethnic differences in PK were observed for 10 marketed ADCs.

- A Phase 1 and pharmacological study of exatecan mesylate (DX-8951f), infused over 30 min Q3W was conducted in Japan (Minami 2001). PK parameters, as well as safety and tolerability, were comparable to what has been reported from clinical trials in Caucasian populations.

Table 16 Comparison of Main PK Parameters for Exatecan Between Caucasian and Japanese Populations (Median, Range) Based on Published Data

	CL (L/h/m^2)	Vss (L/m^2)
**Caucasian**	1.86 (0.67 – 4.47)	16.5 (9.91 – 38.5)
**Japanese**	1.9 (1.6 – 2.8)	20 (20 – 25)

Median (range) clearance (CL) and volume of distribution (Vss) values were calculated based on published data (Ajani 2005, Boige 2000, Braybrooke 2003, Esteva 2003, Giles 2002, Minami 2001, Rowinsky 2000, Royce 2001, Royce 2004, Sharma 2001, Verschraegen 2004).

The SMC will review PK and safety data by race and the Bayesian logistic regression model analysis will include sensitivity analyses for Western-only and Japanese-only populations to assess consistency in dose-toxicity relationships across populations during the course of escalation and expansion.

Part 2 – Dose Expansion
Parts 2A, 2B, 2C, and 2D:

- Participants with documented histopathological diagnosis of locally advanced or metastatic CRC.
- Participants must have demonstrated progressive disease, according to RECIST v1.1, during or after the most recent treatment regimen. They must also have received at least 1 previous systemic treatment regimen in the advanced/metastatic disease setting but no more than 2.

  Exception: patients with MSI-H disease or BRAF positive disease are allowed to have had 3 previous regimens.
- Previous systemic regimens must have included a [redacted] (for participants with [redacted]), treatment with an immune checkpoint inhibitor (for participants with a known MSI-H status) and previous treatment with [redacted] (for participants with known BRAF gene mutations).

For full details of the study population, see Section 5.1.

See Appendix 11 for additional requirements in Japan.

The criteria in Sections 5.1 and 5.2 are designed to enroll only participants, who are appropriate for the study; thereby ensuring the study fulfills its objectives. All relevant medical and

nonmedical conditions are considered when deciding whether a participant is suitable for this study.

Prospective approval of protocol deviations to inclusion and exclusion criteria, also known as protocol waivers or exemptions, is not permitted.

Before performing any study assessments that are not part of the participant's routine medical care, the Investigator will confirm that the individual has provided written informed consent, as indicated in Appendix 2.

5.1 Inclusion Criteria

Participants are eligible to be included in the study only if all the following criteria apply:

Category	Criterion
Age	1. Are ≥ 18 years of age at the time of signing the informed consent. In Taiwan, participants who are ≥ 20 years of age and in South Korea, participants who are ≥19 years at the time of signing the informed consent.
Type of Participant and Disease Characteristics	2.<br>Part 1 – Dose Escalation: Participants with documented histopathological diagnosis of locally advanced or metastatic CRC, who were intolerant/refractory to or progressed after systemic therapies in the advanced/metastatic setting that included and are restricted to a [redacted], and an [redacted]. Participants may have received previous lines of treatment with [redacted], if locally indicated and available to the patient. Participants with a known MSI-H status must have received treatment with an immune checkpoint inhibitor (if locally indicated and available) unless contraindicated. See Appendix 11 for additional requirements in Japan.<br>Part 2 – Dose Expansion<br>**Parts 2A, 2B, 2C, and 2D**:<br>• Participants with documented histopathological diagnosis of locally advanced or metastatic CRC.<br>• Participants must have demonstrated progressive disease according to RECIST v1.1 during or after the most recent regimen.<br>• Participants must have received at least 1 previous systemic treatment regimen in the advanced/metastatic disease setting but no more than 2.

Category	Criterion
	Exception: patients with MSI-H disease or BRAF positive disease are allowed to have had 3 previous regimens.<br>• Previous systemic regimens must have included the following (provided no medical contraindication and agent is locally available):<br>○ CCI [redacted]<br>○ CCI [redacted].<br>○ treatment with an immune checkpoint inhibitor for participants with a known MSI-H status.<br>○ previous treatment with CCI [redacted] for participants with known BRAF gene mutations.<br><br>Notes:<br>• CCI [redacted].<br>• Participants may have previously received experimental treatments, as long as the maximum number of lines is respected, and no protocol-prohibited agents were administered.<br>• Changes of regimen components, due to unacceptable toxicity without signs of progression, will not be counted as a line.<br>See Appendix 11 for Japan-specific requirements.
Sex and Contraception/Barrier Requirements	3. All sexes allowed<br>The Investigator confirms that each participant agrees to use appropriate contraception and barriers, if applicable. The contraception, barrier, and pregnancy testing requirements are below.<br>Male participants:<br>• Agree to the following during the study intervention period and for at least 6 months after the last dose of study intervention:<br>○ Refrain from donating fresh unwashed sperm<br>PLUS EITHER:

Category	Criterion
	○ Abstain from any activity that allows for exposure to ejaculate<br>OR<br>○ Use a male condom<br>▪ When having sexual intercourse with a WOCBP, who is not currently pregnant, and instruct her to use a highly effective contraceptive method with a failure rate of < 1% per year, as described in Appendix 3, since a condom may break or leak.<br>▪ When engaging in any activity that allows for exposure to ejaculate.<br><u>Female participants:</u><br>• Are not pregnant (i.e., WOCBP have a negative serum or highly sensitive urine pregnancy test, as required by local regulations, within 24 h before the first dose of study intervention). If a urine test cannot be confirmed as negative (e.g., an ambiguous result), a serum pregnancy test is required.<br>• Are not a WOCBP.<br>• If a WOCBP, use a highly effective contraceptive method (i.e., with a failure rate of < 1% per year), preferably with low user dependency, as described in Appendix 3 for the following time periods:<br>○ Before the first dose of the study intervention(s). If using hormonal contraception:<br>1) Participant has completed at least one 4-week cycle of an oral contraception pill and either had or has begun her menses;<br>OR<br>2) Has used a depot contraceptive or extended-cycle oral contraceptive for at least 28 days and has a documented negative serum pregnancy test using a highly sensitive assay.<br>○ During the study intervention period.<br>○ After the study intervention period (i.e., after the last dose of study intervention is administered) for at least 9 months and agree not to donate eggs (ova, oocytes) for reproduction during this period.<br>• Women should not breastfeed during the study and for at least 3 months (Part 1, 2A, and 2B) or 6 months (Part 2C and 2D)

Category	Criterion
	after the study period, (i.e., after the last dose of any study intervention is administered).<br>• The Investigator evaluates the effectiveness of the contraceptive method in relationship to the first dose of any study intervention.<br>The Investigator reviews the medical history, menstrual history, and recent sexual activity to decrease the risk for inclusion of a female with an early undetected pregnancy.<br>See Appendix 11 for Japan-specific recommendations.
Informed Consent	4. Capable of giving signed informed consent, as indicated in Appendix 2, which includes compliance with the requirements and restrictions listed in the ICF and this protocol.
ECOG Performance Status	5. ≤ 1
Hematologic Function	6. Is adequate, as indicated by:<br>• Platelet count ≥ 100,000/mm^3 (no transfusion in the past 2 weeks before first dose)<br>• Hemoglobin ≥ 9.0 g/dL (no transfusion in the past 2 weeks before first dose)<br>• ANC ≥ 1,500/μL (no hematopoietic growth factors or G-CSF in the past 2 weeks before first dose)<br>• INR ≤ 1.5 × ULN
Hepatic Function	7. Is adequate, as defined by a total bilirubin level ≤ 1.5 × ULN, an AST level ≤ 2.5 × ULN, and an ALT level ≤ 2.5 × ULN<br>• For documented Gilbert's Syndrome, a total bilirubin < 3 × ULN is accepted<br>• For participants with liver metastases, AST and ALT < 5 × ULN is accepted
Renal Function	8. Is adequate, as defined by creatinine clearance of ≥ 60 mL/min by calculation using Cockcroft-Gault formula:<br>CrCl (mL/min) = {((140–age (years)) × weight (kg)) / (72 × Serum creatinine (mg/dL))} × 0.85 (if female)
Prior Therapy	9. Participants who received prior CT, RT (except limited local palliative RT), biological therapy (e.g., antibodies) or any other anticancer therapy or investigational drugs, must have a wash-out period of 21 days or 5x half-lives, whichever is shorter, before receiving the first dose of any study intervention.

Category	Criterion
**Other Inclusion Criteria for Part 1 only**	
	10. [redacted] concentration level ≤ 10,000 ng/mL.
Archival Tumor Tissue collection	11. Archival FFPE tumor tissue is required. If archived tumor material is not available, fresh biopsy is required.
**Other Inclusion Criteria for Part 2 only**	
Archival Tumor Tissue collection	12. Archival FFPE tumor tissue is required. If archived tumor material is not available, fresh biopsy is required.
Optional Fresh Tumor Biopsy (Part 2A and 2C only)	13. **For Parts 2A1, 2A2, 2C1 and 2C2:** Participants may decide to consent to collection of fresh biopsies. Fresh tumor biopsies will be obtained after participant's study eligibility has been confirmed and before participant receives first dose in the study.
Measurable lesion	14. Participant must have at least 1 lesion that is measurable using RECIST v1.1.

5.2 Exclusion Criteria

Participants are excluded from the study if any of the following criteria apply:

Category	Criterion
Medical Conditions	1. If AEs related to previous therapies have not recovered to Grade ≤1 by NCI-CTC v5.0 (except for lymphopenia, Grade 2 peripheral neuropathy, Grade 2 alopecia, Grade 2 lab abnormalities that are clinically not relevant, and Grade 2 AEs from prior immune checkpoint inhibitor therapy that are not relevant as an exclusion criterion per Investigator’s assessment [e.g., stable, substituted hypothyroidism]).
	2. Participant has a history of malignancy within 3 years before the date of enrollment (exceptions are squamous and basal cell carcinomas of the skin and carcinoma in situ of the cervix, benign prostate neoplasm/hypertropia, or malignancy that in the opinion of the Investigator, with concurrence with the Sponsor’s Medical Monitor, is considered cured with minimal risk of recurrence within 3 years).
	3. Participants with known brain metastases, except those meeting both of the following criteria:<br>a. All brain metastases have been treated locally and are clinically stable for at least 4 weeks prior to the start of treatment.<br>b. No ongoing neurological symptoms that are related to the brain localization of the disease (sequelae that are a consequence of the treatment of the brain metastases are acceptable).
	4. Participants with diarrhea (liquid stool) or ileus Grade > 1.
	5. Participants with active chronic inflammatory bowel disease (e.g., ulcerative colitis, Crohn’s disease, intestinal perforation) and/or bowel obstruction.
	6. Unstable angina, myocardial infarction, congestive heart failure (NYHA ≥ II) or a coronary revascularization procedure within 180 days of study entry. Calculated QTc average (using the Fridericia correction calculation) of > 470 ms.
	7. Cerebrovascular accident/stroke (< 6 months prior to enrollment).
	8. Active or prior ILD/pneumonitis. History of idiopathic pulmonary fibrosis, obliterative bronchiolitis, or idiopathic pneumonitis (history of prior resolved radiation pneumonitis allowed).

Category	Criterion
	9. Active symptomatic fungal, bacterial, and/or viral infection. Individuals with known or positive testing for HIV or actively infected viral hepatitis B (see Appendix 11 for Japan-specific measures regarding hepatitis B infection) or hepatitis C based on local standards of detection are excluded. Participants with hepatitis C, who have been treated with curative therapy are not considered actively infected. Participants with history of hepatitis C infection will be eligible for enrollment only if the viral load according to the local standards of detection is documented to be below the level of detection in the absence of anti-viral therapy during the previous 12 weeks (e.g., sustained viral response according to the local product label but no less than 12 weeks whichever is longer). Opportunistic infections and active COVID-19 infection. Testing for COVID-19 according to local medical practice.
	10. Uncontrolled concurrent illness (e.g., serious uncontrolled diabetes [fasted blood glucose > 250 mg/dL], symptomatic congestive heart failure, unstable angina pectoris, cardiac arrhythmia, or psychiatric illness/social situations that would limit compliance with the study requirements).
	11. Estimated life expectancy of < 4 months.
	12. Steroid therapy for antineoplastic intent taken < 7 days prior to the first dose of study intervention.
	13. Prior therapy targeting CEACAM5 (e.g., anti-CEACAM5 ADC) or an ADC with a TOP1i payload (e.g., trastuzumab deruxtecan) is not allowed.
	14. Participants currently receiving (or unable to stop using prior to the first dose of study intervention) prohibited medication (Section 6.8.3).
	15. Received growth factors (including EPO, darbepoetin, G-CSF, GM-CSF, and platelet stimulators [e.g., eltrombopag, romiplostim, or IL-11]) or transfusions within 2 weeks prior to the first day of study intervention.
	16. Major surgery within 4 weeks prior start of study interventional drug.
	17. History of severe hypersensitivity reactions to prior therapies with biologicals or excipient of M9140. See Appendix 11 for additional Japan-specific requirements.

Category	Criterion
Diagnostic Assessments	None
**Other Exclusion Criteria for Parts 2C and 2D only**	
Medical Conditions	18. Hemoptysis, thrombotic, or hemorrhagic event within the past 6 months prior to the first dose of study intervention.
	19. Participants under treatment with oral or parenteral anticoagulants or thrombolytic agents for therapeutic purposes, or evidence of bleeding diathesis or coagulopathy.
	20. Abdominal fistula, gastrointestinal perforation, or intra-abdominal abscess within the past 6 months prior to the first dose of study intervention.
	21. Unhealed wound following surgery, significant traumatic injury within 28 days prior to the first dose of study intervention, or an anticipated need for major surgery during the study.
	22. Uncontrolled hypertension (defined as either systolic blood pressure ≥ 140 mmHg or diastolic blood pressure ≥ 90 mmHg), even after indicated antihypertensive treatment.
	23. Participants with proteinuria > 2 g/24 hours
	24. History of hypersensitivity to bevacizumab or its excipients
**Other Exclusion Criteria for Parts 2C2 and 2D only**	
Medical Conditions	25. Known dihydropyrimidine dehydrogenase (DPD) deficiency.
	26. History of hypersensitivity to:<br>• capecitabine or its excipients (Part 2C2 only)<br>• 5-FU and/or folinic acid or their excipients (Part 2D only)
	27. Participants with rare hereditary problems of galactose intolerance, total lactase deficiency or glucose-galactose malabsorption (Part 2C2 only)
Prior therapy	28. Recent (within 4 weeks prior to the first dose of study intervention) or concomitant treatment with brivudine, sorivudine or their analogues.

5.3 Lifestyle Considerations

5.3.1 Meals and Dietary Restrictions

Participants will be instructed to refrain from consumption of St. John's wort, as well as any other CYP3A4 inhibitors/inducers such as herbal supplements (e.g., essiac tea) and foods or drinks with CYP3A4 inhibition potential (i.e., grapefruits and grapefruit juice, Seville oranges, pomelos, starfruits) starting at least 7 days before the first administration of the any study intervention and during the study intervention (see Appendix 7 for details).

5.3.2 Caffeine, Alcohol, Tobacco, and Cannabinoid

- During each dosing period, participants will abstain from ingesting caffeine- or xanthine-containing products (e.g., coffee, tea, cola drinks, and chocolate) for 12 hours prior to ECG and vital sign assessments.
- During each dosing period, participants will abstain from alcohol and cannabinoid-containing products for 12 hours prior to ECG and vital sign assessments.
- Participants who use tobacco products will be instructed that use of nicotine-containing products (including nicotine patches) will not be permitted while they are in the clinical unit.

5.3.3 Activity

Participants will abstain from strenuous exercise for 4 hours before each blood collection for clinical laboratory tests. Participants may participate in light recreational activities (e.g., watching television or reading).

5.3.4 Other Lifestyle Considerations

Participants are advised to avoid prolonged direct sunlight exposure while participating in the study. Participants are also encouraged to take protective measures such as applying broad-spectrum sunscreens (on exposed body parts) and lip-balm, wearing UV-protective clothing, hats, and sunglasses when outdoors.

5.4 Screen Failures

Individuals who do not meet the criteria for participation in this study (screen failure) may be rescreened 1 time. Rescreened participants will be assigned a new participant number.

6 Study Intervention and Concomitant Therapies

Study intervention is any investigational intervention(s), marketed product(s), placebo, or medical device(s) intended to be administered to a study participant per the study protocol.

6.1 Study Intervention Administration

Table 17 Study Intervention Administered in Part 1A and Part 1B

**Arm Name**	**M9140 Q3W Part 1A and Part 1B**	**M9140 Q3W Part 1B**
**Arm Type**	Experimental	Experimental
**Intervention Name**	M9140	Pegfilgrastim (or pegfilgrastim biosimilars)
**Type**	Antibody-drug conjugate	Granulocyte colony stimulating factor
**Dose Formulation**	[redacted]	[redacted]
**Unit Dose Strength(s)**	[redacted] mg/mL	[redacted] mg/mL
**Dose Amount**	Dose escalation study starting at [redacted] mg/kg	[redacted]
**Frequency**	Every 3 weeks (Q3W).	On day 8 (±3 days) in the first 2 cycles
**Route of Administration**	Intravenous	Subcutaneous
**Use**	Experimental	Auxiliary
**IMP or NIMP/AxMP**	IMP	AxMP
**Sourcing**	Provided centrally by the Sponsor	Sourced locally from the hospital pharmacies of the clinical sites or supplied by Sponsor.
**Packaging and Labeling**	Study Intervention will be provided in [redacted]. Each vial will be labeled per country-specific requirement(s) (see Appendix 11)	N/A
**Authorization status of IMP**	M9140 (IMP) is not yet authorized in any country worldwide	Authorized

Table 18 Study Interventions Administered in Part 2A, Part 2B, Part 2C, and Part 2D

Arm Name	Part 2A, Part 2B, Part 2C, and Part 2D	Part 2C and Part 2D	Part 2C	Part 2D	Part 2D
**Arm Type**	Experimental	Experimental	Experimental	Experimental	Experimental
**Intervention Name**	M9140	Bevacizumab (including biosimilars)	Capecitabine (including generics)	5-FU	Folinic acid
**Type**	Antibody-drug conjugate	Antibody	Chemotherapy	Chemotherapy	Folic acid derivative
**Dose Formulation**	[redacted]	Injectable solution for iv infusion	Oral tablet	Injectable solution for iv infusion	According to product information
**Unit Dose Strength(s)**	[redacted] mg/mL	[redacted] mg/mL	[redacted] mg tablets	Refer to corresponding local prescribing information or Package inserts for more information.	Refer to corresponding local prescribing information or Package inserts for more information.
**Dose Amount**	Up to a maximum of 2.8 mg/kg	7.5 mg/kg in Part 2C or [redacted] mg/kg in Part 2D	800 mg/m^2 or 1,000 mg/m^2<br>Dosing should be rounded to the nearest available tablet strength to enable delivery of a measurable dose	[redacted] mg/m^2 or [redacted] mg/m^2	[redacted] mg/m^2
**Frequency**	Q3W in Part 2A and Part 2C or [redacted] in Part 2B and Part 2D	Q3W in Part 2C or [redacted] in Part 2D	Twice daily for 14 days followed by a 7-day rest period	[redacted]	[redacted]
**Route of Administration**	Intravenous	Intravenous	Oral	Intravenous	Intravenous
**Use**	Experimental	Background intervention/SoC	Background intervention/SoC	Background intervention/SoC	Background intervention/SoC
**IMP or NIMP/AxMP**	IMP	AxMP/IMP depending on local regulation	AxMP/IMP depending on local regulation	AxMP/IMP depending on local regulation	AxMP/IMP depending on local regulation

Arm Name	Part 2A, Part 2B, Part 2C, and Part 2D	Part 2C and Part 2D	Part 2C	Part 2D	Part 2D
**Sourcing**	Provided centrally by the Sponsor	Provided locally from the hospital pharmacies of the clinical sites or centrally by Sponsor/CRO or designee	Provided locally from the hospital pharmacies of the clinical sites or centrally by Sponsor/CRO or designee	Provided locally from the hospital pharmacies of the clinical sites or centrally by Sponsor/CRO or designee	Provided locally from the hospital pharmacies of the clinical sites or centrally by Sponsor/CRO or designee
**Packaging and Labeling**	Study Intervention will be provided in [redacted]. Each vial will be labeled per country-specific requirement(s) (see Appendix 11)	Depending on the local regulations, bevacizumab may either be sourced from a local hospital pharmacy or supplied by the Sponsor (or designated service provider) and will be packaged/ labeled per all applicable regulatory requirements and GMP guidelines	Depending on the local regulations, capecitabine may either be sourced from a local hospital pharmacy or supplied by the Sponsor (or designated service provider) and will be packaged/labeled per all applicable regulatory requirements and GMP guidelines	Depending on the local regulations, 5-FU may either be sourced from a local hospital pharmacy or supplied by the Sponsor (or designated service provider) and will be packaged/labeled per all applicable regulatory requirements and GMP guidelines	Depending on the local regulations, folinic acid may either be sourced from a local hospital pharmacy or supplied by the Sponsor (or designated service provider) and will be packaged/labeled per all applicable regulatory requirements and GMP guidelines
**Authorization status of IMP**	M9140 (IMP) is not yet authorized in any country worldwide	Authorized	Authorized	Authorized	Authorized

M9140 should be given as an iv infusion (approximately 1 hour infusion duration) on Day 1 of each cycle.

Bevacizumab (Part 2C and 2D) should be given as an iv infusion at least 30 minutes after completion of M9140 administration. The initial dose should be delivered over 90 minutes. If the first infusion is well tolerated, the second infusion may be administered over 60 minutes. If the 60-minute infusion is well tolerated, all subsequent infusions may be administered over 30 minutes. It should not be administered as an intravenous push or bolus. If the participant was previously exposed to bevacizumab with no tolerability issues during infusion, the initial infusion time can be reduced to 30 minutes.

Capecitabine (Part 2C2) will be administered orally bid (within 30 minutes after meals) from the evening after completion of M9140 administration on Day 1 to the morning of Day 15.

For Part 2D, infusion of M9140 and bevacizumab will be followed by iv infusion of folinic acid over 120 minutes. Afterwards, continuous infusion of 5-FU over 46 hours will be started, using a portable pump.

Body surface area for capecitabine, folinic acid and 5-FU dosing will be calculated using the Mosteller formula (Mosteller 1987):

$$BSA\ [m^2] = \sqrt{\frac{height\ [cm] \times weight[kg]}{3600}}$$

6.2 Study Intervention Preparation, Handling, Storage, and Accountability

The Investigator, institution, or the head of the medical institution (where applicable) is responsible for study intervention accountability, reconciliation, and record maintenance (i.e., receipt, reconciliation, and final disposition records).

- Upon receipt of the study intervention(s), the Investigator or designee will confirm appropriate temperature conditions have been maintained during transit and any discrepancies are reported and resolved before use. Also, the responsible person will check for accurate delivery. Further guidance and information for study intervention accountability are provided in the Pharmacy Manual.
- Only participants enrolled in the study may receive study intervention(s) and only authorized site staff may supply it. All study intervention(s) will be stored in a secure, environmentally controlled, and monitored (manual or automated) area, per the labeled storage conditions, and with access limited to the Investigator and authorized site staff.
- M9140 drug product is a CCI [redacted] diluted prior administration by clinical site personnel who are

appropriately trained on the procedure. Further guidance and information for drug product preparation and administration are provided in the Pharmacy Manual.

- Dispensing will be recorded on the appropriate accountability forms so that accurate records will be available for verification at each monitoring visit.
- Study intervention(s) accountability records at the study site will include the following:
  - Confirmation of receipt, in good condition and in the defined temperature range.
  - The inventory provided for the clinical study and prepared at the site.
  - The dose(s) each participant used during the study.
  - The disposition (including return, if applicable) of any unused study intervention(s).
  - Dates, quantities, batch numbers, medication [redacted] numbers, expiry dates, and the participant numbers.
- The Investigator's site will maintain records, which adequately documents that participants were provided the doses specified in this protocol, and all study intervention(s) provided were fully reconciled.
- Unused study intervention(s) will not be discarded or used for any purpose other than the present study. No study intervention that is dispensed to a participant may be re-dispensed to a different participant.
- A Study Monitor will periodically collect the study intervention(s) accountability forms.
- Further guidance and information for the final disposition of unused study intervention(s) are provided in the Pharmacy Manual.

6.3 Measures to Minimize Bias: Study Intervention Assignment and Blinding

6.3.1 Study Intervention Assignment

This study will use an IRT (called Cenduit®). The IRT will be used to assign unique participant numbers and allocate study intervention to participants at each study intervention visit. Before the study is initiated, the directions for the IRT will be provided to each site. The site will contact the IRT prior to starting study intervention administration for each participant.

In the dose escalation, assignment to Part 1A (without pegfilgrastim) or Part 1B (with pegfilgrastim) will be sequential to the cohorts open by SMC decision.

In the dose expansion (Part 2A) for Arms A1 and A2, after confirmation of participant's eligibility and at the last practical moment prior to study intervention administration, participants will be centrally allocated to either 2.8 mg/kg of M9140 or 2.4 mg/kg of M9140 in a 1:1 ratio, stratified by BMI (BMI $\leq$ 30 vs BMI $>$ 30) to ensure a balanced ratio of participants with a dosing cap due to their BMI across both arms, using an IRT randomization system and per a computer-generated randomization list.

6.3.2 Blinding

The study intervention administration is open label and none of the assessments are blinded. Aggregated data analyses will be only provided at planned interim analyses.

6.3.3 Emergency Unblinding

Not applicable.

6.4 Study Intervention Compliance

In this study, participants will receive M9140 at the investigational site on Day 1 of each cycle.

For Part 2C1, participants will receive M9140 and bevacizumab at the investigational site on Day 1 of each cycle.

For Part 2C2, participants will receive M9140 and bevacizumab at the investigational site on Day 1 of each cycle. Capecitabine will be self-administered at home (in case participants are hospitalized, capecitabine will be administered in the hospital). The first dose will be taken in the evening of D1. On the following days capecitabine has to be taken twice daily for 14 days with a last tablet on Day 15 in the morning, followed by a 7-day rest period. Capecitabine has to be taken within 30 minutes after a meal and swallowed whole with water. When participants are dosed at the site, they will receive study intervention directly from the Investigator or designee, under medical supervision. The date and time of each dose administered in the clinic will be recorded in the source documents and in the eCRF. The dose of study intervention and study participant identification will be confirmed at the time of dosing by a member of the study site staff other than the person administering the study intervention.

Participants will be instructed by the investigator/designee regarding off-site self-administration of capecitabine and asked to record self-administration in a dosing diary. When participants self-administer at home, compliance with study intervention will be assessed at each visit. Compliance will be assessed by reviewing the dosing diary, direct questioning, and counting returned tablets during the site visits and document it in the source documents and eCRF. Any deviation(s) from the prescribed dosage regimen are recorded in the eCRF. A record of the number of tablets dispensed to and taken by each participant will be maintained and reconciled. Capecitabine start and stop dates, missed doses, and dose reductions will also be recorded in the eCRF. For specified timepoints the exact time of capecitabine administration will be recorded as well. The Investigator will ensure that the information entered into the eCRF regarding capecitabine administration is accurate for each participant. Any reason for noncompliance should be documented.

For Part 2D, participants will receive M9140, folinic acid and bevacizumab at the investigational site on Day 1 of each cycle. 5-FU will be administered via a portable pump as continuous infusion over 46 hours according to local institution guidelines, which will be started at the investigational site on Day 1 of each cycle.

6.5 Dose Modification

6.5.1 Retreatment Criteria

Not applicable.

6.5.2 Dose Selection

Part 1: The SMC will recommend proceeding to the next higher dose level (including the decision to which dose level), or decreasing the dose in the next cohort, or expanding the current dose level to include additional participants based on safety, tolerability, and available PK data. At least two participants in each cohort should have received ≥80% of the actual non-capped dose before recommending to proceed to a higher dose level. The SMC receives outputs of a Bayesian dose-toxicity model with estimated DLT probabilities for potential next dose levels to support their recommendation. The Bayesian model will be based on prior information, observed number of evaluable participants and number of participants with DLT, considering participants with a capped dose in their assigned (non-capped) dose level. As sensitivity analyses, the SMC will in addition receive results from the same Bayesian model using data from only Japanese and only Western participants, respectively. For participants whose absolute dose was capped due to their BMI, the SMC will in addition receive results from the same Bayesian model, where capped participants are considered in the dose level (in mg/kg) that matches their actual received (capped) dose (in mg). The SMC will also recommend the starting dose of the Part 1B (with pegfilgrastim) dose escalation. The same decisions using the same procedures as outlined above for Part 1A (without pegfilgrastim) dose escalation will be applicable.

Further details on the Bayesian model are provided in Section 9.4.2.1.

Part 2A: The SMC will receive outputs of Arm A1 and Arm A2 at selected timepoints (see Section 6.5.3). At each timepoint, the SMC will evaluate the safety, efficacy, and available PK data to recommend on the continuation of a cohort or dose level group(s). The SMC in collaboration with the Sponsor will evaluate the totality of safety, efficacy, and PK data at the final analysis for the selection of the RP2D for further development (Part 2A) (for details on dose selection analysis see Section 9.2.2). Selection of the RP2D for further development remains however at the discretion of the Sponsor. The SMC may recommend by consensus on continuation at the same dose, change in dose or stop of expansion arms. Decision making of the SMC will be supported by the results of a Bayesian 2-parameter logistic model (the same model as for Part 1).

Parts 2B, 2C, and 2D: The SMC will recommend expanding the current dose level to include additional participants, proceeding to a higher dose level of M9140, capecitabine, or 5-FU (including the decision to which dose level), or decreasing the dose of M9140, capecitabine, or 5-FU in the next cohort, based on safety, tolerability, and available PK data. At least 2 participants in each cohort should have received ≥80% of the actual non-capped M9140 dose before recommending proceeding to a higher dose level. The SMC receives outputs of a Bayesian dose-toxicity model (Part 2B), from an iBOIN design (Part 2C1) or from a CRM model (Part 2C2

and Part 2D) with recommendations for potential next dose levels to support their recommendation. Further details on the statistical methods are provided in Section 9.4.2.2.

Further details on the SMC are in Section 6.5.3.

6.5.3 Safety Monitoring Committee

The SMC consists of core (internal and external voting) members for the Sponsor (Global Patient Safety Product Leader, Medical Responsible, Clinical Pharmacologist and Biostatistician), and the Coordinating Investigator. Ad hoc members may be invited as needed.

During the dose escalation part (both Part 1A and Part 1B) of the study, the SMC will evaluate the safety (including DLTs) and available PK data. The SMC will decide on dose escalation, de-escalation, additional enrollment on the same dose level, MTD, and suspension of enrollment. In cases where enrollment of the last participant in a dosing cohort was delayed, the SMC may decide (based on available data) on the enrollment and dose for the next dosing cohort before all participants in a cohort have completed the DLT period. For this participant, the SMC will consider all available data and any subsequent emerging data at a subsequent meeting. An ad hoc meeting will be convened if this participant experiences a DLT. The SMC can recommend modifying the schedule of administration.

The SMC will review PK and safety to determine if adjustment of schedule is appropriate during the dose escalation based on the collected data. Based on the observed toxicity profile and available PK, dose level(s) that are different than, or higher or lower than the prespecified doses may be tested (see Section 9.4.2).

The SMC may also recommend changing the dose escalation schedule for Q3W based on all available safety, tolerability, PK and biological active dose range data. The SMC may also propose altering the timing of primary prophylactic pegfilgrastim administration based on accumulating safety data.

During the expansion part of the study (Part 2), for Part 2A, SMC meetings will be held to evaluate the safety and tolerability of M9140 based on all available safety, tolerability, and PK data after 8 and 15 participants for each Arm A1 and Arm A2 have completed the first cycle of therapy or dropped out. Additional SMCs can be held as needed. Medical monitoring will include observation of AEs meeting the DLT criteria as described in Section 6.5.4. Enrollment will continue during preparation of the SMC.

For Parts 2B, 2C, and 2D of the study, the SMC will evaluate the safety (including DLTs) and available PK data. The SMC will decide on dose escalation, de-escalation, additional enrollment on the same dose level, and suspension of enrollment. In cases where enrollment of the last participant in a dose level was delayed, the SMC may decide (based on available data) on the enrollment and dose for the next dose level before all participants in a cohort have completed the DLT period. For this participant, the SMC will consider all available data and any emerging data at a subsequent meeting. An ad hoc meeting will be convened if this participant experiences a DLT. The SMC can recommend modifying the schedule of administration. Enrollment will be halted during preparation of the SMC.

The usual planned cohort size is 3 participants for Part 2B, and 2C, and 2D dose levels, but the SMC can decide to change the size of cohorts. Parallel screening and enrollment of participants may allow multiple eligible participants to enroll at about the same time, which may therefore increase the cohort size to 4 participants. Any additional participants enrolled in the study at a given dose level, will complete all study procedures and assessments as per protocol. An SMC meeting with selection of dose for the next cohort will also be held if not all participants in a cohort are evaluable. The Sponsor upon SMC recommendation may decide to add more participants in any cohort based on emerging data from Part 2A, not exceeding the pre-specified maximum number of participants in each study Part.

For all parts, if a situation of unacceptable toxicity occurs (as specified below), there will be an ad hoc SMC meeting and the SMC will decide whether the dose for newly enrolled participants should be lowered (including to what dose).

An ad hoc SMC meeting will be convened to advise on whether a participant, a DL, or a study part should be discontinued in case:

- 2 or more DLTs occur within the first cycle of treatment at any dose level (applicable for Parts 2B and 2C).
- any of the administered doses exceeds the MTD conditions defined for the Bayesian model (e.g. if the median estimated DLT probability exceeds 30%; see Appendix 8), applicable for Part 2A. The same DLT criteria as for the dose escalation will apply.
- of an occurrence of a Grade 5 toxicity by the NCI-CTCAE v.5.0, not clearly attributable to underlying disease or extraneous circumstances (applicable for all parts).

Enrollment will be halted during preparation of the ad hoc SMC.

To ensure adequate representation of Caucasian and Asian population, reasonable effort will be made to ensure that an adequate number of participants will be enrolled in all cohorts from Asian countries. Based on safety observations and SMC recommendation the Sponsor may decide to expand enrollment in one or more cohorts to ensure adequate representation of participants from all geographical areas participating in the study.

The specific working procedures will be described in an SMC charter, which will be established before first informed consent signed.

6.5.4 Definition of Dose-limiting Toxicity

A DLT is defined as any of the following AEs according to the NCI-CTCAE v5.0 assessed by the Investigator or the Sponsor at any dose and judged not to be related to the underlying disease or any previous or concomitant medication or concurrent condition occurring during the DLT observation period (21 days for Part 1 and Part 2C, and 28 days for Parts 2B and 2D).

Participants who develop a DLT will discontinue study intervention based on Investigator's decision and Sponsor's Medical Monitor agreement.

AEs judged as DLT will include:

- Any Grade ≥ 3 **non-hematologic** AE with **exception** of:
  - Laboratory values out of normal range that have no clinical correlate or asymptomatic Grade ≥ 3 lipase, or amylase elevation not associated with clinical manifestation of pancreatitis.
  - IRR resolving within 6 h from the end of infusion and controlled with medical management.
  - Grade 3 diarrhea persisting ≤ 72 h after initiation of medical management.
  - Nausea and vomiting of ≤ 72 h duration with adequate and optimal therapy.
  - Transient (≤ 72 h) fatigue, local reactions, flu-like symptoms, Grade 3 fever, headache, Grade 3 hypertension that resolves to Grade ≤ 1 with adequate treatment.
  - Grade 3 non-recurrent skin toxicity that resolves to Grade ≤ 1 in less than 7 days after initiation of medical management.
  - Tumor flare phenomenon defined as local pain, irritation, or rash localized at sites of known or suspected tumor that resolve to Grade ≤ 2 within 6 days.
  - Any death due to the underlying disease or extraneous causes.
- Any Grade 4 non-hematologic life-threatening event is a DLT.
- Any Grade ≥ 4 hematologic AE, and in addition:
  - Grade ≥ 3 neutropenia with clinical signs/symptoms, such as fever > 38.5°C (e.g., for febrile neutropenia, an ANC < 1000/mm^3 with single temp of 38.3°C [101°F] or a sustained temperature of ≥ 38°C [100.4°F] for > 1 h).
  - Grade 3 thrombocytopenia with medically concerning bleeding.

  Except:
  - Isolated Grade 4 lymphopenia without clinical correlate.
  - Any Grade 4 neutropenia of <7 days duration not associated with any clinical symptoms or fever.
  - Any Grade 4 thrombocytopenia of <7 days duration not associated with any clinical symptoms or clinically significant bleeding.
- Evidence of hepatocellular toxicity without a clear alternative reason to explain the observed liver-related laboratory abnormalities, such as increase in AST or ALT of ≥ 3 x ULN elevation and elevation of serum total bilirubin ≥ 2 x ULN, without initial findings of cholestasis (elevated serum ALP) or other apparent clinical causality (e.g., viral hepatitis A, B, C, or comedication), per Hy’s Law definition, will be reported as an SAE.
- Additionally, the SMC may recommend as a DLT: a TEAE (inside or outside of the DLT period) that in the SMC’s opinion is of potential clinical significance such that further dose escalation would expose participants to unacceptable risk.

- The treatment with transfusions of blood cells or hematopoietic growth factors (e.g., G-CSF, erythropoietin) is allowed according to clinical need and physicians discretion. Cases of participants that were transfused or were given hematopoietic growth factors during the DLT period but for which the underlying cause does not constitute a DLT by the SMC decision are considered ICEs that will be handled according to composite strategy. This means that participants with such an ICE will be considered to have experienced a DLT in the Bayesian model. Mandatory prophylactic pegfilgrastim administrations do not constitute a DLT.

Any DLT must be confirmed by the SMC.

6.5.5 Dose Modification

Individual dose modifications, interruptions, or discontinuations are listed in:

- Table 19 for M9140
- Table 20 for capecitabine (Part 2C2)
- Table 21 for bevacizumab (Part 2C and 2D)
- Source: Bevacizumab local prescribing information.
- Table 22 for 5-FU (Part 2D)

Physicians may diverge from these recommendations if clinically indicated according to their clinical judgment after discussion with the Sponsor.

Before starting a new treatment cycle, all toxicities related to study treatment must have resolved according to Table 19, Table 20, Table 21, and Table 22, (except for those toxicities considered by the treating Investigator to be unlikely to become serious or life-threatening).

If the toxicities do not resolve during the given cycle according to Table 19, Table 20, Table 21, and Table 22, the start of the next cycle must be delayed for a maximum of 28 days from the scheduled start date of the next cycle. If more than 28 days are needed to recover, the participant will be withdrawn from treatment as described in the Section 7.1.

For M9140, participants who experience toxicities as described in Table 19 should have the dose reduced to the next lower level tested and deemed tolerable by the SMC (Parts 2B, 2C, and 2D). For participants who are treated at the lower dose level tested and experience toxicities, dose should be modified as per Table 19.

In Parts 2C and 2D, if at the beginning of a treatment cycle, a treatment delay is indicated for either M9140 or another agent in the combination regimen, then administration of all therapy should be delayed until the requirements for restarting all study interventions are met.

- Part 2C2: Treatment interruptions are regarded as lost treatment days for capecitabine and missed doses should not be replaced; the planned treatment schedule should be maintained. Once the dose of capecitabine has been reduced, it should not be increased at a later time.

- Part 2C2: If unscheduled laboratory assessments during a treatment cycle show that the neutrophil count drops below 1.0 x 10^9/L or that the platelet count drops below 75 x 10^9/L, treatment with capecitabine should be interrupted.
- Part 2C2: If the calculated creatinine clearance decreases during treatment to a value below 30 mL/min, capecitabine should be discontinued.
- If, in the opinion of the Investigator, the toxicity is clearly related to one of the treatments, reduction of one and not the other agent is appropriate. If, in the opinion of the Investigator, the toxicity is related to M9140, only the dose of M9140 should be reduced and if it is related to capecitabine or 5-FU, only the dose of capecitabine or 5-FU should be reduced (see Table 19 for M9140, Table 20 for capecitabine, and Source: Bevacizumab local prescribing information.
- Table 22 for 5-FU). However, if the toxicity is related to the combination of two agents, doses for both agents should be reduced, interrupted, or discontinued according to the recommended dose modifications.
- Dose reduction for adverse events is not recommended for bevacizumab. In case of toxicity that is considered related to bevacizumab and in the opinion of the Investigator bevacizumab continuation may put participant's safety at risk, bevacizumab should be permanently discontinued or temporarily suspended as described in the local prescribing information and Table 21.
- Participants may have capecitabine, 5-FU, and/or bevacizumab discontinued and continue M9140 alone, if this is deemed beneficial for the participant according to the Investigator's clinical judgment. If a participant needs to discontinue M9140 then the participant should be discontinued from the study.

In all the parts, for patients who participate in the lower projected dose level only one dose reduction is allowed.

Table 19 Dose Modifications and Temporary/Permanent Treatment Discontinuation of M9140

**Specifics**	**Toxicity (NCI-CTCAE v5.0 Grade)**	**Define criteria with permanent discontinuation or dose interruption and modification**
**Hematological Toxicity**		
**Neutrophils count decreased:** < 1000 – 500/mm^3; < 1.0 – 0.5 x 10e9/L).	Grade 3	Withhold treatment until a resolution or recovery to ≤ Grade 2 (G-CSF may be used in accordance with institutional guidelines).<br>Upon recovery restart treatment at current dose level consider treatment with G-CSF in accordance with institutional guidelines.

Specifics	Toxicity (NCI-CTCAE v5.0 Grade)	Define criteria with permanent discontinuation or dose interruption and modification
**Neutrophils count decreased:** (< 500/mm^3; < 0.5 x 10e9/L).	Grade 4	**First occurrence:** Withhold treatment until recovery to ≤ Grade 2 and consider treatment with G-CSF.<br>Upon recovery restart treatment at the same dose level and consider G-CSF treatment.<br>**In case of first recurrence:** reduce to the next lower dose level. For participants at the lower DL: reduce M9140 by 25%.<br>**In case of a second recurrence:** Each case will be discussed between Investigator and Sponsor.
**Febrile neutropenia:** (i.e., ANC < 1000/mm^3 with single temperature of 38.3°C [101°F] or a sustained temperature of ≥ 38°C [100.4°F] for >1 hour).	Grade 3-4	**First occurrence:** Withhold treatment until complete resolution.<br>Upon recovery, restart treatment at current dose and consider G-CSF support.<br>**In case of first recurrence:** reduce to the next lower dose level.<br>For participants at the lower DL: reduce M9140 by 25%.<br>**In case of a second recurrence**: permanently discontinue treatment.
**Anemia**: Hgb < 8.0 g/dL; < 4.9 mmol/L; < 80 g/L; transfusion indicated.	Grade 3	**First occurrence:** Withhold treatment until recovery to ≤ Grade 2.<br>Upon recovery, restart treatment at current dose.<br>**In case of first recurrence:** reduce to the next lower dose level.<br>For participants at the lower DL: reduce M9140 by 25%.<br>**In case of a second recurrence:** permanently discontinue treatment.
**Anemia**: Life-threatening consequences, urgent intervention indicated.	Grade 4	**First occurrence:** Withhold treatment until recovery to ≤ Grade 2 then reduce to the next lower dose level.<br>For participants at the lower DL: reduce M9140 by 25%.<br>**In case of (first) recurrence:** permanently discontinue treatment.
**Thrombocytopenia without clinically significant bleeding.**	Grade 3<br>or<br>Grade 4 < 7 days	In case of asymptomatic Grade 3 or an asymptomatic Grade 4 event lasting < 7 days withhold treatment until recovery to ≤ Grade 2.
**Thrombocytopenia with clinically significant bleeding.**	Grade ≥ 3	**First occurrence:** In cases of Grade ≥ 3 that are associated with significant bleeding withhold treatment until recovery to ≤ Grade 2 and reduce to the next lower dose level.<br>For participants at the lower DL: reduce M9140 by 25%.<br>**In case of (first) recurrence**: permanently discontinue treatment.
**Long-lasting thrombocytopenia** with platelet count < 25,000/mm3 **without clinically significant bleeding.**	Grade 4 ≥ 7 days	**First occurrence:** For Grade 4 thrombocytopenia events with no complications that last ≥ 7 days, withhold M9140 until recovery to ≤ Grade 2 and consider reducing to the next lower DL.<br>For participants at the lower DL: reduce M9140 by 25%.<br>**In case of (first) recurrence**: permanently discontinue treatment.

Specifics	Toxicity (NCI-CTCAE v5.0 Grade)	Define criteria with permanent discontinuation or dose interruption and modification
**Non-hematological**		
**Diarrhea/Colitis**: Increase of ≥7 stools per day over baseline with complications (bloody stool, severe abdominal pain, dehydration, peritoneal signs that require hospitalization).	Grade 3	Withhold treatment until recovery to ≤ Grade 1.<br>If resolved within 2 weeks from the day of onset reduce to the next lower dose level. For participants at the lower DL: reduce M9140 by 25%.<br>If not resolved within 2 weeks from the day of the onset or there is a recurrence, consider permanent discontinuation.<br>Appropriate anti-diarrheal therapy must be initiated immediately in accordance with institutional guidelines.
**Diarrhea/Colitis:** Life-threatening consequences, urgent intervention indicated.	Grade 4	Permanently discontinue M9140.
**Other non-hematological toxicities (except GI)**	Grade 2	Decide if type of AE requires withholding treatment until recovery to ≤ Grade 1.
	Grade 3	Withhold treatment until recovery to baseline or ≤ Grade 1.<br>If resolved within 2 weeks from the day of onset reduce to the next lower dose level. For participants at the lower DL: reduce M9140 by 25%.<br>If not resolved within 2 weeks from the day of onset or there is a recurrence, consider permanent discontinuation according to the Investigator's medical judgment and Sponsor`s Medical Monitor consultation.<br>Consider symptomatic treatment in accordance with institutional guidelines.
	Grade 4	**First occurrence:** Withhold treatment until recovery to baseline or ≤ Grade 1 and implement dose reduction as for Grade 3 at first occurrence. Permanent discontinuation of treatment should be considered.<br>**In case of (first) recurrence:** permanently discontinue treatment
**Infusion-related reactions (IRR)**	Grade 1	• If IRR is observed during administration, the infusion speed should be reduced by 50% and participants should be closely monitored.<br>• If no other reactions appear, the subsequent infusion could be resumed at the initial planned speed.
	Grade 2	• Administration of M9140 should be interrupted briefly.<br>• Consider symptomatic treatment (e.g., antihistamines, NSAIDs, narcotics, iv fluids)<br>• If the event resolves or improves to Grade 1, infusion can be restarted at a 50% reduced infusion speed.<br>• Subsequent administrations should be conducted at the reduced speed.<br>• Additional premedication* can be considered for subsequent infusions according to physician's clinical judgment.
	≥ Grade 3 (including anaphylaxis/anaphylactic reactions)	• Administration of M9140 should be discontinued immediately and permanently.<br>• Urgent intervention indicated. Antihistamines, steroids, epinephrine, bronchodilators, vasopressors, intravenous fluid therapy, oxygen inhalation etc., should be administered as clinically indicated.

ANC: absolute neutrophil count, G-CSF: granulocyte colony-stimulating factor, GI: gastrointestinal, Hgb: hemoglobin.

*Premedication for prophylaxis of IRRs can be considered according to physician's judgment prior to each subsequent dose of M9140 for participants who have experienced Grade 2 IRR.

The following agents are considered; recommended doses are provided:

- Glucocorticoid therapy: equivalent to 80 mg-100 mg iv methylprednisolone, approximately 0.5 to 2 hours prior to the start of M9140 administration (glucocorticoid premedication may be omitted in participants with insulin-dependent diabetes).
- Antihistamine (H1 antagonist): equivalent to 25 mg-50 mg iv diphenhydramine, approximately 0.5 hours prior to the start of M9140 administration.

The following agents may be added to the premedication regimen at any time during the study at the Investigator's discretion; recommended doses are provided:

- Antihistamine (H2 antagonist): equivalent to 50 mg iv ranitidine or 20 mg iv famotidine or equivalent, approximately 0.5 hours prior to the start of M9140 administration.
- Acetaminophen: 1,000 mg iv (where available, or po), or equivalent, approximately 0.5 hours prior to the start of M9140 administration.

Note: Doses may be adjusted based on institutional practices.

Table 20 Dose Modifications for Capecitabine According to Toxicity Grades

Toxicity Grades*	Dose Changes Within a Treatment Cycle	Dose Adjustment for the Next Cycle/Dose (% of Starting Dose)
**Grade 1**	Maintain dose level	Maintain dose level
**Grade 2**		
1st appearance	Interrupt until resolved to Grade 0-1	100%
2nd appearance		75%
3rd appearance		50%
4th appearance	Discontinue permanently	Not applicable
**Grade 3**		
1st appearance	Interrupt until resolved to Grade 0-1	75%
2nd appearance		50%
3rd appearance	Discontinue permanently	Not applicable
**Grade 4**		
1st appearance	Discontinue permanently<br>or<br>If physician deems it to be in the patient's best interest to continue, interrupt until resolved to Grade 0-1	50%
2nd appearance	Discontinue permanently	Not applicable

Source: capecitabine local prescribing information.

* According to the National Cancer Institute of Canada Clinical Trial Group (NCIC CTG) Common Toxicity Criteria (version 1) or the Common Terminology Criteria for Adverse Events (CTCAE) of the Cancer Therapy Evaluation Program, US National Cancer Institute, version 4.0.

For those toxicities considered by the treating physician to be unlikely to become serious or life-threatening, e.g. alopecia, altered taste, nail changes, capecitabine treatment can be continued at the same dose without reduction or interruption.

Table 21 Temporary/Permanent Discontinuation of Bevacizumab for Bevacizumab-related Toxicities (Part 2C and 2D)

Specifics	Toxicity (NCI-CTCAE v5.0 Grade)	Dose Interruption and Modification
Gastrointestinal perforations and fistula	• Gastrointestinal perforations, Grade ≥ 1<br>• Tracheoesophageal fistula, Grade ≥ 1<br>• Fistula, Grade 4<br>• Fistula perforation involving any internal organ, Grade ≥ 1	Discontinue bevacizumab
Wound healing complications	• Wound healing complications requiring medical intervention<br>• Necrotizing fasciitis	Discontinue bevacizumab
Hemorrhage	Grade ≥ 3	Discontinue bevacizumab
	Recent history of hemoptysis of ½ teaspoon (2.5 ml) or more	Withhold bevacizumab
Thromboembolic events	Arterial thromboembolism, Grade ≥ 3	Discontinue bevacizumab
	Venous thromboembolism, Grade ≥ 4	Discontinue bevacizumab
Hypertension	• Hypertensive crisis<br>• Hypertensive encephalopathy	Discontinue bevacizumab
	Hypertension, Grade ≥ 3	Withhold bevacizumab, if not controlled with medical management. Resume once controlled
Posterior reversible encephalopathy syndrome (PRES)	Grade ≥1	Discontinue bevacizumab
Renal injury and proteinuria	Nephrotic syndrome	Discontinue bevacizumab
	Proteinuria greater than or equal to 2 g per 24 h in the absence of Nephrotic syndrome	Withhold bevacizumab until proteinuria less than 2 g per 24 h
Infusion-related reaction	Mild, clinically insignificant	Decrease infusion rate
	Mild or Moderate, clinically significant	Interrupt infusion, resume at a decreased rate of infusion after symptoms resolve
	Severe	Discontinue bevacizumab
Congestive heart failure	Any Grade	Discontinue bevacizumab
Anaphylaxis	Grade ≥ 3	Discontinue bevacizumab

Source: Bevacizumab local prescribing information.

Table 22 Dose Modification and Temporary/Permanent Discontinuation of 5-FU for related Toxicities (Part 2D)

Specifics	Category	Dose Interruption and Modification
WBC count	< 3,000/mm^3	Withhold until resolution, resume at 100% dose
Neutrophil count	< 1,000/mm^3	Withhold until resolution, resume at 100% dose
Platelet count	< 100,000/mm^3	Withhold until resolution, resume at 100% dose
Diarrhea	Grade 1	Withhold until resolution, resume at 100% dose
	Grade 2	Withhold until resolved; resume at 100% dose

	Grade 3	First occurrence:<br>Withhold until resolution; resume at 100% dose<br>Subsequent occurrence:<br>Withhold until resolved to Grade ≤1; resume at 75% dose
	Grade 4	First occurrence:<br>Withhold until resolution; resume at 100% dose<br>Subsequent occurrence:<br>Withhold until resolved to Grade ≤1; resume at 50% dose
Mucositis	Grade ≥ 1	Withhold until resolution, resume at 100% dose
Stomatitis	Grade 1	Withhold until resolution, resume at 100% dose
	Grade 2	Withhold until resolved; resume at 100% dose
	Grade 3	First occurrence:<br>Withhold until resolution; resume at 100% dose<br>Subsequent occurrence:<br>Withhold until resolved to Grade ≤1; resume at 75% dose
	Grade 4	First occurrence:<br>Withhold until resolution; resume at 100% dose<br>Subsequent occurrence:<br>Withhold until resolved to Grade ≤1; resume at 50% dose
Hand/foot syndrome	Grade 1	Withhold until resolution, resume at 100% dose
	Grade 2	Withhold until resolution, resume at 100% dose
	Grade ≥ 3	Discontinue permanently
Myocardial Ischemia	Grade ≥ 1	Discontinue permanently
Any other non-hematological toxicity	Grade ≥ 2	Withhold until resolution, resume at 100% dose

Source: 5-FU local prescribing information.
Toxicity grade according to NCI-CTCAE v5.0

In cases of development of angina, myocardial infarction/ischemia, arrhythmia, or heart failure in participants with no history of coronary artery disease or myocardial dysfunction, 5-FU or capecitabine should be discontinued permanently.

Although ILD/pneumonitis has not been observed in preclinical studies with M9140 or in the dose escalation Part 1 of the study and is considered unlikely for M9140, as a precaution this protocol provides the following guidance for management of ILD/pneumonitis, if it may occur, since this AE has been described for other ADCs (see Appendix 11 for Japan-specific risk management measures). If a participant develops an acute onset of new or worsening pulmonary or other related signs/symptoms such as dyspnea, cough, or fever, rule out ILD/pneumonitis. If the AE is suspected to be ILD/pneumonitis, treatment with M9140 should be interrupted pending further evaluations. Evaluations should include as soon as possible high-resolution CT scan, pulmonologist consultation (infectious diseases consultation as clinically indicated), blood culture and CBC and other blood tests could be considered, consider bronchoscopy and bronchoalveolar lavage if clinically indicated and feasible, pulmonary function test and pulse oximetry (SpO_2), arterial blood gases if clinically indicated as well as one blood sample collection for PK [redacted] analysis, if feasible. Other tests could be considered as needed.

If the AE is confirmed to have another etiology than treatment-related ILD/pneumonitis, follow the appropriate management guidance. As soon as ILD/pneumonitis is suspected, pause M9140 and start promptly corticosteroid treatment. If ILD/pneumonitis is Grade 1 monitor and follow-up clinically, by pulse oximetry and imaging as indicated and consider starting systemic steroids (e.g., at least 0.5 mg/kg/day prednisone or equivalent) until improvement, followed by gradual taper over at least 4 weeks. M9140 can be restarted only if the event is fully resolved to Grade 0. If resolved in ≤ 28 days from day of onset, maintain dose. If resolved in >28 days from day of onset, reduce M9140 to the next lower dose level for Part 1 or 25% dose reduction for Part 2.

If ILD/pneumonitis is Grade ≥ 2, permanently discontinue study medication and promptly start systemic steroids with at least 1 mg/kg/day. If Grade 3 or 4, hospitalize participant and promptly initiate empiric high-dose methylprednisolone iv treatment (e.g., 500-1,000 mg/day for 3 days followed by at least 1.0 mg/kg/day prednisone [or equivalent]) for at least 14 days or until complete resolution of clinical symptoms and chest CT findings with subsequent gradual taper.

6.5.5.1 Switching Between M9140 Dose Levels: Part 2A Dose Optimization

If the lower dose is discontinued early, participants will have the option to switch to the higher dose group if they:

- Did not experience an IMP related SAE, or an IMP related Grade ≥ 3 AE.
- The treating physician considers that M9140 treatment is beneficial for the participant.

If the higher dose is discontinued early, participants treated at this dose will have the option to switch to the lower dose group provided that the treating physician considers that M9140 treatment is beneficial for the participant.

In cases when the participant has achieved an OR and did not experience toxicity requiring a dose reduction and is willing to continue at the higher dose level, the participant will have the option to stay on their initial dose.

6.6 Continued Access to Study Intervention After the End of the Study

For participants who are still benefiting from study intervention at study termination the Sponsor will ensure access to study intervention via a rollover study, expanded access, marketed product, or another mechanism of access as appropriate.

6.7 Treatment of Overdose

For this study, any dose of any study intervention greater than 10% than the calculated dose for that particular administration within a 24-hour time period will be considered an overdose.

The Sponsor does not recommend specific treatment for an overdose of M9140. For treating an overdose, the Investigator will use his/her clinical judgment to manage any overdose, considering the symptoms and any site procedures or standards.

For any overdose of bevacizumab, capecitabine, 5-FU, and folinic acid, refer to the local prescribing information.

Even if not associated with an AE or a SAE, any overdose of any study intervention is recorded in the CRF and reported to Global Patient Safety in an expedited manner. Overdoses are reported on an SAE form, following the procedure in Appendix 4, the section on Reporting SAEs.

6.8 Concomitant Therapy

Record in the eCRF all concomitant therapies (e.g., medicines or nondrug interventions) used from the time the participant signs the informed consent until Safety Follow-up Visit, including any changes. For prescription and over-the-counter medicines, vaccines, vitamins, and herbal supplements, record the name, reason for use, dates administered, and dosing information.

Contact the Medical Monitor for any questions on concomitant or prior therapy.

6.8.1 Rescue Medicine

Not applicable.

6.8.2 Permitted Medicines

The only permitted medicines are the following:

- Prophylactic treatment:
  a. Prophylactic antiemetic agents and antihistamines at the Investigator's discretion and in accordance with institutional guidelines.
  b. See Appendix 11 for Japan-specific information on permitted hematopoietic growth factors.
  c. In the Part 1B regimen use of pegfilgrastim as primary prophylaxis for neutropenia is mandatory on D8 (±3 days), for the first 2 cycles and according to Investigator's discretion afterwards.
- In Part 1A, use of G-CSF is permitted during the DLT observation period for participants who have developed neutropenia, if clinically indicated according to physician's discretion, but if there is no DLT as reason for this, its use will constitute an ICE that in the Bayesian modeling will be treated like a DLT.
- For participants in Part 1A who have experienced Grade ≥ 3 neutropenia and/or febrile neutropenia secondary prophylactic administration of G-CSF is permitted for subsequent cycles according to physician's discretion (see Section 6.5.5).
- For participants in Parts 2A, 2B, 2C, and 2D, upfront, primary prophylactic administration of G-CSF is not permitted. For participants who have experienced Grade ≥ 3 neutropenia and/or febrile neutropenia, secondary prophylactic administration of G-CSF (pegylated or not) is permitted for all subsequent cycles according to physician's discretion (see Section 6.5.5).

- For Parts 2B, 2C, and 2D, use of G-CSF is permitted during the DLT observation period for participants who have developed neutropenia, if clinically indicated according to physician's discretion, but if there is no DLT as reason for this, its use will constitute an ICE that in the Bayesian modeling will be treated like a DLT.
- Outside the DLT observation period therapeutic use of G-CSF is permitted for all participants and both regimens, based on investigators judgment.
- Antidiarrheal therapy (e.g., loperamide) should be considered and administered in accordance with the prescribing information or institutional guidelines.
- Inhaled steroids, topical steroids, or intra articular steroid injections are permitted in the study. Brief, limited use of systemic corticosteroids (< 14 days) is also permitted where such use is considered standard of care (e.g. for COPD exacerbation).
- Vaccines: Investigators should assess prior to or at the beginning of the screening period, whether a participant should be vaccinated before receiving the first dose of IMP against a specific infectious disease, that may be a significant risk to the participant during the study. This assessment should include also for example the risk of COVID-19 infection or monkeypox infection. In general, investigators may take into consideration that the vaccination effect may be reduced in patients with cancer and may not lead to protection depending on the extent of the immunocompromised state of the participant including the impact of prior therapies.

Any medicines that are considered necessary to protect the participant's welfare in emergencies may be given at the Investigator's discretion, regardless of if it results in a protocol deviation.

6.8.3 Prohibited Medicines

If not otherwise specified, prohibited medications should not be used until the Safety Follow-up Visit.

- During the treatment period and until EOT visit, participants may not receive any other anticancer therapy (e.g chemotherapy, biological therapies, hormonal therapies, alternative traditional medicines [e.g., herbal medicines, traditional Chinese medicines]) or investigational therapeutic agents or surgery.
- In Parts 1A, 2B, 2C, and 2D, prophylactic use of G-CSF is not permitted during the DLT observation period.
- Steroid therapy for anti-neoplastic intent taken < 7 days prior to the first dose of study intervention.
- Concomitant or chronic use of corticosteroids in a dose ≥ 10 mg per day of prednisone or equivalent when administered for ≥ 2 weeks. If the Investigator deems that corticosteroid use is needed in a higher dose or longer duration (e.g. for toxicity management, AE or intercurrent disease), the Medical Monitor must be consulted.
- Live vaccines are prohibited during the 28 days prior to C1D1 and during the whole study, as they may cause severe vaccine-induced infections in immunocompromised individuals.

Non-live vaccines are prohibited during the 14 days prior to C1D1, in the DLT period (dose escalation and dose expansion parts) and during the ± 7 days of each subsequent study intervention administration.

- Growth factors (including EPO, darbepoetin, G-CSF, GM-CSF, and platelet stimulators [e.g., eltrombopag, romiplostim, or IL-11]) or transfusions within 2 weeks prior to the first day of study intervention.
- Radiotherapy (except palliative stereotactic radiotherapy to known metastatic sites as long as it does not interrupt any study intervention for longer than the maximum time specified in dose modification and does not affect assessment of the response).
- All strong CYP3A4 inhibitors/inducers taken < 7 days prior to the first dose of study intervention. For a listing refer to Appendix 7 (https://www.fda.gov/drugs/drug-interactions-labeling/drug-development-and-drug-interactions-table-substrates-inhibitors-and-inducers).
- Moderate CYP3A4 inhibitors should be avoided unless no alternative treatment exists. If coadministered, to be used cautiously with close monitoring of AEs.
- Strong CYP1A2 inhibitors should be avoided unless no alternative treatment exists. If coadministered, to be used cautiously with close monitoring of AEs.
- Participants will be instructed to refrain from consumption of St. John's wort, as well as any other herbal/natural products which are potential CYP3A inhibitors/inducers (e.g., essiac tea) starting at least 7 days before the first administration of any study intervention and during the study intervention administration.
- **Part 2C and 2D**: Treatment with oral or parenteral anticoagulants or thrombolytic agents for therapeutic purpose.
- **Part 2C2 and 2D**: Brivudine, sorivudine, or their analogues must not be administered concomitantly to participants treated with capecitabine or 5-FU. Concomitant use of allopurinol with capecitabine should be avoided.
- For details on drug-drug interactions and prohibited medications with capecitabine, 5-FU, bevacizumab or folinic acid, refer to the local product labels.

6.8.4 Other Interventions

Major surgery within 4 weeks prior start of study intervention is not permitted.

Part 2C and 2D: Treatment with bevacizumab should be interrupted for elective surgery.

Concurrent participation in another clinical study is not allowed until the Safety Follow-up has been completed.

Concomitant use of dietary supplements, medications not prescribed by the Investigator and alternative/complementary treatment (e.g., traditional Chinese medicines) are not recommended.

7 Discontinuation of Study Intervention and Participant Discontinuation/Withdrawal

7.1 Discontinuation of Study Intervention

If study intervention is permanently discontinued, the participant will remain in the study to be evaluated for the End of Treatment and Safety Follow-up Visit as well as Survival Follow-up visits (only for Part 2A). The SoA indicates data to be collected at the time of discontinuation of study intervention and follow-up and for any further evaluations that need to be completed.

Participants must be withdrawn from any study intervention if any of the following occurs:

- A participant's study intervention should be discontinued if the Investigator believes that for safety reasons (e.g., AE) it is in the participant's best interest.
- The Investigator is required to discontinue any study intervention for abnormal liver function when a participant meets 1 of the conditions outlined below or if the Investigator believes that it is in best interest of the participant.

  All events of ALT/AST ≥ 3 × ULN and bilirubin ≥ 2 × ULN (> 35% direct bilirubin) or ALT > 3 × ULN and INR > 1.5, if INR measured, may indicate potential severe liver injury (possible Hy's Law), and will be reported as an SAE.
- Cardiac changes (e.g., QTc):

  If a clinically significant finding is identified (including changes from baseline in QT interval corrected using Fridericia's formula [QTcF]) by > 60 ms or above 500 ms after start of study intervention, the Investigator or qualified designee upon consultation with the Medical Monitor will determine if the participant can continue in the study and if any change in participant management is needed. While increases in QT/QTc to > 500 ms or of > 60 ms over baseline are commonly used as thresholds for potential discontinuation, the exact criteria chosen for this study will depend on the risk-benefit level considered appropriate for the participant in question. This review of the ECG at the time of collection will be documented. Any new clinically relevant finding is reported as an AE.
- The participant becomes pregnant.
- The participant requests to been withdrawn from administration of any study intervention.
- The participant's dose is held for more than 28 days, or if > 1 consecutive planned administration of M9140 are missed for reasons other than toxicity unless, upon consultation with the Sponsor and the review of safety and efficacy, continuation is agreed upon.
- Prohibited concomitant medication: If the administration of a prohibited concomitant medication becomes necessary during the study, study intervention will be discontinued. The Medical Monitor will be contacted first to discuss whether study intervention will be discontinued.
- Occurrence of disease progression according to RECIST v1.1

- Noncompliance with the study intervention or failure to attend scheduled assessments that are deemed necessary for the participant's safety or study integrity for more than 28 days.
- Any DLT during the DLT observation period: Participants experiencing DLTs as described in Section 6.5.4 will not receive further study intervention based on Investigator's decision and Sponsor's Medical Monitor agreement.

7.2 Participant Discontinuation/Withdrawal from the Study

- A participant may discontinue from the study at any time, at his or her own request or at the discretion of the Investigator for safety, behavioral, compliance, or administrative reasons.
- At the time of study discontinuation, if possible, a discontinuation visit will be conducted, as listed in the SoA. The SoA specifies the data to collect at study discontinuation and follow-up, and any additional evaluations that need to be completed.
- If the participant revokes consent for the study, any data collected up to that point may still be used, but no future data can be generated, and any biological samples collected will be destroyed.
- If a participant requests the destruction of any biological samples still remaining, the Investigator will document this in the site study records and inform the Sponsor. The samples will be destroyed.

7.3 Lost to Follow-Up

A participant will be considered lost to follow-up if he or she repeatedly fails to return for scheduled visits and is unable to be contacted by the study site.

The following actions will be taken if a participant fails to return to the clinic for a required study visit:

- The site will attempt to contact the participant and reschedule the missed visit as soon as possible, counsel the participant on the importance of maintaining the assigned visit schedule and ascertain if the participant wants to or should continue in the study.
- Before a participant is deemed "lost to follow-up", the Investigator or designee will make every effort to regain contact with the participant: 1) where possible, make 3 telephone calls; 2) if necessary, send a certified letter (or an equivalent local method) to the participant's last known mailing address, and 3) if a participant has given the appropriate consent, contact the participant's general practitioner or caretaker (where allowed by local regulations) for information. These contact attempts will be documented in the participant's medical record.
- If the participant continues to be unreachable, he/she will be deemed as "lost to follow-up".

8 Study Assessments and Procedures

- Study assessments and procedures and their timing are summarized in the SoA.
- **No** protocol waivers or exemptions are allowed.

- Immediate safety concerns are discussed with the Sponsor immediately upon occurrence or awareness to determine if the participant should continue or discontinue study intervention.
- Adherence to the study design requirements, including those specified in the SoA, is essential and required for study conduct.
- All screening evaluations will be completed and reviewed to confirm that potential participants meet all eligibility criteria. The eligibility will be confirmed at Cycle 1 Day 1. The Investigator will maintain a screening log to record details of all participants screened, to confirm eligibility, and if applicable, record reasons for screening failure.
- Demographics will include birth year, age at informed consent, sex, race, and ethnicity (collected only if allowed by local laws/ regulations).
- Past and Current Medical History will include previous illness and surgeries (all during the past year and only major ones prior to that), concomitant illness, allergies, and disease history.
- Prior to performing any study assessments that are not part of the participant's routine medical care, the Investigator will obtain written informed consent as specified in Appendix 2.
- Procedures conducted as part of the participant's routine medical care (e.g., blood count) and obtained before signing of the ICF may be used for screening or baseline purposes provided the procedures met the protocol specified criteria and were performed within the time frame defined in the SoA.
- Where allowed by local law/regulations, samples collected during this clinical study may be transferred to a biobank and used for future research outside the clinical protocol when additional consent for this purpose is given. Transfer to the biobank will be documented and any testing of coded biobank samples will **not** be reported in the CSR.
- The long-term storage of samples after study completion for future research may be performed with all sample types collected in the study (e.g., PK, CCI [redacted]) if the participant consents to optional future medical research.
- Details on processes for collection and handling of these samples are in the Laboratory Manual. The Sponsor will store the samples in a secure storage space with adequate measures to protect confidentiality. Retention time and possible analyses of samples after the end of study are specified in the respective ICF.

8.1 Efficacy Assessments and Procedures

Efficacy assessments (Contrast-enhanced CT/MRI) and procedures and their timing are summarized in the SoAs (Section 1.3).

In Part 1 in addition to a baseline assessment prior to the start of study intervention, CT scans will be done for M9140 tumor assessments every 6 weeks (±7 days) following the Cycle 1 Day 1 Visit. After the 3rd tumor scan (Screening, Evaluation 1, and Evaluation 2) the following tumor assessments will be done every 9 weeks (±7 days) until EOT visit.

For Part 2 CT scans will be done every 6 weeks (±7 days) following the Cycle 1 Day 1 Visit. After the 4th tumor scan (Screening, Evaluation 1, Evaluation 2, and Evaluation 3) the following

tumor assessments will be done every 12 weeks (±7 days) until disease progression per RECIST v1.1 or start of new anticancer therapy (Part 2A), whichever comes first, or until EOT visit (Parts 2B, 2C, and 2D).

In case a participant discontinues treatment before reaching the next scheduled tumor assessment for example due to an AE or due to clinical signs of progression, a CT or MRI scan should be performed at the time of treatment discontinuation, if the participant had received the last dose at least 2 weeks ago, if deemed appropriate by the Investigator in order to assess tumor response. This CT or MRI is to be reported in the eCRF.

Regular on-study tumor assessments (besides by regular CT scans) can also be done by MRI or PET CT scan (if MRI is used, CT of chest is mandatory), using the same method at all subsequent assessment time points. Imaging of the chest/abdomen/pelvis (plus other anatomical regions as clinically indicated) and other established assessments of tumor burden are to be performed in case CT/MRI imaging is insufficient for the individual participant. A brain CT/MRI scan will be performed, if clinically indicated by development of new specific symptoms. Skin metastasis can be used as target lesions according to RECIST v1.1 using measurements by caliper, if they fulfill RECIST v1.1 for target lesions (see Appendix 9). All the scans performed at Baseline need to be repeated at subsequent visits for tumor assessment. In general, lesions detected at Baseline need to be followed using the same imaging methodology and preferably the same imaging equipment at subsequent tumor evaluation visits.

At the Sponsor's discretion an independent review committee may be implemented to independently assess confirmed ORR, DOR, and PFS. Imaging data should be stored for possible later central assessment.

8.2 Safety Assessments and Procedures

The safety profile of any study intervention will be assessed through the recording, reporting, and analysis of baseline medical conditions, AEs, physical examination findings, vital signs, electrocardiograms, laboratory tests, and patient-reported symptoms.

Comprehensive assessment of any potential toxicity experienced by each participant will be conducted starting when the participants give informed consent and throughout the study until the 30-day Safety Follow-up Visit. The Investigator will report any AEs, whether observed by the Investigator or reported by the participant; the reporting period is specified in Section 8.3.

8.2.1 Physical Examinations

- A complete physical examination will include, at a minimum, assessments of the cardiovascular, respiratory, gastrointestinal, skin, and neurological systems.
- Investigators will pay special attention to clinical signs related to previous serious illnesses.

8.2.2 Vital Signs

- Blood pressure and participant's position; pulse; respiratory rate; temperature and location of measurement as indicated in the Schedule of Activities (Section 1.3). Weight and BMI (at

Day 1 of each cycle), BSA (Day 1 of each cycle, Parts 2C2 and 2D only), and height (at Screening only) will be measured and recorded.

- Blood pressure and pulse measurements will be preceded by at least 5 minutes of rest for the participant in a quiet setting without distractions (e.g., television, cell phones), in the sitting or semi-recumbent position and measured with an automated device. Manual techniques will be used only if an automated device is not available.
- Vital signs (to be taken before blood collection for laboratory tests) will consist of 1 pulse and 3 blood pressure measurements (3 consecutive blood pressure readings will be recorded at intervals of at least 1 minute). The average of the 3 blood pressure readings will be recorded on the CRF.

8.2.3 Electrocardiograms

2 sets of ECGs are requested for this trial: Safety ECG and triplicate ECG for QT/QTc evaluation. Both ECGs can be recorded using the same ECG device, a provided Holter recorder (long-term/continue ECG). For the Safety ECGs local equipment is acceptable as well.

Holter recorder is not intended to be utilized for long-term ECG evaluation (e.g., 24 hours) but only due to practical reasons in order to facilitate several ECG measurement timepoints on 1 day. Holter ECG will be set up and collection started before any blood sample collection on this day and can be stopped and device be removed after all required assessment timepoints have been recorded on this day.

8.2.3.1 Safety ECGs

Standard single 12-Lead ECGs will be obtained for safety monitoring as outlined in the SoA using an ECG machine (Holter recorder) that automatically measures heart rate, PR, RR, QRS, QT, and QTcF. The safety ECG should be performed after at least 10 minutes rest in a recumbent or semi-recumbent position and prior to blood sampling. Safety ECGs will be read locally and should be assessed on the day of collection and on dosing days before study drug administration by a physician or a qualified delegate according to the site’s policy.

At times where both, Safety ECG, and triplicate ECG for QT/QTc evaluation are required, a print-out of the triplicate ECG for QT/QTc evaluation can be used for the Safety ECG assessment.

Investigators should assess in the Safety ECGs, if a clinically significant finding is identified, including changes from Baseline in QT interval corrected using Fridericia’s formula (QTcF) by > 60 ms or above 500 ms after start of treatment. In case of a clinically significant finding, Investigators should interrupt study treatment until further clinical evaluation. ECGs should be repeated if QTc is outside the range until resolution. To assess the safety and tolerability of the study intervention, an ECG can be repeated at the Investigator’s discretion at unscheduled visits.

8.2.3.2 QT/QTc Evaluation

Triplicate 12-lead ECG for QT/QTc Evaluation will be obtained as outlined in Table 2 and Table 4 using a provided ECG machine (Holter recorder) that automatically measures heart rate, PR, RR, QRS, QT, and QTcF. The ECG should be performed after at least 10 minutes rest in a recumbent or semi-recumbent position and prior to blood sampling. Triplicate digital ECGs will be extracted from continuous Holter ECGs as outlined in Table 2 and Table 4. The data will be archived in digital format using a central ECG vendor and will be analyzed by a specialized central laboratory.

At each timepoint at which triplicate ECG are required, 3 individual ECG tracings will be obtained as closely as possible in succession, but no more than 2 minutes apart. The full set of triplicates will be completed in less than 4 minutes.

For Part 2 of the study, triplicate 12-lead ECG for QT/QTc evaluation is requested only in Part 2A for approximately 24 participants (n≈12 for each Arm 2A1 and Arm 2A2).

8.2.4 Clinical Safety Laboratory Assessments

- Blood and urine samples will be collected for the clinical laboratory tests listed in Appendix 6 at the time points listed in the SoA. All samples will be clearly identified.
- Additional tests may be performed at any time during the study, as determined necessary by the Investigator or required by local regulations.
- The tests will be performed by the local laboratory.
- Any changes to the ranges during the study will be forwarded to the Sponsor or designated organization.
- The Investigator will review each laboratory report, document this review, and record any clinically significant changes occurring during the study as an AE, unless it does **not** meet the AE definition, as specified in Appendix 4. The laboratory reports will be filed with the source documents.
- Pregnancy testing will be conducted during Screening (serum pregnancy test) and within the time frame for female participant contraception as stated in Section 5.1 (Inclusion Criteria). Additionally, pregnancy testing (highly sensitive urine or serum test as required by local regulations) will be performed at the timepoints indicated in the SoAs (see Table 1, Table 3, Table 5, Table 7, and Table 9 for details).

8.2.5 Patient-Reported Symptomatic Adverse Events

The NCI has created a PRO version of the CTCAE to assess the patient perspective on symptomatic AEs (PRO-CTCAE). The PRO-CTCAE is a patient-reported measure that ascertains in the frequency, severity, interference, and presence/absence of symptomatic toxicities that can be meaningfully reported from the patient perspective. Research indicates the psychometric properties including validity, reliability, responsiveness, and test-retest reliability, are acceptable (Basch 2014, Dueck 2015).

The PRO-CTCAE assessments will be completed independently by participants and where possible, prior to administration of study intervention. The CRO and the Sponsor will review available data rate on a regular basis. PROs will not be reviewed or used to inform care decisions nor for safety purposes.

8.2.5.1 PRO-CTCAE Item Library

The PRO-CTCAE item library consists of 124 items representing 78 symptomatic toxicities and has been validated in more than 30 languages. The measure is intended to serve as a complement to the physician-reported CTCAE to better reflect the patient's experience of low-grade symptoms and how they impact patients' lives. After careful review of supporting literature and safety data from Part 1 of the study, the AEs of fatigue, GI distress (nausea, diarrhea, and vomiting) and mouth sores were considered the most salient symptomatic AEs for M9140 in this patient population (Table 23; Appendix 12). See Part 2A SoA for details on the frequency of PRO-CTCAE assessment (Table 3).

Table 23 Symptomatic Toxicity Items

Symptomatic Toxicity	Symptomatic Toxicity Items
Sleep/ Wake	CCI [redacted]
Gastrointestinal	Nausea<br>Vomiting<br>Diarrhea
Oral	Mouth Sores

8.2.6 Suicidal Ideation and Behavior Risk Monitoring

Not applicable.

8.3 Adverse Events, Serious Adverse Events, and Other Safety Reporting

- The definitions of an AE and a SAE are in Appendix 4 and the definition of new facts is in Appendix 11.
- The Investigator and any qualified designees (e.g., Sub-Investigators) are responsible for detecting, documenting, and recording events that meet the definition of an AE or SAE. The Investigator remains responsible for following up all AEs or AEs that are serious, considered related to any study intervention or study procedures, or that caused the participant to discontinue the study intervention or study, as specified in Section 8.3.2.
- Requests for follow-up will usually be made via the Sponsor or CRO-designated study team member, although in exceptional circumstances the Global Patient Safety department may contact the Investigator directly to obtain further information or to discuss the event.
- The method of recording, evaluating, and assessing causality of AEs and SAEs and the procedures for completing and transmitting SAE reports are in Appendix 4.

- All AEs and SAEs will be collected from the signing of the ICF until the Safety Follow-up Visit at the time points specified in the SoA (Section 1.3). Beyond this reporting period, any new unsolicited SAEs that the Investigator spontaneously reports to the Sponsor will be collected and processed.
- All SAEs will be recorded and reported to the Sponsor or designee immediately and under no circumstance will this exceed 24 hours, as indicated in Appendix 4. The Investigator will submit any updated SAE data to the Sponsor within 24 hours of it being available using the same procedure that was used for the initial report.
- Investigators are not obligated to actively solicit information on AEs or SAEs after the end of study participation. However, if the Investigator learns of any SAE, including a death, at any time after a participant has been discharged from the study, and he/she considers the event to be reasonably related to any study intervention or study participation, the Investigator will promptly notify the Sponsor.

8.3.1 Method of Detecting Adverse Events and Serious Adverse Events

At each study visit, the participant will be queried on changes in his or her condition.

Care will be taken not to introduce bias when detecting AEs and/or SAEs. Open-ended and non-leading verbal questioning of the participant is the preferred method to inquire about AE occurrences.

The method of recording, evaluating, and assessing causality of AEs and SAEs and the procedures for completing and transmitting SAE reports are in Appendix 4.

8.3.2 Follow-up of Adverse Events and Serious Adverse Events

After the initial AE/SAE report, the Investigator is required to proactively follow each participant at subsequent visits/contacts. All SAEs will be followed until resolution, stabilization, the event is otherwise explained, or the participant is lost to follow-up (as defined in Section 7.3). Reasonable attempts to obtain this information will be made and documented. It is also the Investigator's responsibility to ensure that any necessary additional therapeutic measures and follow-up procedures are performed. Further information on follow-up procedures is in Appendix 4.

8.3.3 Regulatory Reporting Requirements for Serious Adverse Events

Prompt notification by the Investigator to the Sponsor of an SAE (particularly life-threatening and deaths) is essential so that legal obligations and ethical responsibilities toward the safety of participants and the safety of a study intervention under clinical investigation are met.

The Sponsor has a legal responsibility to notify both the local regulatory authority and other regulatory agencies about the safety of a study intervention under clinical investigation. The

Sponsor will comply with country-specific regulatory requirements relating to safety reporting to the regulatory authority, IRB/IEC, and Investigators.

Individual Case Safety Reports will be prepared for SUSARs according to local regulatory requirements and Sponsor policy and forwarded to Investigators, as necessary.

An Investigator or sub-investigator who receives an Individual Case Safety Report describing a SUSAR or other specific safety information (e.g. Emerging Safety Issue Report, summary or listing of SAEs/SUSARs) from the Sponsor will review the safety reports and confirm completion of this review. This information will be filed it in the Investigator's Site File, and the IRB/IEC will be notified, if appropriate, according to applicable local laws/regulations and site SOPs.

For studies in EU/EEA and submitted under the EU Clinical Trial Regulation 536/2014, SUSARs will be reported centrally via the EudraVigilance database to Health Authorities by the Sponsor.

In this global clinical multicenter study, the Sponsor is in the best position to determine an unanticipated problem (as defined in US Regulations 21 CFR 312.66). The Sponsor will immediately notify all Investigators of findings that could adversely affect the safety of participants, impact the conduct of the study, or alter the IRB's approval/favorable opinion to continue the study. An unanticipated problem is a SAE that by its nature, incidence, severity, or outcome has not been identified in the current version of the risk analysis report, specified in Section 2.3.1.

8.3.4 Pregnancy

- Details of all pregnancies in female participants and, if indicated, female partners of male participants will be collected after the start of any study intervention and until 9 months after the last dose of any study intervention is administered for female participants and 6 months after the last dose of study intervention is administered for female partners of male participants.
- If a pregnancy is reported, the Investigator will record the pregnancy information on the appropriate form and submit it to the Sponsor within 24 hours of female participant or female partner of male participant (after obtaining the necessary signed informed consent from the female partner) pregnancy.
- While pregnancy itself is not considered to be an AE or SAE, any pregnancy complication or elective termination of a pregnancy will be reported as an AE or SAE. Adverse pregnancy outcomes (e.g., spontaneous abortion, fetal death, stillbirth, congenital anomalies, ectopic pregnancy) are considered and reported as SAEs. A spontaneous abortion (occurring at < 22 weeks gestational age) or stillbirth (occurring at > 22 weeks gestational age) is always considered to be an SAE and will be reported as such.
- The participant/pregnant female partner will be followed to determine the outcome of the pregnancy. The Investigator will collect follow-up information on the participant/pregnant female partner and the neonate, and the information will be forwarded to the Sponsor.

Generally, follow-up will not be required for longer than 6 to 8 weeks beyond the estimated delivery date for a healthy newborn. In case of a congenital anomaly or other illness of the newborn, follow-up will continue until the illness has resolved or there is a definite outcome of the event.

- Any post-study pregnancy related SAE considered reasonably related to any study intervention by the Investigator will be reported to the Sponsor as specified in Section 8.3.3. While the Investigator is not obligated to actively seek this information in former study participants/pregnant female partner, he or she may learn of an SAE through spontaneous reporting.
- Any female participant who becomes pregnant while participating in the study will discontinue all study intervention or be withdrawn from the study.

8.3.5 Cardiovascular and Death Events

Not applicable.

8.3.6 Disease-Related Events and/or Disease-Related Outcomes Not Qualifying as AEs or SAEs

The following disease-related events (DREs) are common in participants with locally advanced or metastatic CRC and can be serious/life-threatening:

- Progressive disease, including brain metastasis.

Because these events are typically associated with the disease under study, they will not be reported according to the standard process for expedited reporting of SAEs even though the event may meet the definition of an SAE. These events will be recorded within the next visit.

However, if any of the following conditions applies, then the event will be recorded and reported as an AE and SAE (instead of a DRE):

- The event is, in the Investigator's opinion, of greater intensity, frequency, or duration than expected for the individual participant.

  OR

- The Investigator considers that there is a reasonable possibility that the event was related to study intervention.

8.3.7 Adverse Events of Special Interest (Part 2)

For Part 2 of this study, AESI(s) include only the following:

- CCI [redacted]
- CCI [redacted]

The Investigator must report any AESI to the Sponsor within 24 hours of becoming aware of the event. Non-serious AESIs must be reported using the AESI Report Form and serious AESIs must be reported using the SAE form (Appendix 4).

8.4 Pharmacokinetics

The following PK parameters will be calculated for M9140 (conjugated antibody, unconjugated payload, and total antibody), when appropriate:

AUC_{τ}	The area under the concentration-time curve (AUC) over the dosing interval from T_1=0 h to T_2=τ h. Calculated using the mixed log-linear trapezoidal rule (linear up, log down). For single dose, AUC_{τ} is calculated as a partial area with the defined time range. In multiple dose profiles AUC_{τ} is calculated at steady state from one predose timepoint to the dosing interval time. In cases where the actual observation time is not equal to the scheduled observation time AUC_{τ} will be calculated based on the estimated concentration at τ hours, and not the concentration at the actual observation time.
AUC_{τ} /Dose	The Dose normalized AUC over the interval from T_1=0 h to T_2=τ h. Normalized using actual dose, using the formula AUC_{τ} /Dose.
$AUC_{0\text{-tlast}}$	The area under the concentration-time curve (AUC) from time zero (= dosing time) to the last sampling time (t_{last}) at which the concentration is at or above the lower limit of quantification. Calculated using the mixed log-linear trapezoidal rule (linear up, log down).
$AUC_{0\text{-tlast}}$/Dose	The Dose normalized AUC from time zero to the last sampling time (t_{last}) at which the concentration is at or above the lower limit of quantification. Normalized using the actual dose, using the formula $AUC_{0\text{-tlast}}$ /Dose.
$AUC_{0-\infty}$	The AUC from time zero (dosing time) extrapolated to infinity, based on the predicted value for the concentration at $t_{last,}$ as estimated using the linear regression from λ_z determination. $AUC_{0-\infty}$= $AUC_{0\text{-tlast}}$ +$C_{last\ pred}$/ λ_z.
$AUC_{0-\infty}$ /Dose	The dose normalized AUC from time zero extrapolated to infinity. Normalized using actual dose, using the formula $AUC_{0-\infty}$ /Dose.
$AUC_{extra\%}$	The AUC from time t_{last} extrapolated to infinity given as percentage of $AUC_{0-\infty}$. AUC_{extra} = (extrapolated area/$AUC_{0-\infty}$) *100.
C_{eoi}	The observed concentration at the end of the infusion period.
CL	The total body clearance of study intervention following intravenous administration. Apparent clearance will be calculated for total antibody and unconjugated payload. CL = Dose $_{i.v}$ /$AUC_{0-\infty}$.
C_{trough}	The concentration observed immediately before next dosing (corresponding to predose or trough concentration for multiple dosing).

$R_{acc(AUC\tau)}$	The accumulation ratio after repeated administration calculated as $R_{acc(AUC\tau)}$= (AUC_τ after multiple dose (at steady state))/(AUC_τ after single dose).
$R_{acc(Cmax)}$	The accumulation factor to assess the increase in maximum concentration until steady state is reached. $R_{acc(Cmax)}$ = (C_{max} after multiple dose (at steady state))/ (C_{max} after single dose).
$t_{1/2}$	Apparent terminal half-life. $t_{1/2} = \ln(2)/\lambda_z$.
t_{last}	The last sampling time at which the concentration is at or above the lower limit of quantification
t_{max}	The time to reach the maximum observed concentration collected during a dosing interval (unless otherwise defined, take the 1st occurrence in case of multiple/identical C_{max} values)
V_Z	The apparent volume of distribution during the terminal phase following intravenous administration. Apparent volume of distribution will be calculated for total antibody and unconjugated payload. V_Z = Dose/($AUC_{0-\infty}$*λ_z) following single dose. V_Z = Dose/(AUC_τ*λ_z) following multiple doses

Renal clearance (CL_r) for the payload in each participant will be calculated based on available urine and plasma PK data in Part 1 only. As permitted by the data, renal clearance will be estimated as the ratio of the cumulative drug amount excreted in urine over the entire collection period and the plasma AUC over the same period or CL_r= Ae_{0-t}/AUC_{0-t}.

The PK parameters will be summarized using descriptive statistics. Individual as well as mean concentration-time plots will be depicted.

[redacted].

Whole blood samples for measurement of [redacted] plasma concentrations of M9140 (conjugated antibody, total Ab and unconjugated exatecan), bevacizumab, capecitabine, and 5-FU will be collected. Collection times are specified in the SoA. The actual date and time (24-hour clock time) of each sample will be recorded to calculate actual time elapsed since the prior dose administration. The sampling timing may be altered during the study based on newly available data (e.g., to obtain data closer to the time of peak plasma concentrations) to ensure appropriate monitoring.

- Part 1 only: Urine samples for measurement of urine concentrations of unconjugated exatecan will be collected at every clinic visit up to Day 15 in Cycle 1. Urine volume and collection time will be recorded in the eCRF. Collection days are specified in the SoA. Upon arrival on Day 1, the participant will be asked for bladder emptying without collecting this urine; subsequently, the participant will be hydrated by drinking water or fruit juice and all urine after study drug administration until departure from the clinic will be collected. On all other collection days, urine will be collected upon arrival in the clinic and urine collection time (collection end time in eCRF), as well as the time of previous urination at home (collection start time in eCRF), recorded. In case of hospitalization (applicable to Japan sites, see

Appendix 11) all urine will be collected during the days specified in the SoA, and collection times recorded during the stay in the hospital.

- The quantification of M9140 (conjugated antibody, total Ab, and unconjugated exatecan) in plasma will be performed using validated methods. Concentrations will be used to evaluate the PK of M9140 (conjugated antibody, total Ab, and unconjugated exatecan).
- The quantification of unconjugated exatecan in urine will be performed using a validated method. Remaining samples may be analyzed for additional metabolites based on newly available data.
- Remaining plasma samples collected for analyses of M9140 (conjugated antibody, unconjugated payload, and total antibody) concentration may also be used to evaluate additional metabolites, immunogenicity and safety or efficacy aspects related to concerns arising during or after the study. Details on processes for collection and handling of these samples are in the Laboratory Manual. Retention time and possible analyses of samples after the end of study are specified in the respective ICF.
- The quantification of bevacizumab, capecitabine (parent drug and metabolite 5-FU), and 5-FU in plasma will be performed using validated methods. Concentrations will be used to evaluate their PK profiles as compared to literature data.

CCI [redacted]

CCI [redacted]

8.7 Immunogenicity Assessments

- Samples are collected only where allowed by local law/regulations.
- Whole blood samples will be collected for detection of antibodies against M9140 in plasma. Collection times are specified in the SoA.
- The detection of antibodies to M9140 will be performed using a validated method CCI [redacted].
- Where allowed by local law/regulations, remaining samples collected for analysis of anti-M9140 antibodies may also be used to evaluate M9140 concentration CCI [redacted] during or after the study.

- Details on processes for collection and handling of these samples are in the Laboratory Manual. Retention time and possible analyses of samples after the end of study are specified in the respective ICF.

9 Statistical Considerations

All analyses will be prepared by dose level and will be described in detail in the IAP.

There is no formal family-wise Type I error control for this study, as such all analyses are considered descriptive.

9.1 Statistical Hypotheses

This is an exploratory study. No formal statistical hypothesis will be tested.

9.2 Sample Size Determination

Dose Escalation – Part 1

The planned cohort size is 3 participants. Parallel screening and enrollment of participants will allow eligible participants to enroll, the cohort size may therefore increase to a maximum of 4 participants. Additionally, the SMC may recommend changing the cohort size.

The total sample size will depend on the number of cohorts to be evaluated. It is anticipated that approximately 21 to 30 participants (5 projected dose levels with 3 to 9 participants each) may be needed in Part 1A of the dose escalation (without pegfilgrastim prophylaxis). For Part 1B (dose escalation with pegfilgrastim prophylaxis) 9-15 participants (3 projected dose levels with 3 to 9 participants) are anticipated.

Dose Expansion – Part 2

Part 2 will include 4 Parts (2A, 2B, 2C, and 2D) with approximately 135 to 170 participants in total.

- Part 2A will randomize 60 participants to Arm A1 and Arm A2 (30 in each), stratified by BMI, to ensure a balanced distribution of participants with a dose cap across both arms.
- Approximately 20 to 30 participants will be enrolled in Part 2B to evaluate an additional dosing regimen ([redacted]). The total sample size will depend on the number of cohorts and dose levels to be evaluated. It is anticipated that approximately 20 to 30 participants (2 projected dose levels with 3 to 15 participants each) may be needed to inform RP2D selection. With n=15, if a DLT rate of 27% is observed, then the posterior probability that the true rate is below 35% is > 71%. The planned cohort size is 3 participants. The SMC may recommend changing the cohort size.
- Part 2C will include 35 to 50 participants (approximately 15 to 20 in Part 2C1 and approximately 20 to 30 in Part 2C2).
- Part 2D will include 20 to 30 participants.

If the M9140 dose is modified at the interim analysis in Part 2A (described below) or upon recommendation of the SMC, then only participants treated at the same dose will be considered for interim futility analyses. After the dose modification, participants will be enrolled at the new dose until additional 15 participants treated at the new dose are evaluable for response. Subsequently, a new interim analysis will be performed with the same decision criteria. Therefore, the number of participants might increase by approximately 15 participants to reach the required number of 30 participants treated at the same dose who are evaluable for response according to RECIST v1.1.

For Arm A1 and Arm A2, the sample size will give a posterior probability of 83% that the ORR is ≥ 15% if 6/30 responses are seen at the final analysis. Based on the treatment landscape as described in Section 4.2, 15% is regarded as a notable improvement in the targeted population.

Possible decisions at interim analysis are shown in Section 9.2.1, Table 24.

9.2.1 Part 2A: Interim Futility Analyses

Part 2A: An interim analysis for futility will be performed after approximately 15 participants in each Arm A1 and Arm A2 are evaluable for response (see Section 9.4.4) to consider stopping one or both of the two dose groups. Moreover, at selected time points (see Section 6.5.3) the SMC will evaluate the totality of safety data to determine the tolerability of each dose regimen.

Table 24 Decision Criteria at Interim Analysis for Part 2A

Arm	Interim Analysis (Numbers apply to each Arm A1 and Arm A2 separately)			Final Analysis
	N	Reassess to stop or how to continue if	Continue if	N
A1 and A2	15	< 2 responders, perform thorough assessment of available data and decide whether to stop, or to continue with same dose or with different dose*	≥ 2 responders, continue until additional 15 participants treated at the same dose are evaluable for response	30

* Dose ≤ MTD or highest tested dose declared safe

The cutoff for the interim analysis was chosen to balance sufficient information for decision with the potential of sparing participants an inefficient treatment. The probability of correctly stopping at the interim analysis (assuming an ORR of 10%) is 55% in Arm A1 and Arm A2. The interim analysis will be based on confirmed response according to RECIST v1.1, if available at time of data cutoff. If confirmed response is not available for all participants, then unconfirmed response will be used in these cases.

9.2.2 Part 2A: Dose Optimization

Overall, the RP2D selection will be informed by an overall assessment of available clinical antitumor activity, safety, long-term tolerability, PK, exposure-response analyses, and available PRO and other relevant data. The RP2D selection will include minimizing the risk to choose an inferior arm in terms of efficacy and safety.

The sample size for the dose optimization part in Part 2A (N=60; 30 participants per arm) is chosen by means of characteristics of posterior probabilities for differences in ORR and differences in Grade ≥ 3 Adverse Events of Interest rate (Section 8.3.7).

Table 25 shows the posterior probability that the lower dose is not inferior for different observed ORR differences. If the observed difference in ORR is 0%, the posterior probability is 82% that the difference in ORR between the higher and lower dose is less than 7%. Table 26 shows the posterior probability that the higher dose is not considered more toxic given observed Grade 3 neutropenia or dose modification differences. If the difference in the observed rate is ≤ 6.7%, the posterior probability is 81% that the difference in observed rate between the higher and lower dose is less than 15%.

Table 25 Posterior Probabilities for ORR Difference Between Higher And Lower Dose Given Observed Data.

Observed ORR (Lower dose vs. higher dose)	Observed ΔORR	Posterior probability that ORR difference is < 7%
3/30 vs. 6/30	10.0%	37%
3/30 vs. 5/30	6.7%	51%
3/30 vs. 4/30	3.3%	67%
3/30 vs. 3/30	0.0%	82%
3/30 vs. 2/30	-3.3%	93%
3/30 vs. 1/30	-6.7%	98%

Table 26 Posterior Probabilities for Difference In Grade ≥ 3 AEoI Or Dose Modification Rates Between Higher and Lower Dose Given Observed Data.

Observed rate (Lower dose vs. higher dose)	Observed Δrate	Posterior probability that rate difference is < 15%
4/30 vs. 7/30	10.0%	69%
4/30 vs. 6/30	6.7%	81%
4/30 vs. 5/30	3.3%	90%
4/30 vs. 4/30	0.0%	96%
4/30 vs. 3/30	-3.3%	99%
4/30 vs. 2/30	-6.7%	100%

Safety and tolerability of each dose regimen will be assessed by the SMC at selected time points (see Section 6.5.3). If a dosing regimen is stopped at the interim analysis as described in Section 9.2.1 or is deemed unsafe or intolerable by the SMC, further enrollment into that dosing regimen will be discontinued, and that dose regimen will not be considered for further dose selection. If both dose regimens are continued after the interim analysis and are considered safe and tolerable, then enrollment will continue until 30 evaluable participants have been enrolled in each Arm. An integrated analysis leveraging clinical efficacy and safety data,

exposure-response analyses, PRO-CTCAE and other relevant data will be performed at this point to inform the RP2D decision for further development. More details will be described in the IAP.

The RP2D needs to at least fulfill the following criteria:

- Median rate of DLT is ≤ 30%
- The overall rate of dose reductions/interruptions or discontinuations due to AEs/transfusions/G-CSF is < 50%

9.3 Analyses Sets

The analysis populations are specified below. The final decision to exclude participants from any analysis population will be made during a data review meeting prior to database lock except for the DLT analysis population. Decision for inclusion in DLT population depends on SMC recommendation.

Table 27 Description of the Analysis Sets

Analysis Set	Description
SCR	The Screening analysis set includes all participants who signed the informed consent.
SAF	All participants, who were administered any dose of any study intervention. Analyses will consider participants as treated.
DLT	The DLT Set will include all participants who received at least 1 dose of study intervention and meet at least one of the following criteria:<br>• Experienced at least 1 DLT confirmed by the SMC, regardless of the administered number of doses of study intervention/completion in the DLT period.<br>• Received at least 80% of the planned cumulative dose during the DLT period, completed the DLT period and did not have a delay of > 4 days in administration of infusion.<br>• Participants who did not receive 80% of the planned total dose of study intervention, but at least 80% dosing of a different dose cohort and finished the DLT period are eligible for the DLT analysis set to be analyzed in the highest dose cohort for which they received 80% of dosing.<br>The SMC will recommend the evaluability for dose escalation analysis (e.g., considering relevant deviations from dosing schedule).
PK	The PK Analysis Set (PKAS) will consist of all participants, who receive at least one dose of study intervention, and provide at least one measurable postdose concentration. A measurement below lower limit of quantification (BLQ) is considered a valid measurement. Participants will be analyzed per the actual study intervention they received.
Immunogenicity	All participants who receive at least one dose of study intervention and have at least one valid ADA result. All ADA analyses will be based on this analysis set.

ADA: antibody-drug conjugate, BLQ: below lower limit of quantification, DLT: dose-limiting toxicity, PK: pharmacokinetics, SMC: safety monitoring committee

9.4 Statistical Analyses

- In general, continuous variables will be summarized using number of participants (n); mean, standard deviation; median, 25th Percentile to 75th Percentile (Q1-Q3), minimum, and maximum. If there are less than 5 observations available only mean and the observed data will be given.
- Categorical variables will be summarized using frequency counts and percentages.
- The calculation of proportions will be based on the number of participants in the analysis set of interest, unless otherwise specified in the study IAP.
- Besides the details outlined below, more details will be specified in the IAP finalized before database lock.

9.4.1 Efficacy Analyses

Table 28 Efficacy Analyses

Estimand Reference # [a]	Category	Statistical Analysis Methods/Further Estimand Attributes
**4, 11, 26 – OR**	**Main**	Objective response rate will be determined as the proportion of participants with a confirmed objective response of PR or CR. Confirmation of response according to RECIST v1.1 will be required no sooner than 4 weeks after the initial documentation of CR or PR.<br><br>The 95% two-sided Confidence interval for the ORR will be calculated using the Clopper Pearson method.<br>The difference in ORRs between the 2 arms in Part 2A will be determined as well as the 95% CI using the normal approximation.<br>Posterior probabilities for the ORR to be above certain limits, given the observed data will be calculated.<br>The posterior probability for the ORR differences between dose levels in Part 2A will be determined using a normal approximation.
**4, 11, 26 - OR**	**Sensitivity**	IRC assessment, if implemented.
**13, 26 - DC**	**Main**	Disease control will be evaluated by the disease control rate (DCR) defined as the number of participants with disease control (CR, PR, SD, or non-CR/non-PD at week 12 visit) divided by the number of participants in the analysis population.<br>The 95% 2-sided Confidence interval for the DCR will be calculated using the Clopper Pearson method.
**5, 6, 11, 14, 15, 27, 28, 29 – DoR, TTR, PFS, OS**	**Main**	DoR is calculated for participants with confirmed objective response as the time from first documentation of objective response (CR or PR) to the date of first documentation of PD or death (due to any cause). If a participant has not had an event (PD or death), DoR is censored at the date of last adequate tumor assessment.<br>TTR will be calculated for participants with response as the time from the study intervention start date to the first documentation of objective response (CR or PR).

Estimand Reference # [a]	Category	Statistical Analysis Methods/Further Estimand Attributes
		TTR will be summarized using summary statistics (mean, SD, median, min, max. Q1, Q3).<br>Kaplan-Meier estimates (product-limit estimates) will be presented for the analysis of DoR, PFS, and OS together with a summary of associated statistics (median, survival time, and survival rate estimates at 3, 6, 12 months and every 6 months thereafter if applicable) including the corresponding 2-sided 95% CIs.<br>PFS and DoR data will be censored on the date of the last non-missing tumor assessment for participants who do not have an event (PD or death) or for participants for which the event is reported after 2 or more missed subsequent scheduled tumor assessments. Participants who do not have a baseline tumor assessment or who do not have any post baseline tumor assessments will be censored at the date of the start of study intervention.
**5, 6, 11, 14, 27, 28, 29 – DoR, TTR, PFS**	**Sensitivity**	IRC assessment, if implemented.
CCI [redacted]		

CI: confidence interval, CR: complete response, DC: disease control, DoR: duration of response, IRC: independent review committee; OR: objective response, ORR: objective response rate, OS: overall survival, PD: progressive disease, PFS: progression-free survival, PR: partial response, RECIST v1.1: Response Evaluation Criteria in Solid Tumors version 1.1, TTR: time to response

[a] See Table 12 and Table 13

9.4.2 Safety Analyses

Table 29 Safety Analyses

Estimand reference # [a]	Category	Statistical Analysis Methods/Further Estimand Attributes
**1, 2, 25 – DLT**	**Main**	Bayesian logistic regression analysis as described in Appendix 8.<br>At end of dose escalation analysis and main analysis, the number and proportion of participants experiencing DLTs will be reported by dose level, based on observations during the first study intervention cycle. Posterior probabilities (2.5%, 25%, 50%, 75%, 95%, and 97.5% quantiles) for DLT probabilities at selected doses will be estimated from the BLRM.
**1, 2, 25 – DLT**	**Sensitivity**	Separate BLRMs for Western and Japanese participants.<br>2 parameter frequentist modeling (without prior).<br>Separate BLRMs with and without pegfilgrastim<br>BLRM with actual received dose level (based on absolute dose) for capped participants.
**1, 2, 12, 25 – AEs, TRAEs**	**Main**	TEAES are defined as AEs emerging or worsening after start of treatment until 30 days after end of treatment. Adverse events will be coded according to the latest available version of MedDRA. Severity of AEs will be graded by

Estimand reference # [a]	Category	Statistical Analysis Methods/Further Estimand Attributes
		the Investigator using the NCI-CTCAE v.5.0 toxicity grades. TRAEs will be defined as any AE considered as related to M9140. Incidence of TEAEs and TRAEs will be summarized by SOC and PT.<br>The difference in incidence proportions between the 2 arms in Part 2A will be determined as well as the 95% CI using the normal approximation.<br>The posterior probability for the difference in incidence proportions between dose levels in Part 2A will be determined using a normal approximation.
**1, 2, 25 – Deaths**	**Main**	Counts and percentages.
Changes in laboratory measurements and vital signs	**Main**	Summary statistics and line plots.<br>Laboratory results will also be classified by Grade according to NCI-CTCAE. Worst on-treatment grades as well as shifts to worst on-treatment grades will be summarized. Measurements without NCI-CTCAE grading will be summarized by above, within, and below normal limits.
**8, 16 –** To assess the effect of M9140 on QTc interval	**Main**	Digital ECG measures, change from baseline QTc (ΔQTc) over time.

CCI [redacted]

a. See Table 12 for details on the objectives and endpoints/estimands.

AE: adverse event, BLRM: Bayesian logistic regression model, DLT: dose-limiting toxicity, ECG: electrocardiogram, CCI [redacted], MedDRA: Medical Dictionary for Regulatory Activities, PT: preferred term, QTc: corrected QT interval, SOC: system organ class, TEAE: treatment-emergent adverse event, TRAE: treatment-related adverse event.

9.4.2.1 Dose Escalation

Analyses to decide on dose escalation will be performed on the DLT set and will be based on available safety, and preliminary PK data. To support the recommendation on dose escalation, the SMC will receive results of a Bayesian dose-toxicity model, including the recommendation of the next dose level. This Bayesian two-parameter logistic regression model (BLRM) (Neuenschwander 2008) is further specified in Appendix 8. For each SMC meeting, the model will be updated with the number of DLTs and evaluable participants per dose level, considering participants with a capped dose in their assigned (non-capped) dose level. The following dose levels are foreseen: 0.6, 1.2, 1.8, 2.4, 3.0 mg/kg for dose levels DL1 to DL5, respectively, followed by 20% dose increments for additional dose levels (DLn). However, the SMC may recommend investigating doses that are different from the prespecified doses/dose steps. As sensitivity analyses, the SMC will in addition receive results from the same Bayesian model using data from only Japanese and only Western participants, respectively. For participants whose absolute dose was capped due to their BMI (as described in Section 4.1), the SMC will in addition receive results from the same Bayesian model, where capped participants are considered in the dose level (in mg/kg) that matches their actual received (capped) dose (in mg).

The dose suggested by the model for the next cohort will be based on minimizing the Bayesian Risk.

The SMC may recommend a different dose than suggested by the Bayesian escalation approach. The SMC can only recommend choosing a dose that is higher than the next planned one in the dose grid if the 75% quantile of P(DLT) of this higher dose is below 35% and the SMC considers it safe to do so. The maximum allowed dose increase will be limited as follows: CCI [redacted]. Also, the SMC may recommend investigating additional dosing regimens (e.g., CCI [redacted]). In such a case the dose-toxicity model will be extended, or a separate model will be set up.

The SMC will recommend the MTD. The target DLT probability for the suggested MTD by the Bayesian model is 30%. The prerequisites for MTD suggestion from the model are described in Appendix 8. See Appendix 11 for Japan-specific considerations.

Analyses for the Bayesian dose escalation will be performed on the DLT set. Usually, decisions on dose escalation are taken once all participants of the most recent cohort have completed the DLT period or dropped out. In exceptional cases, however, the SMC may recommend the next cohort earlier i.e., before the last participant of a cohort has finished the DLT period (considering the model recommendation). Per definition of the DLT set, participants who have not completed the DLT period are not included for update of the model, unless they experienced a DLT. However, data of such participants will be included at next SMC (if criteria for the dose escalation set are fulfilled).

Details on analyses for SMCs will be described in the SMC IAP.

Before first dosing, the assumed relationship between dose level and toxicity is specified through the prior distribution. The prior distribution chosen for this study for Part 1A (without pegfilgrastim prophylaxis) corresponds to the following:

CCI [redacted]

CCI [redacted]

A separate model will be set up for the Part 1B dose escalation, but the same specifications and the same prior parameter settings will be used for Part 1B BLRM as for Part 1A.

In case other regimens will be investigated, the Bayesian 2-parameter logistic regression model can be extended to include a binary covariate to account for the different regimen (Bailey 2009), or a new, separate model will be set up. The extended model is further specified in Appendix 8. In case the model will be extended, the target DLT probability for the MTD, and the escalation rules for recommending the next dose remain unchanged.

The prior distribution for a new regimen will either be based on the prior distribution from the Q3W regimen, or, if necessary, a new prior will be set up. The prior will be specified in the SMC charter prior to dosing of the first participant in the new regimen.

9.4.2.2 Dose Recommendations for Expansion

Part 2A

In Part 2A, a BLRM will support the SMC, e.g., when a change in dose or stopping for safety reasons is considered, by providing toxicity estimates for the doses. The same model as for the

dose escalation will be used, i.e., using the prior distribution from the dose escalation and the observed data from the dose escalation.

Part 2B

In Part 2B, a BLRM will support the SMC, by providing toxicity estimates for the doses. The Bayesian 2-parameter logistic regression model from dose escalation will be extended to include a binary covariate to account for the different regimen (Bailey 2009), or a new, separate model will be set up. The extended model is further specified in Appendix 8. In case the model will be extended, the target DLT probability for the MTD, and the escalation rules for recommending the next dose remain unchanged. For participants whose absolute dose was capped due to their BMI (as described in Section 4.1), the SMC will in addition receive results from the same Bayesian model, where capped participants are considered in the dose level (in mg/kg) that matches their actual received (capped) dose (in mg).

The prior distribution for a new regimen will either be based on the posterior distribution from the Q3W regimen, or, if necessary, a new prior will be set up. The prior will be specified in the SMC charter or IAP prior to dosing of the first participant in the new regimen.

Part 2C1:

For Part 2C1, it is expected that only 2 dose levels will be tested, which would lead to insufficient data to sensibly fit a BLRM. Therefore, the SMC will be supported by a Bayesian optimal interval design with informative prior (iBOIN, Zhou 2021).

There are 2 planned dose levels and 1 fallback dose level, in case the first dose level is not tolerated well, i.e., 3 dose levels in total with a planned cohort size of 3 participants:

i. M9140 dose below 2.4 mg/kg + Bevacizumab 7.5 mg/kg
ii. **[starting dose]** M9140 2.4 mg/kg + Bevacizumab 7.5 mg/kg
iii. M9140 2.8 mg/kg + Bevacizumab 7.5 mg/kg

It is possible that not all listed dose levels will be tested. The SMC may suggest a different M9140 dose than what is pre-specified, in which case this dose level will be added to the decision rules. The prior toxicity assumption for this dose level will be based on the weighted average of the neighboring dose levels (weighing based on distance to the neighboring doses).

The target DLT rate for the MTD is $\phi = 0.3$. The prior DLT rate for the 3 planned doses and the respective prior effective sample sizes will be based on the available data from Part 1 and Part 2A and will be specified in the IAP or SMC charter prior to dosing of the first participant in Part 2C.

As shown in Figure 5, the iBOIN design uses the following rules, optimized to minimize the probability of incorrect dose assignment, to guide dose escalation/de-escalation:

- if the observed DLT rate at the current dose is $\leq \lambda_e$, escalate the dose to the next higher dose level;

- if the observed DLT rate at the current dose is $>\lambda_d$, de-escalate the dose to the next lower dose level;
- otherwise, stay at the current dose.

The values for λ_e and λ_d vary with dose level and the number of participants treated on a dose. A table with the decision boundaries (taking into account the informative prior) which will be used in the trial for dose escalation decisions will also be specified in the IAP or SMC charter prior to dosing of the first participant in Part 2C.

Figure 5 **Flowchart for trial conduct using the iBOIN design.**

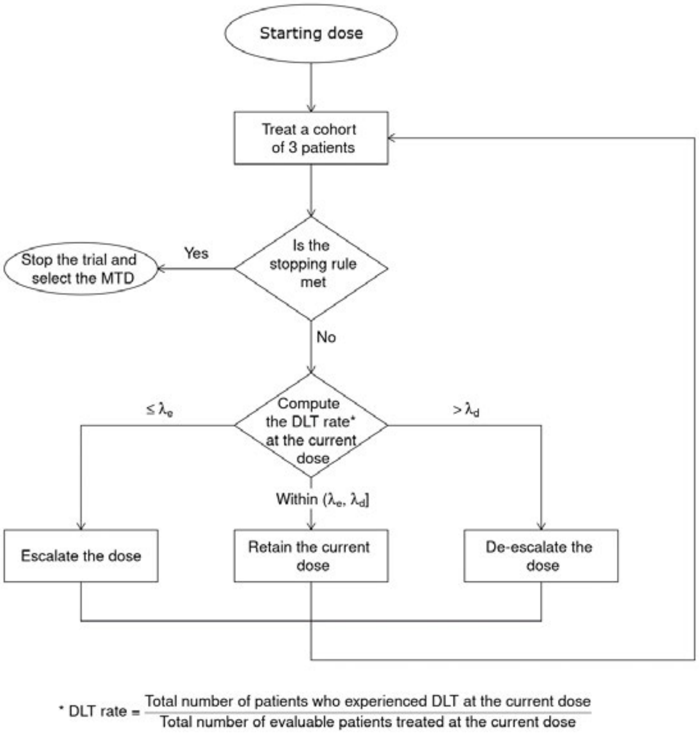

The steps to implement the iBOIN design are described as follows:

- Participants in the first cohort are treated at the starting dose.
- To assign a dose to the next cohort of participants, conduct dose escalation/de-escalation according to the table containing the decision boundaries.

Please note the following:

- If none of the actions (i.e., escalation, de-escalation, or elimination) is triggered, treat the new participants at the current dose.
- If the current dose is the lowest dose and the rule indicates dose de-escalation, treat the new participants at the lowest dose unless the number of DLTs reaches the elimination boundary, at which point terminate the trial for safety.

- If the current dose is the highest dose and the rule indicates dose escalation, treat the new participants at the highest dose.
- Repeat Step 2 until the maximum sample size is reached, or the trial is stopped early due to the elimination of the lowest dose.

Additional participants can be enrolled to collect further safety and efficacy data, not exceeding the maximum sample size specified in Section 9.2. The table containing the decision boundaries will still be applied for toxicity monitoring.

Part 2C2

For Part 2C2, a Bayesian CRM (continual reassessment method) model (O'Quigley 1990) with overdose control (EWOC) (Babb 1998) will support the SMC. It is further specified in Appendix 8. The CRM is a model-based approach, similar to the BLRM used in the other study parts. In contrast to the BLRM, the CRM uses numerical dose labels d_i which are not the actual doses administered, but rather are defined on a conceptual scale that represents an ordering of the risks of toxicity i.e., since the CRM operates on a discrete set of dose levels, a physical interpretation for the dose labels is not required, as long as they constitute a strictly increasing sequence. Therefore, it can be used for dose escalation settings with combination dose levels, where the ordering of the dose levels with respect to toxicity is straightforward. Based on the available data from Part 1 and the publicly available data on the SoC components used for combination, the following orderings are assumed (from lower to higher toxicity):

i. M9140 2.0 mg/kg + Capecitabine 800 mg/m^2 + Bevacizumab 7.5 mg/kg

ii. **[starting dose]** M9140 2.4 mg/kg + Capecitabine 800 mg/m^2+ Bevacizumab 7.5 mg/kg

iii. M9140 2.4 mg/kg + Capecitabine 1,000 mg/m^2+ Bevacizumab 7.5 mg/kg

iv. M9140 2.8 mg/kg + Capecitabine 1,000 mg/m^2 + Bevacizumab 7.5 mg/kg

It is possible that not all listed dose levels will be tested, and the starting dose may be lowered depending on emerging data from Part 2C1. The SMC may suggest a different M9140 dose, which will be added to the ordering based on the same logic, i.e., between the dose levels with the next lower and higher M9140 doses. If, after testing dose level iv., the SMC suggests de-escalating (only) capecitabine, resulting in the dose level M9140 2.8 mg/kg + capecitabine 800 mg/m^2 + bevacizumab 7.5 mg/kg, the CRM will be modified, to include the dose in the ordering between iii. and iv., and in addition as sensitivity analysis between ii. and iii., in the skeleton, which will be specified in the SMC charter or IAP prior to the first SMC in Part 2C.

Prior assumptions on the relationship between dose level combinations and toxicity will be specified through a so-called skeleton, a strictly increasing sequence, containing the prior assumptions on the DLT probability for each dose level (combination). These assumptions will be based on all available data from Part 1 and Part 2A, and publicly available data on capecitabine, bevacizumab, and the related XELIRI regimen. The skeleton will be specified in the SMC charter or the IAP prior to dosing of the first participant in Part 2C.

For participants whose absolute dose was capped due to their BMI (as described in Section 4.1), the SMC will in addition receive results from the same CRM model, where capped participants are considered in the dose level (in mg/kg) that matches their actual received (capped) dose (in mg).

More details about the CRM with EWOC can be found in Appendix 8.

Part 2D

For Part 2D, a Bayesian CRM model will support the SMC, using the same methodology as in Part 2C2. Based on the available data from Part 1 and the publicly available data on the SoC components used for combination, the following orderings are assumed (from lower to higher toxicity):

[redacted]

It is possible that not all listed dose levels will be tested, and the starting dose may be lowered depending on emerging data from Part 2B and Part 2C1. The SMC may suggest a different M9140 dose, which will be added to the ordering based on the same logic, i.e., between the dose levels with the next lower and higher M9140 doses. If, after testing dose level iv, the SMC suggests de-escalating (only) 5-FU, resulting in the dose level [redacted], the CRM will be modified, to include the dose in the ordering between iii. and iv., and in addition as sensitivity analysis between ii. and iii., in the skeleton, which will be specified in the SMC charter or IAP prior to the first SMC in Part 2D.

Prior assumptions on the relationship between dose level combinations and toxicity will be specified through a so-called skeleton, a strictly increasing sequence, containing the prior assumptions on the DLT probability for each dose level (combination). These assumptions will be based on all available data from Part 1, Part 2A and 2B, and publicly available data on 5-FU, bevacizumab, and the related FOLFIRI regimen. The skeleton will be specified in the SMC charter or the IAP prior to dosing of the first participant in Part 2D. For participants whose absolute dose was capped due to their BMI (as described in Section 4.1), the SMC will in addition receive results from the same CRM model, where capped participants are considered in the dose level (in mg/kg) that matches their actual received (capped) dose (in mg).

9.4.3 Other Analyses

Details on the PK, immunogenicity, [redacted] analyses will be in the IAP that will be finalized before database lock. [redacted].

Pharmacokinetic profile

Estimation of Individual PK Parameters:

Pharmacokinetic parameters for M9140 (conjugated antibody, unconjugated payload, and total antibody) will be calculated using standard non-compartmental methods and the actual administered dose, for each dose level. PK parameters will be calculated using the actual elapsed time since dosing. When the actual sampling time is missing, calculations will be performed using the scheduled time. For samples to be collected within a time range the nominal midpoint will be used for PK evaluation, in case where the actual sampling time is missing. Otherwise, there will be no further imputation of missing data.

Non-compartmental computation of PK parameters will be performed using the computer program CCI [redacted].

The statistical software SAS® (Statistical Analysis System, SAS-Institute, Cary NC, USA, windows version 9.1 or higher) may be used to produce tables, listings, and figures and in the calculation of PK Parameters if appropriate.

Estimation of renal clearance:

For unconjugated payload in urine, PK parameters will be calculated to estimate the renal clearance, i.e., urine volume and amount of payload excreted during defined collection periods.

Population PK analysis

Population PK models for M9140 (conjugated antibody and unconjugated payload) will tentatively be constructed. Exposure-safety and exposure-efficacy relationships will be explored, as permitted by the data.

Details of the analysis will be included in the Population Modeling and Simulation Analysis Plan. Results will be reported separately from the CSR.

Immunogenicity (ADA)

The participants who are pre-existing positive, transient treatment-emergent positive, or persistent treatment-emergent positive will be listed. Titers of ADA-positive samples will be reported. The impact of ADA formation on PK will be evaluated as permitted by the data. The details will be described in the IAP.

Patient Reported Outcomes (PROs)

The 9 PRO-CTCAE items measuring the severity and interference of 5 symptoms as described in Section 8.2.5 will be scored from 0 to 4 and scores for each attribute (frequency, severity and/or interference) will be presented descriptively using appropriate summary statistics. PRO-CTCAE analyses will consider participants as actually treated to inform tolerability

objectives. Analyses will be performed on the Safety Analysis set. Further details will be provided in the IAP. The proportion of missing data will also be summarized to aid interpretation.

9.4.4 Sequence of Analyses

SMCs: The SMC will review available data during study conduct.

- During Part 1, the cutoff for dose escalation assessments by the SMC will usually be triggered by the completion of the DLT period (or dropout) of the last participant in the respective dose escalation cohort of usually 3 participants. When enrollment of the last participant in a dosing cohort is delayed, the SMC may recommend (based on available data) enrollment and dose for the next dosing cohort before all participants in a cohort have completed Cycle 1. The Bayesian model will then be updated with the available data, and the data from the participant not having completed the DLT period at time of SMC will be considered in the next SMC. In these cases, the cutoff can be earlier (after the DLT period of the first 2 participants are finished or they experienced a DLT).
- During Part 2A, the cutoff for the safety assessments will be triggered when 8 and 15 participants in each Arm of Part 2A have completed the first cycle of therapy or dropped out. Additional safety assessments can be scheduled as needed.
- During Parts 2B, 2C, and 2D, the cutoff for dose escalation assessments by the SMC will usually be triggered by the completion of the DLT period (or dropout) of the last participant in the respective dose escalation cohort of usually 3 participants, unless the SMC decides to change the size of the cohorts. When enrollment of the last participant in a dosing cohort is delayed, the SMC may recommend (based on available data) enrollment and dose for the next dosing cohort before all participants in a cohort have completed the DLT period. The statistical model will then be updated with the available data, and the data from the participant not having completed the DLT period at time of SMC will be considered in the next SMC. In these cases, the cutoff can be earlier (after the DLT period of the first 2 participants are finished or they experienced a DLT).
- End of dose escalation (Part 1): The cutoff for an exploratory analysis of the safety, available PK, and preliminary antitumor activity data from the complete dose escalation (both Part 1A and Part 1B) will be triggered when all participants enrolled in dose escalation have reached either the first on-study intervention tumor assessment or experienced death or premature withdrawal for any reason, whichever comes first.
- Interim Analysis (Part 2A): The cut-off for the interim analysis in Part 2A in each arm will be triggered when 15 participants of the respective arm are evaluable for response, i.e., have had at least one on-study tumor assessment or have dropped out. Enrollment will continue during preparation of the interim analysis.
- End of dose optimization (Part 2A): If other study parts are still ongoing at this time, the cut off for an exploratory analysis of the safety, available PK, and preliminary antitumor activity data from the dose optimization (Part 2A) will be last subject last visit or 13 weeks after last subject first dose in Part 2A.
- Primary Analysis: The cutoff for the primary analysis will be last subject last visit or 19 weeks after last subject first dose in Part 2, whichever comes first.

- Follow-up analyses to report further efficacy and safety data will be done once the End of Study has been reached.

CCI [redacted].

More details will be described in the IAP.

10 References

Aapro MS, Bohlius J, Cameron DA, et al. 2010 update of EORTC guidelines for the use of granulocyte-colony stimulating factor to reduce the incidence of chemotherapy-induced febrile neutropenia in adult patients with lymphoproliferative disorders and solid tumours. Eur J Cancer. 2011;47:8-32.

Ajani JA, Takimoto C, Becerra CR, et al. A phase II clinical and pharmacokinetic study of intravenous exatecan mesylate (DX-8951f) in patients with untreated metastatic gastric cancer. Invest New Drugs. 2005;23:479-84.

Aparicio T, Bouché O, Taieb J, et al; for PRODIGE 20 Investigators. Bevacizumab+chemotherapy versus chemotherapy alone in elderly patients with untreated metastatic colorectal cancer: a randomized phase II trial-PRODIGE 20 study results. Ann Oncol. 2018;29:133-8.

Arnold D, Prager GW, Quintela A, et al. Beyond second-line therapy in patients with metastatic colorectal cancer: a systematic review. Ann Oncol. 2018;29:835-56.

Babb J, Rogatko A, Zacks S. Cancer phase I clinical trials: efficient dose escalation with overdose control. Stat Med. 1998;17:1103-20.

Bailey S, Neuenschwander B, Laird G, et al. A Bayesian case study in oncology Phase I combination dose-finding using logistic regression with covariates. J Biopharm Stat. 2009;19:469-84.

Basch E, Reeve BB, Mitchell SA,et al. Development of the National Cancer Institute's patient-reported outcomes version of the common terminology criteria for adverse events (PRO-CTCAE). J Natl Cancer Inst. 2014;106:dju244.

Beauchemin N, Arabzadeh A. Carcinoembryonic antigen-related cell adhesion molecules (CEACAMs) in cancer progression and metastasis. Cancer Metastasis Rev. 2013;32:643-71.

Boige V, Raymond E, Faivre S, et al. Phase I and pharmacokinetic study of the camptothecin analog DX-8951f administered as a 30-minute infusion every 3 weeks in patients with advanced cancer. J Clin Oncol. 2000;18:3986-92.

Bové DS, Yeung WY, Palermo G, et al. Model-based dose escalation designs in R with crmPack. J Stat Softw. 2019;89:1-22.

Braybrooke JP, Boven E, Bates NP, et al. Phase I and pharmacokinetic study of the topoisomerase I inhibitor, exatecan mesylate (DX-8951f), using a weekly 30-minute intravenous infusion, in patients with advanced solid malignancies. Ann Oncol. 2003;14:913-21.

Cervantes A, Adam R, Roselló S, et al. Metastatic colorectal cancer: ESMO Clinical Practice Guideline for diagnosis, treatment and follow-up. Ann Oncol. 2023;34:10-32.

Chau I, Norman AR, Cunningham D, et al. A randomised comparison between 6 months of bolus fluorouracil/leucovorin and 12 weeks of protracted venous infusion fluorouracil as adjuvant treatment in colorectal cancer. Ann Oncol. 2005;16:549-57.

Chiba K, Yoshitsugu H, Kyosaka Y, et al. A comprehensive review of the pharmacokinetics of approved therapeutic monoclonal antibodies in Japan: Are Japanese phase I studies still needed? J Clin Pharmacol. 2014;54:483-94.

Criscitiello C, Morganti S, Curigliano G. Antibody-drug conjugates in solid tumors: a look into novel targets. J Hematol Oncol. 2021 ;14 :20.

De Jager R, Cheverton P, Tamanoi K, et al. DX-8951f: summary of phase I clinical trials. Ann N Y Acad Sci. 2000;922:260-73.

Decary S, Berne PF, Nicolazzi C, et al. Preclinical Activity of SAR408701: A Novel Anti-CEACAM5-maytansinoid Antibody-drug Conjugate for the Treatment of CEACAM5-positive Epithelial Tumors. Clin Cancer Res. 2020;26:6589-6599.

Dekker E, Tanis PJ, Vleugels JLA, et al. Colorectal cancer. Lancet. 2019;394:1467-80.

Dueck AC, Mendoza TR, Mitchell SA, et al. Validity and Reliability of the US National Cancer Institute's Patient-Reported Outcomes Version of the Common Terminology Criteria for Adverse Events (PRO-CTCAE). JAMA Oncol. 2015;1:1051-9.

Eisenhauer EA, Therasse P, Bogaerts J, et al. New response evaluation criteria in solid tumours: Revised RECIST guideline (version 1.1). Eur J Cancer. 2009 ;45 :228-47.

Esteva FJ, Rivera E, Cristofanilli M, et al. A Phase II study of intravenous exatecan mesylate (DX-8951f) administered daily for 5 days every 3 weeks to patients with metastatic breast carcinoma. Cancer. 2003;98:900-7.

Fernández-Montes A, Grávalos C, Pericay C, et al. Current Options for Third-line and Beyond Treatment of Metastatic Colorectal Cancer. Spanish TTD Group Expert Opinion. Clin Colorectal Cancer. 2020;19:165-77.

Garrison MA, Hammond LA, Geyer CE Jr, et al. A Phase I and pharmacokinetic study of exatecan mesylate administered as a protracted 21-day infusion in patients with advanced solid malignancies. Clin Cancer Res. 2003 ;9 :2527-37.

Giles FJ, Cortes JE, Thomas DA, et al. Phase I and pharmacokinetic study of DX-8951f (exatecan mesylate), a hexacyclic camptothecin, on a daily-times-five schedule in patients with advanced leukemia. Clin Cancer Res. 2002;8:2134-41.

Heinemann V, von Weikersthal LF, Decker T, et al. FOLFIRI plus cetuximab versus FOLFIRI plus bevacizumab as first-line treatment for patients with metastatic colorectal cancer (FIRE-3): a randomised, open-label, phase 3 trial. Lancet Oncol. 2014;15:1065-75.

Janjigian YY, Oaknin A, Lang JM, et al. TROPION-PanTumor03: Phase 2, multicenter study of datopotamab deruxtecan (Dato-DXd) as monotherapy and in combination with anticancer agents in patients (pts) with advanced/metastatic solid tumors. J Clin Oncol. 2023;41:16 Suppl. TPS3153

Janjigian YY, Oh DY, Rha SY, et al. Dose-escalation and dose-expansion study of trastuzumab deruxtecan (T-DXd) monotherapy and combinations in patients (pts) with advanced/metastatic HER2+ gastric cancer (GC)/gastroesophageal junction adenocarcinoma (GEJA): DESTINY-Gastric03. J Clin Oncol. 2022;40:4 Suppl. 295

Kopetz S, Boni V, Kato K, et al. First-in-human trial of M9140, an anti-CEACAM5 antibody drug conjugate (ADC) with exatecan payload, in patients (pts) with metastatic colorectal cancer (mCRC). Meeting Abstract: 2024 ASCO Annual Meeting I. 2024;42(16_suppl)

Köhne CH, Bedenne L, Carrato A, et al. A randomised phase III intergroup trial comparing high-dose infusional 5-fluorouracil with or without folinic acid with standard bolus 5-fluorouracil/folinic acid in the adjuvant treatment of stage III colon cancer: the Pan-European Trial in Adjuvant Colon Cancer 2 study. Eur J Cancer. 2013;49:1868-75.

Mahmood I. Effect of Intrinsic and Extrinsic Factors on the Pharmacokinetics of Antibody-Drug Conjugates (ADCs). Antibodies (Basel). 2021 ;10 :40.

Masuishi T, Tsuji A, Kotaka M, et al. Phase 2 study of irinotecan plus cetuximab rechallenge as third-line treatment in KRAS wild-type metastatic colorectal cancer: JACCRO CC-08. Br J Cancer. 2020;23:1490-95.

Minami H, Fujii H, Igarashi T, et al. Phase I and pharmacological study of a new camptothecin derivative, exatecan mesylate (DX-8951f), infused over 30 minutes every three weeks. Clin Cancer Res. 2001;7:3056-64.

Mitsui I, Kumazawa E, Hirota Y, et al. A new water-soluble camptothecin derivative, DX-8951f, exhibits potent antitumor activity against human tumors in vitro and in vivo. Jpn J Cancer Res. 1995;86:776-82.

Modest DP, Fischer von Weikersthal L, Decker T, et al; XELAVIRI/AIO KRK0110 Investigators. Sequential Versus Combination Therapy of Metastatic Colorectal Cancer Using Fluoropyrimidines, Irinotecan, and Bevacizumab: A Randomized, Controlled Study-XELAVIRI (AIO KRK0110). J Clin Oncol. 2018;37:22-32.

Mosteller RD. Simplified calculation of body-surface area. N Engl J Med. 1987;317:1098.

National Comprehensive Cancer Network. Colon Cancer (Version 3.2024). https://www.nccn.org/professionals/physician_gls/pdf/colon.pdf. Accessed June 18, 2024.

Neuenschwander B, Branson M, Gsponer T. Critical aspects of the Bayesian approach to phase I cancer trials. Stat Med. 2008;27:2420-39.

O'Quigley J, Pepe M, Fisher L. Continual reassessment method: a practical design for phase 1 clinical trials in cancer. Biometrics. 1990;46:33-48

Pommier Y. Topoisomerase I inhibitors: camptothecins and beyond. Nat Rev Cancer. 2006;6:789-802.

Poplin EA, Benedetti JK, Estes NC, et al. Phase III Southwest Oncology Group 9415/Intergroup 0153 randomized trial of fluorouracil, leucovorin, and levamisole versus fluorouracil continuous infusion and levamisole for adjuvant treatment of stage III and high-risk stage II colon cancer. J Clin Oncol. 2005;23(9):1819-25.

Prasanna T, Karapetis CS, Roder D, et al. The survival outcome of patients with metastatic colorectal cancer based on the site of metastases and the impact of molecular markers and site of primary cancer on metastatic pattern. Acta Oncol. 2018;57:1438-44.

Raab-Westphal S, Hart F, Sloot W, et al. Preclinical efficacy and safety of M9140, a novel antibody-drug conjugate (ADC) with topoisomerase 1 (TOP1) inhibitor payload targeting carcinoembryonic antigen-related cell adhesion molecule 5 (CEACAM5)-expressing colorectal tumors. Abstract #2362. AACR 2024 Meeting Proceedings (available at

https://www.aacr.org/wp-content/uploads/2024/04/AACR2024_Regular_Abstracts_04-01-24.pdf) Accessed 7-June-2024

Rowinsky EK, Johnson TR, Geyer CE Jr, et al. DX-8951f, a hexacyclic camptothecin analog, on a daily-times-five schedule: a phase I and pharmacokinetic study in patients with advanced solid malignancies. J Clin Oncol. 2000;18:3151-63.

Rowinsky, EK. (2005). Preclinical and clinical development of exatecan (DX-8951f). In Camptothecins in Cancer Therapy (pp. 317-341). Humana Press.

Sweeting M, Mander A, Sabin T. Bcrm: Bayesian continual reassessment method designs for phase I dose-finding trials. J Stat Softw. 2013;54:(13);1-26.

Royce ME, Hoff PM, Dumas P, et al. Phase I and pharmacokinetic study of exatecan mesylate (DX-8951f): a novel camptothecin analog. J Clin Oncol. 2001;19:1493-500.

Royce ME, Rowinsky EK, Hoff PM, et al. A phase II study of intravenous exatecan mesylate (DX-8951f) administered daily for five days every three weeks to patients with metastatic adenocarcinoma of the colon or rectum. Invest New Drugs. 2004;22:53-61.

Saber H, Leighton JK. An FDA oncology analysis of antibody-drug conjugates. Regul Toxicol Pharmacol. 2015;71:444-52.

SEER database. https://seer.cancer.gov/statfacts/html/colorect.html (Accessed June 18, 2024).

Sharma S, Kemeny N, Schwartz GK, et al. Phase I study of topoisomerase I inhibitor exatecan mesylate (DX-8951f) given as weekly 24-hour infusions three of every four weeks. Clin Cancer Res. 2001;7:3963-70.

Smith TJ, Bohlke K, Lyman GH, et al. American Society of Clinical Oncology. Recommendations for the Use of WBC Growth Factors: American Society of Clinical Oncology Clinical Practice Guideline Update. J Clin Oncol. 2015;33:3199-212.

Sorich MJ, Wiese MD, Rowland A, et al. Extended RAS mutations and anti-EGFR monoclonal antibody survival benefit in metastatic colorectal cancer: a meta-analysis of randomized, controlled trials. Ann Oncol. 2015;26:13-21.

Tabernero J, Grothey A, Van Cutsem E, et al. Encorafenib plus cetuximab as a new standard of care for previously treated BRAF V600E-mutant metastatic colorectal cancer: updated survival results and subgroup analyses from the BEACON study. J Clin Oncol. 2021;39:273-84.

Tchoupa AK, Schuhmacher T, Hauck CR. Signaling by epithelial members of the CEACAM family – mucosal docking sites for pathogenic bacteria. Cell Commun Signal. 2014;12:27.

Van Cutsem E, Cervantes A, Nordlinger B, et al. Metastatic colorectal cancer: ESMO Clinical Practice Guidelines for diagnosis, treatment and follow-up. Ann Oncol. 2014;25 Suppl 3:iii1-9.

Verschraegen CF, Kudelka AP, Hu W, et al. A phase II study of intravenous exatecan mesylate (DX-8951f) administered daily for 5 days every 3 weeks to patients with advanced ovarian, tubal, or peritoneal cancer resistant to platinum, taxane and topotecan. Cancer Chemother Pharmacol. 2004;53:1-7.

Weinberg BA, Marshall JL, Salem ME. Trifluridine/tipiracil and regorafenib: new weapons in the war against metastatic colorectal cancer. Clin Adv Hematol Oncol. 2016;14:630-8.

Xu J, Kim TW, Shen L, et al. Results of a randomized, double-blind, placebo-controlled, Phase III trial of trifluridine/tipiracil (TAS-102) monotherapy in Asian patients with previously treated metastatic colorectal cancer: The TERRA Study. J Clin Oncol. 2018;36:350-358.

Xu RH, Muro K, Morita S, et al. Modified XELIRI (capecitabine plus irinotecan) versus FOLFIRI (leucovorin, fluorouracil, and irinotecan), both either with or without bevacizumab, as second-line therapy for metastatic colorectal cancer (AXEPT): a multicentre, open-label, randomised, non-inferiority, phase 3 trial. Lancet Oncol. 2018;19:660-671.

Yoshino T, Arnold D, Taniguchi H, et al. Pan-Asian adapted ESMO consensus guidelines for the management of patients with metastatic colorectal cancer: a JSMO-ESMO initiative endorsed by CSCO, KACO, MOS, SSO and TOS. Ann Oncol. 2018;29:44-70.

Zhou J, Fan X, Chen N, et al. Identification of CEACAM5 as a biomarker for prewarning and prognosis in gastric cancer. J Histochem Cytochem. 2015;63:922-30.

Zhou Y, Lee JJ, Wang S, et al. Incorporating historical information to improve phase I clinical trials. Pharm Stat. 2021;20:1017-34.

11 Appendices

Appendix 1 Abbreviations

1L	First Line
2L	Second Line
3L	Third Line
4L	Fourth Line
5-FU	5-Fluorouracil
ADA	Anti-Drug Antibody
ADC	Antibody-Drug Conjugate
AE	Adverse Event
AESI	Adverse Events of Special Interest
BLQ	Below Lower Limit of Quantification
BLRM	Bayesian Logistic Regression Model
BMI	Body Mass Index
BSA	Body Surface Area
BSC	Best Supportive Care
C	Cycle
CBC	Complete Blood Count
CDX	Cell Line-derived Xenograft
CEA	Carcinoembryonic Antigen
CEACAM	Carcinoembryonic Antigen-Related Cell Adhesion Molecule 5
CI	Confidence Interval
CIOMS	Council for International Organizations of Medical Sciences
CRC	Colorectal Cancer
CRF	Case Report Form
CRM	Continual Reassessment Method
CRO	Clinical Research Organization
CT	Computed Tomography, Chemotherapy
[redacted]	Circulating Tumor DNA
CV	Cardiovascular

CYP	Cytochrome P450
DAR	Drug Antibody Rate
DCR	Disease Control Rate
DL	Dose Level
DLT	Dose-Limiting Toxicity
DNA	Deoxyribonucleic Acid
DoR	Duration of Response
ECG	Electrocardiogram
ECOG PS	Eastern Cooperative Oncology Group Performance Status
eCRF	Electronic Case Report Form
EGFR	Epidermal Growth Factor Receptor
EOI	End of Infusion
EOT	End of Treatment
EU	European Union
EudraCT	European Clinical Trials Database
FFPE	Formalin-fixed Paraffin-embedded
FIH	First in Human
GC	Gastric Cancer
GCP	Good Clinical Practice
G-CSF	Granulocyte Colony-stimulating Factor
GEJC	Gastroesophageal Junction Cancer
GM-CSF	Granulocyte-macrophage Colony-stimulating Factor
HA	Health Authority
HNSTD	Highest Non-Severely Toxic Dose
HR	Hazard Ratio
IAP	Integrated Analysis Plan
IB	Investigator's Brochure
ICF	Informed Consent Form
ICH	International Council for Harmonization
IDMC	Independent Data Monitoring Committee
IEC	Independent Ethics Committee

IHC	Immunohistochemistry
IL-11	Interleukin 11
ILD	Interstitial Lung Disease
IMP	Investigational Medicinal Product
IMPD	Investigational Medicinal Product Dossier
INR	International Normalized Ratio
IRB	Institutional Review Board
IRC	Independent Review Committee
IRR	Infusion-Related Reactions
LSFD	Last Subject First Dose
LSLV	Last Subject Last Visit
mCRC	Metastatic Colorectal Cancer
MoA	Mechanism of Action
mOS	Median Overall Survival
MRI	Magnetic resonance imaging
MSI-H	Microsatellite Instability High
MTD	Maximum Tolerated Dose
N	Number
NCCN	National Comprehensive Cancer Network
NCI-CTCAE	National Cancer Institute Common Terminology Criteria for Adverse Events
NOAEL	No-Observed-Adverse-Effect Level
NSCLC	Non-Small Cell Lung Cancer
NYHA	New York Heart Association
OR	Overall Response
ORR	Overall Response Rate
OS	Overall Survival
PD	Progressive Disease
PDX	Patient-derived Xenograft
PFS	Progression-free survival
PK	Pharmacokinetic

PR	Partial Response
CCI [redacted]	[redacted]
Q3W	Every 3 Weeks
CCI [redacted]	[redacted]
QTc	Corrected QT interval
RDE	Recommended Dose for Expansion
RECIST	Response Evaluation Criteria in Solid Tumor
RNA	Ribonucleic Acid
RP2D	Recommended Phase 2 Dose
SAE	Serious Adverse Event
CCI [redacted]	[redacted]
SD	Stable Disease
SMC	Safety Monitoring Committee
SmPC	Summary of Product Characteristics
SoA	Schedule of Activities
SoC	Standard of Care
SOC	System Organ Class
SUSAR	Suspected Unexpected Serious Adverse Reactions
TEAE	Treatment-emergent Adverse Event
TRAE	Treatment-related Adverse Event
TOP1	Topoisomerase 1
TOP1i	Topoisomerase-1 Inhibitor
US	United States
VEGF	Vascular Endothelial Growth Factor
WOCBP	Woman of Childbearing Potential
wt	Wild Type

Appendix 2 Study Governance

Financial Disclosure

Investigators and Sub-Investigators will provide the Sponsor with sufficient, accurate financial information, as requested, for the Sponsor to submit complete and accurate financial certification or disclosure statements to the appropriate regulatory authorities. This information is required during the study and for 1 year after completion of the study.

Informed Consent Process

- The Investigator or his/her representative will explain the nature of the study to the participant and answer all questions on the study.
- Participants will be informed that their participation is voluntary.
- Participants will be required to sign a statement of informed consent that meets the requirements of 21 CFR 50; the Japanese ministerial ordinance on GCP; local regulations; ICH guidelines; HIPAA requirements, where applicable; and the IRB/IEC or study center.
- The medical record will include a statement that written informed consent was obtained before the participant was enrolled in the study and the date the written consent was obtained.
- If the ICF is updated during their participation in the study, participants will be re-consented to the most current, approved version.
- Participants who are rescreened are required to sign a new ICF.

Data Protection

- The Sponsor will assign a unique identifier to participants after obtaining their informed consent. Any participant records or datasets that are transferred to the Sponsor will contain the identifier only; participant names or any identifiable information will not be transferred.
- The Sponsor will inform participants that their personal study-related data will be used per local data protection and privacy laws. The level of disclosure will also be explained to the participant and pregnant partners (if applicable), who will be required to give consent for their data to be used, as specified in the informed consent.
- The participant will be informed that his/her medical records may be examined by Clinical Quality Assurance auditors or other Sponsor-appointed, authorized personnel, by appropriate IRB/IEC members, and by regulatory authority inspectors. All such persons will strictly maintain participants' confidentiality.
- The Investigator and Sponsor will comply with all applicable regulations to protect personal data. If a data security breach occurs at the site, the Investigator will inform the Sponsor within 24 hours after becoming aware of the event. The Sponsor will manage the breach in accordance with their processes, including where applicable regulatory authority and/or IRB/EC notification.

Study Administrative

- The Coordinating Investigator listed on the title page represents all Investigators for decisions and discussions on this study, per ICH GCP. The Coordinating Investigator will provide expert medical input and advice on the study design and execution and is responsible for the review and signoff of the clinical study report.
- A Safety Monitoring Committee (SMC) will be formed in this study. For details on the SMC see Section 6.5.3.
- Details of structures and associated procedures will be defined in a separate Clinical Operations Manual.

Regulatory and Ethical Considerations

- This study will be conducted in accordance with the protocol and the following:
- Consensus ethical principles derived from international guidelines, including the Declaration of Helsinki and CIOMS International Ethical Guidelines
- Applicable ICH GCP Guidelines
- For studies with Japanese sites, the Japanese ministerial ordinance on GCP
- For studies with EU member states sites, with Regulation [EU] No 536/2014
- Applicable laws and regulations
- The protocol, protocol amendments (if applicable), ICF, Investigator Brochure, and other relevant documents (e.g., advertisements) will be submitted to an IRB/IEC for review and approval before the study is initiated.
- For studies with Japanese sites, the Sponsor initiates the study at a site after obtaining written approval from the Head of the study site, based on favorable opinion/approval from the concerned IRB.
- Any protocol amendments (i.e., changes to the protocol) will be documented in writing and require IRB/IEC approval before implementation of changes, except for changes necessary to eliminate an immediate hazard to study participants. When applicable, amendments will be submitted to the appropriate Health Authorities.
- The protocol and any applicable documentation will be submitted or notified to the Health Authorities in accordance with all local and national regulations for each site.

Scientific Rationale for Submission of the Summary of Clinical Study Results Beyond One Year from the End of the Clinical Trial in all EU Member States

- Since this study includes third countries, the clinical trial may still be ongoing in third countries at the timepoint when end of clinical trial is notified in all EU member states. Due to the study's sample size, it is considered justified to await completion in all countries and provide a summary of study results within 1 year from global end of trial, rather than providing summary results based on partial information.

- Thus, in such a case, the summary of study results will be provided within one year from notification in the EU portal of the global end of the clinical trial in all countries (including all EU Member States concerned and all third countries) in which the clinical trial has been conducted (Article 37 (4)).

Emergency Medical Support

- The Sponsor or designee will provide Emergency Medical Support cards to participants for use during the study. These provide the means for participants to identify themselves as participating in a clinical study. Also, these give health care providers access to any information about this participation that may be needed to determine the course of medical treatment for the participant. The information on the Emergency Medical Support card may include the process for emergency unblinding (if applicable).
- The first point of contact for all emergencies will be the clinical study Investigator caring for the participant. Consequently, the Investigator agrees to provide his or her emergency contact information on the card. If the Investigator is available when an event occurs, they will answer any questions. Any subsequent action (e.g., unblinding) will follow the standard process established for Investigators.

  When the Investigator is not available, the Sponsor provides the appropriate means to contact a Sponsor (or designee) physician. This includes provision of a 24-hour contact number at a call center, whereby the health care providers will be given access to the appropriate Sponsor (or designee) physician to assist with the medical emergency.

Clinical Study Insurance and Compensation to Participants

The Sponsor is entirely responsible for AEs that are associated with this study and cause damage to the health of the participants, except for AEs caused by an intentional and/or significant deviation on the part of the Investigator, the study site, and/or the participant. The Sponsor takes out insurance to fulfill the responsibility.

Insurance coverage will be provided for each country participating in the study. Insurance conditions will meet good local standards, as applicable.

Clinical Study Report

After study completion, the Sponsor will write a clinical study report in consultation with the Coordinating Investigator.

Publication

- The results of this study may be published or presented at scientific meetings. If this is foreseen, the Investigator agrees to submit all manuscripts or abstracts to the Sponsor before submission. This allows Merck to protect proprietary information and to provide comments.
- The Sponsor will comply with the requirements for publication of study results. Per standard editorial and ethical practice, the Sponsor will generally support publication of multicenter studies only in their entirety and not as individual site data.

- Authorship will be determined by agreement and in line with International Committee of Medical Journal Editors authorship requirements.

Dissemination of Clinical Study Data

- Any and all scientific, commercial, and technical information disclosed by the Sponsor in this protocol or elsewhere should be considered the confidential and proprietary property of the Sponsor. The Investigator shall hold such information in confidence and shall not disclose the information to any third party except to such of the Investigator's employees and staff who had been made aware that the information is confidential and who are bound to treat it as such and to whom disclosure is necessary to evaluate that information. The Investigator shall not use such information for any purpose other than for determining mutual interest in performing the study and, if the parties decide to proceed with the study, for the purpose of conducting the study.
- The Investigator understands that the information developed from this clinical study will be used by the Sponsor in connection with the development of the study intervention and therefore may be disclosed as required to other clinical Investigators, to the US Food and Drug Administration, and to other government agencies. The Investigator also understands that, to allow for the use of the information derived from the clinical study, the Investigator has the obligation to provide the Sponsor with complete test results and all data developed in the study.
- No publication or disclosure of study results will be permitted except under the terms and conditions of a separate written agreement.

Data Quality Assurance

- All participant study data will be recorded on printed or electronic CRFs or transmitted to the Sponsor or designee electronically (e.g., laboratory data). The Investigator is responsible for verifying that data entries are complete, accurate, legible, and timely by physically or electronically signing the CRF. Details for managing CRFs are in the Data Management Plan.
- The Investigator will maintain accurate documentation (source data) that supports the information in the CRF.
- The Investigator will permit study-related monitoring, quality assurance audits, IRB/IEC review, and regulatory agency inspections and provide direct access to the study file and source data.
- QTLs will be predefined and documented in the Trial Master File (TMF) and project management database to help support the identification of systematic issues that could potentially impact participant safety and/or reliability of study results. These predefined parameters will be monitored during the study and important deviations from the QTL thresholds and remedial actions taken will be summarized in the clinical study report.
- Monitoring details describing strategy (e.g., risk-based initiatives in operations and quality such as Risk Management and Mitigation Strategies and Analytical Risk-Based Monitoring), methods, responsibilities and requirements, including handling of noncompliance issues and monitoring techniques (central, remote, or on-site monitoring) are in the Monitoring Plan.

- The Sponsor or designee is responsible for data management of this study, including quality checking of the data and maintaining a validated database. Database lock will occur once quality control and quality assurance procedures have been completed. Details will be outlined in Data Management documents and procedures.
- Study Monitors will perform ongoing source data verification to confirm that data in the CRF are accurate, complete, and verifiable; that the safety and rights of participants are being protected; and that the study is being conducted per the currently approved protocol and any other study agreements, ICH GCP, the Japanese ministerial ordinance on GCP, and all applicable regulatory requirements.
- The Investigator will retain records and documents, including signed ICFs, pertaining to the conduct of this study for 15 years after study completion, unless local regulations, institutional policies, or the Sponsor requires a longer retention. No records may be destroyed during the retention period without the Sponsor’s written approval. No records may be transferred to another location or party without the Sponsor’s written notification.
- For studies with EU member states, the data will be collected and processed in accordance with Directive 95/46/EEC.

Source Documents

- Source documents provide evidence for the existence of the participant and substantiate the integrity of the data collected.
- The Investigator will maintain source documents that support the data recorded in the CRFs.
- Data recorded on CRFs that are transcribed from source documents will be consistent with the source documents or the discrepancies will be explained. The Investigator may need to request previous medical records or transfer records, depending on the study. Also, current medical records will be available.
- Source documents are stored at the site for the longest possible time permitted by the applicable regulations, and/or as per ICH GCP guidelines, whichever is longer. The Investigator and a record retainer designated by the Head of the study site ensures that no destruction of medical records is performed without the Sponsor’s written approval.
- Definition of what constitutes source data is found in the Monitoring Plan.

Study and Site Start and Closure

- The study start date is when the first participant signs the Informed Consent Form.
- The Investigator may initiate site closure at any time, provided there is reasonable cause and enough notice is given in advance of the intended closure.
- Reasons for the early closure of a study site by the Sponsor or Investigator may include:
  - Failure of the Investigator to comply with the protocol, the requirements of the IRB/IEC or local health authorities, the Sponsor's procedures, or GCP guidelines
  - Inadequate recruitment of participants by the Investigator

- Discontinuation of further development of the Sponsor's compound
- If the study is prematurely terminated or suspended, the Sponsor will promptly inform the Investigators, the IECs/IRBs, the regulatory authorities, and any third-party service providers of the reason for termination or suspension, as specified by the applicable regulatory requirements. The Investigator will promptly inform the participants and assure appropriate participant therapy and/or follow-up.

Appendix 3 Contraception and Barrier Requirements

Definitions

WOCBP:

A woman is of childbearing potential (fertile) following menarche and until becoming postmenopausal unless permanently sterile, as specified below.

If fertility is unclear (e.g., amenorrhea in adolescents or athletes) and a menstrual cycle cannot be confirmed before the first dose of study intervention, consider additional evaluation.

Postmenopause:

Postmenopause is defined as no menses for 12 months without an alternative medical cause.

- A high FSH level in the postmenopausal range may be used to confirm a postmenopausal state in a female not using hormonal contraception or HRT. However, in the absence of 12 months of amenorrhea, confirmation with more than 1 FSH measurement is required.
- A female on HRT and whose menopausal status are in doubt will be required to use one of the non-estrogen hormonal highly effective contraception methods if she wishes to continue her HRT during the study. Otherwise, she must discontinue HRT to allow confirmation of postmenopausal status before study enrollment.

Permanent sterilization:

For this study, permanent sterilization includes:

- Documented hysterectomy
- Documented bilateral salpingectomy
- Documented bilateral oophorectomy

Documentation can come from the site personnel's review of the individual's medical records, medical examination, or medical history interview.

For individuals with permanent infertility due to an alternate medical cause other than the above, (e.g., Mullerian agenesis, androgen insensitivity), Investigator discretion applies to determine study entry.

Contraception Guidance:

CONTRACEPTIVES ALLOWED DURING THE STUDY INCLUDE:
**Highly Effective Methods That Have Low User Dependency**<br>• Implantable progestogen-only hormone contraception associated with inhibition of ovulation<br>• IUD<br>• IUS<br>• Bilateral tubal occlusion

<table>
<tr><td>

- Azoospermic partner (vasectomized or due to a medical cause)

Azoospermia is a highly effective contraceptive method provided the partner is the sole sexual partner of the WOCBP and the absence of sperm has been confirmed. Otherwise, use an additional highly effective method of contraception. The spermatogenesis cycle is approximately 90 days.

Documentation can come from the site personnel's review of the individual's medical records, medical examination, or medical history interview.
</td></tr>
<tr><td>

Highly Effective Methods That Are User Dependent

Contraceptive measures which are considered highly effective comprise combined:

- estrogen and progestogen containing,
- hormonal contraception associated with inhibition of ovulation (oral, intravaginal, transdermal),
- progestogen-only hormonal contraception associated with inhibition of ovulation (oral, injectable, implantable),
- intrauterine device, intrauterine hormone-releasing system, bilateral tubal occlusion, vasectomized partner and sexual abstinence.
- sexual abstinence: a highly effective method only if defined as refraining from intercourse during the entire period of risk associated with the study intervention. The reliability of sexual abstinence needs to be evaluated in relation to the duration of the study and the preferred and usual lifestyle of the participant.

For Japan-specific contraceptive requirements, see Appendix 11.
</td></tr>
<tr><td>

Barrier Methods (to be used in addition to a highly effective method)

- Male or female condom with or without spermicide
- Cap, diaphragm, or sponge with spermicide

For Japan-specific barrier methods requirements, see Appendix 11.
</td></tr>
<tr><td>

Notes:

Contraceptive use by men or women is consistent with local regulations on the use of contraceptive methods for clinical study participants.

Highly effective methods are those with a failure rate of <1% per year when used consistently and correctly. Typical use failure rates differ from those when used consistently and correctly.

If locally required, in accordance with CTFG guidelines, acceptable contraceptive methods are limited to those which inhibit ovulation as the primary mode of action.

Periodic abstinence (calendar, symptothermal, post-ovulation methods), withdrawal (coitus interruptus), spermicides only, and LAM are **not** acceptable methods of contraception for this study. Male condom and female condom cannot be used together (due to risk of failure from friction).
</td></tr>
</table>

Appendix 4 Adverse Events: Definitions and Procedures for Recording, Evaluating, Follow-up, and Reporting

AE Definition

AE Definition

- An AE is any untoward medical occurrence in a patient or clinical study participant, temporally associated with the use of study intervention, whether considered related to the study intervention or not.
- An AE can therefore be any unfavorable and unintended sign (including an abnormal laboratory finding), symptom, or disease (new or exacerbated) temporally associated with the use of study intervention. For surgical or diagnostic procedures, the condition/illness leading to such a procedure is considered as the AE rather than the procedure itself.

Events Meeting the AE Definition

- Any abnormal laboratory test results (hematology, clinical chemistry, or urinalysis) or other safety assessments (e.g., ECG, radiological scans, vital signs measurements), including those that worsen from baseline and are judged to be more severe than expected for the participant's condition are considered clinically significant in the medical and scientific judgment of the Investigator (i.e., not related to progression of underlying disease, but may be leading to study intervention discontinuation).
- Exacerbation of a chronic or intermittent pre-existing condition including either an increase in frequency and/or intensity of the condition.
- New conditions detected or diagnosed after study intervention administration even though it may have been present before the start of the study.
- Signs, symptoms, or the clinical sequelae of a suspected drug-drug interaction.
- Signs, symptoms, or the clinical sequelae of a suspected overdose of either study intervention or a concomitant medication.
- "Lack of efficacy" or "failure of expected pharmacological action" per se will not be reported as an AE or a SAE. However, the signs, symptoms, and/or clinical sequelae resulting from lack of efficacy will be reported as an AE or a SAE if they fulfill the definition of an AE or SAE.

Events NOT Meeting the AE Definition

- Unless judged by the Investigator to be more severe than expected for the participant's condition, any clinically significant abnormal laboratory findings, other abnormal safety assessments that are associated with the underlying disease, the disease/disorder being studied within the expectedness for participant's condition, as judged by the Investigator.
- Medical or surgical procedure (e.g., endoscopy, appendectomy): the condition that leads to the procedure is the AE.

• Situations in which an untoward medical occurrence did not occur (social and/or convenience admission to a hospital).<br>• Anticipated day-to-day fluctuations of pre-existing disease(s) or condition(s) present or detected at the start of the study that do not worsen.

AE/SAEs Observed in Association with Disease Progression

Progression of the disease/disorder being studied assessed by measurement of lesions on radiographs or other methods as well as associated clinical signs or symptoms (including laboratory abnormalities) will not be reported as AEs/SAEs, unless the participant’s general condition is more severe than expected for the his/her condition and/or unless the outcome is fatal within the AE reporting period, as defined in Section 8.3.

Other Adverse Events to be Reported Using a Specialized Procedure or Form

Dose-limiting toxicities, pregnancies, overdoses, AESIs, or birth defects should follow the reporting procedures described in Section 6.7 (overdoses) and Section 8.3.

SAE Definition

If an event is not an AE per the definition above, then it cannot be an SAE even if serious conditions are met (e.g., hospitalization for signs/symptoms of the disease under study, death due to progression of disease).

**A SAE is defined as any untoward medical occurrence that, at any dose:**
a. Results in death
b. Is life-threatening<br>The term 'life-threatening' in the definition of 'serious' refers to an event in which the participant was at risk of death at the time of the event. It does not refer to an event, which hypothetically might have caused death, if it were more severe.
c. Requires inpatient hospitalization or prolongation of existing hospitalization<br>• In general, hospitalization signifies that the participant has been detained (usually involving at least an overnight stay) at the hospital or emergency ward for observation and/or treatment that would not have been appropriate in the physician’s office or outpatient setting. Complications that occur during hospitalization are AEs. If a complication prolongs hospitalization or fulfills any other serious criteria, the event is serious. When in doubt as to whether “hospitalization” occurred or was necessary, the AE will be considered serious.<br>• Hospitalization for elective treatment of a pre-existing condition that did not worsen from baseline is not considered an AE.<br>• However, all events leading to unplanned hospitalizations or unplanned prolongation of an elective hospitalization must be documented and reported as SAEs.

d. Results in persistent disability/incapacity<br>The term disability means a substantial disruption of a person's ability to conduct normal life functions.<br>This definition is **not** intended to include experiences of relatively minor medical significance such as uncomplicated headache, nausea, vomiting, diarrhea, influenza, and accidental trauma (e.g., sprained ankle) which may interfere with or prevent everyday life functions but do not constitute a substantial disruption.
e. Is a congenital anomaly/birth defect
f. Other situations:<br>• Medical or scientific judgment will be exercised in deciding whether SAE reporting is appropriate in other situations, such as important medical events that may not be immediately life-threatening or result in death or hospitalization but may jeopardize the participant or may require medical or surgical intervention to prevent one of the other outcomes listed in the above definition. These events are usually considered as serious.<br>• Examples of such events include invasive or malignant cancers, intensive treatment in an emergency room or at home for allergic bronchospasm, blood dyscrasias or convulsions that do not result in hospitalization, or development of drug dependency or drug abuse.

Any suspected transmission of an infectious agent via a study intervention is also considered an SAE for reporting purposes, as specified below for reporting SAEs.

Recording and Follow-Up of AE and/or SAE

AE and SAE Recording
• When an AE/SAE occurs, it is the responsibility of the Investigator to review all documentation (e.g., hospital progress notes, laboratory reports, and diagnostics reports) related to the event.<br>• The Investigator will then record all relevant AE/SAE information in the CRF.<br>• As needed, Sponsor/Designee may ask for copies of certain medical records (e.g., autopsy reports, supplemental lab reports, documents on medical history/concomitant medications, discharge letters), as supporting source documentation. All participant identifiers, except the participant number, will be redacted on these copies before submission to Sponsor/Designee.<br>• The Investigator will attempt to establish a diagnosis of the event based on signs, symptoms, and/or other clinical information. Whenever possible, the diagnosis (not the individual signs/symptoms) will be documented as the AE/SAE.<br>• If an AE constitutes a DLT this is documented accordingly.<br>• Specific guidance is in the CRF Completion and Monitoring Conventions.

**Assessment of Intensity**
The Investigator will assess the intensity of each AE and SAE reported during the study and assign it to 1 of the following categories:<br>• Mild: An event that is easily tolerated by the participant, causing minimal discomfort and not interfering with everyday activities.<br>• Moderate: An event that causes sufficient discomfort and interferes with normal everyday activities.<br>• Severe: An event that prevents normal everyday activities. Do not confuse an AE that is assessed as severe with a SAE. Severe is a category used to rate the intensity of an event; both AEs and SAEs can be assessed as severe.<br>An event is defined as “serious” when it meets at least 1 of the predefined criteria specified in the definition of an SAE, NOT when it is rated as severe.<br>Investigators will reference the NCI-CTCAE, version 5.0 (published on 27 November 2017), a descriptive terminology that can be used for AE reporting.<br>A general grading (severity/intensity; hereafter referred to as severity) scale is provided at the beginning of the above referenced document, and specific event grades are also provided.<br>If the severity for an AE is not specifically graded by NCI-CTCAE, the Investigator is to use the general NCI-CTCAE definitions of Grade 1 through Grade 5, using his or her best medical judgment.<br>The 5 general grades are:<br>• Grade 1 or Mild<br>• Grade 2 or Moderate<br>• Grade 3 or Severe<br>• Grade 4 or Life-threatening<br>• Grade 5 or Death<br>Any clinical AE with severity of Grade 4 or 5 must also be reported as an SAE. However, a laboratory abnormality of Grade 4, such as anemia or neutropenia, is considered serious only if the condition meets one of the serious criteria specified below.<br>If death occurs, the primary cause of death or event leading to death will be recorded and reported as an SAE. “Fatal” will be recorded as the outcome of this specific event and death will not be recorded as separate event. Only, if no cause of death can be reported (e.g., sudden death, unexplained death), the death per se might then be reported as an SAE.

Assessment of Causality
• The Investigator will assess the relationship between study intervention and each AE/SAE occurrence:<br>• Unrelated: Not reasonably related to the study intervention. AE could not medically (pharmacologically/clinically) be attributed to the study intervention. A reasonable alternative explanation will be available.<br>• Related: Reasonably related to the study intervention. AE could medically (pharmacologically/clinically) be attributed to the study intervention.<br>• A "reasonable possibility" of a relationship conveys that there are facts, evidence, and/or arguments to suggest a causal relationship, rather than a relationship cannot be ruled out.<br>• The Investigator will use clinical judgment to determine the relationship.<br>• Alternative causes, such as underlying disease(s), concomitant therapy, and other risk factors, as well as the temporal relationship of the event to study intervention administration will be considered and investigated.<br>• The Investigator will also consult the IB and/or Product Information, for marketed products, in his/her assessment.<br>• For each AE/SAE, the Investigator will document in the medical notes that he/she has reviewed the AE/SAE and assessed causality.<br>• There may be situations when an SAE has occurred, and the Investigator has minimal information to include in the initial report to the Sponsor or its designee. To meet the reporting timeline, the causality assessment is not required for the initial report.<br>• The Investigator may change his/her causality assessment after considering follow-up information and send a SAE follow-up report with the updated causality assessment.<br>• The causality assessment is one of the criteria used when determining regulatory reporting requirements.

**Follow-up of AEs and SAEs**
• The Investigator will perform or arrange for the conduct of supplemental measurements and/or evaluations, as medically indicated or as requested by the Sponsor or Designee to elucidate the nature and/or causality of the AE or SAE, as fully as possible. This may include additional laboratory tests or investigations, histopathological examinations, or consultation with other health care professionals.<br>• If a participant dies during participation in the study or during a recognized follow-up period, the Investigator will provide the Sponsor or Designee with a copy of any post-mortem findings including histopathology.<br>• New or updated information will be recorded in the originally completed CRF.<br>• The Investigator will submit any updated SAE data to the Sponsor or Designee within 24 hours of receipt of the information.

Reporting of SAEs

**SAE Reporting by an Electronic Data Collection Tool**
• The primary mechanism for reporting an SAE in multicenter studies to the Sponsor or its designee will be the electronic data collection tool.<br>• If the electronic system is unavailable, then the site will use the paper SAE form, specified below, to report the event within 24 hours.<br>• The site will enter into the electronic system the SAE data within 24 hours after becoming aware of the event. It is expected that the investigator/sub-investigator signs off this data in the system and any relevant associated data (e.g. additional laboratory tests, medical records, diagnostic reports, histopathological examinations, or consultation with other health care professionals) will be entered as soon as it becomes available.<br>• After the study is completed at a site, the electronic data collection tool will be taken off-line to prevent the entry of new data or changes to existing data.<br>• If a site receives a report of a new SAE from a study participant or receives updated data on a previously reported SAE after the electronic data collection tool has been taken off-line, then the site can report this information on a paper SAE form or to the Sponsor's safety department.<br>• By exception, an SAE (or follow-up information) may be reported by telephone. The site will complete the electronic SAE data entry immediately thereafter.
**SAE Reporting by a Paper Form**
• SAE reporting on a paper report form may be used in single center studies in addition to the standard electronic CRF and as a back-up method for an EDC system failure. The form includes completion instructions for the Investigator, names, addresses, and telephone and fax numbers. All information from the paper form will be transcribed into the electronic form as soon as the system becomes available.

- Facsimile transmission (fax to mail) of the paper form or any follow-up information is the preferred method for transmission and will be done within 24 hours to the Sponsor or its designee.
- In rare circumstances and in the absence of facsimile equipment, notification by telephone is acceptable with a copy of the form sent by overnight mail or courier service.
- Initial notification via telephone does not replace the need for the Investigator to complete and sign the form within 24 hours after becoming aware of the event.
- Additional documents (e.g., laboratory reports, autopsy report, hospital discharge letter) and relevant pages from the CRF may be required in addition (e.g. medical history, concomitant medication). The data provided will be consistent with the information in the CRF.

Recording and Reporting of DLTs

- Each event that meets the DLT criteria, as specified in Section 6.5.4, will be recorded in the CRF within 24 hours after awareness of the event.
- Serious DLTs will be reported in an expedited manner, using the SAE reporting process, as specified above.
- Notification of each DLT related event (non-serious and serious) will be reported to the Sponsor or its designee within 24 hours from the date of awareness.

Reporting of AESIs

For a nonserious AESI, the site will complete the specific AESI report form and notify the Sponsor immediately (within 24 hours). For a serious AESI, the site will complete an SAE report form. For both AESI types, the site will use the same process as stated in the above section entitled "Reporting of SAEs".

Reporting of Pregnancies

- Pregnancy will be reported whether or not related to the study intervention using the applicable paper form.
- The applicable form will be used to report if an abnormal outcome of the pregnancy occurs and the child/fetus sustains an event.
- Facsimile transmission (fax to mail) of the paper form or any follow-up information is the preferred method for transmission and will be done within 24 hours to the Sponsor or its designee.

Appendix 5 Liver Safety: Suggested Actions and Follow-up Assessments

If a study participant is noted to have ALT or AST elevated 3 times or greater above ULN (new or worsening of preexisting), the abnormality should be recorded as an AE, regardless if clinical symptoms are present or not. If a study participant is noted to have ALT or AST 3 times or greater above ULN and total bilirubin ≥ 2 ULN for which an alternative etiology has not been identified, the event should be reported as a SAE. The Investigator must contact the Sponsor Medical Responsible for discussion.

Appendix 6 Clinical Laboratory Tests

The protocol-required clinical laboratory assessments are in the following table:

Laboratory Assessments[1]	Parameters			
Hematology	Platelet count<br>Mean platelet volume (MPV)		Erythrocytes (RBC)<br>Reticulocytes count[5]<br>Mean corpuscular volume (MCV)	<u>White Blood Cell Count with Differential</u>:<br>• Neutrophils<br>• Lymphocytes<br>• Monocytes<br>• Eosinophils<br>• Basophils
	Hemoglobin<br>Ferritin[6]<br>Transferrin saturation[6]		Mean corpuscular hemoglobin (MCH)<br>Erythrocytes Distribution Width (RDW)	
	Hematocrit			
Biochemistry[2, 3]	Blood Urea Nitrogen or Urea	Potassium	Aspartate aminotransferase	Bilirubin<br>Direct or Indirect bilirubin
	Creatinine<br>Creatinine clearance	Sodium	Alanine aminotransferase	Protein (total)
	Glucose	Calcium<br>Calcium corrected	Alkaline phosphatase	Albumin
	Amylase	Lipase	Gamma Glutamyl Transferase	Lactate dehydrogenase
Coagulation[4]	Activated Partial Thromboplastin Time	Prothrombin Time	Prothrombin Intl. Normalized Ratio	

Notes:

[1] Part 1 only: On C2D15 only CBC needs to be performed and blood draw may be performed at patient's local physician's practice. Results need to be reported to the Principal Investigator for review and entered in the eCRF.

[2] Details of liver chemistry stopping criteria and required actions and follow-up assessments after liver stopping or monitoring event are given in Section 7.1 and Appendix 5.

[3] For Japan-specific assessments related to ILD risk management (i.e., KL-6), see Appendix 11.

[4] Part 2 only: Coagulation required only on D1 of each cycle, EOT and Safety Follow-Up Visit.

[5] Part 2 only: Reticulocyte count for Part 2A and 2C only needed on D15 and for Part 2B and 2D only needed on Day 8.

[6] In Part 1 at Screening only. Not required for Part 2D.

Laboratory Assessments	Parameters
Routine Urinalysis	• pH, glucose, protein, blood, ketones, bilirubin, urobilinogen, nitrite, leukocytes, specific gravity by dipstick<br>• Microscopic examination (if blood or protein is abnormal, if applicable based on the locally institutional guidelines)<br>• **Part 2C and 2D**: If dipstick proteinuria ≥ 2+, a 24-hour urine collection is required, and quantitative assessment of proteinuria should be performed.
Other Screening Tests	• Serum (at Screening) or highly sensitive urine hCG pregnancy test (as needed for a WOCBP). Note: urine testing at a local laboratory will be standard for the protocol. If required by local regulations or the IRB/IEC, serum testing will be done instead.<br>• HBsAg, HBcAb<br>• HBV DNA (quantitative PCR)<br>• HCVAb, HCV RNA (quantitative PCR)<br>• HIV Antibody tests (optional unless locally required)

	• [redacted]<br>All study-required laboratory assessments will be performed by local laboratory. It is required that these local laboratories are certified, perform and document interlaboratory testing at regular time intervals and provide a list of normal range laboratory values including units as defined by international system of units.

Appendix 7 List of Strong and Moderate CYP Inhibitors and Inducers

	Inhibitors	Strong inducers
**CYP3A4**	Examples of strong inhibitors include<br>boceprevir,<br>cobicistat,<br>danoprevir and ritonavir*,<br>elvitegravir and ritonavir*,<br>indinavir and ritonavir*,<br>itraconazole,<br>ketoconazole,<br>lopinavir and ritonavir*,<br>mibefradil (withdrawn in US),<br>paritaprevir and ritonavir and (ombitasvir and/or dasabuvir)*,<br>posaconazole,<br>ritonavir,<br>saquinavir and ritonavir*,<br>telaprevir,<br>tipranavir and ritonavir*,<br>telithromycin,<br>troleandomycin,<br>voriconazole,<br>idelalisib,<br>nefazodone,<br>nelfinavir,<br>clarithromycin<br><br>Examples of moderate inhibitors include<br>aprepitant,<br>atazanavir*,<br>ciprofloxacin,<br>darunavir*,<br>diltiazem,<br>dronedarone,<br>erythromycin,<br>fluconazole,<br>isavuconazole,<br>netupitant,<br>tofizopam,<br>verapamil	apalutamide,<br>carbamazepine,<br>enzalutamide,<br>mitotane,<br>phenytoin,<br>rifampin,<br>St. John’s wort
**CYP1A2**	Examples of strong inhibitors<br>fluvoxamine,<br>ciprofloxacin,<br>enoxacin,	

* Ritonavir is usually given in combination with other anti-HIV or anti-HCV drugs in clinical practice. Caution should be used when extrapolating the observed effect of ritonavir alone to the effect of combination regimens on CYP3A activities.

Refer to website for details: https://www.fda.gov/drugs/drug-interactions-labeling/drug-development-and-drug-interactions-table-substrates-inhibitors-and-inducers

Appendix 8 Model for Bayesian Dose Escalation

The Bayesian model results are based on the number of DLTs and evaluable participants per dose level. The SMC will receive results of a Bayesian two-parameter logistic regression model updated with the observed DLT data (Neuenschwander 2008), including a recommendation for the next dose. For a dose level d_j, the relationship between dose and probability of toxicity P (DLT) is defined by:

$$P\left(DLT \middle| d_j, \alpha, \beta\right) = \frac{\exp\left(\alpha + \exp(\beta) * \log\left(\frac{d_j}{d_{ref}}\right)\right)}{1 + \exp\left(\alpha + \exp(\beta) * \log\left(\frac{d_j}{d_{ref}}\right)\right)},$$

with bivariate normally distributed parameters (α, β), using the following parameterization:

- [redacted]
- [redacted]
- [redacted]
- [redacted]
- [redacted]

The following toxicity regions will be defined:

	Probability of DLT	Loss term (weight in loss function)
**Under-Dosing**	0.0, 0.20	1
**Target toxicity**	0.20, 0.35	0
**Excessive toxicity**	0.35, 0.60	1
**Unacceptable toxicity**	0.60, 1.00	2

The model-based recommendation for the next dose level is the dose level that minimizes the loss function. The loss function is defined as the sum of products of the probability to lie within each of the toxicity regions, and the associated loss term:

- 1 × P (Under-Dosing) + 0 × P (targeted toxicity) + 1 × P (excessive toxicity) + 2 × P (unacceptable toxicity).

The model will be provided with the pre-selected dose levels and/or other potential dose levels (e.g., at SMC request). The SMC can recommend changes to the set of doses any time.

The target DLT probability for the MTD for each part suggested by the Bayesian model is 30%.

The SMC will be notified of a potential MTD for each part once the estimate for DLT probability of a potential MTD reaches sufficient precision, i.e.:

- The upper bound of the one-sided 90% credible interval is not more than 40% and the median estimated DLT probability for the suggested MTD is in 17%-30%.

or

- At least 6 participants have been treated at the suggested MTD and the model recommends the suggested MTD as the next dose level and the upper bound of the one-sided 95% credible interval is not more than 60%.

In case information arises from other studies that changes current knowledge on the dose-toxicity relationship, the prior distribution will be updated prior to the first participant being treated in this study. This change will be documented in the SMC charter.

Posterior distribution and the recommended next dose level suggested by the model will be calculated using SAS v 9.1 or higher, or R version 3.6 or higher with library package bcrm (Sweeting 2013) or package CRMpack (Bové 2019).

Extension for additional regimens (Part 2B)

The above-described Bayesian 2-parameter logistic regression model can be extended to include one binary covariate corresponding to whether the participant receives a new regimen (=1), e.g., [redacted], or the Q3W regimen (=0).

E.g., for a [redacted] regimen and dose level dj, the relationship between dose and probability of toxicity P (DLT) is defined by:

$$P\left(DLT \middle| d_j, \alpha, \beta\right) = \frac{\exp\left(\alpha + \exp(\beta) * \log\left(\frac{d_j}{d_{ref}}\right) + \gamma * \mathrm{I}[\text{regimen}=\text{[redacted]}]\right)}{1 + \exp\left(\alpha + \exp(\beta) * \log\left(\frac{d_j}{d_{ref}}\right) + \gamma * \mathrm{I}[\text{regimen}=\text{[redacted]}]\right)},$$

with bivariate normally distributed parameters (α, β) as defined above and I is the indicator function corresponding to whether the [redacted] regimen is administered, and γ is normally distributed.

Note that if more than one regimen will be explored, the proposed model may be extended by additional covariates (one per regimen). The parametrization of γ and of potential additional covariates will be specified in the IAP or SMC Charter prior to dosing of the first participant in the new regimen.

Continual Reassessment Method (CRM) (Part 2C2 and Part 2D)

The model results for the CRM are based on the number of DLTs and evaluable participants per dose level. The SMC will receive results of a CRM model updated with the observed DLT data (O'Quigley 1990), including a recommendation for the next dose. For a combination dose level

D_i, the relationship between dose and probability of toxicity P (DLT) is defined by a one-parameter function which operates on a discrete set of increasing doses:

$$P(DLT|\pi_{ir},\beta) = {\pi_{ir}}^{\beta} \quad ,$$

where π_{ir} is the standardized dose level i under the ordering r (as defined in Section 9.4.2.2) and the parameter β follows the prior distribution

$$\beta \sim N(\hat{\beta}_0, \sigma_{\beta}^2),$$

where $\hat{\beta}_0$ and σ_{β}^2 are the prior mean and prior variance, respectively. The parametrization will be based on all available data from Part 1 and Part 2A and will be specified in the IAP or the SMC charter prior to dosing of the first participant in Part 2C.

The dose recommendation with overdose control (EWOC) follows the methodology by Babb 1998, where doses with a probability of > 25% that P(DLT) is ≥ 33% will not be considered for dose recommendation for the next cohort.

Appendix 9 Response Evaluation Criteria in Solid Tumors (RECIST) Version 1.1

The text below was obtained from Eisenhauer 2009.

Definitions

Response and progression will be evaluated in this trial using the international criteria proposed by the RECIST Committee (Version 1.1). Changes in only the largest diameter (unidimensional measurement) of the tumor lesions are used in the RECIST criteria, except lymph nodes, as detailed below. Note: Lesions are either measurable or non-measurable using the criteria provided below. The term "evaluable" in reference to measurability will not be used because it does not provide additional meaning or accuracy.

<u>Measurable Disease</u>

Tumor lesions: Must be accurately measured in at least 1 dimension (longest diameter in the plane of measurement is to be recorded) with a minimum size of:

- 10 mm by CT scan (irrespective of scanner type) and MRI (no less than double the slice thickness and a minimum of 10 mm)
- 10 mm caliper measurement by clinical exam (when superficial)
- 20 mm by chest X-ray (if clearly defined and surrounded by aerated lung).

Malignant lymph nodes: To be considered pathologically enlarged and measurable, a lymph node must be ≥ 15 mm in short axis when assessed by CT scan (CT scan slice thickness recommended to be no greater than 5 mm). At baseline and in follow-up, only the short axis will be measured and followed.

<u>Non-measurable Disease</u>

All other lesions (or sites of disease), including small lesions (longest diameter ≥ 10 to < 15 mm with conventional techniques or < 10 mm using spiral CT scan), are considered non-measurable disease. Leptomeningeal disease, ascites, pleural or pericardial effusion, inflammatory breast disease, lymphangitic involvement of skin or lung, abdominal masses/abdominal organomegaly identified by physical exam that is not measurable by reproducible imaging techniques are all non-measurable.

Bone lesions:

- Bone scan, PET scan, or plain films are not considered adequate imaging techniques to measure bone lesions. However, these techniques can be used to confirm the presence or disappearance of bone lesions

- Lytic bone lesions or mixed lytic-blastic lesions, with identifiable soft tissue components, that can be evaluated by cross-sectional imaging techniques such as CT or MRI can be considered as measurable lesions if the soft tissue component meets the definition of measurability described above

- Blastic bone lesions are non-measurable.

Cystic lesions:

- Lesions that meet the criteria for radiographically defined simple cysts should not be considered as malignant lesions (neither measurable nor non-measurable) since they are, by definition, simple cysts

- Cystic lesions thought to represent cystic metastases can be considered as measurable lesions, if they meet the definition of measurability described above. However, if non-cystic lesions are present in the same patient, these are preferred for selection as target lesions.

Lesions with prior local treatment:

- Tumor lesions situated in a previously irradiated area, or in an area subjected to other local regional therapy, are usually not considered measurable unless there has been demonstrated progression in the lesion. Trial protocols should detail the conditions under which such lesions would be considered measurable.

Target Lesions

All measurable lesions up to a maximum of 2 lesions per organ and 5 lesions in total, should be identified as **target lesions** and recorded and measured at baseline. Target lesions should be selected on the basis of their size (lesions with the longest diameter), be representative of all involved organs, but in addition should be those that lend themselves to reproducible repeated measurements.

Lymph nodes merit special mention since they are normal anatomical structures which may be visible by imaging even if not involved by tumor. Pathological nodes which are defined as measurable and may be identified as target lesions must meet the criterion of a short axis of ≥ 15 mm by CT scan. Only the short axis of these nodes will contribute to the baseline sum. The short axis of the node is the diameter normally used by radiologists to judge if a node is involved by solid tumor. Nodal size is normally reported as 2 dimensions in the plane in which the image is obtained (for CT scan this is almost always the axial plane; for MRI the plane of acquisition may be axial, sagittal, or coronal). The smaller of these measures is the short axis. For example, an abdominal node which is reported as being 20 mm × 30 mm has a short axis of 20 mm and qualifies as a malignant, measurable node. In this example, 20 mm should be recorded as the node measurement. All other pathological nodes (those with short axis ≥ 10 mm but < 15 mm) should be considered non-target lesions. Nodes that have a short axis < 10 mm are considered non-pathological and should not be recorded or followed.

A sum of the diameters (longest for non-nodal lesions, short axis for nodal lesions) for all target lesions will be calculated and reported as the baseline sum diameters. If lymph nodes are to be included in the sum, then as noted above, only the short axis is added into the sum. The baseline sum diameters will be used as reference to further characterize any objective tumor regression in the measurable dimension of the disease.

Non-target Lesions

All other lesions (or sites of disease) including pathological lymph nodes should be identified as non-target lesions and should also be recorded at baseline. Measurements are not required and these lesions should be followed as 'present', 'absent', or in rare cases 'unequivocal progression' (more details to follow). In addition, it is possible to record multiple non-target lesions involving the same organ as a single item on the case record form (e.g., 'multiple enlarged pelvic lymph nodes' or 'multiple liver metastases').

GUIDELINES FOR EVALUATION OF MEASURABLE DISEASE

All measurements should be recorded in metric notation, using calipers if clinically assessed. All baseline evaluations should be performed as close as possible to the treatment start and never more than 4 weeks before the beginning of the treatment.

The same method of assessment and the same technique should be used to characterize each identified and reported lesion at baseline and during follow-up. Imaging based evaluation should always be done rather than clinical examination unless the lesion(s) being followed cannot be imaged but are assessable by clinical exam.

Clinical lesions: Clinical lesions will only be considered measurable when they are superficial and ≥ 10 mm diameter as assessed using calipers (e.g., skin nodules). For the case of skin lesions, documentation by color photography including a ruler to estimate the size of the lesion is suggested. As noted above, when lesions can be evaluated by both clinical exam and imaging, imaging evaluation should be undertaken since it is more objective and may also be reviewed at the end of the trial.

Chest X-ray: Chest CT is preferred over chest X-ray, particularly when progression is an important endpoint, since CT is more sensitive than X-ray, particularly in identifying new lesions. However, lesions on chest X-ray may be considered measurable if they are clearly defined and surrounded by aerated lung.

CT, MRI: CT is the best currently available and reproducible method to measure lesions selected for response assessment. This guideline has defined measurability of lesions on CT scan based on the assumption that CT slice thickness is 5 mm or less. As is described in Appendix II of the original source article cited above, when CT scans have slice thickness greater than 5 mm, the minimum size for a measurable lesion should be twice the slice thickness. MRI is also acceptable in certain situations (e.g., for body scans).

Ultrasound: Ultrasound is not useful in assessment of lesion size and should not be used as a method of measurement. Ultrasound examinations cannot be reproduced in their entirety for

independent review at a later date and, because they are operator dependent, it cannot be guaranteed that the same technique and measurements will be taken from 1 assessment to the next. If new lesions are identified by ultrasound in the course of the trial, confirmation by CT or MRI is advised. If there is concern about radiation exposure at CT, MRI may be used instead of CT in selected instances.

Endoscopy, laparoscopy: The utilization of these techniques for objective tumor evaluation is not advised. However, they can be useful to confirm complete pathological response when biopsies are obtained or to determine relapse in trials where recurrence following complete response or surgical resection is an endpoint.

Tumor markers: Tumor markers alone cannot be used to assess objective tumor response. If markers are initially above the upper normal limit, however, they must normalize for a patient to be considered in complete response. Because tumor markers are disease specific, instructions for their measurement should be incorporated into protocols on a disease specific basis. Specific guidelines for both CA-125 response (in recurrent ovarian cancer) and prostate-specific antigen response (in recurrent prostate cancer), have been published. In addition, the Gynecologic Cancer Intergroup has developed CA-125 progression criteria which are to be integrated with objective tumor assessment for use in first-line trials in ovarian cancer.

Cytology, histology: These techniques can be used to differentiate between PR and CR in rare cases if required by protocol (for example, residual lesions in tumor types such as germ cell tumors, where known residual benign tumors can remain). When effusions are known to be a potential adverse event (AE) of treatment, the cytological confirmation of the neoplastic origin of any effusion that appears or worsens during treatment can be considered if the measurable tumor has met criteria for response or stable disease in order to differentiate between response (or stable disease) and progressive disease.

RESPONSE CRITERIA

Evaluation of Target Lesions

Complete Response (CR): Disappearance of all target lesions. Any pathological lymph nodes (whether target or non-target) must have reduction in short axis to < 10 mm.

Partial Response (PR): At least a 30% decrease in the sum of diameters of target lesions, taking as reference the baseline sum diameters.

Progressive Disease (PD): At least a 20% increase in the sum of diameters of target lesions, taking as reference the smallest sum on trial (this includes the baseline sum if that is the smallest on trial). In addition to the relative increase of 20%, the sum must also demonstrate an absolute increase of at least 5 mm. (Note: the appearance of 1 or more new lesions is also considered progression).

Stable Disease (SD): Neither sufficient shrinkage to qualify for PR nor sufficient increase to qualify for PD, taking as reference the smallest sum diameters while on trial.

Lymph nodes. Lymph nodes identified as target lesions should always have the actual short axis measurement recorded (measured in the same anatomical plane as the baseline examination), even if the nodes regress to below 10 mm on trial. This means that when lymph nodes are included as target lesions, the 'sum' of lesions may not be zero even if complete response criteria are met, since a normal lymph node is defined as having a short axis of < 10 mm. Case report forms or other data collection methods may therefore be designed to have target nodal lesions recorded in a separate section where, in order to qualify for CR, each node must achieve a short axis < 10 mm. For PR, SD, and PD, the actual short axis measurement of the nodes is to be included in the sum of target lesions.

Target lesions that become 'too small to measure'. While on trial, all lesions (nodal and non-nodal) recorded at baseline should have their actual measurements recorded at each subsequent evaluation, even when very small (e.g., 2 mm). However, sometimes lesions or lymph nodes which are recorded as target lesions at baseline become so faint on CT scan that the radiologist may not feel comfortable assigning an exact measure and may report them as being 'too small to measure'. When this occurs it is important that a value be recorded on the eCRF. If it is the opinion of the radiologist that the lesion has likely disappeared, the measurement should be recorded as 0 mm. If the lesion is believed to be present and is faintly seen but too small to measure, a default value of 5 mm should be assigned. (Note: It is less likely that this rule will be used for lymph nodes since they usually have a definable size when normal and are frequently surrounded by fat, such as in the retroperitoneum; however, if a lymph node is believed to be present and is faintly seen but too small to measure, a default value of 5 mm should be assigned in this circumstance as well). This default value is derived from the 5 mm CT slice thickness (but should not be changed with varying CT slice thickness). The measurement of these lesions is potentially non-reproducible; therefore, providing this default value will prevent false responses or progressions based upon measurement error. To reiterate, however, if the radiologist is able to provide an actual measure, that should be recorded, even if it is below 5 mm.

Lesions that split or coalesce on treatment. When non-nodal lesions 'fragment', the longest diameters of the fragmented portions should be added together to calculate the target lesion sum. Similarly, as lesions coalesce, a plane between them may be maintained that would aid in obtaining maximal diameter measurements of each individual lesion. If the lesions have truly coalesced such that they are no longer separable, the vector of the longest diameter in this instance should be the maximal longest diameter for the 'coalesced lesion'.

<u>Evaluation of Non-target Lesions</u>

While some non-target lesions may actually be measurable, they need not be measured and instead should be assessed only qualitatively at the time points specified in the protocol.

Complete Response (CR): Disappearance of all non-target lesions and normalization of tumor marker level. All lymph nodes must be non-pathological in size (< 10 mm short axis).

Non-CR/Non-PD: Persistence of one or more non-target lesion(s) and/or maintenance of tumor marker level above the normal limits.

Progressive Disease (PD): Unequivocal progression (see comments below) of existing non-target lesions. (Note: the appearance of one or more new lesions is also considered progression).

When the patient also has measurable disease. In this setting, to achieve ‘unequivocal progression’ on the basis of the non-target disease, there must be an overall level of substantial worsening in non-target disease such that, even in the presence of SD or PR in target disease, the overall tumor burden has increased sufficiently to merit discontinuation of therapy. A modest ‘increase’ in the size of 1 or more non-target lesions is usually not sufficient to quality for unequivocal progression status. The designation of overall progression solely on the basis of change in non-target disease in the face of SD or PR of target disease will therefore be extremely rare.

When the patient has only non-measurable disease. This circumstance arises in some Phase III trials when it is not a criterion of trial entry to have measurable disease. The same general concept applies here as noted above, however, in this instance there is no measurable disease assessment to factor into the interpretation of an increase in non-measurable disease burden. Because worsening in non-target disease cannot be easily quantified (by definition: if all lesions are truly non-measurable), a useful test that can be applied when assessing patients for unequivocal progression is to consider if the increase in overall disease burden based on the change in non-measurable disease is comparable in magnitude to the increase that would be required to declare PD for measurable disease: i.e., an increase in tumor burden representing an additional 73% increase in ‘volume’ (which is equivalent to a 20% increase diameter in a measurable lesion). Examples include an increase in a pleural effusion from ‘trace’ to ‘large’, an increase in lymphangitic disease from localized to widespread, or may be described in protocols as ‘sufficient to require a change in therapy’. If ‘unequivocal progression’ is seen, the patient should be considered to have had overall PD at that point. While it would be ideal to have objective criteria to apply to non-measurable disease, the very nature of that disease makes it impossible to do so; therefore, the increase must be substantial.

New Lesions

The appearance of new malignant lesions denotes disease progression; therefore, some comments on detection of new lesions are important. There are no specific criteria for the identification of new radiographic lesions; however, the finding of a new lesion should be unequivocal: i.e., not attributable to differences in scanning technique, change in imaging modality, or findings thought to represent something other than tumor (for example, some ‘new’ bone lesions may be simply healing or flare of pre-existing lesions). This is particularly important when the patient’s baseline lesions show partial or complete response. For example, necrosis of a liver lesion may be reported on a CT scan report as a ‘new’ cystic lesion, which it is not.

A lesion identified on a follow-up trial in an anatomical location that was not scanned at baseline is considered a new lesion and will indicate disease progression. An example of this is the patient who has visceral disease at baseline and while on trial has a brain CT or MRI ordered which reveals metastases. The patient’s brain metastases are considered to be evidence of PD even if he/she did not have brain imaging at baseline.

If a new lesion is equivocal, for example because of its small size, continued therapy and follow-up evaluation will clarify if it represents truly new disease. If repeat scans confirm there is definitely a new lesion, then progression should be declared using the date of the initial scan.

While fludeoxyglucose positron emission tomography (FDG-PET) response assessments need additional trial, it is sometimes reasonable to incorporate the use of FDG-PET scanning to complement CT scanning in assessment of progression (particularly possible 'new' disease). New lesions on the basis of FDG-PET imaging can be identified according to the following algorithm:

a. Negative FDG-PET at baseline, with a positive FDG-PET at follow-up is a sign of PD based on a new lesion.

b. No FDG-PET at baseline and a positive FDG-PET at follow-up: If the positive FDG-PET at follow-up corresponds to a new site of disease confirmed by CT, this is PD. If the positive FDG-PET at follow-up is not confirmed as a new site of disease on CT, additional follow-up CT scans are needed to determine if there is truly progression occurring at that site (if so, the date of PD will be the date of the initial abnormal FDG-PET scan). If the positive FDG-PET at follow-up corresponds to a pre-existing site of disease on CT that is not progressing on the basis of the anatomic images, this is not PD.

<u>Evaluation of Best Overall Response</u>

The best overall response is the best response recorded from the start of the trial treatment until the end of treatment taking into account any requirement for confirmation. On occasion, a response may not be documented until after the end of therapy, so protocols should be clear if post treatment assessments are to be considered in determination of best overall response. Protocols must specify how any new therapy introduced before progression will affect best response designation. The patient's best overall response assignment will depend on the findings of both target and non-target disease and will also take into consideration the appearance of new lesions. Furthermore, depending on the nature of the trial and the protocol requirements, it may also require confirmatory measurement. Specifically, in non-randomized trials where response is the primary endpoint, confirmation of PR or CR is needed to deem either one the 'best overall response'.

The best overall response is determined once all the data for the patient is known. Best response determination in trials where confirmation of complete or partial response IS NOT required: Best response in these trials is defined as the best response across all time points (for example, a patient who has SD at first assessment, PR at second assessment, and PD on last assessment has a best overall response of PR). When SD is believed to be best response, it must also meet the protocol specified minimum time from baseline. If the minimum time is not met when SD is otherwise the best timepoint response, the patient's best response depends on the subsequent assessments. For example, a patient who has SD at first assessment, PD at second and does not meet minimum duration for SD, will have a best response of PD. The same patient lost to follow-up after the first SD assessment would be considered inevaluable.

Target Lesions	Non-target Lesions	New Lesions	Overall Response
CR*	CR	No	CR
CR	Non-CR/non-PD	No	PR
CR	Not Evaluated	No	PR
PR	Non-PD or not all evaluated	No	PR
SD	Non-PD or not all evaluated	No	SD
Not all evaluated	Non-PD	No	NE
PD	Any	Yes or No	PD
Any	PD	Yes or No	PD
Any	Any	Yes	PD

CR: complete response; PR: partial response; SD: stable disease; PD: progressive disease; NE: inevaluable.
See text for more details.

Note:

When nodal disease is included in the sum of target lesions and the nodes decrease to ‘normal’ size (< 10 mm), they may still have a measurement reported on scans. This measurement should be recorded even though the nodes are normal in order not to overstate progression should it be based on increase in size of the nodes. As noted earlier, this means that patients with CR may not have a total sum of ‘zero’ on the eCRF.

In trials where confirmation of response is required, repeated ‘NE’ timepoint assessments may complicate best response determination. The analysis plan for the trial must address how missing data/assessments will be addressed in determination of response and progression. For example, in most trials, it is reasonable to consider a patient with timepoint responses of PR-NE-PR as a confirmed response.

Patients with a global deterioration of health status requiring discontinuation of treatment without objective evidence of disease progression at that time should be reported as ‘symptomatic deterioration’. Every effort should be made to document objective progression even after discontinuation of treatment. Symptomatic deterioration is not a descriptor of an objective response; it is a reason for stopping trial therapy.

Conditions that define ‘early progression, early death, and inevaluability’ are trial-specific and should be clearly described in each protocol (depending on treatment duration, treatment periodicity).

In some circumstances it may be difficult to distinguish residual disease from normal tissue. When the evaluation of complete response depends upon this determination, it is recommended that the residual lesion be investigated (fine needle aspirate/biopsy) before assigning a status of complete response. The use of FDG-PET may be used to upgrade a response to a CR in a manner similar to a biopsy in cases where a residual radiographic abnormality is thought to represent fibrosis or scarring. The use of FDG-PET in this circumstance should be prospectively described in the protocol and supported by disease specific medical literature for the indication. However, it must be acknowledged that both approaches may lead to false positive CR due to limitations of FDG-PET and biopsy resolution/sensitivity.

For equivocal findings of progression (e.g., very small and uncertain new lesions; cystic changes or necrosis in existing lesions), treatment may continue until the next scheduled assessment. If at the next scheduled assessment, progression is confirmed, the date of progression should be the earlier date when progression was suspected.

CONFIRMATORY MEASUREMENT/DURATION OF RESPONSE

Confirmation

In non-randomized trials where response is the primary endpoint, confirmation of PR and CR is required to ensure the responses identified are not the result of measurement error. This will also permit appropriate interpretation of results in the context of historical data where response has traditionally required confirmation in such trials. However, in all other circumstances, i.e., in randomized trials (Phase II or III) or studies where stable disease or progression are the primary endpoints, confirmation of response is not required since it will not add value to the interpretation of the trial results. However, elimination of the requirement for response confirmation may increase the importance of central review to protect against bias, in particular in studies which are not blinded.

In the case of SD, measurements must have met the SD criteria at least once after trial entry at a minimum interval (in general not less than 6 to 8 weeks) that is defined in the trial protocol.

Duration of Overall Response

The duration of overall response is measured from the time measurement criteria are first met for CR/PR (whichever is first recorded) until the first date that recurrent or PD is objectively documented (taking as reference for PD the smallest measurements recorded on trial).

The duration of overall complete response is measured from the time measurement criteria are first met for CR until the first date that recurrent disease is objectively documented.

Duration of Stable Disease

Stable disease is measured from the start of the treatment (in randomized trials, from date of randomization) until the criteria for progression are met, taking as reference the smallest sum on trial (if the baseline sum is the smallest, this is the reference for calculation of PD).

The clinical relevance of the duration of stable disease varies in different studies and diseases. If the proportion of patients achieving stable disease for a minimum period of time is an endpoint of importance in a particular trial, the protocol should specify the minimal time interval required between 2 measurements for determination of stable disease.

Note: The duration of response and stable disease as well as the progression-free survival are influenced by the frequency of follow-up after baseline evaluation. It is not in the scope of this guideline to define a standard follow-up frequency. The frequency should take into account many parameters including disease types and stages, treatment periodicity, and standard practice.

However, these limitations of the precision of the measured endpoint should be taken into account if comparisons between trials are to be made.

Appendix 10 Protocol Amendment History

The information for the current amendment is on the title page.

Protocol Version 3.3 EU (18 July 2024)

Overall Rationale for the Amendment

The protocol is amended in response to feedback from the European National Health Authorities.

Section # and Name	Description of Change	Brief Rationale
4.1 Overall Design (Part 2B)	Text added to describe that for Part 2B, the Sponsor upon recommendation by the SMC and using emerging data from Part 2A may evaluate other doses "as long as they do not exceed the time-averaged dose intensity corresponding to the MTD defined with Q3W regimen."	To clarify for Part 2B the highest dose tested cannot exceed the time-averaged dose intensity corresponding to the MTD in the Q3W regimen.
4.1 Overall Design (Part 2C2)	i. Paragraph revision on sentinel dosing in Parts 2C1 and 2C2<br>ii. Clarification that enrollment in Part 2C2 can only start after dose level 1 in Part 2C1 has been deemed safe and tolerable by the SMC.	iii. Implementation of sentinel dosing verbiage between corresponding dose levels of different parts (2C1 and 2C2) in Part 2C<br>iv. To clarify sequence of enrollment in Part 2C.<br>.
4.2.3 Rationale for Treatment of CRC with anti CEACAM5 ADC	Text added to describe the all-comers approach without the need of participant selection given the high prevalence of CEACAM5 expression in mCRC.	To justify that participants can be included in the study without prospectively testing for CEACAM5 expression.
4.3.2 Parts 2A, 2B, and 2C – Dose Expansion	Text added to provide additional information regarding the preliminary clinical data from the dose escalation part of the study to include occurrence of a Grade 5 event.	Requested by HA to provide additional information in that part.
5 Study Population<br>7.1 Discontinuation of Study Intervention<br>Appendix 2	ICF signing by legal representative removed.	Participant must be able to sign the ICF.
5.2 Exclusion Criteria	Exclusion criteria added for Parts 2C1 and 2C2	To conform with the use of bevacizumab and capecitabine in these study parts.

Section # and Name	Description of Change	Brief Rationale
6.1 Study Intervention Administration (Table 15 and Table 16)<br><br>Appendix 11 Country-specific Requirements	v. Clarified that each vial will be labeled per country-specific requirements.<br>vi. Changed Appendix 11 title to 'Country-specific Requirements' and text added to describe EU Regulation 536/2024 (Annex VI, A1, paragraph 3).	vii. To clarify labeling requirements<br>viii. To clarify that as per EU Regulation 536/2024 (Annex VI, A1, paragraph 3), the address and telephone number of the main contact for information on the product and clinical trial is not in the label because it is present in the Patient ID card.
6.5.3 Safety Monitoring Committee<br>6.5.4 Definition of Dose-limiting Toxicity	Text added to describe criteria that would trigger an ad hoc SMC meeting to decide continuation or discontinuation of a subject, dose level or study part in case of unexpected toxicity.	To define criteria that would trigger an ad hoc SMC meeting as requested by HA.
6.5.5 Dose Modification	Modified text and tables on individual dose modifications, interruptions, or discontinuations for the combination partners.	To clarify the dose modification with respect to the different SoC compounds.
6.8.2 Permitted Medicines<br>6.8.3 Prohibited Medicines	Clarified the guidance on use of live and non-live vaccines before first M9140 dose and during the study.	To provide clearer guidance on vaccination prior and during the study.
6.8.3 Prohibited Medicines	Text added to refer to the local product labels for details on drug-drug interactions with capecitabine or bevacizumab.	Modified in line with reference to drug-drug interactions described in local product labels.
8.3 Adverse Events, Serious Adverse Events, and Other Safety Reporting<br>Appendix 11 Country-specific Requirements	Added definition of new fact.	Include definition of new fact as requested by HA.
8.3.3 Regulatory Reporting Requirements for Serious Adverse Events<br>Appendix 11 Country-specific Requirements	Added SUSAR reporting requirements according to EU Regulation 536/2014.	To clarify the SUSAR reporting requirements procedures according to EU Regulation 536/2014.
Throughout document	Minor editorial and document-formatting changes.	Throughout document

Protocol Version 4.0 (20 June 2024)

Overall Rationale for the Amendment

The protocol v4.0, dated 20 June 2024 was planned to introduce a new Part 2D that combines M9140 with 5-FU and bevacizumab. Protocol v4.0 was finalized and released but not submitted to any Health Authority. Meanwhile, feedback from European regulatory authorities triggered a local protocol amendment v3.3 EU. In order to achieve a harmonized protocol version again, all local changes and revised content from protocol v4.0 (introduction of Part 2D) were merged and integrated into a new protocol v5.0.

Section # and Name	Description of Change	Brief Rationale
1.1 Synopsis<br>1.2 Schema<br>1.3.5 Part 2D – M9140 [redacted] in Combination with 5-FU plus Bevacizumab<br>3.3 Part 2B (M9140 [redacted]), Part 2C (M9140 Q3W in Combination with Bevacizumab or Bevacizumab plus Capecitabine), and Part 2D (M9140 [redacted] in Combination with 5-FU plus Bevacizumab)<br>4.1 Overall Design<br>4.2.2 Parts 2A, 2B, 2C, and 2D – Dose Expansion<br>4.3.2.4 Part 2D – M9140 [redacted] in Combination with 5-FU plus Bevacizumab<br>5.2 Exclusion Criteria<br>6.1 Study Intervention Administration<br>6.4 Study Intervention Compliance<br>6.5.2 Dose Selection<br>6.5.3 Safety Monitoring Committee<br>6.5.4 Definition of Dose-limiting Toxicity<br>6.5.5 Dose Modification<br>6.8.2 Permitted Medicines<br>6.8.3 Prohibited Medicines<br>8.1 Efficacy Assessments and Procedures<br>8.2.4 Clinical Safety Laboratory Assessments<br>8.4 Pharmacokinetics<br>9.2 Sample Size Determination<br>9.4.2.2 Dose Recommendations for Expansion<br>9.4.4 Sequence of Analyses<br>10 References<br>Appendix 6	Addition of Part 2D with M9140 in combination with 5-FU, folinic acid, and bevacizumab.	To evaluate the potential of M9140 to be combined with standard of care agents in the treatment of mCRC.

Section # and Name	Description of Change	Brief Rationale
1.1 Synopsis<br>3 Objectives and Endpoints<br>9.4.1 Efficacy Analyses	Inclusion of a secondary endpoint to assess disease control rate in Part 2.	The dose escalation part demonstrated that a significant percentage of participants experienced a benefit from the treatment in terms of long-term disease stabilization that was also reflected in the mPFS observed. For that reason analyzing disease control rate is a relevant endpoint that would give a more complete picture of the anti-tumor activity of M9140.
1.1 Synopsis<br>1.3.2 Schedule of Activities Part 2A<br>4.1 Overall Design<br>4.4.2 End of Study Definition	Prolongation of maximum survival follow-up up to 24 months.	More mature data from the dose escalation part of the study have demonstrated treatment duration of > 12 cycles for some participants. Longer OS follow up will be needed to capture OS duration for those participants.
1.1 Synopsis	Removal of justification #2.	Legacy text removed to be consistent with main text.
1.3.4 Part 2C – M9140 Q3W in Combination with Bevacizumab or Bevacizumab plus Capecitabine<br>Appendix 6	Additions to Part 2C urinalysis: If dipstick proteinuria ≥ 2+, a 24-hour urine collection is required.	For quantitative assessment of proteinuria due to increased risk of proteinuria when treated with bevacizumab.
2.3.1 Risk Assessment	Benefit-risk assessment updated with details applicable for 5-FU and folinic acid.	Modified to align with risks for the study interventions to be administered in Part 2D.
4.1 Overall Design	Clarified that enrollment in Part 2C2 can only start after dose level 1 in Part 2C1 has been deemed safe and tolerable by the safety monitoring committee.	To clarify sequence of enrollment.
4.1 Overall Design<br>4.3.2.3 Part 2C Justification for Dose<br>9.4.2.2 Dose Recommendations for Expansion	Dose level 1 in Part 2C2 will include capecitabine at its lower approved dose (800 mg/m^2). If the dose is deemed tolerable by the SMC, capecitabine can be escalated to 1000 mg/m^2.	For participant safety purposes, dosing will start with the lower approved dose of capecitabine.
4.3.2 Parts 2A, 2B, 2C, and 2D – Dose Expansion	Updated with end of dose escalation data from Part 1.	To provide the most updated information available.
5 Study Population<br>5.1 Inclusion Criteria	Pasted details for participants with known BRAF mutation.	Aligned with text in Section 4.1.
5.1 Inclusion Criteria	The time period for avoiding breastfeeding is 3 months in Part 1, 2A, and 2B, and 6 months in Part 2C and 2D.	To align with the product information for the respective standard of care agents.
5.2 Exclusion Criteria	Legacy text about GC/[redacted] removed.	As per previous amendment, GC/[redacted] is no longer part of study population. Text removed to align.
	Exclusion criteria added for Part 2C and 2D.	To conform with the use of bevacizumab, capecitabine and 5-FU in these parts.

Section # and Name	Description of Change	Brief Rationale
6.5.5 Dose Modification	Included reference to local prescribing information of bevacizumab.	To conform with the use of bevacizumab.
	Table 19: Clarification that consideration on dose reduction applies only in cases of Grade 4 thrombocytopenia events with no complications that last ≥ 7 days.	Modified for clarity and consistency.
	• Dose-modification for capecitabine included only in Table 20.<br>• Added text to clarify that once dose of capecitabine has been reduced, it should not be increased at a later time.<br>• Added text on dose modification of capecitabine in case of decrease in neutrophil or platelet counts or creatinine clearance.	To clarify which guidelines to apply for managing adverse reactions to capecitabine.
	Addition of Table 21 for management (temporary/permanent discontinuation) of adverse reactions that may occur when administering bevacizumab.	To clarify management of adverse reactions to bevacizumab.
	Addition of Table 22 for management of adverse reactions that may occur when administering 5-FU.	To clarify management of adverse reactions to 5-FU.
6.7 Treatment of Overdose	Clarified that for any overdose of approved therapy, reference should be made to the local prescribing information on handling and side effects for the respective treatment.	To avoid listing details for each approved therapy regarding overdose symptoms and any local treatment details in the protocol.
6.8.3 Prohibited Medicines	Clarification that the period for prohibition of medicines is valid until Safety Follow-up Visit only, unless otherwise noted.	To clarify the end of restriction.
	Use of anticoagulants or thrombolytic agents is not allowed in Part 2C and 2D.	Modified in line with the newly added exclusion criterion conforming with the use of bevacizumab.
	Use of brivudine, sorivudine or their analogues is not allowed in Part 2C2 and 2D.	Modified in line with the newly added exclusion criterion conforming with the use of capecitabine.
	Clarification that also herbal/natural products which are potential CYP3A inhibitors/inducers are prohibited.	To clarify the extent of prohibition of CYP3A inhibitors/inducers.
6.8.4 Other Interventions	Treatment with bevacizumab should be interrupted for elective surgery.	To conform with the use of bevacizumab.

Section # and Name	Description of Change	Brief Rationale
	Clarification added that concurrent participation in other clinical studies is not permitted until Safety Follow-up Visit.	To clarify restrictions in terms of study participation.
7.1 Discontinuation of Study Intervention	Study intervention stopping criteria for liver events have been added.	Included for patient-safety purposes.
8.3.3 Regulatory Reporting Requirements for Serious Adverse Events<br>Appendix 4	Update of reporting procedure for SAEs and SUSARs.	To align with current Sponsor process.
8.3.7 Adverse Event of Special Interest<br>Appendix 4	Update of reporting procedure for AESIs.	To align with current Sponsor process.
8.4 Pharmacokinetics<br>8.6 Biomarkers<br>8.7 Immunogenicity Assessments	Removal of specific volumes to be collected.	To avoid local protocol amendments in case of differences in local standards.
9.4.1 Efficacy Analyses	Addition of details for analysis and of DoR and TTR.	To clearly define the planned analyses.
9.4.3 Other Analyses	Removal of non-renal clearance assessment for urine sample	Only renal clearance can be assessed in urine sample
9.4.4 Sequence of Analyses	Added option to perform exploratory analysis at end of dose optimization (Part 2A).	To ensure establishing optimal dose for future studies in a timely manner.
Appendix 2 Study Governance	Removal of language concerning the participant's legally authorized representative.	To align with current Sponsor protocol template
Appendix 6 Clinical Laboratory Tests	Clarified that coagulation profile testing is required also at End of Treatment and Safety Follow-up visits.	To clarify timepoints for assessment of coagulation.
Appendix 7 List of Strong and Moderate CYP Inhibitors and Inducers	Added link to website for more details on CYP inhibitors and inducers.	To make information easier accessible.
Appendix 11 Japan-specific Requirements	Addition of oral progesterone-only containing contraceptives to list of prohibited contraceptive methods in Japan.	To align with contraceptive methods permitted in Japan.
Throughout document	Minor editorial, typographical, and document-formatting changes	

Protocol Version 3.2 KR (22 May 2024)

Overall Rationale for the Amendment

The protocol is amended to revise the exclusion criteria for study Parts 2C1 and 2C2 to conform with the use of bevacizumab and capecitabine.

Section # and Name	Description of Change	Brief Rationale
1.3.4 Part 2C – M9140 Q3W in Combination with Bevacizumab or Bevacizumab plus Capecitabine<br>Appendix 6 Clinical Laboratory Tests	Additions to Part 2C urinalysis: If dipstick proteinuria ≥ 2+, a 24-hour urine collection is required.	For quantitative assessment of proteinuria due to increased risk of proteinuria when treated with bevacizumab.
5.2 Exclusion Criteria	Exclusion criteria added for Parts 2C1 and 2C2	To conform with the use of bevacizumab and capecitabine in these study parts.
6.5.5 Dose Modification	Included reference to SmPC of bevacizumab.	To conform with the use of bevacizumab in these study parts.
	Table 17: Clarification that consideration on dose reduction applies only in cases of Grade 4 thrombocytopenia events with no complications that last ≥ 7 days	Modified for clarity and consistency.
	• Dose-modification for capecitabine included only in Table 18.<br>• Added text to clarify that once dose of capecitabine has been reduced it should not be increased at a later time.<br>• Added text on dose modification of capecitabine in case of decreases in neutrophil or platelet counts or creatinine clearance	To clarify which guidelines to apply for managing adverse reactions to capecitabine.
	Addition of Table 19 for management (temporary/permanent discontinuation) of adverse reactions that may occur when administering bevacizumab.	To clarify management of adverse reactions to bevacizumab.
6.8.3 Prohibited Medicines	• Use of anticoagulants or thrombolytic agents is not allowed in Part 2C.<br>• Use of brivudine, sorivudine or their analogues are not allowed in Part 2C2	Modified in line with the newly added exclusion criterion conforming with the use of bevacizumab and capecitabine.
6.8.4 Other Interventions	Text added that for Part 2C only, treatment with bevacizumab should be interrupted for elective surgery	To conform with the use of bevacizumab.
Throughout document	Minor editorial and document-formatting changes.	

Protocol Version 3.1 US (03 April 2024)

Overall Rationale for the Amendment

The protocol is amended to revise the exclusion criteria for study Parts 2C1 and 2C2 to conform with the use of bevacizumab.

Section # and Name	Description of Change	Brief Rationale
1.3.4 Part 2C – M9140 Q3W in Combination with Bevacizumab or Bevacizumab plus Capecitabine<br>Appendix 6	Additions to Part 2C urinalysis: If dipstick proteinuria ≥ 2+, a 24-hour urine collection is required.	For quantitative assessment of proteinuria due to increased risk of proteinuria when treated with bevacizumab.
5.2 Exclusion Criteria	Exclusion criteria added for Parts 2C1 and 2C2	To conform with the use of bevacizumab in these study parts.
6.5.5 Dose Modification	• Included reference to SmPC of bevacizumab.<br>• Table 17: Clarification that consideration on dose reduction applies only in cases of Grade 4 thrombocytopenia events with no complications that last ≥ 7 days	• To conform with the use of bevacizumab in these study parts.<br>• Modified for clarity and consistency.
6.8.3 Prohibited Medicines	Use of anticoagulants is not allowed.	Modified in line with the newly added exclusion criterion conforming with the use of bevacizumab.

Protocol Version 3.0 (17 January 2024)

Overall Rationale for the Amendment

The protocol is amended to include preliminary safety information from first-in-human experience with M9140 as monotherapy and to revise Part 2 of the study (dose expansion). Part 2 will be limited to colorectal cancer and includes dose optimization (Part 2A), an alternative administration schedule ([redacted]) (Part 2B), and combination of M9140 with standard drugs (Part 2C).

In addition, mentions of gastric and [redacted] (GC/[redacted]) have been removed in this amendment and all previous local amendments have been combined.

Section # and Name	Description of Change	Brief Rationale
1.1 Synopsis<br>1.2 Schema<br>1.3 Schedule of Activities<br>2.1 Study Rationale<br>2.2 Background<br>2.3 Benefit/Risk<br>3. Objectives and Endpoints<br>4 Study Design<br>5 Study Population	• Added preliminary clinical (safety) information from the FIH Part 1	• To inform the rationale and study design for Part 2

Section # and Name	Description of Change	Brief Rationale
6 Study Intervention and Concomitant Therapies<br>8 Study Assessments and Procedures<br>9 Statistical Considerations<br>10 References<br>Throughout the document	• Revised study design and information for Part 2 Dose Expansion on the Part 2 (2A: M9140 monotherapy Q3W dose optimization, 2B: M9140 [redacted] administration, and 2C: M9140 Q3W in combination)<br>• Deleted references to GC/[redacted]<br>• Added PRO assessments	• To update the study design of Part 2 of the study that aims to evaluate safety and efficacy of the following Parts: Part 2A: two doses of M9140 monotherapy using Q3W dosing regimen to inform selection of optimized dose; Part 2B: monotherapy with [redacted] dosing regimen, and Part 2C: Q3W dosing regimen in combination with bevacizumab ± capecitabine.<br>• GC/[redacted] will not be evaluated as part of this study. This study will evaluate M9140 solely in participants with CRC.<br>• PRO will support dose selection in Part 2A of the study
1.1 Synopsis	Updated information relevant to Part 2 (study rationale, endpoints, sample size)	To reflect the changes in study design as outlined above
1.2 Schema	• Updated Part 2 overall schema<br>• Added Part 2C dose levels schema	To present the updated Part 2 study design and the dose levels in Part 2C
1.3 Schedule of Activities	• Added and updated SoAs to inform the procedures for each of the 3 parts in Part 2<br>• Removed central triplicate ECGs in Part 2B and 2C and reduced safety ECGs to only D1 of every other cycle	• To specify the procedures required in Part 2 (e.g. added PRO in Part 2A) and to update the visit times based on Part 1 experience (e.g., safety and labs to be assessed on D15 than D8 considering late toxicities and to better assess C_{max}, except in Part 2C with C1D8 assessments as toxicity of the combination may be expected earlier)<br>• ECGs in dose escalation did not show abnormalities and no impact on QTc was observed. QTc will be analyzed only in a subset of participants in Part 2A
2.1 Study Rationale	Added data from ongoing dose escalation Part 1 of the study	To provide clinical data to further support the rationale of Part 2 of the study
2.3 Benefit/Risk Assessment	Added data from ongoing dose escalation Part 1 of the study	To provide clinical data from dose escalation Part 1 of the study to support Benefit:Risk assessment
3 Objectives and Endpoints	Added Objectives and Endpoints for the updated parts of Part 2 of the study	New cohorts are introduced in the study with this amendment and list of objectives and endpoints needs to reflect these new parts
4.1 Overall Design	Added Parts 2A, 2B, and 2C	To present the revised design of the study
4.2 Scientific Rationale for the Study Design	Described Parts 2A, 2B, and 2C	To explain the revised design of the study
4.3 Justification for Dose	Added data from Part 1 dose escalation and from literature	To explain the rationale for the M9140 selected doses as single agent and in combination regimens.
4.4 End of Study Definition	Added the end of study definition for Part 2	To explain the EoS definition in Part 2

Section # and Name	Description of Change	Brief Rationale
5.1 Inclusion Criteria	Inclusion criteria for Parts 2A, 2B, and 2C were added to clarify that participants with only two previous treatment regimens will be eligible. Additionally, the possibility of an optional fresh biopsy was added for Parts 2A and 2C.	To describe inclusion criteria for new parts
6.1 Study Intervention Administration	• Added new information about study interventions (i.e., M9140 [redacted] [Part 2B] and bevacizumab and capecitabine [Part 2C])<br>• Added recommendation on order of administration of M9140, bevacizumab, and capecitabine in Part 2C	To add a new administration schedule ([redacted]) of M9140 as monotherapy<br>To add bevacizumab and capecitabine that will be administered in Part 2C
6.3.1 Study Intervention Assignment	Added that a randomized IRT system will be used in Part 2A to participants to be allocated to each dose level	To randomize participants to the high or low dose levels in Part 2A and ensure a balanced ratio of participants with a BMI-capped dose
6.4 Study Intervention Compliance	Added guidance to monitor participant's compliance referred to capecitabine	To monitor participant's compliance referred to capecitabine in Part 2C2
6.5.2 Dose Selection	Added criteria for progressing to a new cohort or dose level in Part 2	To explain the criteria for dose level selection in the revised study design
6.5.3 Safety Monitoring Committee	Added information on SMC for Part 2 of the study	To explain that an SMC will also be implemented in Part 2
6.5.4 Definition of Dose-Limiting Toxicity	Added the DLT period for Part 2B and Part 2C	To state that the DLT observation period in Part 2B and Part 2C will be [redacted] and 21 days, respectively
6.5.5 Dose Modification	Added recommendations for dose modifications in case of toxicity	To update the recommendations as new safety information became available and new drugs are used in the study
6.6 Continuous Access to Study Intervention After the End of Study	Added provisions for continuous access for participants that may benefit from the study intervention	To update the wording on continuous access
6.7 Treatment of Overdose	Updated information on M9140 overdose and added wording capecitabine overdose (Part 2C)	To include criteria for M9140 overdose and recommendations for capecitabine overdose management
6.8.2 Permitted Medicines	Clarified the use of preventative and concomitant therapy (e.g., G-CSF and vaccines) in Part 2	To provide information about the use of concomitant medications in Part 2
6.8.3 Prohibited Medicines	Added list of moderate CYP3A4 inhibitors and modified recommendation for strong CYP1A2 inhibitors/inducers	To complete the list with moderate CYP3A4 inhibitors with potential for DDIs with exatecan.<br>Modification for CYP1A2 based on new data.

Section # and Name	Description of Change	Brief Rationale
7.1 Discontinuation of Study Intervention	Added OS follow-up for Part 2A	To specify that participants will be followed up for OS in Part 2A
8.1 Efficacy Assessments	Added timing of CT assessments in Part 2	To specify frequency CT assessments in Part 2
8.2.3 Electrocardiograms	Removed central triplicate ECGs from Part 2B and 2C and reduced safety ECGs to only D1 of every other cycle	ECGs in dose escalation did not show abnormalities and no impact on QTc was observed. Triplicate ECGs will be analyzed only in a subset of participants in Part 2A
8.2.5 Patient-reported Symptomatic AEs<br>9.4.3 Other Analysis<br>Appendix 12 NCI-PRO-CTCAE	• Added PRO assessments for patient-reported symptomatic AEs.<br>• Added adapted NCI-PRO CTCAE questionnaire	PRO collected in Part 2A will be considered to support dose selection
8.3.7 Adverse Events of Special Interest	Added Grade ≥ 3 neutropenia or thrombocytopenia with complications as AESI for Part 2	To update the recommendations as new safety information became available
8.4 Pharmacokinetics	Added bevacizumab and capecitabine PK	To evaluate the PK profile of bevacizumab and capecitabine in combination with M9140
8.6 Biomarkers	Added that optional fresh biopsies may be obtained in Part 2A and Part 2C	To update the tissue biopsies requirements in Part 2
9 Statistical Considerations<br>Appendix 8 Model for Bayesian Dose Escalation	• Updated sample size<br>• Updated efficacy and safety analyses to include Part 2<br>• Updated interim futility analysis in Part 2A<br>• Added statistical considerations for dose selection in Part 2A (dose optimization)<br>• Updated efficacy and safety analyses to include Part 2.	To explain the statistical analyses in the revised study design
Appendix 2 Study Governance	Added wording related to Data Protection, Regulatory and Ethical Considerations, and Data Quality Assurance.	To comply with EU Clinical Trial Regulation
Appendix 6 Clinical Laboratory Tests	Added ferritin and specifications for coagulation and reticulocytes	To update safety laboratory assessments for Part 2
Appendix 7 List of Strong CYP Inhibitors and Inducers	Added list of moderate CYP3A4 inhibitors	To complete the list with moderate CYP3A4 inhibitors with potential for DDIs with exatecan
Appendix 10 Protocol Amendment History	Consolidated all protocol amendment history from the country-specific amendments	To track all the protocol changes made in a common protocol

Section # and Name	Description of Change	Brief Rationale
Appendix 11 Japan-specific Requirements<br>Throughout document	• Added Japan-specific requirements.<br>• Added cross-references to Appendix 11 as appropriate	To include country-specific information into the common protocol
Throughout document	• Consolidated information from the local amendments<br>• Removed redundant PDX and preclinical information that appeared across multiple sections<br>• Minor editorial and document formatting changes.	• To have a single, common protocol for all countries<br>• To simplify and improve readability<br>• Minor, therefore changes have not been summarized.

Protocol Version 2.8 JP (23 August 2023)

Overall Rationale for the Amendment (vs. Version 2.5 JP)

The protocol is amended to implement a maximum absolute dose limit (dose cap) corresponding to a weight that, based on the patient's height, corresponds to a BMI of 30 kg/m^2 for participants, whose BMI is greater than 30 kg/m^2. With this change, obese patients may be protected from excessive exposure and potential increased risk for treatment related adverse events.

Section # and Name	Description of Change	Brief Rationale
1.3 Schedule of Activities (Table 1, Table 3)<br>Appendix 10 (Table 16 and Table 18)<br>4.1 Overall Design<br>8.2.2 Vital Signs	• Added BMI to the vital signs measurements	BMI must be calculated as a BMI greater than 30 kg/m^2 is the threshold for dose cap.
1.3 Schedule of Activities (Table 1 and Table 3)<br>8.1 Efficacy Assessments and Procedures<br>Appendix 10 (Table 16 and Table 18)	• Clarified the timing of tumor assessments.	To clarify that after the 3rd tumor scan (Screening, Evaluation 1, Evaluation 2) evaluations will be done every 9 weeks (±7 days). Tumor assessment at EOT if clinically applicable.
1.3 Schedule of Activities (Table 2, Table 4)<br>Appendix 10 (Table 17, Table 19)	• Clarified that PK, [redacted], and ADA sampling occurs at predose every 2 cycles from C4 until treatment discontinuation rather than until EOT. No samples are needed for the EOT visit.	• To clarify the timing of PK, [redacted], and ADA sampling after C4.<br>• To clarify that no samples are needed for the EOT visit.

Section # and Name	Description of Change	Brief Rationale
2.3.1 Risk Assessment<br>4.1 Overall Design<br>4.2 Scientific Rationale for Study Design<br>4.3 Justification for Dose	• Added rationale for the maximum absolute dose limit (dose cap) in obese participants with a BMI greater than 30 kg/m^2.<br>• Added dosing guidance for participants whose BMI is greater than 30 kg/m^2 as follows:<br>• The dose (in mg) to be administered to each participant will be calculated based on the participant's weight rounded to the nearest kilogram, i.e., assigned cohort dose level in mg/kg * body weight in kg.<br>• For participants whose BMI is greater than 30 kg/m^2, the investigator should use a weight that, based on the participant's height, corresponds to a maximum BMI of 30 and calculate the dose according to the formula provided.	The dose cap for participants whose BMI is greater than 30 kg/m^2 is being implemented to protect obese patients from excessive exposure and potential increased risk for TRAEs.
6.1 Study Intervention Administration	• Added that G-CSF will be provided by the Sponsor.	To clarify the sourcing of G-CSF.
6.5.2 Dose Selection<br>9.4.2 Safety Analyses<br>9.4.2.1 Dose Escalation	• Specified that at least 2 participants in each cohort should have received ≥ 80% of the actual non-capped dose.<br>• Added sensitivity analysis (BLRM with actual received dose level (based on absolute dose). For participants whose absolute dose was capped due to their BMI, the SMC will receive results from the same Bayesian model, where capped participants are considered in the dose level (in mg/kg) that matches their actual received (capped) dose (in mg).	To clarify the composition of each cohort with respect to capped doses as well as how data from participants whose absolute dose was capped will be analyzed.
6.5.5 Dose Modifications	• Added that physicians, based on their clinical judgment and after discussion with the Sponsor, may diverge from these recommendations if clinically indicated.	Since this protocol cannot provide guidance for all circumstances, it is important to permit the possibility to deviate from the general dose modification guidance in justified cases, if clinically indicated and after discussion with the Sponsor.
Throughout document	• Minor editorial and document formatting changes.	Minor, therefore changes have not been summarized.

Protocol Version 2.7 US (23 August 2023)

Overall Rationale for the Amendment (vs. Version 2.4 US)

The protocol is amended to implement a maximum absolute dose limit (dose cap) corresponding to a weight that, based on the patient's height, corresponds to a BMI of 30 kg/m^2 for participants, whose BMI is greater than 30 kg/m^2. With this change, obese patients may be protected from excessive exposure and potential increased risk for treatment related adverse events.

Section # and Name	Description of Change	Brief Rationale
1.3 Schedule of Activities (Table 1, Table 3)<br>4.1 Overall Design<br>8.2.2 Vital Signs	• Added BMI to the vital signs measurements	BMI must be calculated as a BMI greater than 30 kg/m^2 is the threshold for dose cap.
1.3 Schedule of Activities (Table 1 and Table 3)<br>8.1 Efficacy Assessments and Procedures	• Clarified the timing of tumor assessments.	To clarify that after the 3rd tumor scan (Screening, Evaluation 1, Evaluation 2) evaluations will be done every 9 weeks (±7 days). Tumor assessment at EOT if clinically applicable.
1.3 Schedule of Activities (Table 2, Table 4)	• Clarified that PK, [redacted], and ADA sampling occurs at predose every 2 cycles from C4 until treatment discontinuation rather than until EOT. No samples are needed for the EOT visit.	• To clarify the timing of PK, [redacted], and ADA sampling after C4.<br>• To clarify that no samples are needed for the EOT visit.
2.3.1 Risk Assessment<br>4.1 Overall Design<br>4.2 Scientific Rationale for Study Design<br>4.3 Justification for Dose	• Added rationale for the maximum absolute dose limit (dose cap) in obese participants with a BMI greater than 30 kg/m^2.<br>• Added dosing guidance for participants whose BMI is greater than 30 kg/m^2 as follows:<br>• The dose (in mg) to be administered to each participant will be calculated based on the participant's weight rounded to the nearest kilogram, i.e., assigned cohort dose level in mg/kg * body weight in kg.<br>• For participants whose BMI is greater than 30 kg/m^2, the investigator should use a weight that, based on the participant's height, corresponds to a maximum BMI of 30 and calculate the dose according to the formula provided.	The dose cap for participants whose BMI is greater than 30 kg/m^2 is being implemented to protect obese patients from excessive exposure and potential increased risk for TRAEs.
6.5.2 Dose Selection<br>9.4.2 Safety Analyses<br>9.4.2.1 Dose Escalation	• Specified that at least 2 participants in each cohort should have received ≥ 80% of the actual non-capped dose.	To clarify the composition of each cohort with respect to capped doses as well as how data from participants whose absolute dose was capped will be analyzed.

Section # and Name	Description of Change	Brief Rationale
	• Added sensitivity analysis (BLRM with actual received dose level (based on absolute dose). For participants whose absolute dose was capped due to their BMI, the SMC will receive results from the same Bayesian model, where capped participants are considered in the dose level (in mg/kg) that matches their actual received (capped) dose (in mg).	
6.5.5 Dose Modifications	• Added that physicians, based on their clinical judgment and after discussion with the Sponsor, may diverge from these recommendations if clinically indicated.	Since this protocol cannot provide guidance for all circumstances, it is important to permit the possibility to deviate from the general dose modification guidance in justified cases, if clinically indicated and after discussion with the Sponsor.
Throughout document	• Minor editorial and document formatting changes.	Minor, therefore changes have not been summarized.

Protocol Version 2.6 ES (23 August 2023)

Overall Rationale for the Amendment (vs. Protocol Version 2.3 ES)

The protocol is amended to implement a maximum absolute dose limit (dose cap) corresponding to a weight that, based on the patient's height, corresponds to a BMI of 30 kg/m^2 for participants, whose BMI is greater than 30 kg/m^2. With this change, obese patients may be protected from excessive exposure and potential increased risk for treatment related adverse events.

Section # and Name	Description of Change	Brief Rationale
1.3 Schedule of Activities (Table 1, Table 3)<br>Appendix 10 (Table 16 and Table 18)<br>4.1 Overall Design<br>8.2.2 Vital Signs	• Added BMI to the vital signs measurements	BMI must be calculated as a BMI greater than 30 kg/m^2 is the threshold for dose cap.
1.3 Schedule of Activities (Table 1 and Table 3)<br>8.1 Efficacy Assessments and Procedures<br>Appendix 10 (Table 16 and Table 18)	• Clarified the timing of tumor assessments.	To clarify that after the 3rd tumor scan (Screening, Evaluation 1, Evaluation 2) evaluations will be done every 9 weeks (±7 days). Tumor assessment at EOT if clinically applicable.
1.3 Schedule of Activities (Table 2, Table 4)<br>Appendix 10 (Table 17, Table 19)	• Clarified that PK, [redacted], and ADA sampling occurs at predose every 2 cycles from C4 until treatment discontinuation rather than until EOT. No samples are needed for the EOT visit.	• To clarify the timing of PK, [redacted], and ADA sampling after C4.<br>• To clarify that no samples are needed for the EOT visit.
2.3.1 Risk Assessment<br>4.1 Overall Design<br>4.2 Scientific Rationale for Study Design<br>4.3 Justification for Dose	• Added rationale for the maximum absolute dose limit (dose cap) in obese participants with a BMI greater than 30 kg/m^2.<br>• Added dosing guidance for participants whose BMI is greater than 30 kg/m^2 as follows:<br>• The dose (in mg) to be administered to each participant will be calculated based on the participant's weight rounded to the nearest kilogram, i.e., assigned cohort dose level in mg/kg * body weight in kg.<br>• For participants whose BMI is greater than 30 kg/m^2, the investigator should use a weight that, based on the participant's height, corresponds to a maximum BMI of 30 and calculate the dose according to the formula provided.	The dose cap for participants whose BMI is greater than 30 kg/m^2 is being implemented to protect obese patients from excessive exposure and potential increased risk for TRAEs.

Section # and Name	Description of Change	Brief Rationale
6.5.2 Dose Selection<br>9.4.2 Safety Analyses<br>9.4.2.1 Dose Escalation	• Specified that at least 2 participants in each cohort should have received ≥ 80% of the actual non-capped dose.<br>• Added sensitivity analysis (BLRM with actual received dose level (based on absolute dose). For participants whose absolute dose was capped due to their BMI, the SMC will receive results from the same Bayesian model, where capped participants are considered in the dose level (in mg/kg) that matches their actual received (capped) dose (in mg).	To clarify the composition of each cohort with respect to capped doses as well as how data from participants whose absolute dose was capped will be analyzed.
6.5.5 Dose Modifications	• Added that physicians, based on their clinical judgment and after discussion with the Sponsor, may diverge from these recommendations if clinically indicated.	Since this protocol cannot provide guidance for all circumstances, it is important to permit the possibility to deviate from the general dose modification guidance in justified cases, if clinically indicated and after discussion with the Sponsor.
Throughout document	• Minor editorial and document formatting changes.	Minor, therefore changes have not been summarized.

Protocol Version 2.5 JP (27 April 2023)

Overall Rationale for the Amendment (vs. Protocol Version 2.2 JP)

Part 1 of the protocol (dose escalation of M9140 Q3W) has been amended to modify the definition of dose-limiting toxicity with respect to the occurrence of hematological toxicity (severe thrombocytopenia and neutropenia) and to include an additional dose escalation cohort (Part 1B) in which prophylactic pegfilgrastim administration will be mandatory in the first 2 cycles of M9140 administration. Potential changes resulting from the modified Part 1 and affecting Part 2 (dose expansion) might be implemented via an amendment at a later timepoint after finalization of the dose escalation part.

Section # and Name	Description of Change	Brief Rationale
1.1 Synopsis	• Added RDE/MTD endpoints for the Part 1B dose escalation (M9140 plus pegfilgrastim).<br>• Updated sample size.	To align the language with the body text regarding the addition of Part 1B dose escalation cohorts.
1.2 Schema	• Added schema for Part 1B.	To include the new Part 1B dose escalation cohorts.
1.3 Schedule of Activities	• Added pegfilgrastim for Part 1B.<br>• Minor clarifications in Table 2.<br>• Added blood draw on C2D15 for Part 1A and Part 1B.	• Clarification/consequence of the introduction of Part 1B.<br>• Clarification on sample collection.<br>• To monitor neutrophil levels after the first 2 cycles of M9140.
2.3.1 Risk Assessment	• Modified the management of the potential risk of neutropenia by introducing an additional dose escalation regimen with pegfilgrastim as primary prophylaxis for neutropenia.	• To update the mitigation strategy to address the risk of neutropenia.
4.1 Overall Design	• Updated the following in Part 1 only:<br>• Clarified that participants may have received previous treatment with CCI [redacted], if locally indicated and available to the patient.<br>• Updated the sample size to include Part 1B.<br>• Updated the adaptive aspects of the study design to include choice of starting dose for escalation with pegfilgrastim support.<br>• Described the difference of the Q3W M9140 regimen in terms of pegfilgrastim / G-CSF administrations between Part 1A (G-CSF support permitted only outside of DLT period) and Part 1B (pegfilgrastim primary prophylaxis on day 8 mandatory in first 2 cycles).	• To clarify inclusion criteria with regards to previous treatment with CCI [redacted].<br>• To describe how Part 1B of the study will be conducted.<br>• To further describe the detailed conduct of the 7-day observation period.

Section # and Name	Description of Change	Brief Rationale
	• Added Part 1B and specified that enrollment may start after SMC recommendation (and Sponsor agreement) and will proceed in parallel to Part 1A escalation.<br>• Added wording on 7 days observation period before inclusion of additional participants on each dose level for Part 1A and 1B.	
4.2 Scientific Rationale for Study Design	• Added the rationale for including Part 1B dose escalation.	Neutropenia is a well described and common AE for exatecan and has been reported as the DLT in patients treated with exatecan in various Phase I studies. Neutropenia Grade 3 and 4 AEs have been observed in the ongoing dose escalation, Part 1 of the MS202329_001 study.<br>Mandatory pegfilgrastim prophylaxis will reduce the risk of a pronounced (nadir) neutropenia/decreased neutrophil count as well as febrile neutropenia at higher doses of M9140, will improve patient convenience, and will allow higher doses of M9140 to be tested that may result in earlier and more deep responses. It may also facilitate potential combinations of M9140 with other anticancer agents in future studies.<br>Pegfilgrastim is commonly used in routine clinical practice, has a well described safety profile, and is recommended by clinical scientific experts.
5.1 Inclusion Criteria	• In Part 1, clarified that participants may have received previous treatment with [redacted], if locally indicated and available to the patient.<br>• Specification of blood glucose value	• To clarify inclusion criteria with regards to previous treatment with [redacted].<br>• To clarify blood glucose value to be assessed in fasted state.
6.1 Study Intervention Administration	• Added pegfilgrastim as auxiliary medication in the study.	To describe details for the administration of this auxiliary medication used in the study.
6.3.1 Study Intervention Assignment	• Specified how participants will be assigned to the dose escalation levels in Part 1A or Part 1B, following SMC decision and preferentially filling Part 1A first.	To explain how participants will be assigned to Part 1A and Part 1B.
6.5.2 Dose Selection	• Specified that the SMC will recommend the starting dose of the Part 1B dose escalation using the same procedures as for Part 1A.	To describe how the recommended doses in Part 1B will be calculated.

Section # and Name	Description of Change	Brief Rationale
6.5.4 Definition of Dose-limiting Toxicity	• Deleted that treatment with transfusions of blood cells or hematopoietic growth factors (e.g., G-CSF, erythropoietin) is not permitted during the DLT period and if it occurs will be considered a DLT and specified how intercurrent events will be analyzed statistically.<br>• Specified that the any Grade 4 thrombocytopenia of < 7 days duration not associated with any clinical symptoms or medically concerning bleeding is not considered a DLT.	Hematopoietic AEs potentially requiring blood cell transfusions and hematopietic growth factor administrations are expected for exatecan and common routine practice for certain chemotherapeutic regimens. Since prophylactic pegfilgrastim use during the first 2 cycles was included as mandatory to explore higher dose levels of M9140 per recommendation of the study investigators and the SMC, growth factor use had to be excluded as a DLT-defining criterion in Part 1B.<br>According to the literature spontaneous bleeding events are rare in case of short-lived, Grade 4 thrombocytopenia. Grade 4 events that have no clinically relevant bleeding complications, are of limited clinical relevance and should not be considered as dose limiting toxicities. Therefore, a protocol modification is proposed to define only long-lived Grade 4 thrombocytopenia (lasting ≥7 days) or those events complicated with clinically relevant bleeding as DLTs. This modification was discussed and endorsed by the SMC members and participating Investigators.
6.5.5 Dose Modification	• Clarified the management of neutropenia with G-CSF.	Clarification/consequence of the introduction of Part 1B.
6.8.2 Permitted Medicines	• Updated to allow use of G-CSF during the DLT observation period for patients who have developed neutropenia in Part 1A. Also, for patients who have experienced Grade ≥ 3 neutropenia and/or febrile neutropenia prophylactic administration of G-CSF is permitted for subsequent cycles according to physician's discretion.	Clarification/consequence of the introduction of Part 1B.
6.8.3 Prohibited Medicines	• Specified that prophylactic use of G-CSF is not permitted during the DLT observation period in Part 1A.	Clarification/consequence of the introduction of Part 1B.
8.2.3 Electrocardiograms	• Specified ECG collection.	Minor corrections for clarification of ECG collection.
8.4 Pharmacokinetics	• Specified PK urine sample collection.	Minor correction for clarification of PK urine sample collection
9.2 Sample Size Determination	• Updated sample size to include Part 1B dose escalation cohorts.	To reflect the additional Part 1B cohorts.
9.4 Statistical Analyses	• Specified that separate BLRMs will be used for Part 1A and Part 1B.	To describe how Part 1A and Part 1B will be analyzed.
9.4.2.1 Dose Escalation	• Specified the allowed dose increases in Part 1B for the SMC recommendations.<br>• Added that separate BLRMs will be used for Part 1A and Part 1B but with the same specifications and prior parameter settings.	To describe how the dose increases will be calculated and how Part 1A and Part 1B will be analyzed.

Section # and Name	Description of Change	Brief Rationale
Appendix 2. Study Governance	• Added scientific rationale for submission of the summary of clinical study results beyond 1 year from the end of the clinical trial in all EU member states	To clarify submission of the summary of clinical study results
Appendix 6. Clinical Laboratory Tests	• Added calcium corrected.<br>• CCI [redacted].<br>• Specified Laboratory Test on C2D15.	Minor correction for clarification.
Appendix 8. Model for Bayesian Dose Escalation	• Added preselected dose levels for Part 1B dose escalation.	To specify the preselected dose levels for Part 1B.
Appendix 12. Sponsor Signature Page	• Changed the name of the Sponsor representative.	To reflect the change in staff responsibilities.
Throughout	• Minor editorial and document formatting revisions.	Minor; therefore, have not been summarized.

Protocol Version 2.4 US (28 April 2023)

Overall Rationale for the Amendment (vs. Protocol Version 2.1 US)

Part 1 of the protocol (dose escalation of M9140 Q3W) has been amended to modify the definition of dose-limiting toxicity with respect to the occurrence of hematological toxicity (severe thrombocytopenia and neutropenia) and to include an additional dose escalation cohort (Part 1B) in which prophylactic pegfilgrastim administration will be mandatory in the first 2 cycles of M9140 administration. Potential changes resulting from the modified Part 1 and affecting Part 2 (dose expansion) might be implemented via an amendment at a later timepoint after finalization of the dose escalation part.

Section # and Name	Description of Change	Brief Rationale
1.1 Synopsis	• Added RDE/MTD endpoints for the Part 1B dose escalation (M9140 plus pegfilgrastim).<br>• Updated sample size.	To align the language with the body text regarding the addition of Part 1B dose escalation cohorts.
1.2 Schema	• Added schema for Part 1B.	To include the new Part 1B dose escalation cohorts.
1.3 Schedule of Activities	• Added pegfilgrastim for Part 1B.<br>• Minor clarifications in Table 2.<br>• Added blood draw on C2D15 for Part 1A and Part 1B.	• Clarification/consequence of the introduction of Part 1B.<br>• Clarification on sample collection.<br>• To monitor neutrophil levels after the first 2 cycles of M9140.
2.3.1 Risk Assessment	• Modified the management of the potential risk of neutropenia by introducing an additional dose escalation regimen with pegfilgrastim as primary prophylaxis for neutropenia.	To update the mitigation strategy to address the risk of neutropenia.
4.1 Overall Design	• Updated the following in Part 1 only:<br>• Clarified that participants may have received previous treatment with CCI [redacted], if locally indicated and available to the patient.<br>• Updated the sample size to include Part 1B.<br>• Updated the adaptive aspects of the study design to include choice of starting dose for escalation with pegfilgrastim support.<br>• Described the difference of the Q3W M9140 regimen in terms of pegfilgrastim / G-CSF administrations between Part 1A (G-CSF support permitted only outside of DLT period) and Part 1B (pegfilgrastim primary prophylaxis on day 8 mandatory in first 2 cycles).	• To clarify inclusion criteria with regards to previous treatment with CCI [redacted]<br>• To describe how Part 1B of the study will be conducted.<br>• To further describe the detailed conduct of the 7-day observation period.

Section # and Name	Description of Change	Brief Rationale
	• Added Part 1B and specified that enrollment may start after SMC recommendation (and Sponsor agreement) and will proceed in parallel to Part 1A escalation.<br>• Added wording on 7 days observation period before inclusion of additional participants on each dose level for Part 1A and 1B.	
4.2 Scientific Rationale for Study Design	• Added the rationale for including Part 1B dose escalation.	Neutropenia is a well described and common AE for exatecan and has been reported as the DLT in patients treated with exatecan in various Phase I studies. Neutropenia Grade 3 and 4 AEs have been observed in the ongoing dose escalation, Part 1 of the MS202329_001 study.<br>Mandatory pegfilgrastim prophylaxis will reduce the risk of a pronounced (nadir) neutropenia/decreased neutrophil count as well as febrile neutropenia at higher doses of M9140, will improve patient convenience, and will allow higher doses of M9140 to be tested that may result in earlier and more deep responses. It may also facilitate potential combinations of M9140 with other anticancer agents in future studies.<br>Pegfilgrastim is commonly used in routine clinical practice, has a well described safety profile, and is recommended by clinical scientific experts.
5.1 Inclusion Criteria	• In Part 1, clarified that participants may have received previous treatment with [redacted], if locally indicated and available to the patient.<br>• Specification of blood glucose value	• To clarify inclusion criteria with regards to previous treatment with [redacted]<br>• To clarify blood glucose value to be assessed in fasted state.
6.1 Study Intervention Administration	• Added pegfilgrastim as auxiliary medication in the study.	To describe details for the administration of this auxiliary medication used in the study.
6.3.1 Study Intervention Assignment	• Specified how participants will be assigned to the dose escalation levels in Part 1A or Part 1B, following SMC decision and preferentially filling Part 1A first.	To explain how participants will be assigned to Part 1A and Part 1B.
6.5.2 Dose Selection	• Specified that the SMC will recommend the starting dose of the Part 1B dose escalation using the same procedures as for Part 1A.	To describe how the recommended doses in Part 1B will be calculated.

Section # and Name	Description of Change	Brief Rationale
6.5.4 Definition of Dose-limiting Toxicity	• Deleted that treatment with transfusions of blood cells or hematopoietic growth factors (e.g., G-CSF, erythropoietin) is not permitted during the DLT period and if it occurs will be considered a DLT and specified how intercurrent events will be analyzed statistically.<br>• Specified that the any Grade 4 thrombocytopenia of < 7 days duration not associated with any clinical symptoms or medically concerning bleeding is not considered a DLT.	Hematopoietic AEs potentially requiring blood cell transfusions and hematopietic growth factor administrations are expected for exatecan and common routine practice for certain chemotherapeutic regimens. Since prophylactic pegfilgrastim use during the first 2 cycles was included as mandatory to explore higher dose levels of M9140 per recommendation of the study investigators and the SMC, growth factor use had to be excluded as a DLT-defining criterion in Part 1B.<br>According to the literature spontaneous bleeding events are rare in case of short-lived, Grade 4 thrombocytopenia. Grade 4 events that have no clinically relevant bleeding complications, are of limited clinical relevance and should not be considered as dose limiting toxicities. Therefore, a protocol modification is proposed to define only long-lived Grade 4 thrombocytopenia (lasting ≥ 7 days) or those events complicated with clinically relevant bleeding as DLTs. This modification was discussed and endorsed by the SMC members and participating investigators.
6.5.5 Dose Modification	• Clarified the management of neutropenia with G-CSF.	Clarification/consequence of the introduction of Part 1B.
6.8.2 Permitted Medicines	• Updated to allow use of G-CSF during the DLT observation period for patients who have developed neutropenia in Part 1A. Also, for patients who have experienced Grade ≥ 3 neutropenia and/or febrile neutropenia prophylactic administration of G-CSF is permitted for subsequent cycles according to physician's discretion.	Clarification/consequence of the introduction of Part 1B.
6.8.3 Prohibited Medicines	• Specified that prophylactic use of G-CSF is not permitted during the DLT observation period in Part 1A.	Clarification/consequence of the introduction of Part 1B.
8.2.3 Electrocardiograms	• Specified ECG collection.	Minor corrections for clarification of ECG collection.
8.4 Pharmacokinetics	• Specified PK urine sample collection.	Minor correction for clarification of PK urine sample collection
9.2 Sample Size Determination	• Updated sample size to include Part 1B dose escalation cohorts.	To reflect the additional Part 1B cohorts.
9.4 Statistical Analyses	• Specified that separate BLRMs will be used for Part 1A and Part 1B.	To describe how Part 1A and Part 1B will be analyzed.
9.4.2.1 Dose Escalation	• Specified the allowed dose increases in Part 1B for the SMC recommendations.<br>• Added that separate BLRMs will be used for Part 1A and Part 1B but with the same specifications and prior parameter settings.	To describe how the dose increases will be calculated and how Part 1A and Part 1B will be analyzed.

Section # and Name	Description of Change	Brief Rationale
Appendix 2. Study Governance	• Added scientific rationale for submission of the summary of clinical study results beyond 1 year from the end of the clinical trial in all EU member states	To clarify submission of the summary of clinical study results
Appendix 6. Clinical Laboratory Tests	• Added calcium corrected.<br>• CCI [redacted].<br>• Specified Laboratory Test on C2D15.	Minor correction for clarification.
Appendix 8. Model for Bayesian Dose Escalation	• Added preselected dose levels for Part 1B dose escalation.	To specify the preselected dose levels for Part 1B.
Appendix 12. Sponsor Signature Page	• Changed the name of the Sponsor representative.	To reflect the change in staff responsibilities.
Throughout	• Minor editorial and document formatting revisions.	Minor; therefore, have not been summarized.

Protocol Version 2.3 ES (25 April 2023)

Overall Rationale for the Amendment (vs. Protocol Version 2.0)

Part 1 of the protocol (dose escalation of M9140 Q3W) has been amended to modify the definition of dose-limiting toxicity with respect to the occurrence of hematological toxicity (severe thrombocytopenia and neutropenia) and to include an additional dose escalation cohort (Part 1B) in which prophylactic pegfilgrastim administration will be mandatory in the first 2 cycles of M9140 administration. Potential changes resulting from the modified Part 1 and affecting Part 2 (dose expansion) might be implemented via an amendment at a later timepoint after finalization of the dose escalation part.

Section # and Name	Description of Change	Brief Rationale
1.1 Synopsis	• Added RDE/MTD endpoints for the Part 1B dose escalation (M9140 plus pegfilgrastim).<br>• Updated sample size.	To align the language with the body text regarding the addition of Part 1B dose escalation cohorts.
1.2 Schema	• Added schema for Part 1B.	To include the new Part 1B dose escalation cohorts.
1.3 Schedule of Activities	• Added pegfilgrastim for Part 1B.<br>• Minor clarifications in Table 2.<br>• Added blood draw on C2D15 for Part 1A and Part 1B.	• Clarification/consequence of the introduction of Part 1B.<br>• Clarification on sample collection.<br>• To monitor neutrophil levels after the first 2 cycles of M9140.
2.3.1 Risk Assessment	• Modified the management of the potential risk of neutropenia by introducing an additional dose escalation regimen with pegfilgrastim as primary prophylaxis for neutropenia.	To update the mitigation strategy to address the risk of neutropenia.
4.1 Overall Design	Updated the following in Part 1 only:<br>• Clarified that participants may have received previous treatment with CCI [redacted], if locally indicated and available to the patient.<br>• Updated the sample size to include Part 1B.<br>• Updated the adaptive aspects of the study design to include choice of starting dose for escalation with pegfilgrastim support.<br>• Described the difference of the Q3W M9140 regimen in terms of pegfilgrastim / G-CSF administrations between Part 1A (G-CSF support permitted only outside of DLT period) and Part 1B (pegfilgrastim primary prophylaxis on day 8 mandatory in first 2 cycles).	• To clarify inclusion criteria with regards to previous treatment with CCI [redacted]<br>• To describe how Part 1B of the study will be conducted.<br>• To further describe the detailed conduct of the 7-day observation period.

Section # and Name	Description of Change	Brief Rationale
	• Added Part 1B and specified that enrollment may start after SMC recommendation (and Sponsor agreement) and will proceed in parallel to Part 1A escalation.<br>• Added wording on 7 days observation period before inclusion of additional participants on each dose level for Part 1A and 1B.	
4.2 Scientific Rationale for Study Design	• Added the rationale for including Part 1B dose escalation.	Neutropenia is a well described and common AE for exatecan and has been reported as the DLT in patients treated with exatecan in various Phase I studies. Neutropenia Grade 3 and 4 AEs have been observed in the ongoing dose escalation, Part 1 of the MS202329_001 study.<br>Mandatory pegfilgrastim prophylaxis will reduce the risk of a pronounced (nadir) neutropenia/decreased neutrophil count as well as febrile neutropenia at higher doses of M9140, will improve patient convenience, and will allow higher doses of M9140 to be tested that may result in earlier and more deep responses. It may also facilitate potential combinations of M9140 with other anticancer agents in future studies.<br>Pegfilgrastim is commonly used in routine clinical practice, has a well described safety profile, and is recommended by clinical scientific experts.
5.1 Inclusion Criteria	• In Part 1, clarified that participants may have received previous treatment with CCI [redacted] locally indicated and available to the patient.<br>• Specification of blood glucose value	• To clarify inclusion criteria with regards to previous treatment with CCI [redacted]<br>• To clarify blood glucose value to be assessed in fasted state.
6.1 Study Intervention Administration	• Added pegfilgrastim as auxiliary medication in the study.	To describe details for the administration of this auxiliary medication used in the study.
6.3.1 Study Intervention Assignment	• Specified how participants will be assigned to the dose escalation levels in Part 1A or Part 1B, following SMC decision and preferentially filling Part 1A first.	To explain how participants will be assigned to Part 1A and Part 1B.
6.5.2 Dose Selection	• Specified that the SMC will recommend the starting dose of the Part 1B dose escalation using the same procedures as for Part 1A.	To describe how the recommended doses in Part 1B will be calculated.

Section # and Name	Description of Change	Brief Rationale
6.5.4 Definition of Dose-limiting Toxicity	• Deleted that treatment with transfusions of blood cells or hematopoietic growth factors (e.g., G-CSF, erythropoietin) is not permitted during the DLT period and if it occurs will be considered a DLT and specified how intercurrent events will be analyzed statistically.<br>• Specified that the any Grade 4 thrombocytopenia of < 7 days duration not associated with any clinical symptoms or medically concerning bleeding is not considered a DLT.	Hematopoietic AEs potentially requiring blood cell transfusions and hematopietic growth factor administrations are expected for exatecan and common routine practice for certain chemotherapeutic regimens. Since prophylactic pegfilgrastim use during the first 2 cycles was included as mandatory to explore higher dose levels of M9140 per recommendation of the study investigators and the SMC, growth factor use had to be excluded as a DLT-defining criterion in Part 1B.<br>According to the literature spontaneous bleeding events are rare in case of short-lived, Grade 4 thrombocytopenia. Grade 4 events that have no clinically relevant bleeding complications, are of limited clinical relevance and should not be considered as dose limiting toxicities. Therefore, a protocol modification is proposed to define only long-lived Grade 4 thrombocytopenia (lasting ≥7 days) or those events complicated with clinically relevant bleeding as DLTs. This modification was discussed and endorsed by the SMC members and participating investigators.
6.5.5 Dose Modification	• Clarified the management of neutropenia with G-CSF.	Clarification/consequence of the introduction of Part 1B.
6.8.2 Permitted Medicines	• Updated to allow use of G-CSF during the DLT observation period for patients who have developed neutropenia in Part 1A. Also, for patients who have experienced Grade ≥ 3 neutropenia and/or febrile neutropenia prophylactic administration of G-CSF is permitted for subsequent cycles according to physician's discretion.	Clarification/consequence of the introduction of Part 1B.
6.8.3 Prohibited Medicines	• Specified that prophylactic use of G-CSF is not permitted during the DLT observation period in Part 1A.	Clarification/consequence of the introduction of Part 1B.
8.2.3 Electrocardiograms	• Specified ECG collection.	Minor corrections for clarification of ECG collection.
8.4 Pharmacokinetics	• Specified PK urine sample collection.	Minor correction for clarification of PK urine sample collection
9.2 Sample Size Determination	• Updated sample size to include Part 1B dose escalation cohorts.	To reflect the additional Part 1B cohorts.
9.4 Statistical Analyses	• Specified that separate BLRMs will be used for Part 1A and Part 1B.	To describe how Part 1A and Part 1B will be analyzed.
9.4.2.1 Dose Escalation	• Specified the allowed dose increases in Part 1B for the SMC recommendations.<br>• Added that separate BLRMs will be used for Part 1A and Part 1B but with the same specifications and prior parameter settings.	To describe how the dose increases will be calculated and how Part 1A and Part 1B will be analyzed.

Section # and Name	Description of Change	Brief Rationale
Appendix 2. Study Governance	• Added scientific rationale for submission of the summary of clinical study results beyond 1 year from the end of the clinical trial in all EU member states	To clarify submission of the summary of clinical study results
Appendix 6. Clinical Laboratory Tests	• Added calcium corrected.<br>• CCI [redacted].<br>• Specified Laboratory Test on C2D15.	Minor correction for clarification.
Appendix 8. Model for Bayesian Dose Escalation	• Added preselected dose levels for Part 1B dose escalation.	To specify the preselected dose levels for Part 1B.
Appendix 12. Sponsor Signature Page	• Changed the name of the Sponsor representative.	To reflect the change in staff responsibilities.
Throughout	• Minor editorial and document formatting revisions.	Minor; therefore, have not been summarized.

Protocol Version 2.2 (27 June 2022)

Overall Rationale for the Amendment (vs. Protocol Version 2.0)

The protocol has been amended to address PMDA's comments prior to implementation.

Section # and Name	Description of Change	Brief Rationale
9.4.2.1 Dose Escalation	Added criteria for dose escalation	• To specify the criteria that the SMC will consider for the dose escalation
Appendix 12: Japan-specific Requirements	Added appendix listing country-specific requirements	• To include wording on ILD risk mitigation, inclusion of BRAF-mutant mCRC, hepatitis B testing, criteria for study discontinuation and early termination, and hospitalization during Cycle 1.<br>• To specify which hematopoietic growth factors and contraceptive measures are or are not approved in Japan.
Appendix 13: Sponsor Signature Page	Updated with current Sponsor Clinical Lead	• To reflect the change in Sponsor Clinical Lead for the protocol
Throughout	Minor editorial and document formatting revisions	• Minor; therefore, have not been summarized.

Protocol Version 2.1 US (24 May 2022)

Overall Rationale for the Amendment (vs. Protocol Version 2.0)

The protocol has been amended to incorporate minor editorial changes and corrections in addition to FDA's feedback prior to implementation.

Section # and Name	Description of Change	Brief Rationale
1.1. Synopsis<br>4.1 Overall Design	Added that duration of infusion is approximately 1 hour.	To specify the recommended duration of infusion of M9140.
4.1 Overall Design<br>4.2 Scientific Rationale for Study Design<br>6.5.2 Dose Selection<br>6.5.3 Safety Monitoring Committee<br>9.4.3.2.1 Dose Escalation<br>Appendix 8 Model for Bayesian Dose Escalation<br>Appendix 10. Potential Variations in Study Intervention Schedules and Schedules of Activities	Deleted the schedule of assessments for potential alternative schedules and related wording throughout the protocol.	Alternative schedules will be evaluated only upon approval of a protocol amendment by the competent Health Authority.
4.1 Overall Design<br>5 Study Population<br>5.1 Inclusion Criteria<br>5.2 Exclusion Criteria	Revised patient population to exclude patients with HER2-positive GC/[redacted] who are eligible for trastuzumab deruxtecan treatment. Patients with HER2-positive GC/[redacted] are eligible for participation in this study only if they are not eligible for treatment with trastuzumab deruxtecan or trastuzumab deruxtecan is not available for treatment in their country.	Patients who are eligible to receive trastuzumab deruxtecan would not forgo an approved treatment option.
5.1 Inclusion Criteria	Deleted serum creatinine level from the inclusion criteria and adapted the creatinine clearance threshold to ≥ 60 mL/min.	Renal function will be assessed solely based on creatinine clearance and patients with moderate renal impairment are excluded.
5.3.1 Meals and Dietary Restrictions	Added restriction for concomitant use of foods or drinks with CYP3A4 inhibition potential (i.e., grapefruits and grapefruit juice, Seville oranges, pomelos, starfruits).	The risk of interaction of M9140 with CYP3A4 inhibitors from foods or drinks is considered low but cannot be completely excluded.
6.5.4 Definition of Dose-limiting Toxicity	Modified DLT criteria and clarified the wording in the protocol.	Updated to differentiate between Grade 3 and Grade > 3 events.
6.5.5 Dose Modification	Dose modifications and management of infusion-related reactions added.	IRRs are defined as potential risks in the protocol and management of risks was missing.

Section # and Name	Description of Change	Brief Rationale
6.8.3 Prohibited Medicines	Restricted the concomitant use of strong CYP3A4 and CYP1A2 inducers in Part 2 of the study.	Allowance of strong CYP3A4 and CYP1A2 inducers in Part 2 may affect safety evaluation of M9140.
[redacted]		
Throughout	Minor editorial and document formatting revisions.	Minor; therefore, have not been summarized.

Protocol Version 2.0 (24 May 2022)

Overall Rationale for the Amendment

The protocol has been amended to incorporate minor editorial changes and corrections prior to implementation.

Section # and Name	Description of Change	Brief Rationale
1.1. Synopsis<br>4.1 Overall Design	Added that duration of infusion is approximately 1 hour.	To specify the recommended duration of infusion of M9140.
4.1 Overall Design<br>5 Study Population<br>5.1 Inclusion Criteria<br>5.2 Exclusion Criteria	Revised patient population to exclude patients with HER2-positive GC/[redacted] who are eligible for trastuzumab deruxtecan treatment. Patients with HER2-positive GC/[redacted] are eligible for participation in this study only if they are not eligible for treatment with trastuzumab deruxtecan or trastuzumab deruxtecan is not available for treatment in their country.	Patients who are eligible to receive trastuzumab deruxtecan would not forgo an approved treatment option.
5.1 Inclusion Criteria	Deleted serum creatinine level from the inclusion criteria and adapted the creatinine clearance threshold to ≥ 60 mL/min.	Renal function will be assessed solely based on creatinine clearance and patients with moderate renal impairment are excluded.
5.3.1 Meals and Dietary Restrictions	Added restriction for concomitant use of foods or drinks with CYP3A4 inhibition potential (i.e., grapefruits and grapefruit juice, Seville oranges, pomelos, starfruits).	The risk of interaction of M9140 with CYP3A4 inhibitors from foods or drinks is considered low but cannot be completely excluded.
6.5.4 Definition of Dose-limiting Toxicity	Modified DLT criteria and clarified the wording in the protocol.	Updated to differentiate between Grade 3 and Grade > 3 events.
6.5.5 Dose Modification	Dose modifications and management of infusion-related reactions added.	IRRs are defined as potential risks in the protocol and management of risks was missing.

Section # and Name	Description of Change	Brief Rationale
6.8.3 Prohibited Medicines	Restricted the concomitant use of strong CYP3A4 and CYP1A2 inducers in Part 2 of the study.	Allowance of strong CYP3A4 and CYP1A2 inducers in Part 2 may affect safety evaluation of M9140.
[redacted]		
Appendix 10. Table 19. Schedule of Assessments for Part 1 – Dose Escalation [redacted]	Added vital signs assessments in C1D2 and C1 D5 and deleted 12-lead safety ECG at CxD8 (C2, C3, C4, C5, C6).	To correct inconsistencies with the other dose escalation schedules.
Throughout	Minor editorial and document formatting revisions.	Minor; therefore, have not been summarized.

Appendix 11 Country-specific Requirements

EU-specific Requirements

EU-specific requirements are listed below together with the applicable protocol sections.

Section 6.1 Study Intervention Administered

According to EU Regulation 536/2024 (Annex VI, A1, paragraph 3), "The address and telephone number of the main contact shall not be required to appear on the label if subjects have been given a leaflet or card which provides these details and have been instructed to keep this in their possession at all times."

A Patient ID card is used in this study which contains information related to address and telephone number of the main contact for this study.

Section 8.3.3 Regulatory Reporting Requirements for Serious Adverse Events

For studies in EU/EEA and submitted under the EU Clinical Trial Regulation 536/2014, SUSARs will need to be reported centrally via the EV database to HA by the Sponsor.

Section 8.3 Adverse Events, Serious Adverse Events, and Other Safety Reporting

Definition of new fact(s):

Any new data that could lead to:

- the reassessment of the relationship between the benefits and risks of the research or the experimental drug
- changes in the use of this medicine, in the conduct of research, or in documents relating to research
- suspend or interrupt or modify the protocol of the research or similar research.

A new fact may also correspond to a suspicion of an unexpected serious adverse reaction (EIGI or SUSAR).

Japan-specific Requirements

Japan-specific requirements are listed below together with the applicable protocol sections.

Sections 2.3.1 Risk Assessment, 6.5.5 Dose Modification, and 8.2 Safety Assessments and Procedures, Appendix 6

Risk Management for Interstitial Lung Disease

The following tests will be performed for screening of ILD and early detection after the start of treatment with this drug.

- Sialylated carbohydrate antigen (KL-6) will be measured at the time of periodic blood collection.
- Percutaneous arterial oxygen saturation (SpO2) will be performed at vital sign assessments.
- Auscultation will be performed at physical examinations
- For imaging tests, consider imaging tests such as chest X-ray and/or CT if clinically indicated.

Sections 4.1 Overall Design, 5 Study Population, and 5.1 Inclusion Criteria

Part 1 - Dose Escalation AND Part 2 - Dose Expansion

Participants with BRAF gene mutations must have received [redacted] or [redacted].

Section 5.2 Exclusion Criteria

9. In Japan, participants are excluded if HbsAg+ or HBV DNA is ≥ 20 IU/mL (if HbcAb+ and/or HbsAb+). If HBV DNA is < 20 IU/mL for the patients who are HbcAb+ and/or HbsAb+, HBV DNA will be monitored during the study according to the "Guideline for prevention of immunosuppressive therapy/chemotherapy-induced reactivation of hepatitis B virus."

17. History of severe hypersensitivity reactions to prior therapies with biologicals or ingredients of M9140 (active substance and excipients).

Section 6.8.2 Permitted Medicines

[redacted].

Sections 8.2 Safety Assessments and Procedures, 8.4 Pharmacokinetics, and Table 2

Hospitalization

The participant will be hospitalized for at least 24 hours after the completion of M9140 administration in Cycle 1 in Part 1 of the study to allow for observation of possible acute reactions, including IRRs, and to facilitate frequent blood sampling.

Provided that during Cycle 1 no unexpected, clinically relevant toxicity is observed, the proposed study does not require patients to be hospitalized.

Section 9.4.2.1 Dose Escalation

If after testing the starting dose (0.6 mg/kg) in the first cohort, the probability of P(DLT) ≥ 35% is estimated to be above 65% for the dose below the starting dose (e.g., 0.3 mg/kg) according to the Bayesian model, the dose escalation will be withheld in Japan without going to a lower dose and the Sponsor will consult the PMDA for the next steps. This is for example the case, if there are 2 DLTs in 3 participants in the first cohort on the starting dose.

Only in case after investigating the starting dose (0.6 mg/kg) and the estimated probability for P(DLT) at the lower dose (e.g., 0.3 mg/kg) is ≤ 65% according to the Bayesian model, can the lower dose be tested. This is for example the case if there are 1/3 or 3/6 DLTs or less on the starting dose. If after testing the dose below the starting dose (e.g., 0.3 mg/kg), the probability of P(DLT) ≥ 35% is estimated to be above 65% for this dose, dose escalation will be terminated early in Japan.

Section 5.1 Inclusion Criteria and Appendix 3 Contraception and Barrier Requirements.

The following highly effective contraception methods are not approved in Japan:

- Implantable progestogen-only hormone contraception associated with inhibition of ovulation
- Combined (estrogen- and progestogen containing) hormonal contraception associated with inhibition of ovulation:
    - Intravaginal
    - Transdermal
    - Injectable
- Progestogen-only hormone contraception associated with inhibition of ovulation:
    - Injectable
    - Oral

The following barrier methods are not approved in Japan:

- Female condom with or without spermicide
- Cap, diaphragm, or sponge with spermicide

Appendix 12 NCI-PRO-CTCAE® Custom Survey

Item subset derived from PRO-CTCAE® Item Library Version 1.0
(https://healthcaredelivery.cancer.gov/pro-ctcae/builder.html)
English; Form Created on 08 December 2023

As individuals go through treatment for their cancer they sometimes experience different symptoms and side effects. For each question, please select the one response that best describes your experiences over the past 7 days.

**1a. In the last 7 days, what was the SEVERITY of your MOUTH OR THROAT SORES at their WORST?**				
O None	O Mild	O Moderate	O Severe	O Very severe
**1b. In the last 7 days, how much did MOUTH OR THROAT SORES INTERFERE with your usual or daily activities?**				
O Not at all	O A little bit	O Somewhat	O Quite a bit	O Very much
**2a. In the last 7 days, how OFTEN did you have NAUSEA?**				
O Never	O Rarely	O Occasionally	O Frequently	O Almost constantly
**2b. In the last 7 days, what was the SEVERITY of your NAUSEA at its WORST?**				
O None	O Mild	O Moderate	O Severe	O Very severe
**3a. In the last 7 days, how OFTEN did you have VOMITING?**				
O Never	O Rarely	O Occasionally	O Frequently	O Almost constantly
**3b. In the last 7 days, what was the SEVERITY of your VOMITING at its WORST?**				
O None	O Mild	O Moderate	O Severe	O Very severe
**4a. In the last 7 days, how OFTEN did you have LOOSE OR WATERY STOOLS (DIARRHEA/DIARRHOEA)?**				
O Never	O Rarely	O Occasionally	O Frequently	O Almost constantly
**5a. In the last 7 days, what was the SEVERITY of your FATIGUE, TIREDNESS, OR LACK OF ENERGY at its WORST?**				
O None	O Mild	O Moderate	O Severe	O Very severe
**5b. In the last 7 days, how much did FATIGUE, TIREDNESS, OR LACK OF ENERGY INTERFERE with your usual or daily activities?**				
O Not at all	O A little bit	O Somewhat	O Quite a bit	O Very much

The PRO-CTCAE® items and information herein were developed by the Division of Cancer Control and Population Sciences in the NATIONAL CANCER INSTITUTE at the NATIONAL INSTITUTES OF HEALTH, in Bethesda, Maryland, U.S.A. Use of the PRO- CTCAE® is subject to NCI's Terms of Use.

Appendix 13 Sponsor Signature Page

**Study Title:**	A Phase 1, Two-Part, Multicenter, Open-Label First-in-Human Study of anti-CEACAM5 Antibody-Drug Conjugate M9140 in Participants with Advanced Solid Tumors
**Regulatory Agency Identifying Numbers:**	US FDA IND 156588<br>EU trial number: 2022-500508-23-00
**Clinical Study Protocol Version:**	29 July 2024/Version 5.0

I approve the design of the clinical study:

______________________________ ________________________

Signature Date of Signature

**Name, academic degree:**	PPD, MD, PhD
**Function/Title:**	PPD
**Institution:**	Merck Healthcare KGaA
**Address:**	Frankfurter Str. 250, 64293 Darmstadt, Germany
**General Merck Phone Number:**	+49 (0) 6151720
**General Merck Fax Number:**	+49 (0) 6151722000

Appendix 14 Coordinating Investigator Signature Page

**Study Title:**	A Phase 1, Two-Part, Multicenter, Open-Label First-in-Human Study of anti-CEACAM5 Antibody-Drug Conjugate M9140 in Participants with Advanced Solid Tumors
**Regulatory Agency Identifying Numbers:**	US FDA IND 156588<br>EU trial number: 2022-500508-23-00
**Clinical Study Protocol Version:**	29 July 2024/Version 5.0

I approve the design of the clinical study, am responsible for the conduct of the study at this site and understand and will conduct it per the clinical study protocol, any approved protocol amendments, ICH GCP (Topic E6) and all applicable Health Authority requirements and national laws.

______________________________ ________________________

Signature Date of Signature

**Name, academic degree:**	PPD, MD, PhD, FACP
**Function/Title:**	PPD
**Institution:**	PPD<br>[redacted]<br>The University of Texas MD Anderson Cancer Center
**Address:**	1515 Holcombe Blvd.<br>Houston, Texas 77030, US
**Telephone number:**	PPD
**Fax number:**	Not Applicable
**E-mail address:**	PPD

Appendix 15 Principal Investigator Signature Page

**Study Title:**	A Phase 1, Two-Part, Multicenter, Open-Label First-in-Human Study of anti-CEACAM5 Antibody-Drug Conjugate M9140 in Participants with Advanced Solid Tumors
**Regulatory Agency Identifying Numbers:**	US FDA IND 156588<br>EU trial number: 2022-500508-23-00
**Clinical Study Protocol Version:**	29 July 2024/Version 5.0
**Site Number:**	

I am responsible for the conduct of the study at this site and understand and will conduct it per the clinical study protocol, any approved protocol amendments, ICH GCP (Topic E6) and all applicable Health Authority requirements and national laws.

I also understand that Health Authorities may require the Sponsors of clinical studies to obtain and supply details about ownership interests in the Sponsor or Investigational Medicinal Product and any other financial ties with the Sponsor. The Sponsor will use any such information solely for complying with the regulatory requirements. Therefore, I agree to supply the Sponsor with any necessary information regarding ownership interest and financial ties including those of my spouse and dependent children, and to provide updates as necessary to meet Health Authority requirements.

Signature Date of Signature

Name, academic degree:

Function/Title:

Institution:

Address:

Telephone number:

Fax number:

E-mail address:
