# Supplementary material for: Precemtabart tocentecan, an anti-CEACAM5 antibody–drug conjugate, in metastatic colorectal cancer: a phase 1 trial
Source: Nat Med. 2025 Jul 30;31(10):3504–13. doi: 10.1038/s41591-025-03843-z (PMC12532702; doi:10.1038/s41591-025-03843-z)
Supplement: Supplementary file 2 — Reporting Summary [file 41591_2025_3843_MOESM2_ESM.pdf]

nature portfolio

Corresponding author(s): Scott Kopetz

Last updated by author(s): May 26, 2025

Reporting Summary

Nature Portfolio wishes to improve the reproducibility of the work that we publish. This form provides structure for consistency and transparency in reporting. For further information on Nature Portfolio policies, see our Editorial Policies and the Editorial Policy Checklist.

Statistics

For all statistical analyses, confirm that the following items are present in the figure legend, table legend, main text, or Methods section.

n/a	Confirmed	
☐	☒	The exact sample size (*n*) for each experimental group/condition, given as a discrete number and unit of measurement
☐	☒	A statement on whether measurements were taken from distinct samples or whether the same sample was measured repeatedly
☐	☒	The statistical test(s) used AND whether they are one- or two-sided<br>*Only common tests should be described solely by name; describe more complex techniques in the Methods section.*
☒	☐	A description of all covariates tested
☒	☐	A description of any assumptions or corrections, such as tests of normality and adjustment for multiple comparisons
☐	☒	A full description of the statistical parameters including central tendency (e.g. means) or other basic estimates (e.g. regression coefficient) AND variation (e.g. standard deviation) or associated estimates of uncertainty (e.g. confidence intervals)
☒	☐	For null hypothesis testing, the test statistic (e.g. *F*, *t*, *r*) with confidence intervals, effect sizes, degrees of freedom and *P* value noted<br>*Give P values as exact values whenever suitable.*
☐	☒	For Bayesian analysis, information on the choice of priors and Markov chain Monte Carlo settings
☒	☐	For hierarchical and complex designs, identification of the appropriate level for tests and full reporting of outcomes
☒	☐	Estimates of effect sizes (e.g. Cohen's *d*, Pearson's *r*), indicating how they were calculated

Our web collection on statistics for biologists contains articles on many of the points above.

Software and code

Policy information about availability of computer code

Data collection	INFORM (version number 7.0.0.1.41 - 64bit)
Data analysis	1. GraphPad Prism software (version 10.2.0, La Jolla, CA, USA)<br>2. Microsoft Excel (version 16.0, Microsoft Corporation, Redmond, WA, USA)<br>3. FlowJo software (version 10.9.0, BD Life Sciences)<br>4. CQ1 software (version 1.04.02.04, Yokogawa)<br>5. Data analysis software for PK/Pd: Phoenix® WinNonlin® version 6.4 or higher.<br>6. Graphical explorations were performed using R (version 4.2.0 or higher, R Project for Statistical Computing, Vienna, Austria<br>7. Clinical data analysis with R, version 4.2.1

For manuscripts utilizing custom algorithms or software that are central to the research but not yet described in published literature, software must be made available to editors and reviewers. We strongly encourage code deposition in a community repository (e.g. GitHub). See the Nature Portfolio guidelines for submitting code & software for further information.

Data

Policy information about availability of data

All manuscripts must include a data availability statement. This statement should provide the following information, where applicable:

- Accession codes, unique identifiers, or web links for publicly available datasets
- A description of any restrictions on data availability
- For clinical datasets or third party data, please ensure that the statement adheres to our policy

Any requests for data by qualified scientific and medical researchers for legitimate research purposes will be subject to the Data Sharing Policy of Merck. Following completion of the study and finalization of the clinical study reports, de-identified patient data and the statistical analysis plan can be provided upon reasonable request from qualified researchers. Access to the data is controlled for privacy, ethical and compliance reasons. All requests should be submitted in writing to the data sharing portal for Merck: Clinical Trial Data Sharing - Research | Merck Global (https://www.merckgroup.com/en/research/our-approach-to-research-and-development/healthcare/clinical-trials/commitment-responsible-data-sharing.html). When Merck has a co-research, co-development, or co-marketing or co-promotion agreement, or when the product has been out-licensed, the responsibility for disclosure might be dependent on the agreement between parties. Under these circumstances, Merck will endeavor to gain agreement to share data in response to requests. The antibody sequence of precem-TCT has been published57 and is included in the Supplementary Information file. A patent application describing precem-TCT has been filed (published international patent application WO 2022/048883 A1).

Research involving human participants, their data, or biological material

Policy information about studies with human participants or human data. See also policy information about sex, gender (identity/presentation), and sexual orientation and race, ethnicity and racism.

Reporting on sex and gender	Data are reported as male or female in Table 1; sex and gender-based analyses were not part of the analysis plan for this Phase 1 trial; hence outcomes based on sex and/or gender are not reported.
Reporting on race, ethnicity, or other socially relevant groupings	Data are reported as White, Asian, Black or African American, or Other. Race-based analyses were not part of the analysis plan for this Phase 1 trial; hence only aggregated analyses are reported.
Population characteristics	Table 1 in the manuscript provides detailed information on baseline and demographic characteristics of the patients included in the Phase 1 trial.
Recruitment	Patients with documented histopathological diagnosis of locally advanced or metastatic CRC, who were intolerant/refractory to, or progressed after three lines of standard systemic therapies were recruited. Five study sites in three countries were selected based on availability of patients and investigator expertise. Patients were recruited if they met selection criteria and provided informed consent. Attempts to limit bias included the use of Interactive Response Technology (IRT; Cenduit®), which was used to assign unique participant numbers and allocate study intervention to participants at each study intervention visit. Participant assignment to Parts 1A or 1B were sequential, as per dose cohorts opened by SMC decision. Detailed information on methods to reduce bias are provided in the redacted protocol.
Ethics oversight	The study protocol, amendments, and informed consent forms were reviewed and approved by the Institutional Review Board/Independent Ethics Committee at each study site before study initiation.<br>1. NEXT US – approved 14 Jun 2022<br>2. MDACC US – approved 30 Jun 2022<br>3. VHIO Spain – approved 29 Aug 2022 (decision 15 Sep 2022)<br>4. NEXT Spain – approved 20 Aug 2022 (decision 15 Sep 2022)<br>5. NCC Japan – approved 26 Aug 2022

Note that full information on the approval of the study protocol must also be provided in the manuscript.

Field-specific reporting

Please select the one below that is the best fit for your research. If you are not sure, read the appropriate sections before making your selection.

☒ Life sciences ☐ Behavioural & social sciences ☐ Ecological, evolutionary & environmental sciences

For a reference copy of the document with all sections, see nature.com/documents/nr-reporting-summary-flat.pdf

Life sciences study design

All studies must disclose on these points even when the disclosure is negative.

Sample size	This study employed a Bayesian dose-escalation design. The first dose-escalation cohort, consisting of three patients, and subsequent dose escalations proceeded according to the recommendations of the Safety Monitoring Committee (SMC), which were based on available safety and preliminary PK data and supported by a Bayesian two-parameter logistic regression model

Data exclusions	No data were excluded
Replication	Preclinical data are from at least 2 independent experiments. Detailed information on replicates for each experiment are provided as part of the figure legend. Assessments were made on individual patient samples and no replication was possible.
Randomization	This was a non-randomised study
Blinding	This was an open-label study

Reporting for specific materials, systems and methods

We require information from authors about some types of materials, experimental systems and methods used in many studies. Here, indicate whether each material, system or method listed is relevant to your study. If you are not sure if a list item applies to your research, read the appropriate section before selecting a response.

Materials & experimental systems

n/a	Involved in the study	
☐	☒	Antibodies
☐	☒	Eukaryotic cell lines
☒	☐	Palaeontology and archaeology
☐	☒	Animals and other organisms
☐	☒	Clinical data
☒	☐	Dual use research of concern
☒	☐	Plants

Methods

n/a	Involved in the study	
☒	☐	ChIP-seq
☒	☐	Flow cytometry
☒	☐	MRI-based neuroimaging

Antibodies

Antibodies used	1. Human anti-CEACAM5 primary antibody (antibody moiety of M9140), produced internally<br>2. Donkey anti-human IgG fluorescently (phycoerythrin)-labeled secondary antibody (Jackson ImmunoResearch #709 116 149)<br>3. Monoclonal antibody intermediate of tusa.rav.a (an analog of tusamitamab ravtansine)
Validation	The only primary antibody used is human anti-human CEACAM5 antibody which was internally produced. Specificity and species cross-reactivity including affinity measurements and binding domain determination were assessed as part of the investigational new drug (IND) package.

Eukaryotic cell lines

Policy information about cell lines and Sex and Gender in Research

Cell line source(s)	SK-CO-1 ATCC<br>MKN-45 DSMZ<br>MDA-MB-231 ATCC<br>LS513 ATCC<br>LoVo ATCC
Authentication	SK-CO-1 ATCC myco neg STR ID confirmed by STR<br>MKN-45 DSMZ myco neg STR ID confirmed by STR<br>MDA-MB-231 ATCC myco neg STR ID confirmed by STR<br>LS513 ATCC myco neg STR ID confirmed by STR<br>LoVo ATCC myco neg STR ID confirmed by STR
Mycoplasma contamination	All cell lines were tested negative for mycoplasma contamination and identity was confirmed by STR analysis, sources ATCC and DSMZ
Commonly misidentified lines<br>(See ICLAC register)	None of the cell lines used in the study are listed as commonly misidentified cell line(s) in the source database.

Animals and other research organisms

Policy information about studies involving animals; ARRIVE guidelines recommended for reporting animal research, and Sex and Gender in Research

Laboratory animals	1. Male/female cynomolgous monkeys: purpose-bred, naïve Vietnamese cynomolgus monkeys were purchased from Envigo, Horst, The Netherlands. Purchasing, source, housing conditions, bodyweight and age followed recommendations of the AAALAC.<br>2. Immunodeficient female mice (NMRI nu/nu mice; NMRI nude, Charles River, Sulzfeld, Germany) were 4-6 weeks old. Animals were housed in individually ventilated cages (TECNIPLAST Sealsafe-IVC-System, TECNIPLAST, Hohenpeissenberg, Germany), depending on

	group size, either in type III or type II long cages. They were kept under a 14L:10D artificial light cycle. The temperature inside the cages was maintained at 22 –26 °C with a relative humidity of 45–65% and 60–65 air changes/hour in the cage. Dust-free bedding consisting of aspen wood chips with approximate dimensions of 5 mm × 5 mm × 1 mm (ABEDD, LAB & VET Service GmbH, Vienna, Austria, product code: LTE E-001) and additional nesting material were used. The cages including the bedding and the nesting material were changed weekly. The animals were fed autoclaved Teklad Global Extruded 19% Protein Rodent Diet from Envigo RMS SARL and hadaccess to sterile filtered and acidified (pH 2.5) tap water that was changed twice weekly. Feed and water were provided ad libitum. All materials were autoclaved prior to use.<br>3. Immunodeficient female mice NMRI nu/nu mice male/female NU/NU (Crl:NUFoxn1nu, Beijing Vital River Laboratories, China) were 6-7 weeks old when used in experiments. Animal holding rooms were maintained at 20-26°C and 40-70% humidity. Lights were on a 12 hour light/dark cycle. Mice were housed in an AAALAC-accredited SPF facility, and all efforts were made to minimize pain and distress.
Wild animals	Study did not involve wild animals
Reporting on sex	Both male and female cynomolgous monkeys and immunodeficient female mice were used, and data on sex and/or gender of animals used are reported as aggregated data.
Field-collected samples	No field-collected samples were used in this study.
Ethics oversight	1. All procedures with cynomolgus monkeys were in compliance with the Animal Welfare Act(s) following the recommendations of the AAALAC and national Animal Health regulations and animal ethics approval of study protocols by the Institutional Animal Care Use Committee of Merck KGaA (Darmstadt, Germany), and Istituto di Ricerche Biomediche "Antoine Marxer"–RBM S.p.A. (Ivrea, Italy).<br>2. Experiments with immunodeficient female mice (NMRI nu/nu mice) were conducted according to all applicable international, national and local laws and followed the national guidelines for the Care and Use of Laboratory Animals of the Society of Laboratory Animal Science (GV-SOLAS). All animal experiment protocols were approved by the regional council Committee on the Ethics of Animal Experiments. Genehmigungsnummer: G-20/163 Subkutane Tumor-Xenograft-Modelle (19.03.2021 - 18.03.2026) (G-20/163).<br>3. Experiments with NU/NU (Crl:NU-Foxn1nu) mice were conducted in compliance with the Guide for the Care and Use of Laboratory Animals. Protocols were reviewed and approved by the Institutional Animal Care and Use Committee (IACUC) of Xi'an LIDE Biotech Co., Ltd. (AAALAC Unit #001541, Approval No. LDIACUC001, approved on 25 July 2015).

Note that full information on the approval of the study protocol must also be provided in the manuscript.

Clinical data

Policy information about clinical studies
All manuscripts should comply with the ICMJE guidelines for publication of clinical research and a completed CONSORT checklist must be included with all submissions.

Clinical trial registration	NCT05464030
Study protocol	Trial protocol is part of the submission package
Data collection	Data were collected via eCRF at clincial study sites on individual patient basis. Recruitment period was August 2022 to February 2024. Data collection per patient was done for treatment until PD, including safety follow-up visit 30 days after last dose. Detailed information is provided in the schedule of activities in the protocol submitted.
Outcomes	The primary objectives of the study were to determine 1) the dose–toxicity relationship and the maximum tolerated dose (MTD, if reached) of M9140 based on the occurrence of DLTs and AEs and 2) the recommended doses for expansion (RDEs) of M9140, based on its safety, PK profile, and preliminary clinical activity.<br><br>Secondary objectives included characterization of the PK profile of M9140 (conjugated antibody, total antibody, and unconjugated exatecan payload) and evaluation of clinical activity indicators for M9140, specifically the objective response (with objective response rate [ORR] defined as the proportion of patients achieving a confirmed best overall response of complete response [CR] or partial response [PR]), duration of response, and mPFS, all assessed by the investigator using the Response Evaluation Criteria in Solid Tumors, version 1.1. In addition, the disease control rate (DCR) at 12 weeks was assessed, defined as the proportion of patients achieving CR, PR, stable disease, or non-CR/non-PD at the Week 12 visit (or later) prior to documented PD.

Plants

Seed stocks	Not applicable
Novel plant genotypes	Not applicable
Authentication	Not applicable
